# Enabling molecular signaling with temperature and ionic-strength independence or programmable dependence

**DOI:** 10.1038/s41467-026-76207-x

**Published:** 2026-08-07

**Authors:** Egor S. Korenkov, Maxim P. Nikitin

**Affiliations:** 1 Moscow Center for Advanced Studies, Moscow, Russia; 2 Abisense LLC, Sirius, Krasnodar region Russia; 3 Sirius University of Science and Technology, Sirius, Krasnodar region Russia

**Keywords:** Biochemical reaction networks, Biotechnology, Cell signalling, Origin of life

## Abstract

The equilibrium constants of chemical reactions fundamentally depend on temperature, posing challenges for living systems. However, many conformer organisms do not maintain a stable internal temperature. This raises the question: can molecular signaling pathways inherently resist temperature susceptibility? Molecular commutation is a recently discovered, fundamentally distinct mechanism of biological information processing and storage within reversible association/dissociation reactions. Here, we show that molecular commutation enables complex signaling systems that are independent of temperature and ionic strength and, even more generally, programmably dependent on these parameters. Using examples of various DNA logic gates, receptor-activator networks, and systems with complex input–output relationships (e.g., computed as algebraic functions), we demonstrate computationally that introducing compensatory reactions in these networks can render their signaling independent of temperature and ionic strength. We experimentally validate such independence for a case of a YES-logic gate. Finally, we computationally demonstrate networks with outputs that follow predefined functional forms of temperature and ionic strength (e.g., sin(T), where T is temperature). The presented intrinsic capabilities of affinity-based networks provide a remarkable homeostasis and signaling control mechanism that may be used by biological systems of arbitrarily high complexity.

## Abstract (web summary)

Environmental factors such as temperature and ionic strength can strongly influence molecular signaling pathways. Here, the authors show that the principle of molecular commutation allows the design of affinity-based networks that either compensate for or exploit these effects, achieving robust or programmable signaling behaviors across diverse computationally modeled systems and a wet-lab-validated DNA logic gate.

## Introduction

Almost all chemical reactions depend on temperature, typically following an exponential relationship of the form ${e}^{-\frac{E}{{kT}}}$. This principle plays a critical role in biological systems, as even small variations in temperature can lead to significant changes in the parameters of biochemical reactions^1^, potentially resulting in severe physiological consequences at the organismal level. It is therefore unsurprising that many species have evolved complex homeostatic mechanisms to maintain a stable internal temperature. However, a broad range of organisms do not exhibit temperature homeostasis. For instance, numerous pathogenic bacteria^2^ experience temperature shifts of approximately 30 °C during their transition from environmental reservoirs, such as soil, to the human host. Similarly, many eurythermal fish species, such as *Cyprinus carpio*^3^ and *Fundulus heteroclitus*^4^, are capable of thriving in environments ranging from near-freezing (~ 3 °C) to warm summer waters (~ 35 °C). Moreover, even humans can tolerate prolonged exposure (hours to days) of extremities to near-freezing temperatures without significant harm, provided moisture is absent^5^.

A question arises as to how, in all these cases, the complex regulatory and signaling networks of biochemical reactions, which form the basis of any living system, continue to properly function, given that the parameters of all the constituent reactions vary dramatically^6,7^.

In general, signal transduction and processing using molecular systems have long been the subject of active investigation^8,9^. In fact, to date, a large number of biocomputing approaches, i.e., artificial molecular systems that can process data and various signals, have been developed based on enzymatic networks^10^, nucleic acids^11,12^, nanoscale structures^13,14^, among many others. However, most of these systems could hardly have evolved in nature as they require rather specific and unnatural design of participating molecules or nanostructures.

On the other hand, numerous studies have identified and characterized concrete examples of computational pathways in living organisms, including interacting protein networks^15^, as well as instances of biocomputation at the level of large receptor systems^16^ or even entire cells^17^. In such systems, one of the key factors is the presence of “signal integrators”: elements that combine multiple inputs into a single output. For example, in the olfactory receptor system^16^ the integrator is the central nervous system that analyzes signals from many receptors (inputs) to give the sense of a certain smell (a single perceptual output). Similarly, the dimerizing transcription factors system^15^ requires the presence of a specific “integrator” enzyme, which can be phosphorylated or dephosphorylated by multiple network components (inputs) with a very particular single output: the concomitant change in catalytic activity.

Interestingly, enzymatic networks may be involved in temperature independence in naturally occurring systems under certain conditions^18^, namely when the weighted sum of the activation energies of specific reactions in the network equals zero. This mechanism is considered to contribute to the thermal stability of particular biochemical cascades, such as circadian rhythms^19–21^ or bacterial chemotaxis^22^. However, evolution-driven or artificial design of the enzymatic networks that adhere to such a narrow constraint seems to be highly complex as these systems must additionally develop and retain other critical parameters for each molecule of the network, including substrate affinity, substrate selectivity, catalytic function, reaction rates etc.

In a recent 2023 study^23,24^, we demonstrated the biomolecular information processing principle of molecular commutation by experimentally proving that highly complex information processing tasks can be performed by molecular ensembles solely through their reversible “all-to-all” association/dissociation reactions. Moreover, the study experimentally showed that the outcome of such information processing can have a significant direct impact on the key biological processes, such as translation. Molecular commutation (which has also been later referred to as “computations by competitive dimerization networks”^25^) has a notable distinction from other systems involving promiscuous (many-to-many) interactions^26^. In the molecular commutation regime, the entirety of these complex computations happens via affinity-based interactions of molecules according to the law of mass action. In other words, in this computation paradigm, any molecule can act as a signal integrator, and no higher-level signal integrator systems (such as the abovementioned enzymes or brain cell networks) are required. Currently, this computational principle is rapidly developing: theoretical studies have explored the properties of such computations^25^, while both theoretical and experimental work has demonstrated increasingly complex computations^27–31^.

In this work, we report a fundamentally novel property of molecular commutation networks for signaling in biological systems: these networks can overcome the inherent Boltzmann temperature dependence through a topological compensation feature of these networks. To fully counteract a change in reactions of interest caused by increased temperature or ionic strength, one can expand the network’s topology with compensatory species and adjust their parameters (i.e., interaction affinities and concentrations).

We show that this topological compensation can render a molecular commutation signaling system of any complexity—from simple logic gates to large-scale networks—independent of temperature or ionic strength changes with nearly any desired level of accuracy. Moreover, we show that molecular commutation networks can be engineered to exhibit virtually any complex predefined dependence (e.g., the equilibrium concentration of the output molecule behaves as sin(*T*), where *T* is temperature). This phenomenon may serve as an important mechanism of homeostasis or as a universal means of implementing a programmable response of molecular networks to changes in external or internal conditions.

## Results

Before addressing the general case of programmable temperature dependence, we aim to illustrate the key design principles of such systems using an important particular case—complete temperature independence.

As we noted previously^23,24^, information processing systems based on molecular commutation can be implemented with any type of molecule—proteins, DNA, carbohydrates, small molecules, etc. However, DNA is currently the most convenient model for creating and testing commutation circuitry as the equilibrium affinity constants between oligonucleotides can be easily estimated from their sequences (which is incomparably harder for proteins at the moment^32^).

### Concept of easy-to-melt levels

A well-known fact is that two DNA duplexes may have the same affinity at room temperature, but substantially different melting temperatures (in other words, different affinity at another temperature). More formally, affinity (e.g., dissociation constant, *K*_*d*_) is determined by the Gibbs free energy $\left({K}_{d}={e}^{\frac{\varDelta G}{{RT}}}\right)$, which in turn is related to the enthalpy and entropy of binding $\Delta G=\Delta H-T\Delta S$. The different behavior of *K*_*d*_ at two temperatures arises from the relative independence of enthalpy and entropy. For example, this feature has been exploited to implement heat-induced recycling in DNA biocomputing systems^33,34^.

**Fig. 1:**
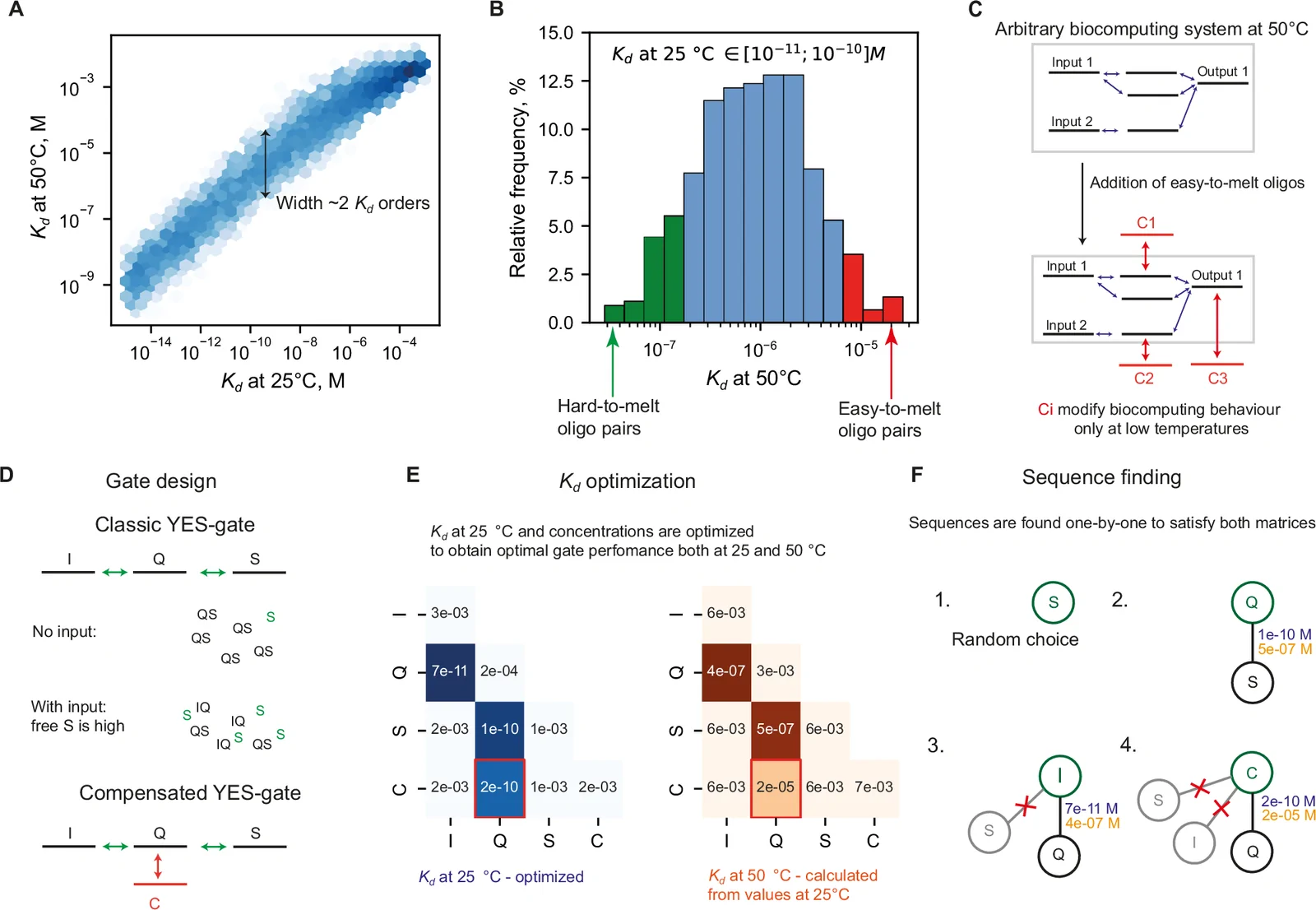
Demonstration of the topological compensation concept. **A** Distribution of dissociation constants (*K*_*d*_) at 25 and 50 °C for 20-nucleotide long oligonucleotides with up to 10 mismatches, as predicted by NUPACK. **B** Distribution of *K*_*d*_ values at 50 °C for oligonucleotide pairs that exhibit *K*_*d*_ values between 10 and 100 pM at 25 °C. **C** General design of a temperature-independent biomolecular signal processing system. **D** Design of a conventional YES gate and its temperature-independent counterpart. **E** Optimization of binding parameters for YES gate components. **F** Overview of the sequence design algorithm. Source data are provided as a Source Data file.

To illustrate the approximate magnitude of this phenomenon, we use NUPACK^35,36^—a widely used tool for the simulation of nucleic acid interactions. Figure 1A shows the distribution of dissociation constants at two arbitrarily chosen temperature points of 25 and 50 °C for a dataset of 10,000 randomly generated oligonucleotide pairs (20-nucleotide-long, 1 to 10 mismatches), as predicted by NUPACK. On average, this temperature increase leads to a rise in *K*_*d*_ by five orders of magnitude, with a variation of approximately two orders of magnitude. Among duplexes with similar stability at 25 °C, we refer to those with stronger binding at 50 °C as hard-to-melt, while those with weaker binding at 50 °C are termed easy-to-melt (Fig. 1B). Importantly, this property is not unique to nucleic acids, and can also be observed, for example, in protein interactions (Supplementary Note 1).

Below, we show that this variety of easy-to-melt and hard-to-melt pairs can be exploited to render a wide range of affinity-based signaling systems temperature-independent. Before proceeding to more complex and challenging cases, we first describe an intuitive strategy for designing simple temperature-independent systems, using logic gates as an example.

Let’s consider such a generic system (Fig. 1C) that operates optimally at 50 °C but exhibits altered behavior at lower temperatures. We can introduce new participants into this system that are extremely easy-to-melt, but still exhibit strong binding at 25 °C. These compensators primarily influence system behavior at lower temperatures, with minimal effects at elevated temperatures. If the parameters of these compensatory reactions are then optimized so that the system functions as intended at low temperature, its performance becomes effectively temperature-independent, despite the fact that all individual reaction components are themselves sensitive to temperature changes.

### Computational design of temperature-independent logic gates

As a specific example of this concept, we turn to the molecular YES gate, following the original work on molecular commutation^24^ (Fig. 1D). This system consists of three oligonucleotides, I (input), Q (quencher) and S (signal). The logical input is defined as 0 when I is absent and 1 when I is present at a fixed concentration. The oligonucleotide S serves as the output. The logical output is considered 0 when the concentration of single-stranded S is below a defined threshold, and 1 when it exceeds this threshold.

Now, when I is absent, Q binds to S, leaving almost no free S. When I is present, it competes for Q, occupying it, leaving much more free S. In these terms, the system behaves like a Boolean function YES: the logic output of this system is always the same as the input.

Consider a YES-gate of this topology that was designed to operate at 50 °C, that is, it exhibits two distinct concentrations of free S (output concentration) at input = 0 and input = 1 states. As the temperature decreases, the output concentration at input = 0 decreases due to stronger hybridization of Q and S at lower temperatures. If the I–Q interaction is less easy-to-melt than Q–S, then the output concentration in the input = 1 state also decreases. In this case, the overall temperature effect can be counteracted by introducing a single compensatory, easy-to-melt oligonucleotide C that binds to Q. This oligonucleotide increases the concentration of free S under both input conditions—but only at 25 °C. In the opposite case, if the I–Q interaction is more easy-to-melt than Q–S, a second compensator binding to I is required. The subsequent analysis is identical for both cases; therefore, for simplicity, we focus on the first one.

The performance of a logic gate is typically perceived as a separation between the output signals in the presence and absence of inputs; the larger this difference at both temperatures, the better the gate operates. However, since logic gates are not our ultimate objective but rather serve as an illustrative example of a signal-processing system, we formulate a more general goal, namely, to achieve preservation of absolute concentration levels at all possible input states. Achieving this, however, requires careful optimization.

We divide this optimization process into two stages. First, we determine the dissociation constants and initial concentrations necessary for the system to function as desired. At this stage, we ignore the specific oligonucleotide sequences and instead work with their numerical parameters (such as affinities); we also first consider only dimerization processes (multimerization will be considered below). Every dimerization network can be fully described by a matrix of dissociation constants (*K*_*d*_) and the initial component concentrations. Given these parameters, the system can be simulated by numerically solving a system of mass-action equilibrium equations to compute the free S concentration (i.e., output state). This leads to a purely numerical continuous optimization problem—in this case, involving the optimization of four initial concentrations (S, Q, C, and I) and three dissociation constants (for Q–S, Q–I, and Q–C interactions) to match the target free S levels at both temperatures. Other interactions are assumed to be absent (corresponding *K*_*d*_ = inf) at this stage and are therefore not optimized.

However, it is not possible to vary the dissociation constants at 25 and 50 °C independently, as they are coupled through the easy-to-melt level. To simplify the design, we discretize the *K*_*d*_ space (Fig. 1A) into 11 distinct easy-to-melt levels (see “Methods” for formal description). Thus, for a given level and a fixed *K*_*d*_ at 25 °C, the corresponding *K*_*d*_ at 50 °C is effectively determined. For all logic gates, easy-to-melt levels were chosen and fixed before the optimization. This approach reduces the number of independent parameters in the model and ensures that the simulated system behavior respects the underlying physical constraints of oligonucleotide thermodynamics. The optimization is performed using the Bayesian adaptive direct search (PyBADS)^37,38^, and the optimized *K*_*d*_ matrices are shown in Fig. 1E.

After identifying the optimal parameters, we proceed to the sequence design stage (Fig. 1F). We begin by randomly initializing the sequence of the first oligonucleotide, for example, S. Next, we design the sequence of Q, which is intended to interact with S. Q is initially set as the fully complementary sequence to S, and then mutated until the NUPACK-predicted dissociation constants *K*_*d*_ (SQ) at both temperatures closely match the previously optimized values. After that, we repeat the process for I, so that *K*_*d*_ (IQ) is close enough to its optimized value, and *K*_*d*_ (IS) is larger than some off-target threshold. The same procedure is also applied to the remaining oligonucleotides in the system (oligonucleotide C in this particular case), to ensure all interactions satisfy their respective design criteria. A detailed description of the optimization process is provided in the “Methods” section.

**Fig. 2:**
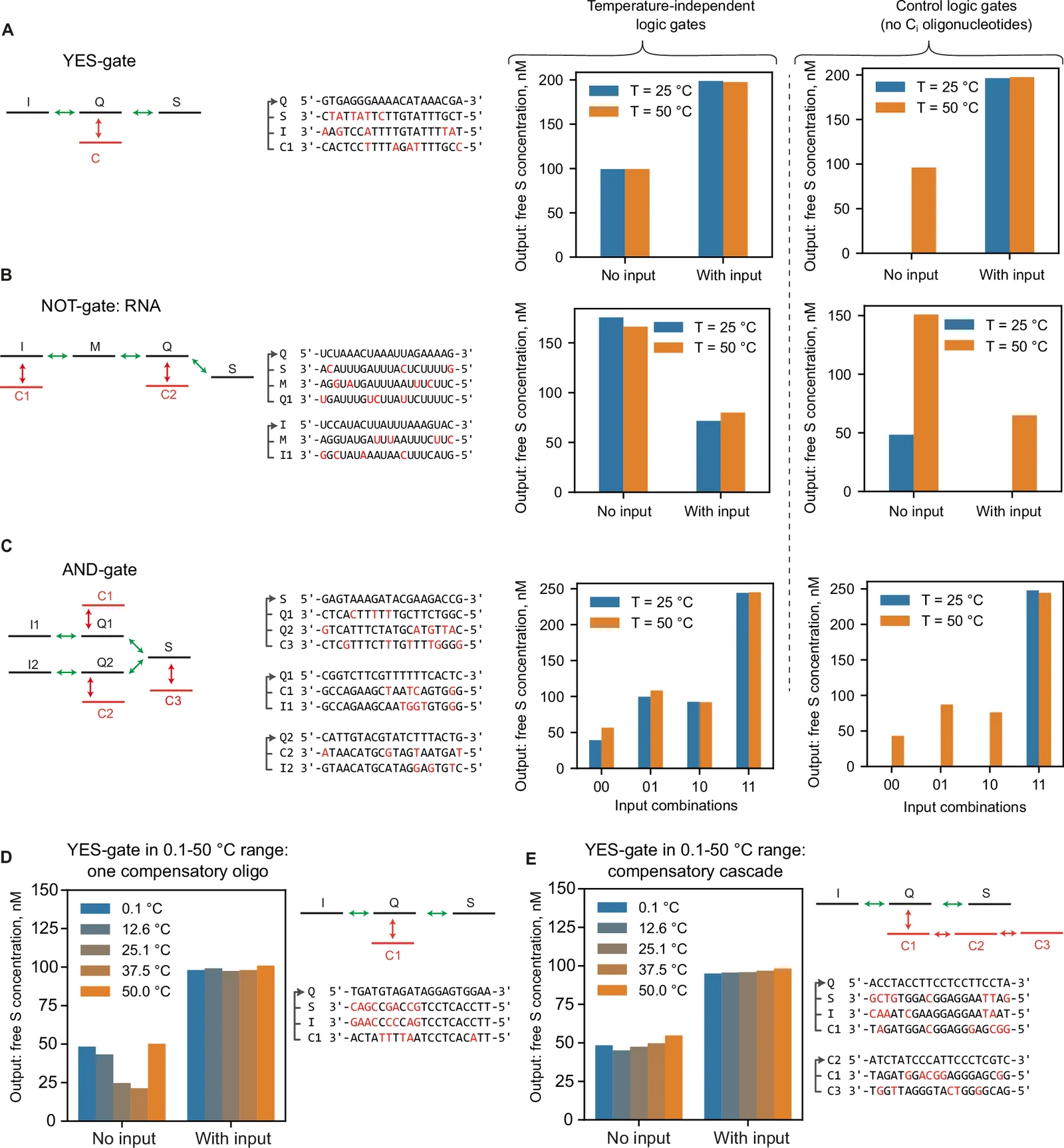
Design and predicted performance of temperature-independent logic gates. **A** DNA-based YES gate. **B** RNA-based NOT gate. **C** DNA-based AND gate. **D**, **E** DNA-based YES gate operating over the 0.1–50 °C temperature range. **D** shows the performance of the gate with a single compensator, and **E** with a compensatory cascade. The performance of the systems fine-tuned to account for the formation of multimeric complexes (i.e., with the NUPACK parameter max_compl_size = 3) is shown in Supplementary Figs. 4–7. For this and subsequent figures, concentrations and other parameters of all components are listed in Supplementary Methods. For (**A**–**C**), the left panels show the performance of the optimized compensated system, while the right panel shows the performance of the same system without compensatory oligonucleotides. Here and below, nucleotides complementary to those in the corresponding sequence (shown with arrows) are written in black, and non-complementary ones in red. Please note that some sequences are written from 3′ to 5′ for demonstrating mismatches. Source data are provided as a Source Data file.

Once all oligonucleotide sequences have been determined, a final simulation of the system is performed to verify that the resulting behavior matches the intended design. It is observed that in the optimized system, the output signals corresponding to both possible input values are nearly identical at both temperature conditions (Fig. 2A). Here and below, the concentrations, sequences, affinities, and other parameters of the systems presented in all figures are listed in Supplementary Methods. For all systems, each species has a concentration within the range of 1 nM to 10 μM.

Using the same design principle, we construct more complex logic gates. The NOT gate is the logical inverse of the YES gate: when the input is 0, the output should be 1, and vice versa. This functionality is implemented using a four-oligonucleotide scheme (Fig. 2B). When the input I is absent, Q binds to M, leaving S mostly unbound and therefore in the output = 1 state. When I is present, M binds to I, reducing M’s availability to bind Q; the remaining unbound Q then binds to S, resulting in S being predominantly in the bound state, which corresponds to output = 0. In this case, two easy-to-melt oligonucleotides are required for temperature compensation: one that binds to I and another that binds to Q. Using this interaction topology, we apply the same sequence design procedure to determine the sequences of all participating oligonucleotides, ensuring that the desired thermodynamic behavior is maintained across temperatures. To demonstrate that the compensation principle is also readily applicable to RNA–RNA interactions, RNA was used in this gate’s design instead of DNA.

The AND gate is a two-input logic function in which the output is 1 only when both inputs are present. Its topology resembles two parallel YES gates that share a common output oligonucleotide, S (Fig. 2C). Each of the input-responsive strands, Q_1_ and Q_2_, can independently bind to S with sufficient affinity such that the presence of either Q_1_ or Q_2_ alone results in S being mostly bound. To enforce temperature robustness, we introduce compensatory oligonucleotides for both Q_1_ and Q_2_, as in the YES-gate design. Additionally, we include a compensatory oligo binding S. Sequence optimization is then performed following the same procedure.

For all three gates, we also consider control systems—identical to the ones just described but lacking the compensatory oligos *C*_*i*_. In all three cases, the output concentration in the control systems changes significantly with temperature, in contrast to the optimized systems.

To simplify the design of the described systems, we considered only two input conditions: complete absence or presence at a single fixed concentration. Nevertheless, the final gates maintain the same input–response pattern at both temperatures across all intermediate input concentrations (Supplementary Fig. 2).

We should also note that, for example, for the compensated YES gate (Fig. 2A), the True-False signal difference is not greater than that in the non-stabilized case at their respective optimal temperatures. This reflects the classic robustness-over-peak-performance trade-off, a well-known concept in biotechnology (e.g., enzyme engineering^39,40^) where expanding the operational range and ensuring stability is typically more advantageous than maximizing performance within a narrow parameter window. On the other hand, this effect arises from the gate construction strategy: the control and compensated gates were designed to operate identically at 50 °C (as compensators are easy-to-melt). However, if we consider the gates that have identical outputs at 25 °C, then the True-False difference at 50 °C becomes significantly greater for the compensated gate. Moreover, if the objective function is this True-False separation (instead of the preservation of absolute concentrations), the gate design problem becomes substantially simpler. In this case, virtually any YES gate with a topology that has a compensatory component binding Q (at a concentration comparable to that of the other components) will outperform a YES gate lacking such a compensator, provided that both exhibit identical performance at 25 °C (i.e., the same level of free S). This behavior is illustrated for both YES and NOT gates in Supplementary Note 2 and Supplementary Fig. 3.

### 0.1–50 °C temperature range and stability at intermediate conditions

But can the mechanism provide compensation for even more drastic temperature changes? And is optimization at the endpoints of the temperature range sufficient to ensure stability under all intermediate conditions? To answer these questions, we constructed a YES gate designed to operate over a significantly broader temperature range—from 0.1 to 50 °C. While the previously described design strategy was still effective in generating sequences that performed well at the extremes of this range, we observed substantial shifts in the concentration of free S at intermediate temperatures (Fig. 2D).

When not only endpoint temperatures are considered, accurate evaluation of binding parameters at intermediate temperatures becomes essential. During the intermediate stage of optimization of dissociation constants, we assumed a linear dependence of the Gibbs free energy on temperature, despite the fact that NUPACK predicts deviations from this behavior in some cases (Supplementary Note 3). Nevertheless, at the final stage after the sequence design, simulations were performed in NUPACK (without such an assumption).

A more general and powerful strategy, compared to using compensatory oligonucleotides, is to employ compensatory cascades instead. We demonstrate this strategy using the same 0.1–50 °C YES-gate (Fig. 2E). In a compensatory cascade, each successive interaction (Q-C_1_, C_2_–C_1_ and C_3_–C_2_) is progressively more “easy-to-melt” than the previous one.

As the temperature increases, the least stable interaction (C_3_–C_2_) dissociates first, followed by C_2_–C_1_, and finally C_1_–Q. This hierarchical compensation allows the system to gradually adjust to temperature changes, providing dynamic correction across the entire range. Using this strategy, we achieved stable and uniform gate performance at all intermediate temperatures. It is worth noting the exceptionally large change in the *K*_*d*_ of the participating reactions—just over 9 orders of magnitude across the temperature range from 0.1 to 50 °C. The described strategy of compensatory cascades is broadly applicable and can be extended to a wide range of systems, including considerably more complex ones (such as those discussed below), to enable fine-tuning of performance at intermediate temperatures.

Another simplification used for the design of the described gates was the consideration of only complexes consisting of at most two strands. At the same time, real biomolecules (especially nucleic acids) are typically capable of forming higher-order complexes. However, accounting for such complexes does not introduce any fundamental changes and does not compromise the compensatory capabilities of the mechanism: Supplementary Figs. 4–7 demonstrate equally robust performance of the YES, NOT, AND, and 0.1–50 °C YES gates, respectively, when triple complexes are taken into account (NUPACK max_compl_size = 3), designed through simple fine-tuning of previously optimized sequences and concentrations.

### Experimental realization of temperature-independent gates

Now, after we have described the general theory for designing temperature-independent logic gates, we want to demonstrate the real-life feasibility of implementing this idea in a wet-lab experiment using the YES gate as an example.

An alternative to the compensation mechanism described above is simply to increase the affinities of all interactions so that on/off behavior is preserved even at high temperatures. However, this approach has two major drawbacks. First, it generalizes poorly to behaviors beyond simple on/off responses, making it difficult to achieve intermediate states. This thereby eliminates the advantages of the molecular commutation regime over the strand displacement technique (for example, elementary algebra or ultrahigh input gates have been shown to date only with molecular commutation, and it is unclear whether a reasonable strand displacement solution can be engineered for such tasks).

Second, it introduces substantial kinetic limitations. In general, higher affinities lead to a significant decrease in the dissociation rates of complexes, which slows down the overall performance of the system. In the extreme case of long, fully complementary strands, the interaction becomes effectively irreversible^41^, and complexes remain stable almost indefinitely under room temperature conditions, making signal propagation mediated by such interactions prohibitively slow in practice.

To identify an upper limit for oligonucleotide affinity that still allows equilibration within several hours, we performed a wet-lab screening experiment. We used a classical YES-gate configuration in which the output strand S was labeled with the fluorophore Cyanine 3, and the quencher strand Q was labeled with BHQ2. In this setup, the concentration of free S is directly proportional to fluorescence intensity. We tested several Q sequences with varying affinities to S, corresponding to dissociation constants ranging from 3.2 nM to 8.6 fM (as predicted by NUPACK). For each Q, we used an input strand I that was identical to S except for having one fewer mismatch with Q. In this experiment, all oligonucleotides had a concentration of 100 nM.

**Fig. 3:**
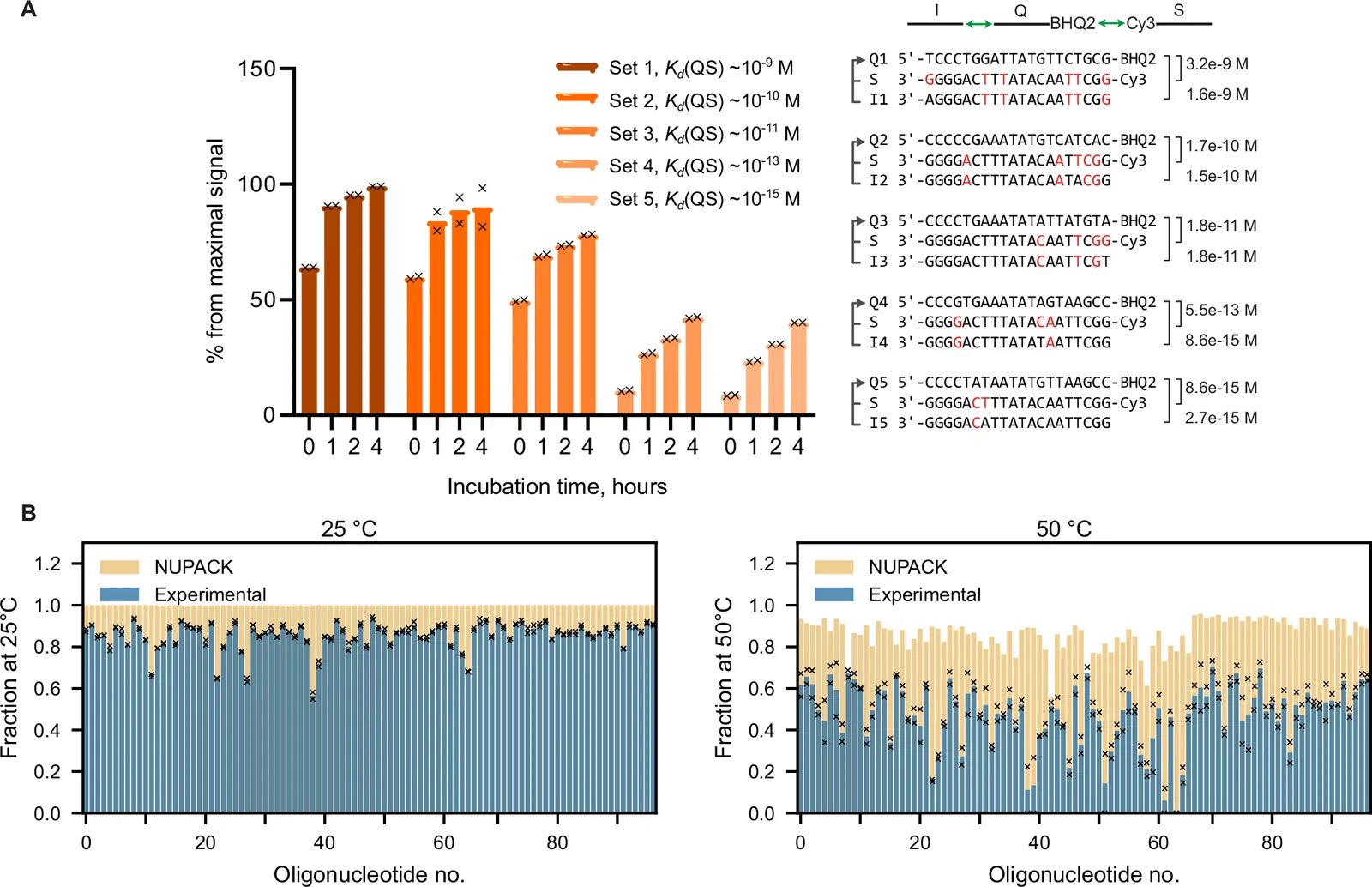
Experimental screening approach for the design of a temperature-independent logic gate. **A** Displacement kinetics of strand Q by input strand I from the QS complex at different affinity levels. The concentration of each oligonucleotide is 100 nM. Data are presented as mean values and individual replicates (*n* = 2 independent samples), expressed as a percentage of the ratio of free S concentration at the indicated time to that after overnight incubation. **B** Accuracy of NUPACK affinity predictions at 25 and 50 °C. Data are presented as mean values and individual replicates (*n* = 2 independent samples), expressed as the concentration of the resulting duplex if both strands are mixed at 1 µM. Each bar represents a distinct oligonucleotide pair. Please note that some sequences are written from 3′ to 5′ for demonstrating mismatches. Source data are provided as a Source Data file.

We initially mixed Q with S and incubated the mixture for 10 min to allow complex formation. Subsequently, we added the input strand I and monitored fluorescence over a 4-h period. Then, a 25–60–25 °C heating-cooling step was performed, and the samples were incubated for 16 h (overnight) to achieve equilibrium. The resulting fluorescence levels, normalized as a percentage of those measured in equilibrium, are shown in Fig. 3A.

The data reveal a clear trend: higher-affinity duplexes equilibrate more slowly. Duplexes with dissociation constants around 1 nM approach equilibrium within approximately 1 h, while those with *K*_*d*_ values near or below 100 fM fail to reach even 50% of their maximum signal after 4 h. Based on these findings, we restrict all sequence optimization procedures to oligonucleotide pairs with *K*_*d*_ > 10 pM (including those described previously) to balance high binding affinity with acceptable equilibration speed. This does not preclude molecular commutation at lower *K*_*d*_; however, in such cases, we believe, a non-equilibrium regime (e.g., when the values of the variable are determined before equilibrium is reached) or a fully kinetic regime (where values of the variables are the rates of the reactions) may be more effective.

To successfully implement the logic gates in a wet-lab experimental setting, the oligonucleotides used must exhibit dissociation constants that closely match those obtained through prior optimization. However, this condition is not always met in practice, as previous studies have shown^24^ that affinity predictions by tools such as NUPACK may not be fully accurate, particularly for oligonucleotide duplexes with low complementarity. Moreover, since our experimental setup uses fluorescent labels, they may also contribute to the interaction energy^42^, which is not accounted for by NUPACK. To verify this and manually identify suitable oligonucleotide sequences for a temperature-independent YES gate, we conducted a screening experiment in which we tested 96 different input strands (I) against a fixed pair of strands, S and Q.

We measured the fluorescence output of each gate to retrieve the affinity of the I–Q interaction. The affinity was plotted in custom units consistent with prior work^24^, namely as the equilibrium concentration of the IQ duplex as if both strands were mixed at a 1 μM concentration. This value ranges from 0 to 1, with values closer to 1 indicating stronger binding. Measurements were performed at both 25 and 50 °C. In all cases, the experimentally measured apparent affinities were substantially lower than those predicted by NUPACK (Fig. 3B).

Based on the screening results, we selected suitable candidates for constructing a temperature-independent YES gate. Oligonucleotides that exhibited the strongest binding to Q at both 25 and 50 °C were chosen to serve as input strands (I). Candidates for the compensatory strand C were selected from sequences that showed relatively strong binding at 25 °C but significantly weaker binding at 50 °C, consistent with the desired easy-to-melt behavior.

With experimentally validated affinities in hand, we re-ran the Bayesian optimization procedure to determine the optimal concentrations of all components under the new constraints. These optimized concentrations were then used in the final experimental implementation of the gate.

**Fig. 4:**
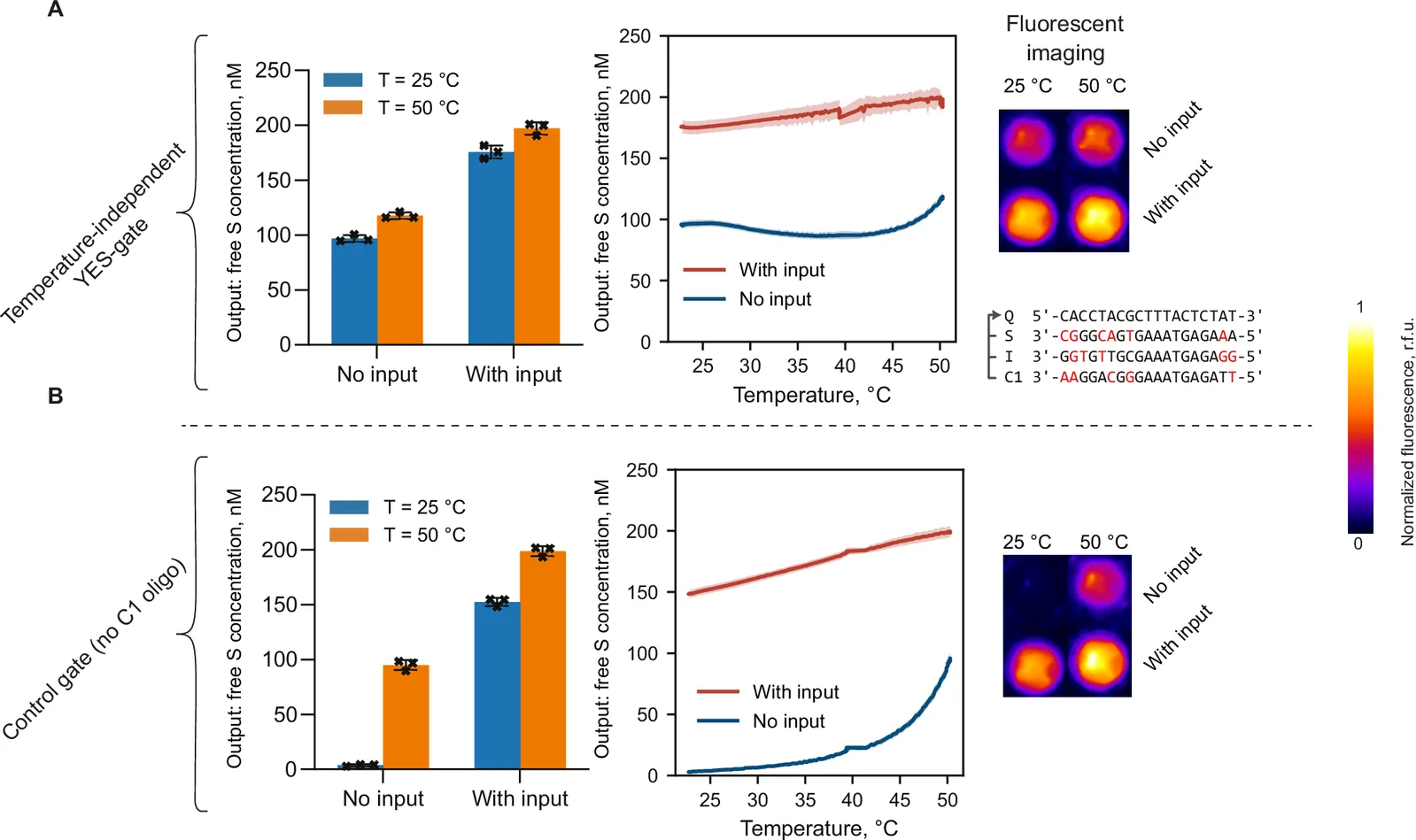
Experimental implementation of the temperature-independent YES gate. Performance of the compensated (**A**) and control (**B**) YES gates. Left panel shows endpoint performance at 25 and 50 °C, and the right panel shows melting curves and fluorescent images of the reaction wells. Bar graphs show mean ± s.d.; black cross markers indicate individual replicates; melting curves show mean values (solid line) with 95% confidence intervals (shaded area), *n* = 3 independent samples for both cases. Due to the temperature dependence of Cyanine3 quantum yield, absolute fluorescence values differ between 25 and 50 °C. For clarity, images were linearly adjusted so that control wells containing Cyanine3 exhibit the same mean fluorescence. A pre-heating-cooling cycle (25–50–25 °C) was used in the experiment to ensure equilibrium at the starting point of the melting curve. Without the cycle, the gate reaches equilibrium within 30 min at 25 °C; the kinetics are shown in Supplementary Fig. 8. Please note that some sequences are written from 3′ to 5′ for demonstrating mismatches. Source data are provided as a Source Data file.

Using the selected oligonucleotide candidates, we measured the performance of the temperature-compensated YES gate across the target temperature range of 25–50 °C and compared it to a non-compensated version of the gate. Melting curves for both systems are presented in Fig. 4, along with fluorescence images of the reaction wells at 25 and 50 °C. To demonstrate that this is not a single unique molecular set that exhibits such behavior, a similar experiment was performed using another oligonucleotide set from a different manufacturer (see Supplementary Fig. 9).

In the non-compensated gate, the melting curve corresponding to the input = 0 state shows a substantial increase in fluorescence, reflecting a 25-fold change in the concentration of free S over the temperature range. In contrast, the temperature-compensated gate maintains a stable output, with the variation in free S concentration remaining below 20% across temperatures, regardless of input state. Therefore, the performed wet-lab experiments suggest that the topological compensation approach indeed can achieve a high degree of temperature independence in biomolecular signal processing systems.

### Ionic strength independence

The vast majority of biomolecular interactions depend on ionic strength, often in a complex, non-monotonic manner. However, in the case of nucleic acids, the effect of ionic strength on equilibrium parameters closely resembles that of temperature: both factors lead to a monotonic change in dissociation constants. Thus, topological compensation in nucleic acid systems provides an additional valuable modality—stabilization of signaling systems against variations in ionic strength.

**Fig. 5:**
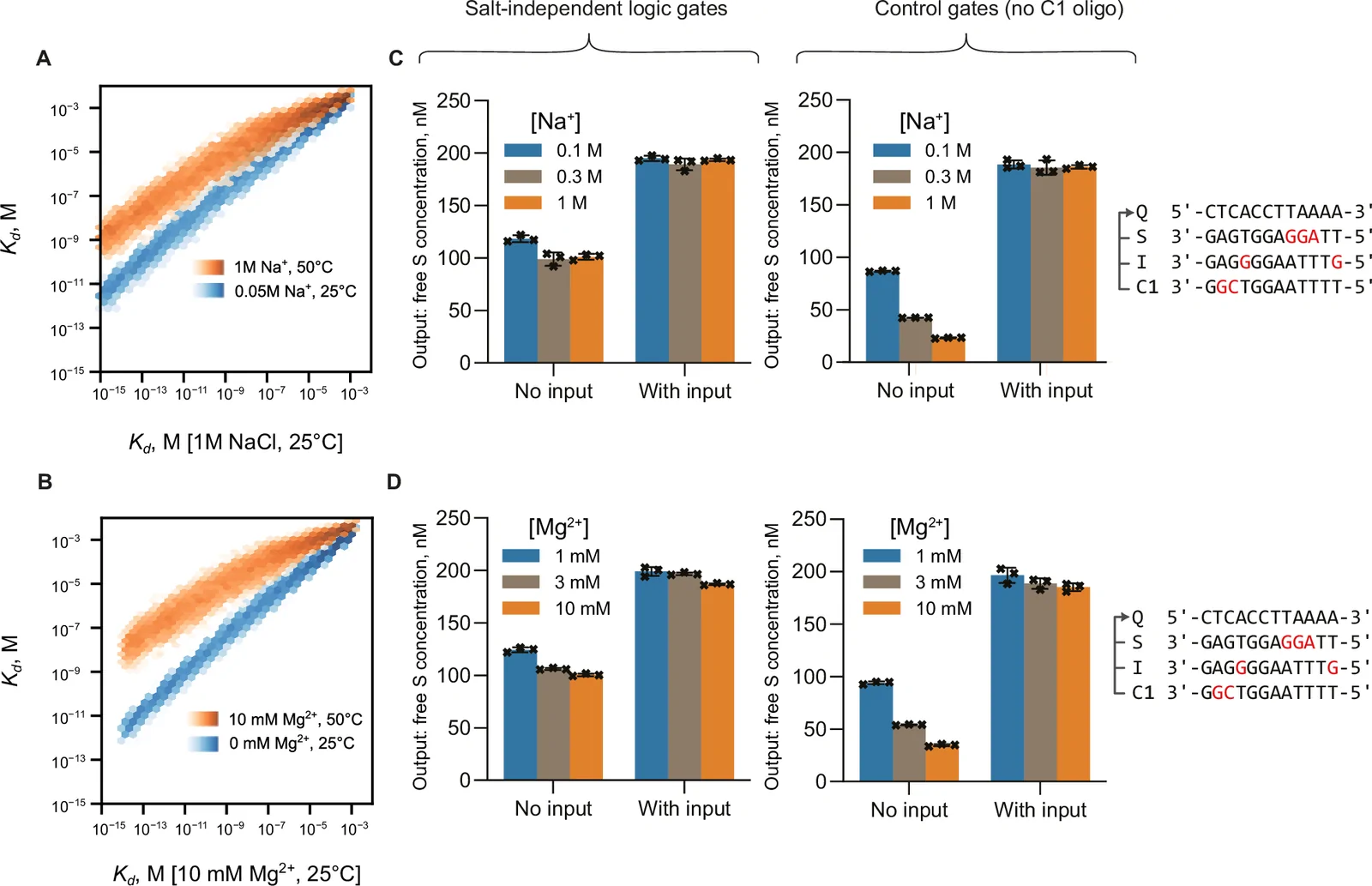
Experimental realization of salt-independent gates. **A**, **B** NUPACK-predicted distribution of dissociation constants under high and low concentrations of Na⁺ and Mg²⁺, compared to that under high and low temperatures for the same oligonucleotides. **C** Experimental performance of salt-independent YES gates across Na⁺ concentrations ranging from 0.1 to 1 M, the magnesium concentration is zero. The left panel shows the performance of the optimized gate with compensatory oligonucleotide, while the right panel shows the same gate without compensation. **D** Experimental performance of salt-independent YES gates across Mg^2+^ concentrations ranging from 1 to 10 mM, the sodium concentration is zero. The left panels show the performance of the optimized gates with a compensatory oligonucleotide, while the right panels show the same gate without compensation. For (**C**, **D**) data are shown as mean ± s.d., black cross markers indicate individual replicates, *n* = 3 independent samples. In both cases, the system reaches equilibrium in less than 5 min; the kinetics are shown in Supplementary Fig. 12. Please note that some sequences are written from 3′ to 5′ for demonstrating mismatches. Source data are provided as a Source Data file.

To illustrate this, we plotted the distributions of dissociation constants (*K*_*d*_) under low and high salt concentrations for two biologically relevant ions, Na⁺ and Mg²⁺, and compared them to *K*_*d*_ distributions at 25 and 50 °C (Fig. 5A, B). For both salts, the average shift in *K*_*d*_ resulting from a decrease in ionic strength is smaller than that caused by an increase in temperature. However, the characteristic width of the *K*_*d*_ distribution is also narrower under ionic strength variation. This implies that although the magnitude of compensation required is lower in the case of salt concentration changes, the diversity of available easy-to-melt and hard-to-melt oligonucleotides is also more limited.

A salt-independent YES gate can be readily designed based on existing sequences developed for the temperature-independent system (Fig. 2A). We applied an evolutionary algorithm to refine both the sequences and their concentrations to achieve robust performance in simulation under varying ionic conditions (Supplementary Fig. 10).

To experimentally implement a salt-independent YES gate, we employed the same strategy as for the temperature-independent system, and the experimental performance of the salt-independent YES gate for both variations in Mg^2+^ and Na^+^ concentrations is shown in Fig. 5C, D. To implement these gates, we performed a screening procedure analogous to that shown in Fig. 3B; the corresponding results are presented in Supplementary Fig. 11. Interestingly, in this screening, a better correlation is observed between NUPACK predictions and experimentally measured affinities compared with that in Fig. 3B. Based on these experimentally measured affinities, candidate input and compensatory oligonucleotides were selected, and the concentrations of all participants were optimized using Bayesian optimization. For variations in sodium and magnesium concentrations, identical sequences were used, with only the concentrations adjusted.

### Receptor activation in complex networks

Up to this point, we have focused on relatively small-scale biochemical systems designed to respond to changes in the concentration of one or more molecular participants. However, can such compensation be performed simultaneously across a large network? In this section, we address this question by simulating such a model receptor–activator system with 100 receptors and 100 activators.

**Fig. 6:**
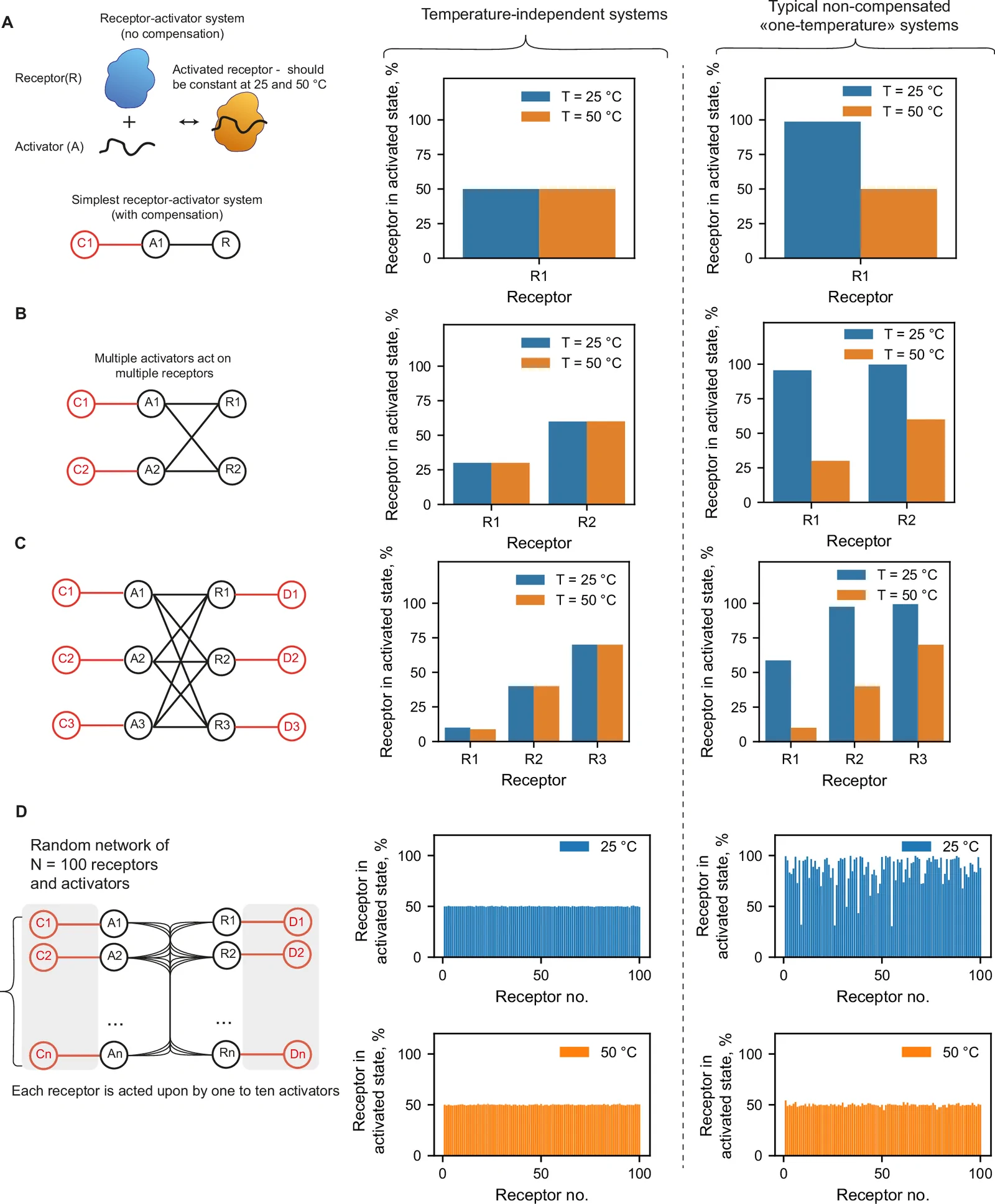
Temperature independence of receptor–activator networks. **A**–**C** Network topology and predicted percentage of activated receptors for systems with 1, 2, and 3 receptor–activator pairs. Each receptor is assigned a predefined target activation level. **D** Topology and predicted performance of a large-scale network with 100 receptors and 100 activators. Each receptor is regulated by between 1 and 10 activators. Simulation of the same system optimized for performance at intermediate temperatures is shown in Supplementary Fig. 14. For (**A**–**D**), the left panels show optimized systems with compensatory components; right panels show systems without compensation, optimized separately to have predefined activation levels at 50 °C only. Source data are provided as a Source Data file.

In such a setup, we consider that binding of the activator to its corresponding receptor activates the receptor, while dissociation of the complex returns the receptor to its inactive state (Fig. 6A). There are many possible implementations of such systems in nature; for example, activation may occur due to conformational changes in a receptor upon its binding to a ligand^43^. Here, we treat the receptor activation level as the output of the system, by analogy with logic gates.

Suppose that the level of receptor activation must remain constant across varying external conditions—for example, across a range of temperatures. As in previous sections, we focus on two representative temperature points: 25 and 50 °C.

Before considering the full 100-receptor network, we first examine the simple case of a system with a single receptor (R1) and a single activator (A1). In this case, temperature-induced changes can be effectively compensated using just one additional component, C1. As temperature increases, both the A1–R1 and C1–A1 interactions weaken. On the one hand, the reduced affinity between A1 and R1 leads to an increase in the concentration of free (and thus non-active) R1, resulting in a lower level of receptor activation. On the other hand, the weakened interaction with C1 increases the concentration of free A1, which in turn promotes more binding to R1 and counteracts the temperature effect. If the strengths of these interactions are properly optimized, the opposing effects can effectively cancel each other out, maintaining a stable level of receptor activation across temperatures. In our specific case, the target activation level of R1 is set arbitrarily to 50% (Fig. 6A).

We can now extend this system to a more complex case involving two receptors (R1 and R2) and two activators (A1 and A2), where each receptor can be activated by either activator. Let us fix arbitrary target activation levels—for example, 30% activation for R1 and 60% for R2. These activation states can be maintained across temperatures (25 and 50 °C) using a similar compensation strategy. Specifically, we introduce two compensatory components, C1 and C2, which bind to A1 and A2, respectively. By tuning the interaction strengths and concentrations of all components, the system can achieve temperature-independent activation levels for both receptors (Fig. 6B).

In the more complex case involving three receptors and three activators, compensation requires an additional design consideration. In this scenario, we introduce compensatory participants that bind to the receptors without inducing activation, thereby competing with the activators for receptor binding sites. These non-activating competitors effectively modulate the concentration of free receptors available for activation, helping to balance the system’s response across temperatures. Using this strategy, we successfully maintained arbitrary, predefined activation levels for all three receptors (10, 40, and 70% for R1, R2, and R3) at both 25 and 50 °C (Fig. 6C).

The approach described for *N* = 3 can be readily scaled to much larger networks: Fig. 6D illustrates the performance of a system comprising 100 activators and 100 receptors. In this network,  each receptor is regulated by 1-10 activators (the distribution of the number of activators acting on a single receptor is shown in Supplementary Fig. 13, with an average of 5).

As in previous cases, each activator and receptor is influenced by a single compensatory component. For simplicity, we assume that the activation level of each receptor must be precisely 50% at both temperatures.

To determine the optimal interaction parameters, approximately 700 affinities and 400 concentrations must be adjusted. The dimensionality of this problem significantly exceeds the capacity of the optimization algorithms employed. Therefore, we adopted the following strategy. We identified the three receptors with the largest deviations from the target activation levels and performed optimization of the interaction affinities with all associated activators, their individual compensators, as well as the compensators of the corresponding activators. Then we optimized the concentrations of these components. Next, again, the receptors with the greatest deviations were re-identified, and the process was repeated until the desired accuracy was achieved.

Using this algorithm, we were able to attain a high degree of consistency in receptor activation levels across all 100 receptors within a few hundred optimization cycles, and the mean deviation from the 50% level was less than 0.2% (Fig. 6D). This demonstrates that the compensation approach remains effective even in systems with significantly increased interaction complexity.

For all four receptor–activator network cases, we also examine typical control systems without the compensatory components and optimized for operation at a single temperature (50 °C). In all cases, the control systems show substantial differences in activation levels at 25 and 50 °C (Fig. 6, right panels).

The data in Fig. 6 show an ideal level of compensation that may even be perceived as unrealistic for biological systems. In practice, biological systems evolve to optimize multiple characteristics simultaneously, often trading off some properties against others. At the same time, we consider it important that, during certain evolutionary stages, the availability of such a tool of ideal compensation may have played a significant role in adaptation to specific environmental conditions and in enabling the emergence of other functional traits.

So far, we have shown compensation in systems in which only dimerization or higher-order processes were present. However, in real molecular systems, temperature dependence may also arise from monomolecular processes, such as protein misfolding. To demonstrate the performance of topological compensation in this case, we introduce a reversible temperature-dependent misfolding process (leading to the transition of participants into a fully inactive state) into a similar large receptor-activator network. Such reactions lead, in addition to the previously considered dimerization temperature dependence, to a temperature-dependent reduction in the effective total concentrations. Supplementary Note 4 and Supplementary Fig. 15 show that such systems can also be efficiently stabilized against temperature changes.

### Algebraic systems

In the previous sections, we have considered rationally designed systems, in which each reaction in the network serves a clearly defined and interpretable function. Here, we aim to demonstrate that compensation is also possible in systems where it is impossible to assign a specific function to each individual reaction, but each reaction makes an essential contribution to the behavior of the network as a whole. In our view, such networks more closely resemble complex, interconnected natural signaling cascades.

Consider an input component I, an output participant S, and an arbitrary black-box set of intermediate components M₁, M₂,…, M_n_. We define a normalized and centered input variable *X* as:

1 $$X=\frac{{C}_{I}-{C}_{0}}{2{C}_{0}},$$

where *C*_*I*_ is the total concentration of input *I*, and *C*_0_ is an arbitrarily chosen reference concentration (set to 5 µM). Similarly, the output variable *Y* is defined as the centered equilibrium concentration of free *S*:

2 $$Y=\left[S\right]-{S}_{0},$$

where *S*_*0*_ is a reference concentration, set to 20 nM. Our previous work^24^ has shown that by carefully selecting the sequences and concentrations of *I*, *S*, and all intermediate participants *M*_*i*_, it is possible to engineer complex nonlinear relationships between *Y* and *X*. For example, specific parameter sets have been identified that allow the system to approximate mathematical functions such as *Y* = *X*^*3*^ or *Y* = sin(*X*).

Here, we will demonstrate that suitable parameter sets (affinities and concentrations of components) can be determined to implement even such complex signal-processing systems in a temperature-independent manner. In the case of nucleic acids, we expect similar stabilization capabilities against variations in ionic strength as well.

To achieve this, we first optimize the system to function at 50 °C. Specifically, we minimize the mean squared error between the system’s output and a predefined target function by adjusting the dissociation constants and initial concentrations of *N* = 10 oligonucleotides using Bayesian optimization.

After this, we introduce one compensator per existing network participant (except for the output component), such that each participant interacts with exactly one distinct compensator. We then optimize the compensators’ parameters (concentrations and the dissociation constants of their interactions) to minimize the objective function at 25 °C. Finally, we perform an optimization of all interactions and concentrations across both temperatures. In all optimization stages, dissociation constants and concentrations are adjusted in an alternating manner: initially, concentrations are held constant while *K*_*d*_ values are optimized; then *K*_*d*_ values are fixed and concentrations are optimized. This iterative process continues until convergence.

After each step, we apply a pruning criterion: if a concentration falls below a defined threshold (10 nM) or a *K*_*d*_ value becomes too weak ( > 10^−4^ M), the corresponding variable is excluded from further optimization. In cases where a concentration drops below the threshold, not only is the concentration removed from the optimization process, but all of its associated interactions are frozen as well, effectively eliminating it from the system.

**Fig. 7:**
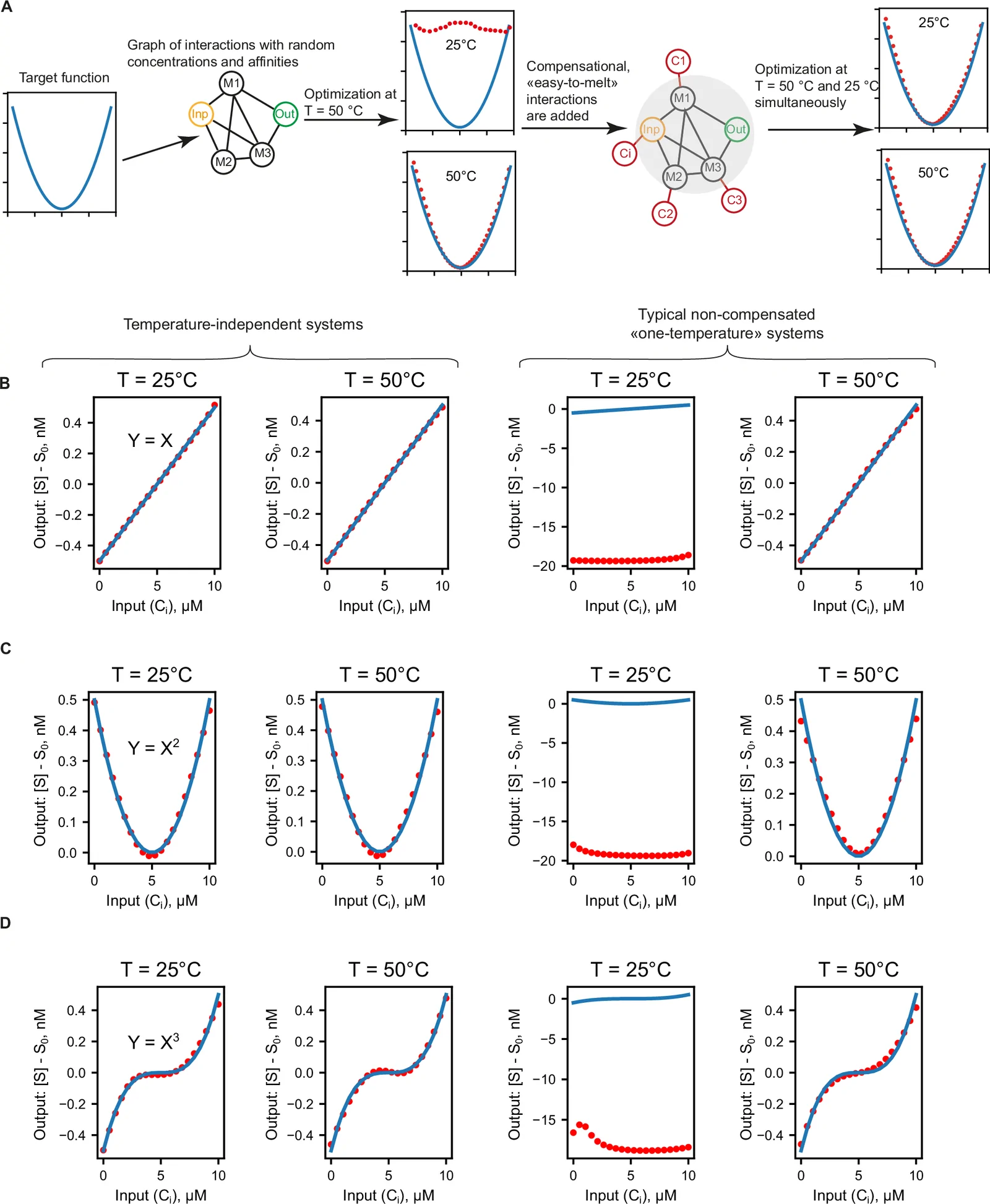
Processing of complex analog signals by biomolecular networks in a temperature-independent manner. **A** Schematic of the optimization algorithm. **B**–**D** Predicted performance of systems implementing linear, quadratic, and cubic functions at 25 and 50 °C. Left panels show optimized systems with compensatory components; right panels show systems without compensation, optimized separately to follow the target function at 50 °C only. Solid lines represent the objective profile, and dots represent the simulated system performance. Source data are provided as a Source Data file.

Using this strategy, we successfully identified system parameters that enable robust analog signaling in the form of linear, quadratic and cubic functions at both 25 and 50 °C (Fig. 7B–D). For comparison, we also show the typical behavior of systems optimized only at high temperature. While they perform ideally at 50 °C, they effectively cease to properly process the signal at low temperature.

It is important to note that, in this section, our objective did not include maximization of the amplitude of the output concentration (*Y*) changes. Our goal was to demonstrate the general principle of compensation in complex networks. The chosen amplitude values (for *X* and *Y*) were therefore selected primarily to simplify the optimization problem. At the same time, achieving larger changes in *Y* in response to smaller changes in *X* is certainly possible, but will require longer optimization or the use of more advanced optimization approaches^31^. Nevertheless, even relatively small variations in *Y* can be biologically significant, as downstream systems may strongly amplify such signals—for instance, when the output corresponds to mRNA concentration, from which many copies of a target protein can be synthesized.

Thus, we have shown that topological compensation can render even complex nonlinear biomolecular signal processing systems temperature-independent. Unlike logic gates, which were designed using a rational approach, these systems were obtained through stochastic methods. While rational approaches are easier for human understanding and experimental implementation, probabilistic, evolution-like algorithms more closely resemble the mechanisms by which such systems might have emerged in living organisms.

### Programmable temperature/ionic strength dependence

After thoroughly discussing temperature independence (i.e., a dependence reduced to a constant), we proceed to the more general task of designing systems with arbitrary dependence on temperature or ionic strength. Such behavior could be beneficial, for example, for inducing signals of arbitrary forms in different cell types as body temperature rises during disease.

Another illustrative example of where such systems may be important is in ensuring temperature independence of a higher-level molecular system with its own complex temperature dependence. For example, consider temperature-independent bacterial chemotaxis. In this well-studied system^22^, the key determinants of this independence include (1) the enzymatic network and (2) the mRNAs encoding these enzymes together with the translation-related machinery. The latter ensures a selective and tightly controlled increase in the expression of specific components of the enzymatic network as temperature rises.

**Fig. 8:**
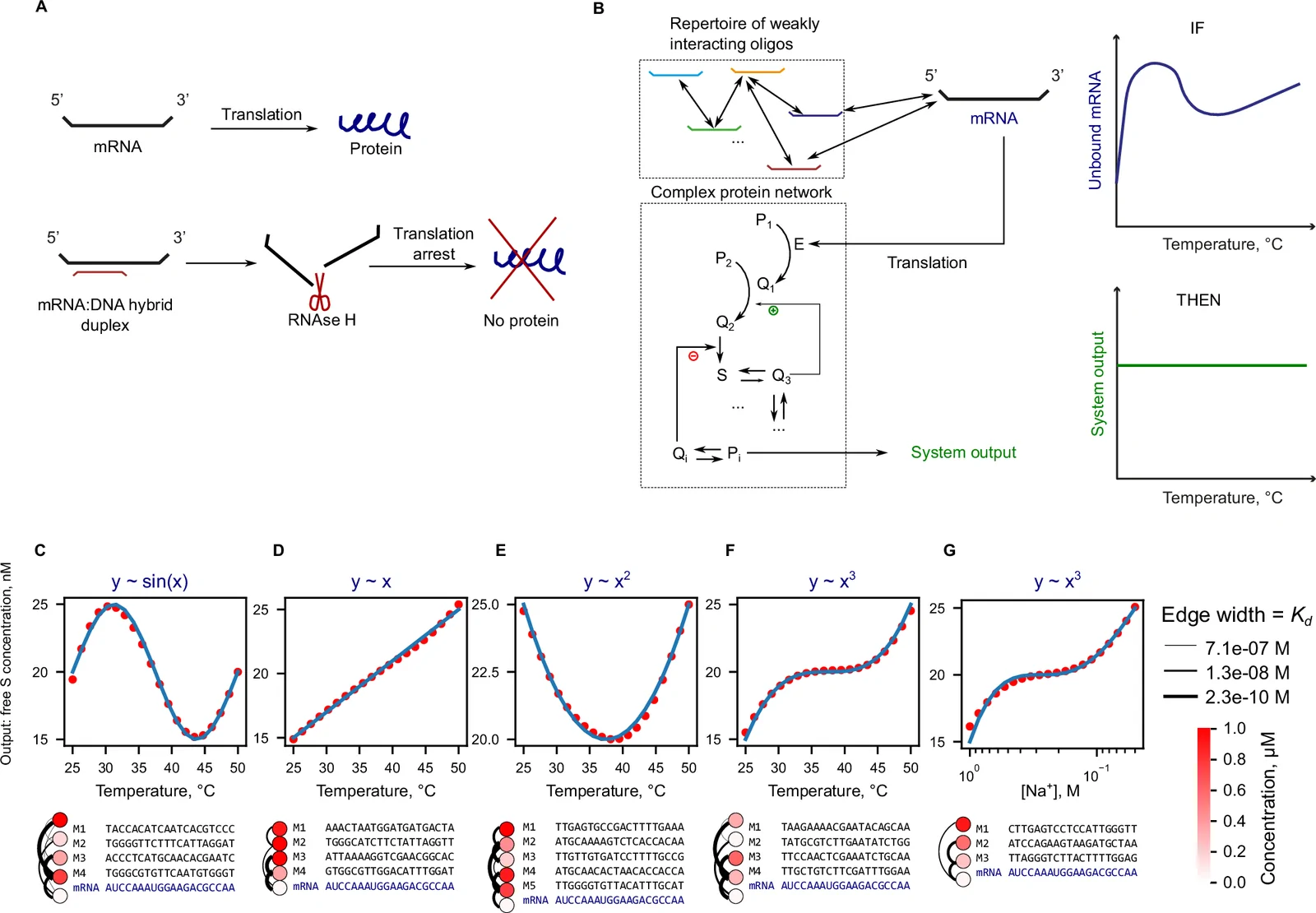
Molecular commutation systems with programmable temperature/ionic strength dependence. **A** Schematic of translation control via an RNase H-mediated mechanism. **B** Scheme of a nucleic acid molecular commutation system that compensates for the complex behavior of a protein network. Temperature compensation of the protein network requires a predefined level of protein expression, which is regulated by mRNA levels. **C**–**F** Performance and interaction graphs of molecular commutation systems exhibiting sinusoidal, linear, quadratic, and cubic temperature dependence. **G** Performance and interaction graph of a molecular commutation system exhibiting cubic dependence on ionic strength. The *X*-axis direction is reversed in (**G**). Solid lines represent the objective profile, and dots represent the simulated system performance. Node colors represent oligonucleotide concentrations, and edge widths represent the corresponding dissociation constants. DNA-RNA interaction was simulated according to the DNA-DNA NUPACK model. The simulation was performed taking into account complexes consisting of up to three strands (NUPACK max_compl_size = 3). Source data are provided as a Source Data file.

In turn, as demonstrated in our prior work^24^, there exist mechanisms by which molecular commutation can significantly influence protein translation levels. Specifically, binding of DNA oligonucleotides to a specific site on an mRNA molecule may lead to its cleavage mediated by RNase H, thereby reducing the expression of the corresponding protein (Fig. 8A). Consequently, free mRNA levels (i.e., mRNA not bound to oligonucleotides), representing the output of the nucleic acid affinity-based network, may directly determine protein concentrations.

Thus, suppose that a certain complex biochemical system requires, for its temperature independence or other higher-level signaling, a specific temperature-dependent profile of the level of free mRNA (Fig. 8B). In this section, we show that it is possible to identify sets of DNA oligonucleotides that realize predefined complex, non-monotonic dependencies on temperature or ionic strength.

The formal statement of the problem is analogous to that of the previous section: the variable *Y* (the level of free mRNA) is defined in exactly the same way, whereas the variable *X* is defined as the normalized and centered temperature:

3 $$X=\frac{T-{T}_{min }}{{{T}_{max }-T}_{min }}.$$

The identification of DNA sets having a predefined temperature dependence *Y(X)* was carried out in a manner similar to the design of logic gates. We first fixed the sequence of the “output oligonucleotide” as the AUG-containing region of the firefly luciferase mRNA that was used in our prior study^24^ for the experimental demonstration of the molecular commutation effect on protein expression.

For each specified function *Y(X)*, the required dissociation constants, easy-to-melt levels, and concentrations of the components were first determined using Bayesian optimization. After that, DNA sequences realizing these parameters were identified using an evolutionary algorithm. Finally, an evolutionary fine-tuning of both sequences and concentrations was performed to ensure that the system exhibited the desired behavior while accounting for nonzero concentrations of triple complexes (NUPACK max_compl_size = 3).

This procedure was carried out for four sample dependencies *Y(X)*: linear, quadratic, cubic, and sinusoidal. The resulting DNA sets, their interaction graphs, and their temperature dependencies are presented in Fig. 8C–F.

In addition, Fig. 8G shows that nucleic acid networks can exhibit similarly complex, predefined dependencies on ionic strength. Since the free energy of hybridization depends logarithmically^44^ on sodium concentration (in contrast to its linear dependence on temperature), for consistency, we chose the logarithm of the normalized sodium concentration as the independent variable (X):

4 $${X}_{{ionic}}={\log }_{10}\left(a\left[{{Na}}^{+}\right]+b\right),$$

where the constants *a* and *b* were chosen such that the sodium concentration range from 50 mM to 1 M is mapped onto the interval [0, 1] for *X*. Using a DNA set previously optimized to produce a cubic dependence on temperature (“Methods” section for details), we applied an evolutionary algorithm to identify a new set that realizes an analogous dependence on ionic strength.

To summarize, this remarkable feature of the molecular commutation networks allows them to receive a single cue from the environment (e.g., temperature or ionic strength) and react in many different ways. This phenomenon seems to be a perfect match for a hypothesis that the elevated body temperature during disease^45^ can act as a universal signal to each cell of the organism to initiate its own unique response profile. Triggering a multitude of distinct programmable responses via a single physical input (temperature) may be highly valuable since it does not dependent on mass transport of molecular messengers throughout the body. Perhaps, such considerations may be also relevant for embryogenesis^46^, in which changes in environmental temperature elicit differential responses across embryonic cells and give rise to temperature-induced phenotypes^47^.

## Discussion

The described phenomenon of topological compensation in affinity-based molecular networks provides a number of interesting ideas and possibilities for both evolutionary theory and molecular regulatory systems in general. Here, we discuss these aspects, compare this phenomenon to the known compensation mechanisms, as well as indicate current technical limitations.

### Applicability to non-equilibrium systems

An important aspect of the applicability of our mechanism to real biological systems is that many key biological processes operate under non-equilibrium conditions, whereas our work has focused exclusively on equilibrium systems. On the one hand, we do not see any fundamental limitations that would render such compensation infeasible in the kinetic regime, as one can expect the same or even greater diversity of temperature dependencies for kinetic rate constants. However, at present, there are no predictive tools for reaction rates comparable in maturity to NUPACK, which significantly complicates such investigations.

On the other hand, one of the key features of the low-affinity interaction regime is its intrinsic tendency toward fast kinetics^24^. Moreover, biological environments can further accelerate processes such as nucleic acid hybridization^41^ through, for example, positively charged biopolymers, which reduce electrostatic repulsion between strands, or molecular crowding effects, which effectively increase local concentrations. Therefore, even if an entire complex system operates under non-equilibrium conditions, some of its subsystems may function on much faster timescales than the rest and can thus be treated as being effectively at equilibrium with slowly varying external parameters.

### Topological compensation in protein-protein networks

In our study, we demonstrate the realization of a compensatory mechanism using nucleic acids as an example. We believe that the results demonstrated here may be extrapolated to other affinity-based molecular networks, including, of course, small molecule and protein networks^48,49^.

With regard to the fundamental feasibility of compensation in such networks, the only property of oligonucleotide networks that we explicitly relied upon is that the behavior of the dimerization reaction at low temperatures does not fully determine its behavior at higher temperatures. Evidently, even with the increased complexity of processes encountered in protein systems, this key assumption remains valid (see Supplementary Note 1). Furthermore, we do not see any conceptual limitations that would prevent this idea from being implemented in mixed networks of nucleic acids, proteins, small molecules, etc.

Moreover, protein networks possess the potential for even more complex behavior. In contrast to oligonucleotides, proteins undergo temperature-dependent folding and denaturation processes, exhibit intricate cooperative effects, and their free energy typically demonstrates a strongly nonlinear dependence on temperature. We believe that the presence of more complex processes does not complicate the problem of compensation; rather, it provides nature with a broader repertoire of mechanisms for its implementation.

It is also interesting to consider a special case of protein interactions, namely, enzymatic networks, which are regarded as the primary source of temperature-independence in some naturally occurring systems (such as circadian rhythms^21^). We, however, believe that affinity-based systems have many important advantages over enzymatic networks in terms of their compensatory capabilities. For an enzymatic network to be temperature-independent, not only must activation energies be tuned in a precise manner, but other more critical parameters, such as substrate selectivity and overall catalytic efficiency, must also be optimized so that each enzyme remains functional. All of these parameters are governed by substrate–enzyme interactions and are typically controlled by only a few amino acid residues. Therefore, fulfilling the compensation requirement (i.e., matching activation energies) is inherently difficult. In contrast, in a general case of an affinity-based network, where binding thermodynamics is largely decoupled from such complex functional constraints, the compensatory behavior is considerably easier to achieve (whether through natural evolution or artificial engineering), as far fewer parameters must be optimized simultaneously, i.e., only the binding affinities themselves and the concentrations.

### Temperature stabilization in evolutionary context

Although it is unclear whether this thermocompensation mechanism manifests in complex modern organisms, an intriguing possibility is that nature may have exploited this mechanism at much earlier stages of evolution. Currently, the RNA world hypothesis is recognized as one of the fundamental stages in the emergence of life^50^. Evidently, life in such a form was insufficiently developed to actively sustain constant physicochemical conditions. In this context, the demonstrated compensation mechanism may have provided an efficient and evolutionarily simple tool enabling a broader range of permissible environmental conditions. Consequently, this mechanism may have played a crucial role in the development of all subsequent forms of life.

In modern organisms, complex biomolecular interaction networks are becoming increasingly recognized as critical for cellular regulation^49,51^. Both in prokaryotes and eukaryotes, a significant role is played by various small RNAs, whose interactions with mRNA substantially define the cellular protein profile^52^. Furthermore, the extensive diversity of small RNAs within a single cell heightens the probability of their mutual interactions, leading to the formation of complex networks with strongly nonlinear behavior^53^. For successful survival under stressful conditions, the cell must sustain the functioning of this network, as with any other vital system. For example, to protect protein systems, an entire class of protective heat-shock proteins has evolved^54^, mitigating the adverse effects of elevated temperatures on the folding of other proteins. By analogy, certain components of nucleic acid interaction networks may fulfill purely compensatory functions to safeguard these systems.

An interesting question is why, if early living systems hypothetically achieved temperature independence via this mechanism, many existing organisms (perhaps their evolutionary descendants) nonetheless exhibit significant sensitivity to changing temperature. One possible explanation is the emergence of dedicated homeostatic mechanisms, which may have relieved molecular networks from the requirement for self-compensation, leading to gradual repurposing of compensatory participants.

As highlighted in Fig. 8, more complex organisms may have adapted this temperature-response mechanism as a highly effective means of systemic signaling coupled to changes in body temperature (e.g., during disease). This may have enabled distinct cell types to independently evolve specialized responses (such as modulating metabolism or triggering particular signaling cascades) while contributing to a coordinated organism-level outcome. It is possible that the universality and flexibility of such signaling could confer significant evolutionary advantages over mechanisms relying solely on chemical signaling via mass transport.

### Limitations

We should note that our work relies on NUPACK calculations, which in some cases might be far from precise (for example, see screening in Fig. 3B). Does this imply that our conclusions drawn from its predictions are incorrect? From the fundamental point of view of the compensation feasibility, the only condition required is the existence of biomolecules exhibiting sufficiently distinct temperature-dependent behaviors, which is definitely true both for nucleic acids and proteins, and most likely, for any other biomolecules.

On the other hand, the phenomenon of compensation (like molecular commutation signal processing more broadly) can be considered both at the level of affinity matrices and at the level of the real molecules that realize these matrices. If we consider only the first level (affinity matrices and law-of-mass-action equations), the compensation is exact, and no additional assumptions are required. In this context, NUPACK and similar prediction tools limit us only in this second aspect of correctly identifying which specific molecules should be chosen to achieve a desired matrix.

This limitation can be addressed in several ways. For example, one may restrict attention to specific subsets of oligonucleotides for which NUPACK performs reliably: for example, as observed in the screening shown in Supplementary Fig. 11, prediction accuracy was noticeably higher than in Fig. 3B, possibly due to shorter sequence lengths. Alternatively, specialized design strategies can be employed, such as the use of domain-based architectures for oligonucleotide-based tasks.

A related question is whether certain target matrices might be inherently unrealizable with the chosen type of molecules. In our view, the diversity of biomolecules (oligonucleotides or proteins) and their affinity continuum^24^ is so vast that this should not pose any limitation. Moreover, this continuum can be expanded in an almost unlimited manner—for example, through the use of non-canonical or chemically modified bases as monomers, or by employing molecules with nontrivial secondary or tertiary structures. The latter, for example, is intrinsic to proteins and can also be achieved with DNA (for example, see metaDNA^55^).

Finally, our work highlights the importance of further developing predictive tools, particularly with an emphasis not only on high-affinity systems and structure prediction, but also on the quantitative characterization of weak interactions: as demonstrated in this and our previous studies^24^, such interactions carry substantial and still unexplored biological significance.

### Outlook

The described mechanism of topological compensation by affinity-based networks enables the existence of non-insulated molecular systems that can perform signal processing in a manner inherently resistant to dramatic fluctuations in physical and chemical conditions. The fundamental nature of this self-sustaining in molecular commutation systems suggests that they may play an important regulatory role in a wide variety of living organisms, while the simplicity of their design implies the possibility of their spontaneous evolutionary emergence. Further investigation and development of such systems may not only give us a better understanding of molecular signaling in living organisms, but also inspire the design of robust biotechnological processes that are more productive, compact, and less demanding of complex instrumentation to maintain constant production conditions.

## Methods

### Experimental section

#### Reagents

RiOs-DI 3 water (Merck Millipore, Burlington, MA, USA) was used for the preparation of aqueous solutions.

The reagents were obtained from the following suppliers: Tris(hydroxymethyl)aminomethane (Dia-M, Moscow, Russia); sodium chloride (Helicon, Moscow, Russia); ethylenediaminetetraacetic acid disodium salt dihydrate, tween 20, magnesium chloride 6-hydrate (Panreac AppliChem, Darmstadt, Germany); triethylammonium acetate (Pallav Chemicals, Mumbai, Maharashtra, India); lithium perchlorate (Thermo Fisher Scientific, Waltham, MA, USA); acetonitrile (Concord Technology, Tianjin, China); acetone (Component Reactiv, Moscow, Russia); β-cyanoethyl-N,N-diisopropylphosphoramidites of 2′-deoxynucleosides (Hongene Biotech, Shanghai, China).

For temperature-independent systems (Figs. 3 and 4 and Supplementary Figs. 8 and 9), oligonucleotide solutions were prepared in a buffer composed of 0.1 M Tris, 1 M NaCl, 0.1 mM EDTA, 0.1 g/L Tween 20, pH 8.

For salt-independent systems (Fig. 5 and Supplementary Figs. 11 and 12), oligonucleotide solutions were prepared in a buffer composed of either NaCl or MgCl_2_ at the desired concentration and 0.01 M Tris, 0.1 g/L Tween 20, pH 8.

#### Oligonucleotide synthesis and purification for Figs. 4 and 5C, D and Supplementary Figs. 8, 11, and 12

Oligonucleotides were synthesized on an ASM-32 automated DNA/RNA synthesizer (BIOSSET Ltd., Novosibirsk, Russia) using standard β-cyanoethyl phosphoramidite chemistry at a 100–200 nmol scale. Standard β-cyanoethyl-N,N-diisopropylphosphoramidites of 2′-deoxynucleosides and universal Controlled Pore Glass solid support with 1000 Å pore size (Lumiprobe, Moscow, Russia) were used. Modifications with 5′-Cyanine3 were introduced using the corresponding phosphoramidite monomers (Lumiprobe (Moscow, Russia)), and modifications with 3′-DusQ2 (BHQ2) were introduced using the pre-functionalized solid supports (Lumiprobe (Moscow, Russia)) according to the manufacturer’s protocols.

Purification of crude 5′-labeled oligonucleotides was performed by ion-pair reversed-phase HPLC on an Agilent 1260 Infinity II system (Agilent Technologies, Santa Clara, CA, USA) equipped with an Alphasil VC-C18AQ column (250 × 4.6 mm, 5 µm, 100 Å; Acchrom Technologies, Wenling, China). Separation was carried out using a linear gradient of 0–70% acetonitrile in 20 mM triethylammonium acetate, pH 7.0, over 30 min at a flow rate of 1.0 mL/min. Purification of crude unmodified and 3′-modified oligonucleotides was performed by precipitation with 3% w/v LiClO4 in acetone. Samples were centrifuged at 15,000 × *g* (5 min) and washed twice with acetone.

#### Oligonucleotide synthesis for Fig. 3A, B and Supplementary Fig. 9

Oligonucleotides were synthesized by Lumiprobe (Moscow, Russia). Oligonucleotides modified with fluorophores or fluorescence quenchers were purified by polyacrylamide gel electrophoresis, while unmodified oligonucleotides were purified using C18 cartridges.

#### Logic gates

Concentrations, affinities, sequences and other parameters of all logic gates and other systems are given in Supplementary Methods, except for the gates in Fig. 6D and Supplementary Figs. 3, 14, and 15, whose parameters are provided in a Source Data file.

#### Fluorescence measurements

The fluorescence measurements for Figs. 3A, B and 5C, D and Supplementary Fig. 11 were performed using a Clariostar spectrophotometer (BMG Labtech, Ortenberg, Germany). Excitation and emission wavelengths were set to 530 nm (bandwidth 20 nm) and 580 nm (bandwidth 30 nm), respectively.

The fluorescence measurements for Fig. 4 and Supplementary Figs. 8, 9, and 12 were performed using a LumoTrace FLUO (Abisense, Sochi, Russia) bioimaging system equipped with a 96-well plate temperature control module using Micromanager^56^ on Icy^57^ software with Abisense plugins. Fluorescence was captured using a 1-s exposure time, a 550 ± 25 nm bandpass excitation filter and a 600 nm longpass emission filter.

#### Affinity measurement (Fig. 3B and Supplementary Fig. 11)

For Fig. 3B, a 40 µL mixture of oligonucleotides S and Q was added to 40 µL of oligonucleotide I, resulting in final concentrations of 50 nM for S, 200 nM for Q, and 800 nM for I. The mixture was incubated at 50 °C for 1 h, after which fluorescence was measured using a Clariostar spectrophotometer preheated to 50 °C. Samples were then allowed to cool to room temperature for 2 h, after which fluorescence measurements were repeated.

For Supplementary Fig. 11, concentrations of 100 nM for S and Q, and 1000 nM for I were used. The mixtures were incubated for 1 h at room temperature and fluorescence was measured using a Clariostar spectrophotometer.

The dissociation constant *K*_*d*_(SQ) was determined from the fluorescence level of the S and Q mixture in the absence of I. Using this value, the fluorescence levels of the samples containing I were converted to corresponding dissociation constants *K*_*d*_(IQ). These constants were then converted to arbitrary C_AB_ units, which are plotted in Fig. 3B and Supplementary Fig. 11 according to the following relation:

5 $${K}_{d}=\frac{(1\mu M-{C}_{{AB}}\left(\,\mu M\right))^2}{{C}_{{AB}}\left(\,\mu M\right)}.$$

#### Melting curve measurements (Fig. 4 and Supplementary Fig. 9)

After mixing oligonucleotides in a 96-well plate at the concentrations provided in Supplementary Methods, the plate was incubated at 50 °C for 1 h and then allowed to cool to room temperature for 1 h. Melting curves were subsequently recorded using the LumoTrace FLUO (Abisense, Sochi, Russia) bioimaging system equipped with a 96-well plate temperature control module, with a heating rate of 0.15 °C/min from room temperature (20–25 °C) to 50 °C.

#### Kinetic measurements (Supplementary Figs. 8 and 12)

Mixtures containing all oligonucleotides except the inputs were prepared and allowed to equilibrate for 1 h at room temperature (concentrations are listed in Supplementary Methods). Inputs were then added, and fluorescence was recorded using the LumoTrace Fluo bioimaging system once every 30 s (for salt-independent gates) or once per minute (for temperature-independent gates) for 30–60 min. The time between the first input addition and the start of measurement did not exceed 1 min.

#### Statistical analysis

No blinding or randomization was employed because the in vitro experiments involved objective instrument-recorded fluorescence measurements and identical analysis workflows across all samples.

No statistical method was used to predetermine sample size. The number of technical replicates was selected based on the controlled nature of the biochemical assays, the objective fluorescence readout, and standard practice for comparable in vitro measurements. Screening experiments were performed with two technical replicates to select candidate for further characterization; main experiments were performed with three technical replicates to quantify technical variability and report mean ± s.d. Statistical calculations were not performed for experiments with *n* < 3. Exact replicate numbers are provided in the figure legends.

Reagent concentrations, incubation conditions, temperature, and measurement settings were kept consistent across compared samples. No additional covariates were included in the statistical analyses.

### Computational section

#### Software

The following software was used for data generation, analysis and visualization: custom Python 3.10.18 scripts for UBUNTU 24.04 with NUPACK 4.0.1.12 (generation of DNA or RNA sequences and analysis of circuit performance), pyBADS 1.0.5 (bayesian optimization), DEAP 1.4.3 (evolutionary optimization) matplotlib 3.10.5 and seaborn 0.13.2 (plots), numpy 2.2.6 and pandas 2.3.2 (general data arrays manipulations), networkx 3.4.2 (interaction graphs storage and analysis), Icy 2.4.2.0 (image brightness/contrast; lookup table), Inkscape 1.4.2 (drawing of the figures).

The full custom code is available via GitHub (github.com/korenkov-es/molec_comut_temp) and Zenodo (10.5281/zenodo.21260296).

#### NUPACK predictions

The NUPACK package for the Python programming language (version 4.0.1.12) was employed in this study. The equilibrium constants were computed according to ref. ^58^:

6 $$\begin{array}{c}{K}_{d}={c}_{{H}_{2}O}\frac{{Z}_{s1}{Z}_{s2}}{{Z}_{s1s2}}\end{array},$$

where the indices s1 and s2 refer to the interacting oligonucleotides, *Z* denotes the partition function of the respective complex, and ${c}_{{H}_{2}O}$ is the molar concentration of water. The partition function was computed as the pfunc attribute of the nupack.complex_analysis object. Unless otherwise specified, the max_compl_size parameter was set to 2. Given the known dissociation constants and initial concentrations of oligonucleotides, the equilibrium concentrations of complexes were computed using the iterative method described by ref. ^59^. Briefly, the equilibrium concentrations (*x*_*i*_) were updated iteratively according to the following fixed-point map:

7 $${x}_{i}^{\left(n\right)}=\frac{{c}_{i}}{1+{\sum }_{i\ne j}{K}_{{ij}}{x}_{j}^{\left(n-1\right)}},\,{x}_{i}^{\left(0\right)}={c}_{i},$$

where *c*_*i*_ denotes the total concentrations, *K*_*ij*_ denotes the association constants (i.e., the inverse of the dissociation constants), *n* denotes current iteration. The iteration process stopped when the following condition was satisfied:

8 $${||}{x}^{\left(n\right)}-{x}^{\left(n-1\right)}{{||}}_{\infty }\le {atol}+{rtol}{{||}{x}^{\left(n\right)}{||}}_{\infty },$$

where atol = 10^−12^, rtol = 10^−6^.

Hairpin formation energy was calculated as the minimum.mfe attribute of the nupack.complex_analysis object across all possible structures consisting only of the strand of interest.

In calculations using max_compl_size = 3, dissociation constants were not used to simulate the system. Instead, the entire system was simulated in NUPACK, including the calculation of equilibrium concentrations of complexes.

#### Approximation of the temperature dependence of oligonucleotide dissociation constants

For the analysis of this relationship, we generated 10,000 pairs of 20-mer oligonucleotides. Each duplex contained between 1 and 10 mismatches, and the secondary-structure folding free energy of all oligonucleotides was constrained to zero.

For each pair, we determined the dissociation constant at 25 and 50 °C, converted these values to Gibbs free energies, and, assuming enthalpy and entropy to be temperature-independent, computed the corresponding enthalpies and entropies. Each oligonucleotide pair is therefore characterized by two parameters: the Gibbs free energy at 25 °C (G25) and the enthalpy (*H*).

We then constructed the frequency distribution of observed G25 values and partitioned it into 200 equal bins. For each bin (within which G25 varies negligibly), we derived the frequency distribution of *H*. From each such *H* distribution, we extracted the 5th, 10th, 20th, …, 80th, 90th, and 95th percentiles. By aggregating these percentile data across all G25 bins, we obtained the dependence of each percentile on G25, which we subsequently approximated using cubic B-splines (coefficients of these splines are listed in Supplementary Table 1). Examples of obtained data and resulting approximations are given in Supplementary Fig. 16 for 5th, 50th, and 95th percentiles.

This approach enables prediction of possible enthalpy values, and hence Gibbs free energies at arbitrary temperatures, for any given G25. For example, the spline corresponding to the 5th percentile (p5) predicts enthalpies for the most “easy-to-melt” duplexes, whereas the 95th-percentile spline (p95) applies to the most “hard-to-melt” duplexes. These cubic-spline functions are denoted as p5, p10, etc.

#### PyBADS optimization

During PyBADS optimization, only numerical parameters were optimized, namely concentrations, affinities (all figures) and easy-to-melt levels (only Fig. 8). PyBADS optimization uses a hybrid approach, combining poll and search stage. Briefly, in the poll stage, points are evaluated on a mesh by taking steps in one direction at a time, until an improvement is found or all directions have been tried. In the search stage, a Gaussian process surrogate model of the target function is fit to a local subset of the points evaluated so far. All internal optimization parameters, including the definitions of priors in the second step, are described in detail in the original papers^37,38^.

Please note that, in some cases, a slight improvement in the objective can be achieved by rerunning the optimization one or two times, using the optimized values from the previous step as initial points. It is also advantageous to run multiple optimizations in parallel and select the run that yields the best result. Typically, for simpler systems (such as a YES gate), a single run is sufficient, whereas more complex systems (such as algebraic systems) may require 24–48 parallel runs.

In Fig. 8, where easy-to-melt levels were also tuned (in addition to dissociation constants and concentrations), they were optimized as a continuous parameter in the range [0, 10] and then converted to the nearest integer to yield the easy-to-melt level.

Below, we provide formal definitions of the objective functions optimized in this step. We use the following notation:

- *T* is the set of temperatures of interest; for most cases, *T* = {25, 50} °C.
- Λ is the set of all possible logic input states. For one-input gates, *Λ* = {0, 1}; for the AND-gate *Λ* = {00, 01, 10, 11}.
- *p* is the set of all system parameters, such as concentrations, dissociation constants, sequences, etc.
- *S*(*t*, *p*, *i*) is the concentration of output oligonucleotide in free form at temperature *t* at input state *i*.
- ${S}_{i}^{{target}}$ is the desired concentration of the output oligonucleotide in free form at input state *i*.

Then, for logic gates, the objective function is defined as:

9 $$L\left(p\right)={\sum }_{t\in T}{\sum }_{i\in \varLambda }\left|S\left(t,p,i\right)-{S}_{i}^{{target}}\right|.$$

For a receptor-activator system, *j* is the receptor index, *N* is the total number of receptors, ${R}_{j}^{{target}}$ is the desired level of activation of the *j*-th receptor, ${R}_{j}^{{act}}\left(t\right)$ is the activation level of the *j*-th receptor at temperature *t*. The objective function is defined as:

10 $$L\left(p\right)={\sum }_{t\in T}{\sum }_{j=1}^{N}\left|{R}_{j}^{{act}}\left(t,p\right)-{R}_{j}^{{target}}\right|.$$

For algebraic systems (Fig. 7), *x* is the normalized and centered input concentration (see main text), *X* is the set of all *x* of interest, *f*(*x*) is a user-defined function. The objective function is defined as:

11 $$L\left(p\right)=\sqrt{{\sum }_{t\in T}{\sum }_{x\in X}{\left(S\left(t,p,x\right)-f\left(x\right)\right)}^{2}}.$$

For molecular commutation systems with programmable temperature/ionic-strength dependence (Fig. 8), *f*(*t*) is a user-defined function. The objective function is defined as:

12 $$L\left(p\right)={\sum }_{t\in T}\left|S\left(t,p\right)-f\left(t\right)\right|.$$

#### Sequence finding optimization

Sequence selection was performed using an evolutionary algorithm using the DEAP Python library^60^. At this stage, the target Gibbs free energies $\varDelta {G}_{{ij}}^{{expected}}$ of hybridization at the specified temperatures (typically 25 and 50 °C) are assumed to be known for all pairs of oligonucleotides in the system.

The algorithm proceeds iteratively, constructing one oligonucleotide at a time. The first oligonucleotide is initialized randomly.

We now describe the procedure for designing the *N*-th oligonucleotide, assuming that the preceding *N*–1 oligonucleotides have already been determined. The initial guess for this oligonucleotide was defined as the reverse complement of the oligonucleotide that must exhibit the strongest binding affinity to the sequence being designed:

13 $${{seq}}_{N}^{{start}}={rev}.{compl}\left({{seq}}_{m}\right),\,m={{argmin}}_{j=1...N-1}\left(\varDelta {G}_{{jN}}\right).$$

The sequences in the initial population were generated from this starting sequence by introducing between one and four random mutations. Subsequently, during the evolutionary process, each oligonucleotide was either modified by introducing between one and six random mutations or reinitialized by applying one to two random mutations to the starting sequence. After evaluating the objective function for all members of the population, the next generation was constructed using random tournament selection with a tournament size of three.

The objective function was defined as the sum of absolute deviations between the Gibbs free energies of hybridization (at all target temperatures) with the previously found *N*–1 oligonucleotides evaluated by NUPACK and the corresponding target Gibbs energy values:

14 $$L={\sum }_{t\in \{25^\circ C,50^\circ C\}}{\sum }_{i=1...N-1}\left|\varDelta {G}_{iN}^{{expected}}\left(t\right)-\varDelta {G}_{iN}^{{NUPACK}}\left(t\right)\right|.$$

In cases where no interaction between oligonucleotides *i* and *j* was desired (i.e., an off-target interaction), the Gibbs free energy of hybridization of the selected sequences was required to remain below a threshold defined as follows. Off-target interactions had to be at least k_atten-fold weaker (in terms of *K*_*d*_) than the weakest target interaction (as determined in the previous parameter-optimization step). If an off-target interaction was weaker than this threshold, it contributed zero to the loss function; otherwise, a large penalty was imposed that was guaranteed to exceed all other terms in the loss function.

The parameter k_atten was specified independently for low and high temperatures and denoted as lowtemp_ka_atten and hightemp_ka_atten, respectively. Additionally, if the dissociation constant for oligonucleotide self-dimerization fell below 5 × 10⁻⁵ M, a penalty term exceeding all other components of the loss function was added. Finally, the loss function included the absolute value of the Gibbs free energy of secondary structure formation for the given oligonucleotide, divided by the parameter hairpin_atten.

Unless otherwise specified, the following optimization parameters were used: sequence length = 20, population size = 280, number of generations = 100, lowtemp_ka_atten = 10^5^, hightemp_ka_atten = 10^4^, hairpin_atten = 5, early stop condition = 1 kJ/mol.

If, upon reaching the maximum number of generations, the error still exceeded the threshold, all *N*–1 sequences identified up to that point were discarded, and the optimization was restarted from scratch.

#### System fine-tuning to account for non-zero concentrations of triple complexes

In all simulations involving NUPACK (Figs. 2 and 8 and Supplementary Figs. 4–7, and 10), an additional optimization step was performed to ensure that the equilibrium concentrations of the output oligonucleotide attained their target values for all possible input states when using max_compl_size = 3 or higher.

The objective function at this stage was analogous to that used in the PyBADS optimization section. During this refinement procedure, evolutionary sequence optimization steps were alternated with Bayesian optimization of concentrations. The concentration-optimization parameters were identical to those used during the initial system design (listed in Supplementary Methods), with the exception of the maximum number of objective function evaluations, which was limited to 100 (no such restriction was imposed at the initial stage).

For sequence fine-tuning, a population size of 192 was employed. At each evolutionary step, between one and three random mutations were introduced into each sequence with a probability of 50% per oligonucleotide.

#### Identification of molecular commutation systems with predefined temperature and ionic-strength dependencies (Fig. 8)

For temperature-dependent functions, temperature was normalized as described in the main text to yield *X*. The target functions were then formulated in terms of *X*:

Linear:

15 $$Y\left({nM}\right)=20+5\cdot \left(2X-1\right).$$

Quadratic:

16 $$Y\left({nM}\right)=20+5\cdot {\left(2X-1\right)}^{2}.$$

Cubic:

17 $$Y\left({nM}\right)=20+5\cdot {\left(2X-1\right)}^{3}.$$

Sine:

18 $$Y\left({nM}\right)=20+5\cdot \sin \left(2\pi X\right).$$

For ionic strength, the following normalization was used:

19 $${X}_{{ionic}}={\log }_{10}\left(a\left[{{Na}}^{+}\right]+b\right).$$

The following values of the coefficients a and b were used so that the [0.05; 1] *M* range of [Na^+^] is mapped onto the [0; 1] interval:

20 $$a=\frac{9}{\left(1-0.05\right)}\approx 9.474,b=10-\frac{9}{\left(1-0.05\right)}\approx 0.526.$$

The optimization for the ionic-strength case was done as follows. We identified the temperature increase from 25 °C at which the average change in dissociation constants is comparable to that observed when the Na⁺ concentration varies over the range [0.05, 1] M. Accordingly, the temperature range of 25–41 °C was selected. Then, the oligonucleotide set exhibiting a cubic temperature dependence within this reduced temperature range was identified as described in the main text. Subsequently, the resulting sequences and concentrations were fine-tuned so that the ionic-strength dependence follows a cubic function (with an inverted sign):

20 $$Y\left({nM}\right)=20-5\cdot {\left(2{X}_{{ionic}}-1\right)}^{3}.$$

### Reporting summary

Further information on research design is available in the Nature Portfolio Reporting Summary linked to this article.

## Supplementary information

Supplementary Information

Reporting Summary

Transparent Peer Review file

## Source data

Source Data

## Data Availability

All data generated or analysed during this study are included in this published article (and its supplementary information files). The full source code is available via GitHub (github.com/korenkov-es/molec_comut_temp) and Zenodo (10.5281/zenodo.21260296). All data are also available upon request. The Supplementary Information file contains Supplementary Figs. 1–16, Supplementary Table 1, Supplementary Notes 1–4, and Supplementary Methods; corresponding references to these elements are provided in the main text. Source data are provided with this paper.

## Code availability

The full source code is available via GitHub (github.com/korenkov-es/molec_comut_temp) and Zenodo (10.5281/zenodo.21260296).
