# Supplementary material for: Enabling molecular signaling with temperature and ionic-strength independence or programmable dependence
Source: Nat Commun. 2026 Aug 7;17:8895. doi: 10.1038/s41467-026-76207-x (PMC13503822; doi:10.1038/s41467-026-76207-x)
Supplement: Supplementary file 1 [file 41467_2026_76207_MOESM1_ESM.pdf]

SUPPLEMENTARY INFORMATION

Enabling molecular signaling with temperature and ionic-strength independence or programmable dependence

Egor S. Korenkov, Maxim P. Nikitin*

*Corresponding author
E-mail: nikitin.mp@talantiuspeh.ru, max.nikitin@gmail.com

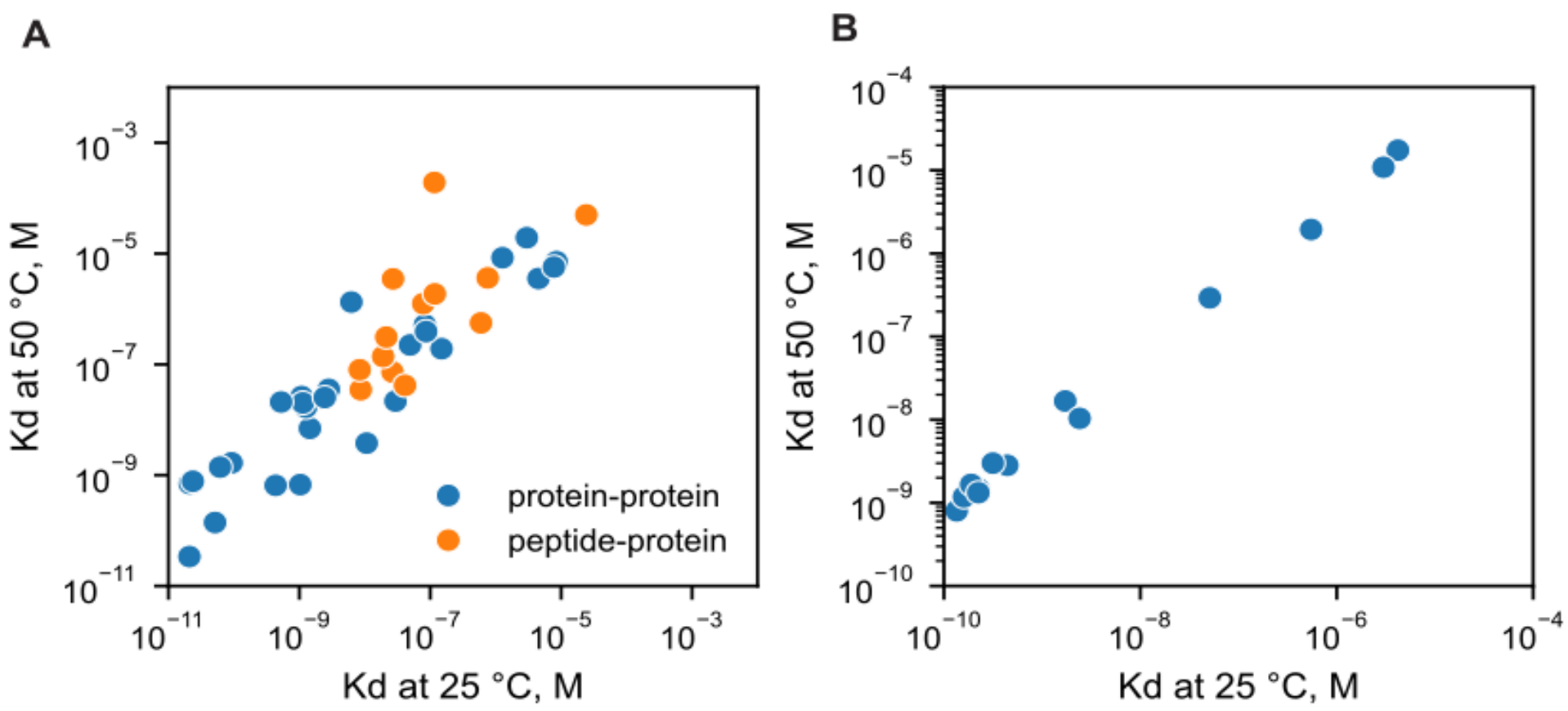

Supplementary Fig. 1. Kd distributions at 25 and 50 °C for protein-protein interactions for (A) various protein-protein and protein-peptide pairs from Stites 1997[1] for (B) single-point mutational scan of an anti-HER2 antibody from Kelley 1993[2]

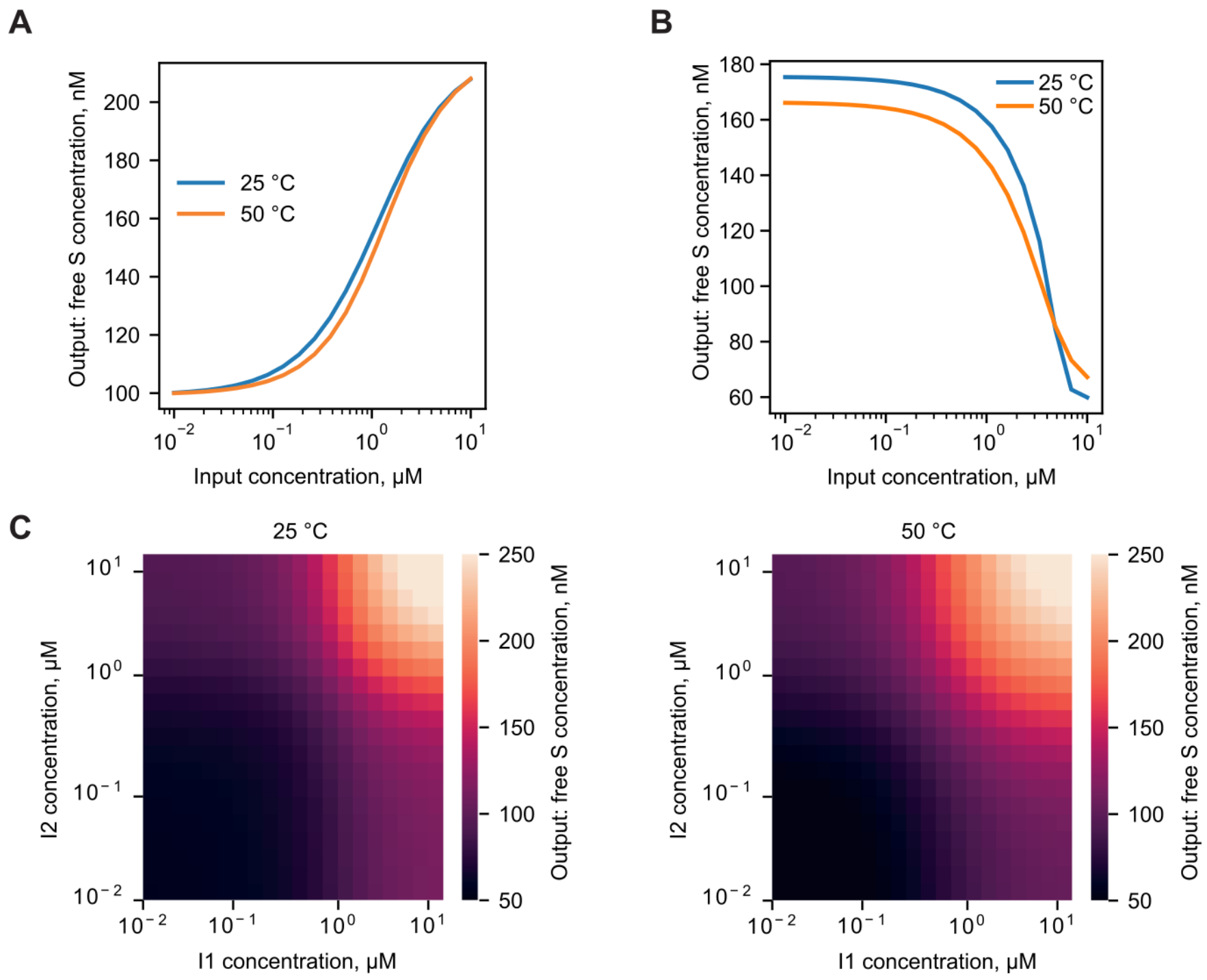

Supplementary Fig. 2. Logic gates performance under different input oligonucleotides concentrations. (A) YES-gate; (B) NOT-gate; (C) AND-gate.

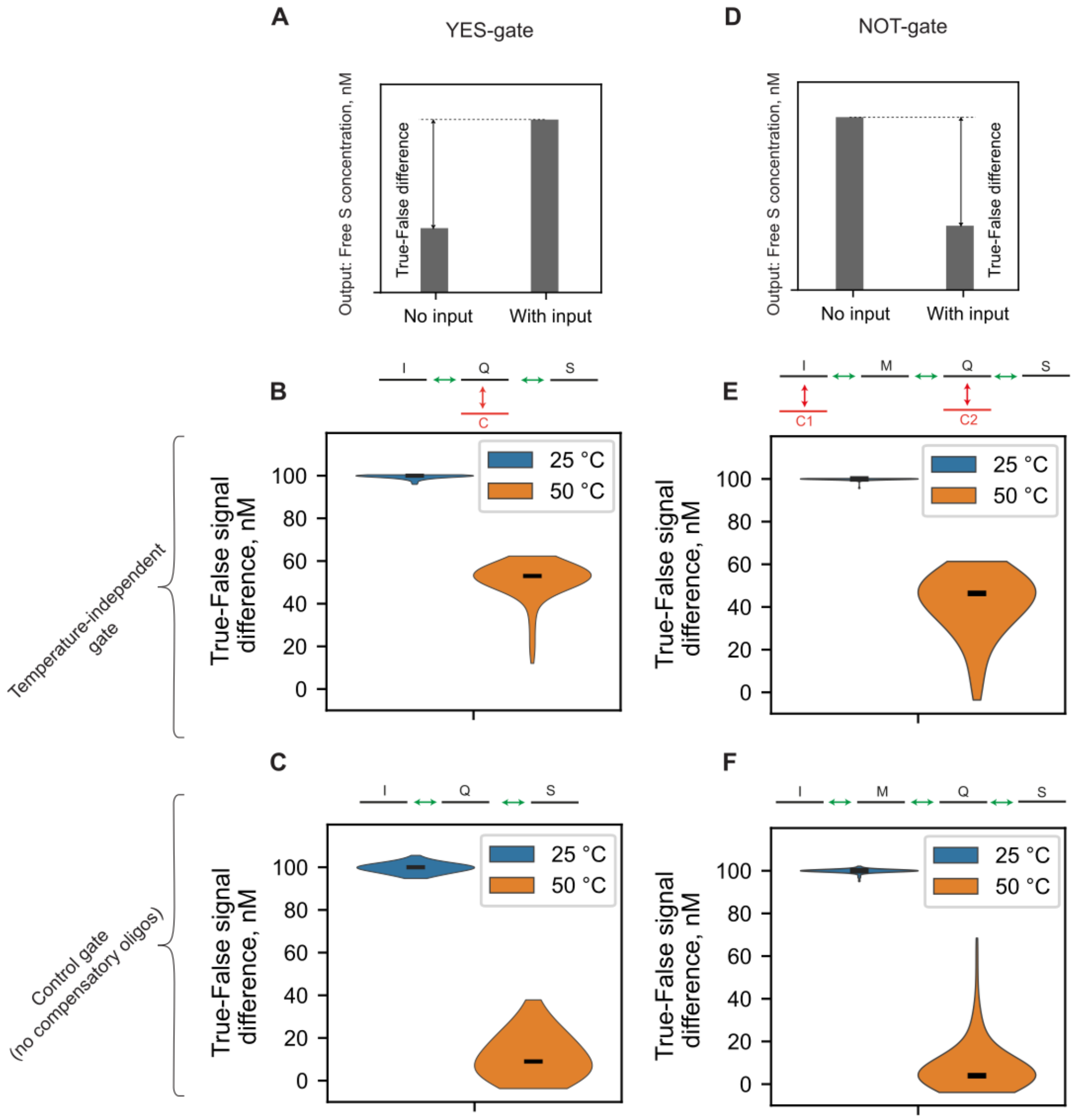

Supplementary Fig. 3. Sufficiency of compensatory topology alone for stabilizing logic gates under temperature variation. (A, D) Visualization of True-False signal difference for YES and NOT gates. Distribution of this True-False signal difference (i.e., with inputs vs. without inputs) at 25 and 50 °C is shown for 100 (B) YES gates and (E) NOT gates. Panels (C, F) show the same distribution for the control YES and NOT gates (i.e., without compensatory oligos). Black lines show the median values. See Supplementary Note 2 for a detailed description.

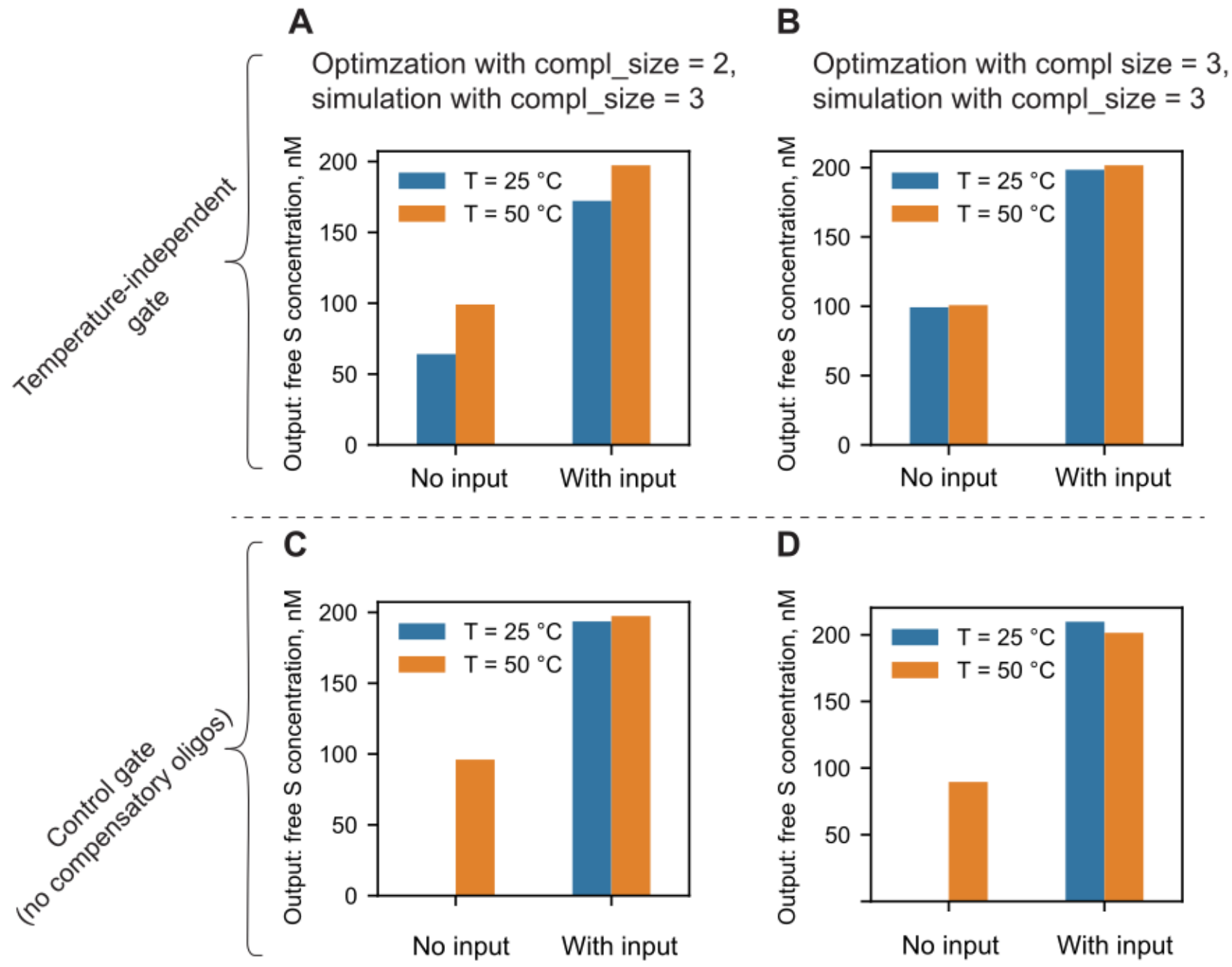

Supplementary Fig. 4. Performance of a temperature-independent and control YES logic gates, simulated in NUPACK while accounting for complexes with up to three strands (compl_size = 3). For (A), the exact gate parameters (sequences and concentrations) from Fig. 2A were used, where only complexes with up to two strands were considered during optimization. Gate in (B) represents the same system from (A), fine-tuned to perform while accounting for complexes with up to three strands. (C) and (D) show simulations of control systems, identical to those in (A) and (B), respectively, but without compensatory species.

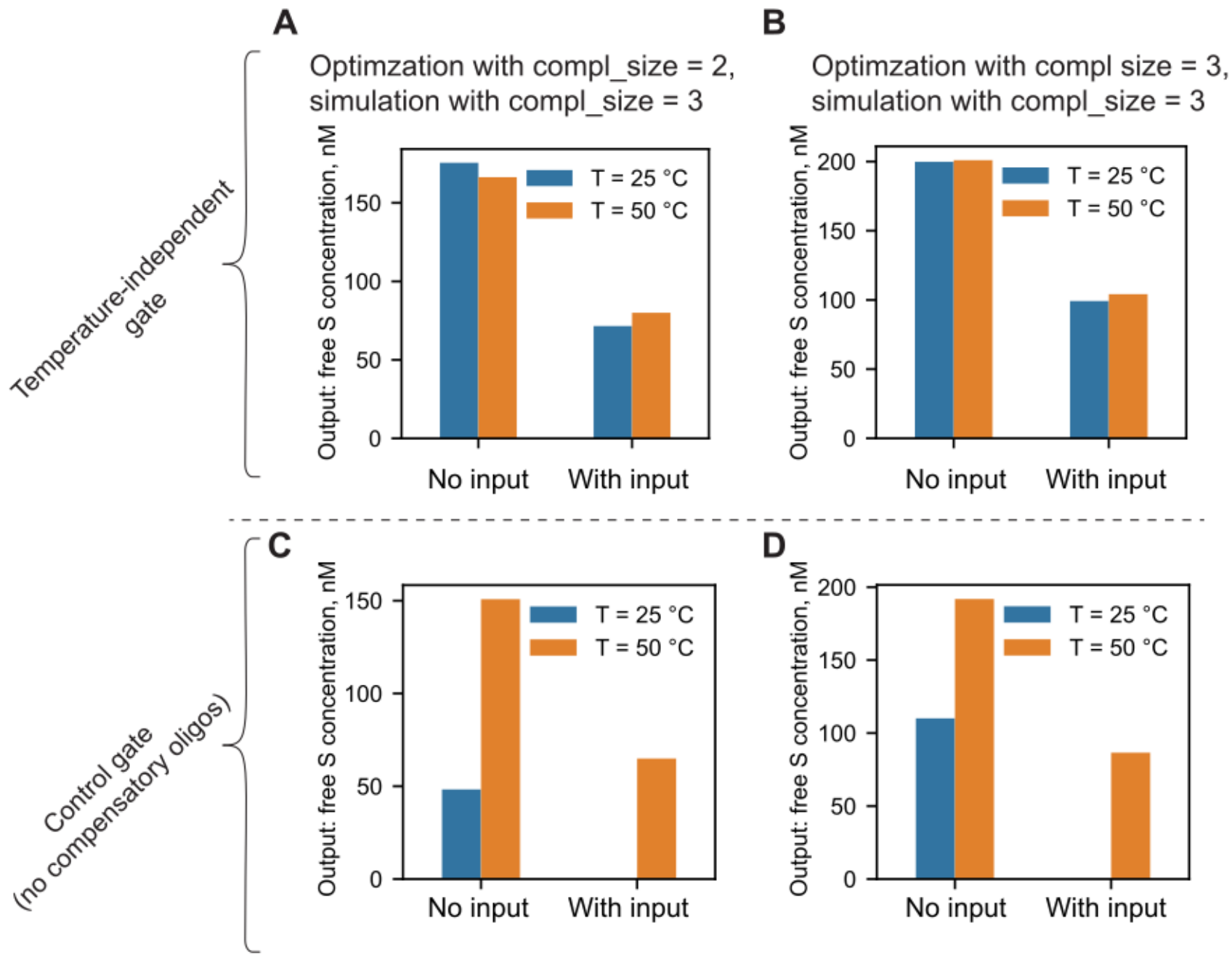

Supplementary Fig. 5. Performance of a temperature-independent and control NOT logic gates, simulated in NUPACK while accounting for complexes with up to three strands (compl_size = 3). For (A), the exact gate parameters (sequences and concentrations) from Fig. 2B were used, where only complexes with up to two strands were considered during optimization. Gate in (B) represents the same system from (A), fine-tuned to perform while accounting for complexes with up to three strands. (C) and (D) show simulations of control systems, identical to those in (A) and (B), respectively, but without compensatory species.

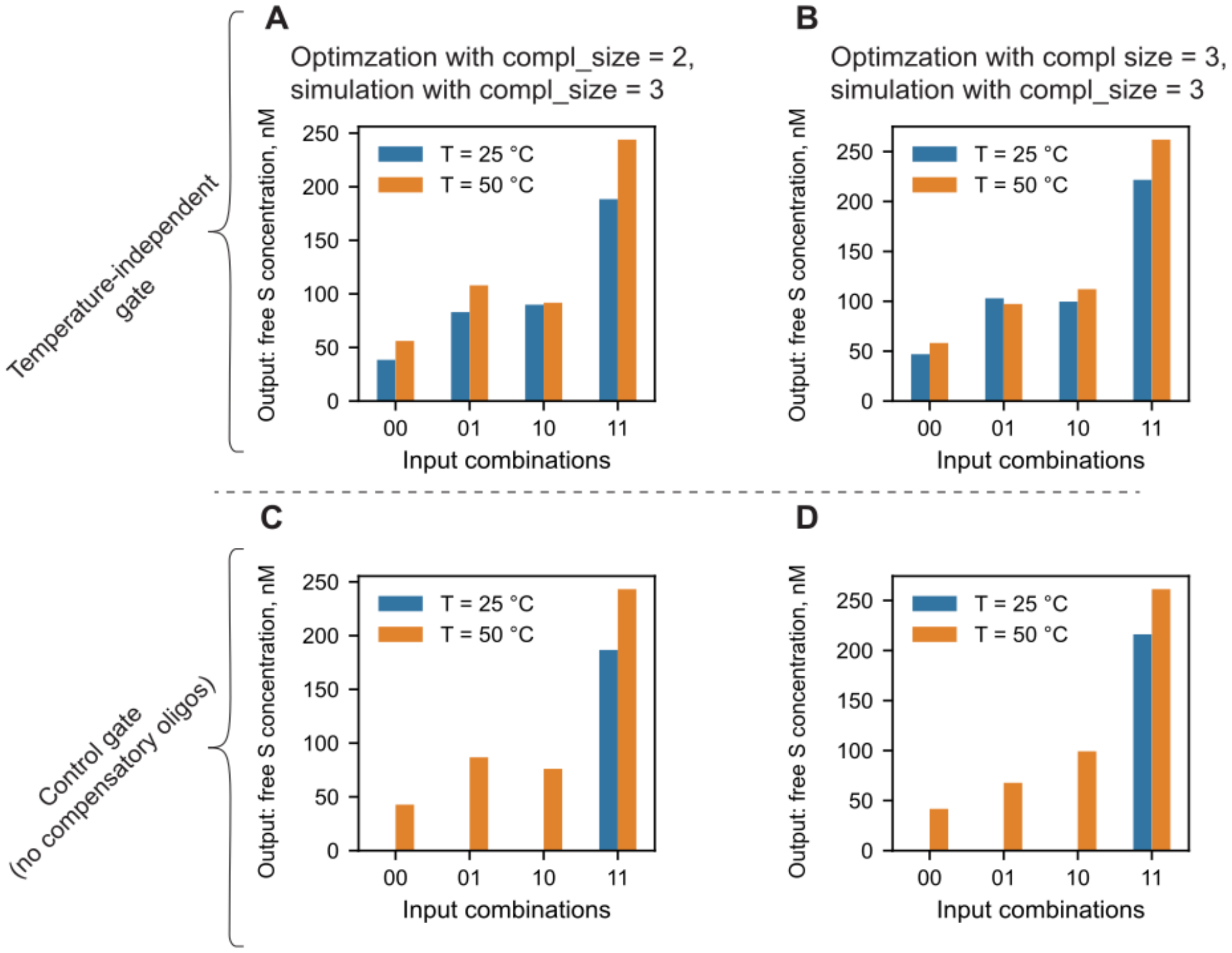

Supplementary Fig. 6. Performance of a temperature-independent and control AND logic gates, simulated in NUPACK while accounting for complexes with up to three strands (compl_size = 3). For (A), the exact gate parameters (sequences and concentrations) from Fig. 2C were used, where only complexes with up to two strands were considered during optimization. Gate in (B) represents the same system from (A), fine-tuned to perform while accounting for complexes with up to three strands. (C) and (D) show simulations of control systems, identical to those in (A) and (B), respectively, but without compensatory species.

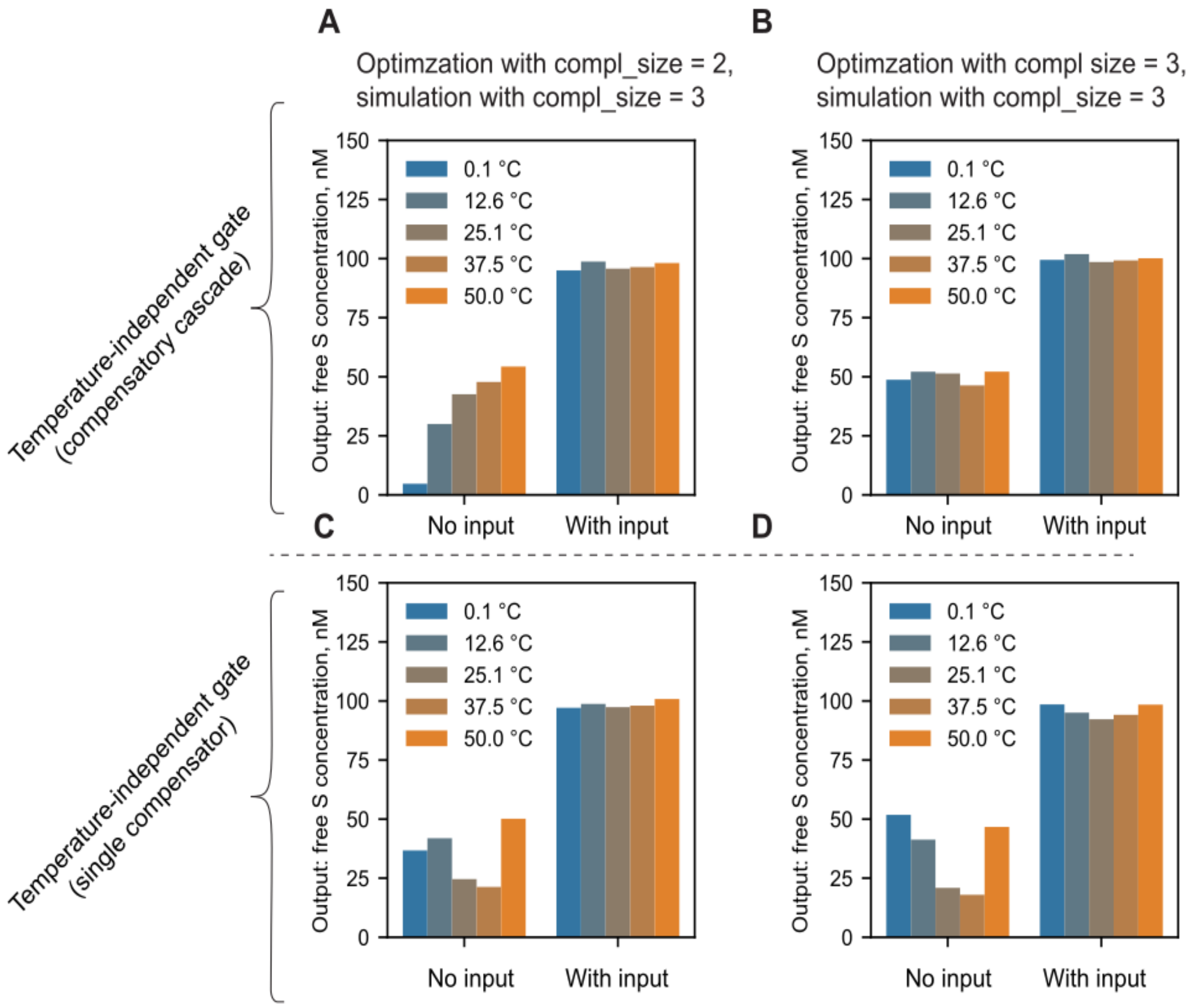

Supplementary Fig. 7. Performance comparison of a temeperature-independent YES logic gate with compensatory cascade and gate with a single compensator (0.1-50 °C range), simulated in NUPACK while accounting for complexes with up to three strands (compl_size = 3). For (A, C), the exact gate parameters (sequences and concentrations) from Fig. 2D and E were used, where only complexes with up to two strands were considered during optimization. Gates in (B, D) represents the same systems from (A, C) respectively, fine-tuned to perform while accounting for complexes with up to three strands.

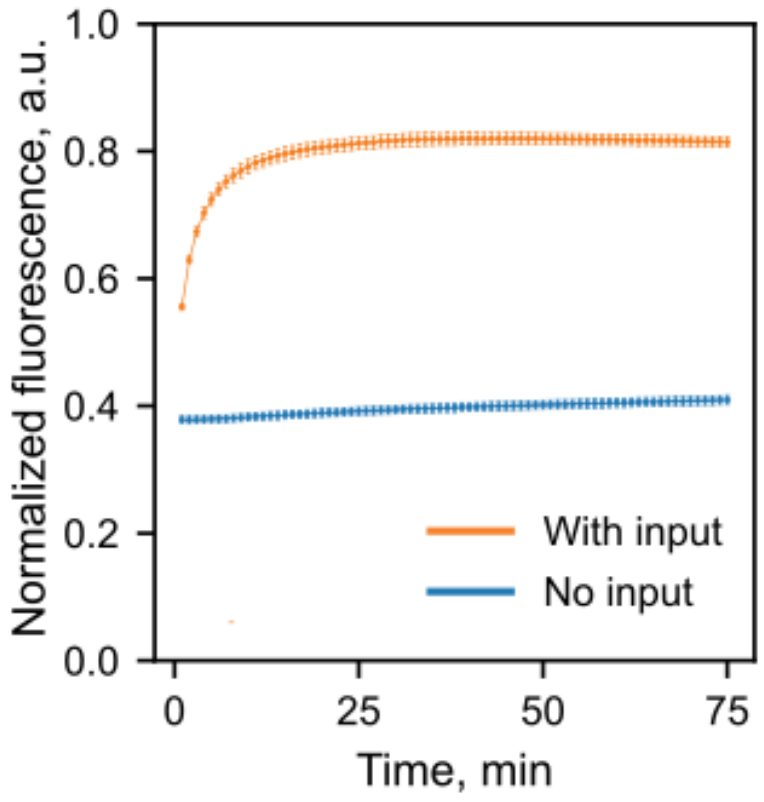

Supplementary Fig. 8. Kinetics of the temperature-independent YES gate from Fig. 4 following the addition of the input oligonucleotide. Other components (S, Q, and C) were pre-equilibrated for 1 hour. Data are presented in relative fluorescence units as mean ± s.d., n = 3 independent samples. Fluorescence is normalized to the signal of a sample containing only S at the same concentration.

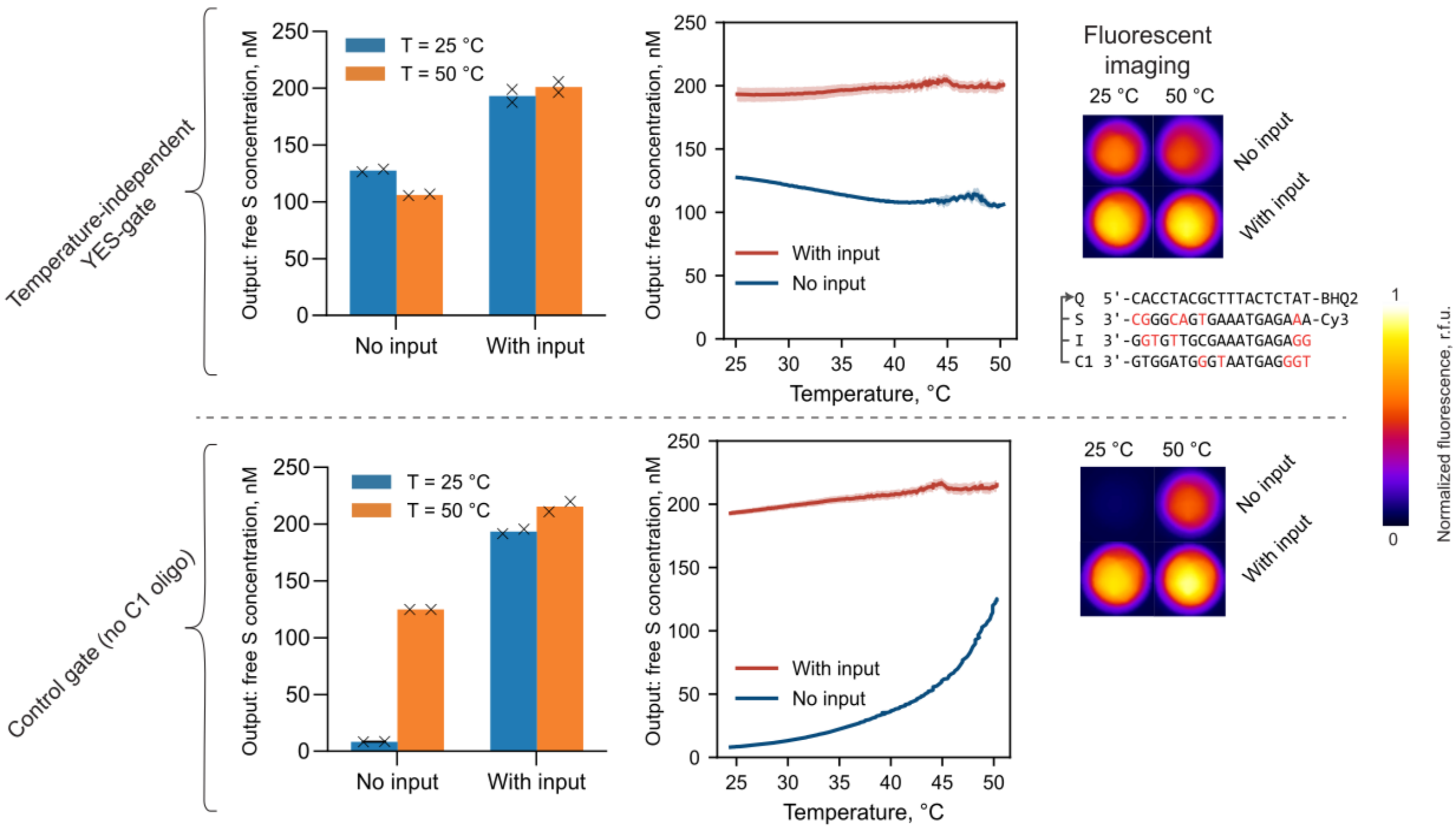

Supplementary Fig. 9. This figure is replicate of Fig. 4 with other oligonucleotides from other manufacturer (Lumiprobe, Moscow, Russia). Experimental performance of the temperature-independent and control YES gates. Left panel shows endpoint performance at 25 and 50 °C, right panel shows melting curves and fluorescent images of the reaction wells. Bar graphs show mean values and individual replicates (black cross markers), melting curves show mean values (solid line) with 95% confidence intervals (shaded area), n = 2 independent samples for both cases. Due to the temperature dependence of Cyanine3 quantum yield, absolute fluorescence values differ between 25 °C and 50 °C. For clarity, images were linearly adjusted so that control wells containing Cyanine3 exhibit the same mean fluorescence. Pre-heating-cooling cycle (25-50-25 °C) was used in the experiment to ensure equilibrium at the starting point of the melting curve. Please note that some sequences are written from 3′ to 5′ for mismatch highlighting

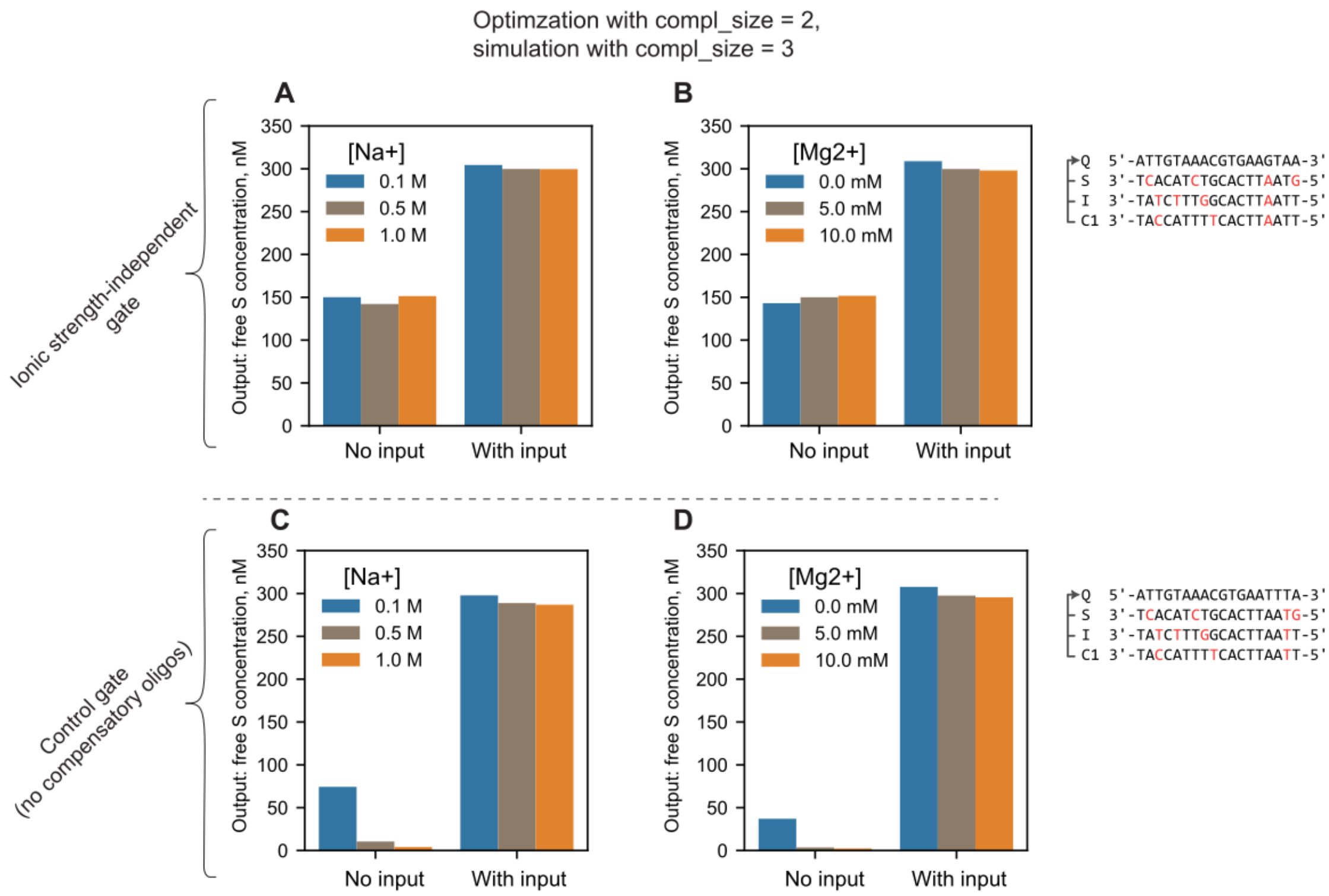

Supplementary Fig. 10. Performance of salt-independent and control YES logic gates, simulated in NUPACK while accounting for complexes with up to three strands (compl_size = 3). (A, C) show gate performance under Na^+ variation (Mg^{2+} concentration is zero), (B, D) show gate performance under Mg^{2+} variation (Na^+ concentration is 50 mM). Please note that some sequences are written from 3′ to 5′ for demonstrating mismatches.

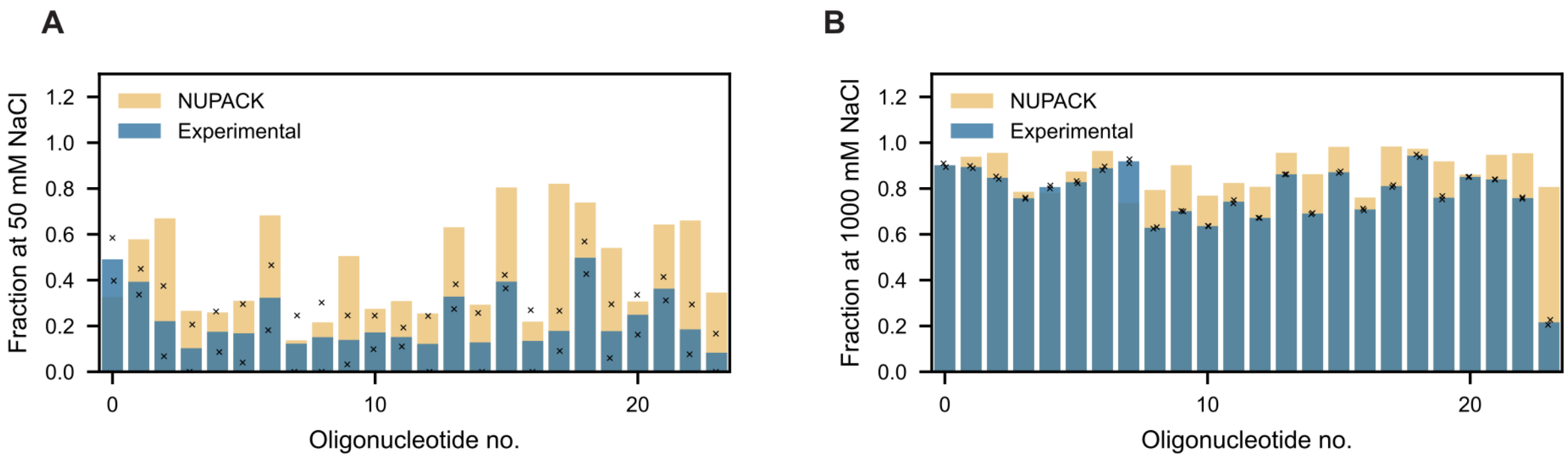

Supplementary Fig. 11. Accuracy of NUPACK affinity predictions at 50 and 1000 mM of Na^+. Data are presented as mean values and individual replicates (black cross markers), expressed as the concentration of the resulting duplex if both strands are mixed at 1 μM (n = 2 independent samples). Each bar represents a distinct oligonucleotide pair.

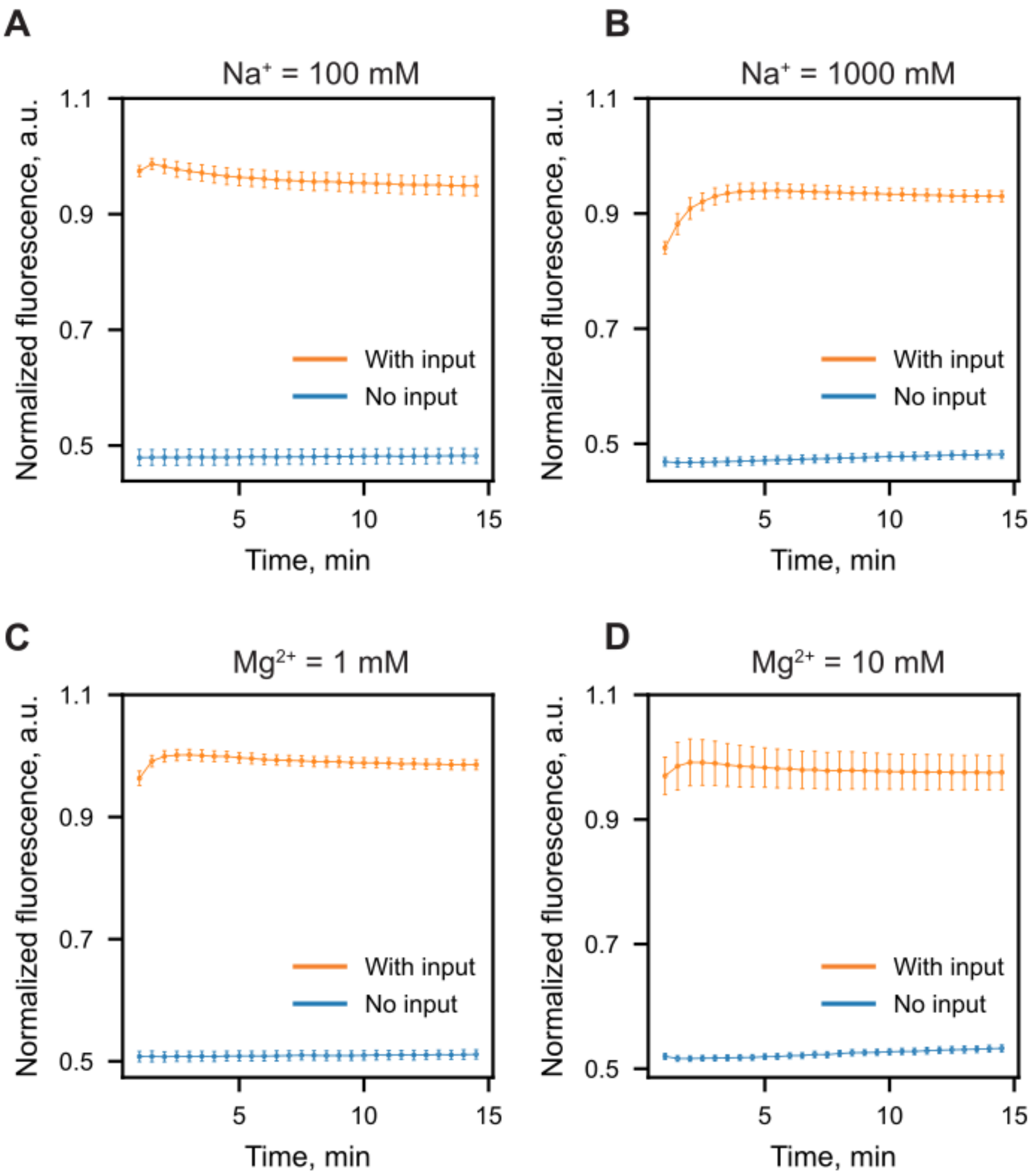

Supplementary Fig. 12. Kinetics of salt independent YES-gates from Fig. 5 following the addition of the input oligonucleotide. Other components (S, Q, and C) were pre-equilibrated for 1 hour. Data are presented in relative fluorescence units as mean ± s.d., n = 3 independent samples. Fluorescence is normalized to the signal of a sample containing only S at the same concentration. (A, B) show the gate performance optimized for robust operation under sodium variation, while (C, D) show the corresponding results for magnesium variation

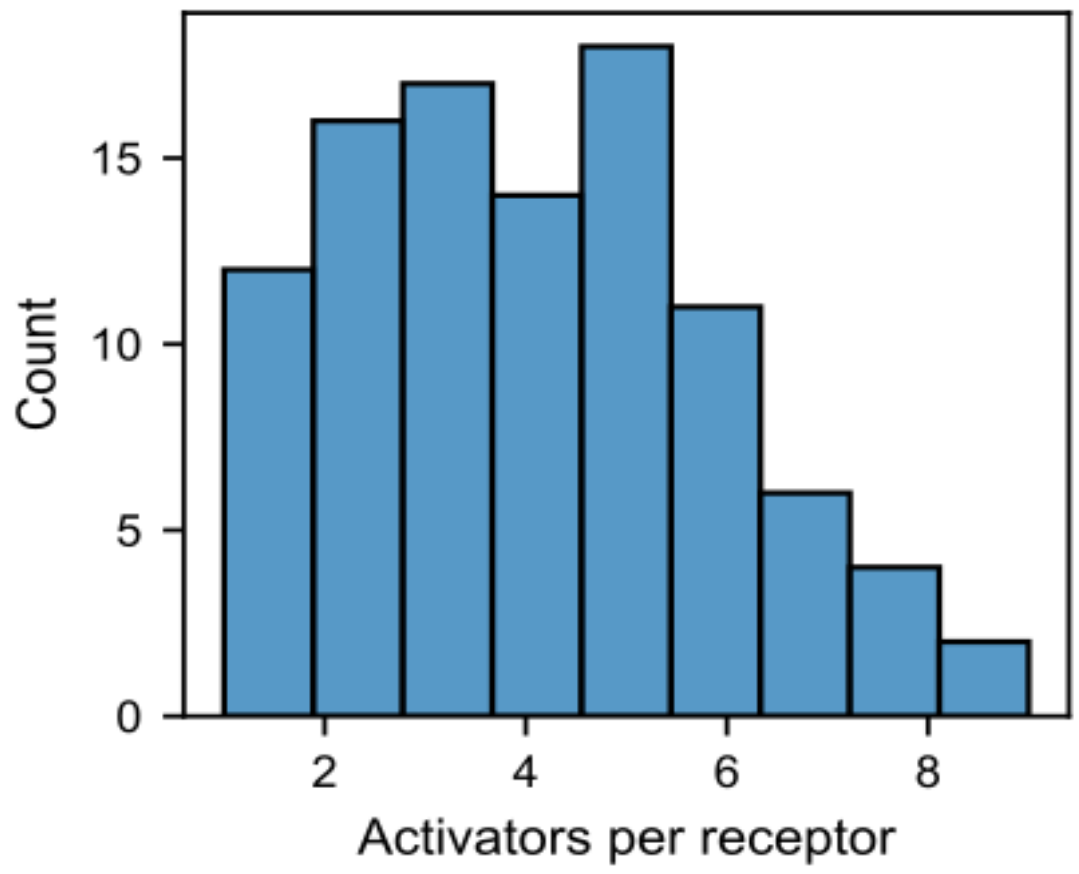

Supplementary Fig. 13. Distribution of activators per one receptor, related to **Fig. 6D**.

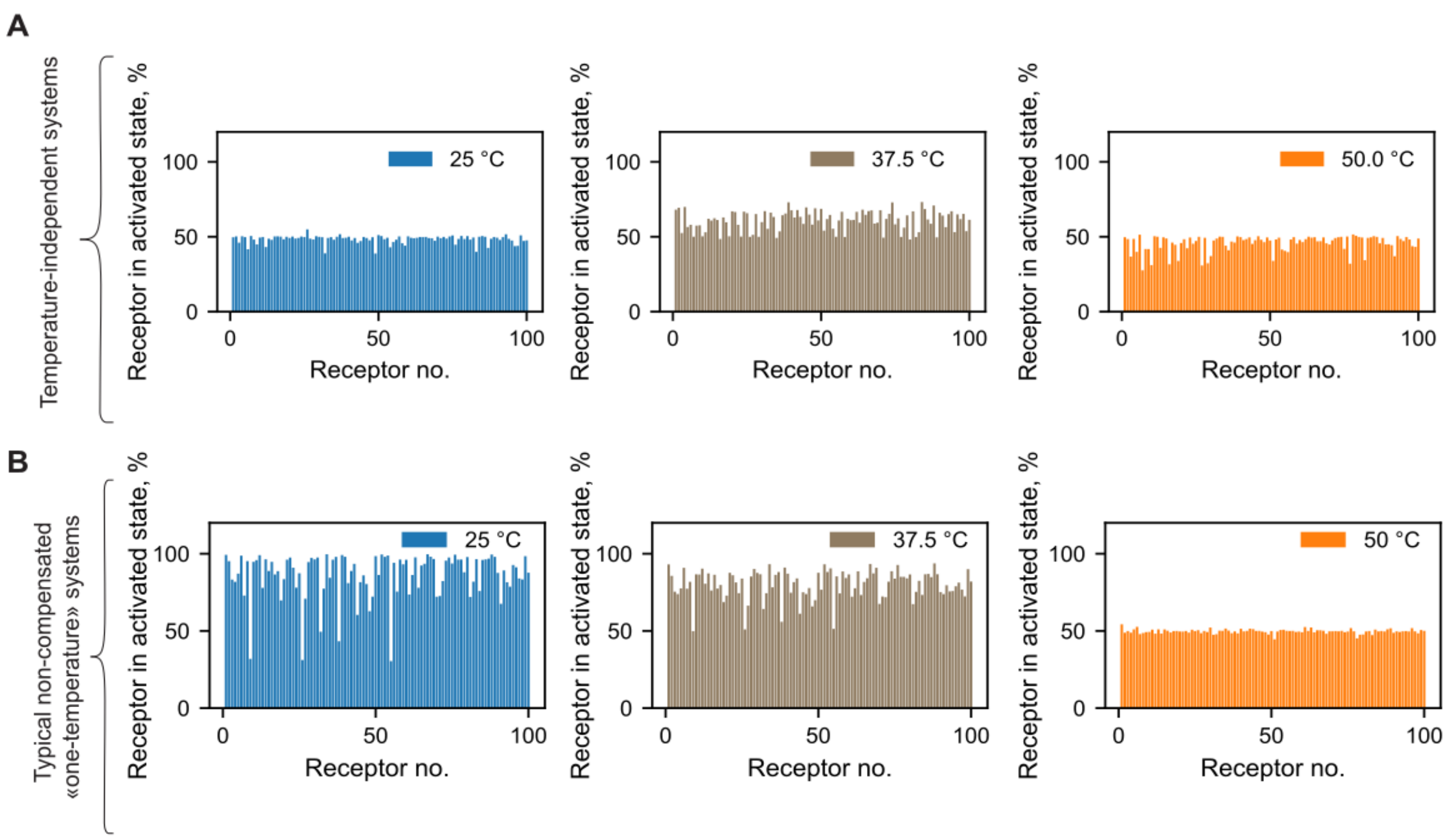

Supplementary Fig. 14. Performance of random receptor-activator network at intermediate temperatures. (A) Temperature-independent system, (B) typical system without compensation, optimized separately to have predefined activation levels at 50 °C only.

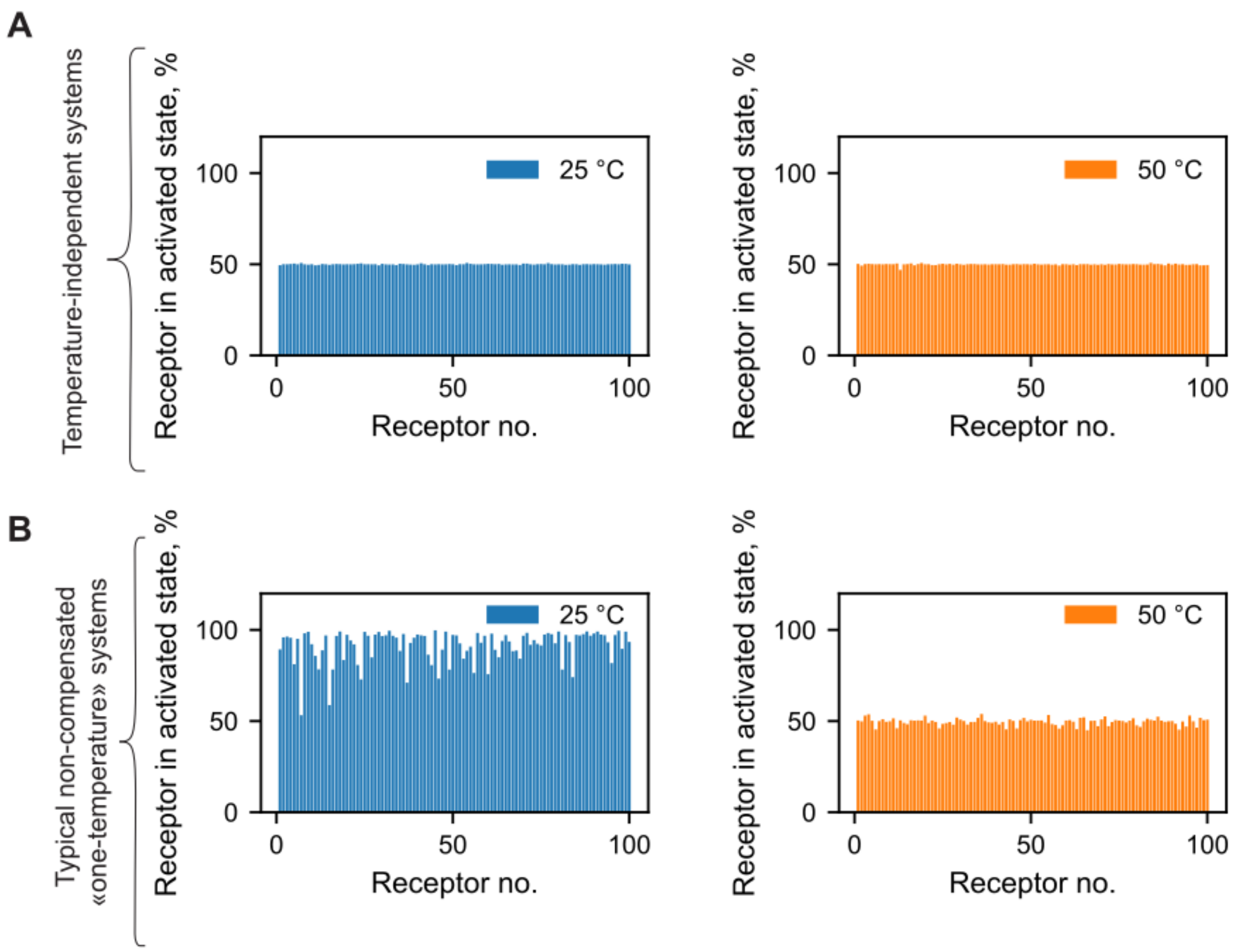

Supplementary Fig. 15. Performance of random receptor-activator network with activator misfolding. (A) Temperature-independent system, (B) typical system without compensation, optimized separately to have predefined activation levels at 50 °C only. A detailed description of the system is provided in **Supplementary Note 4**.

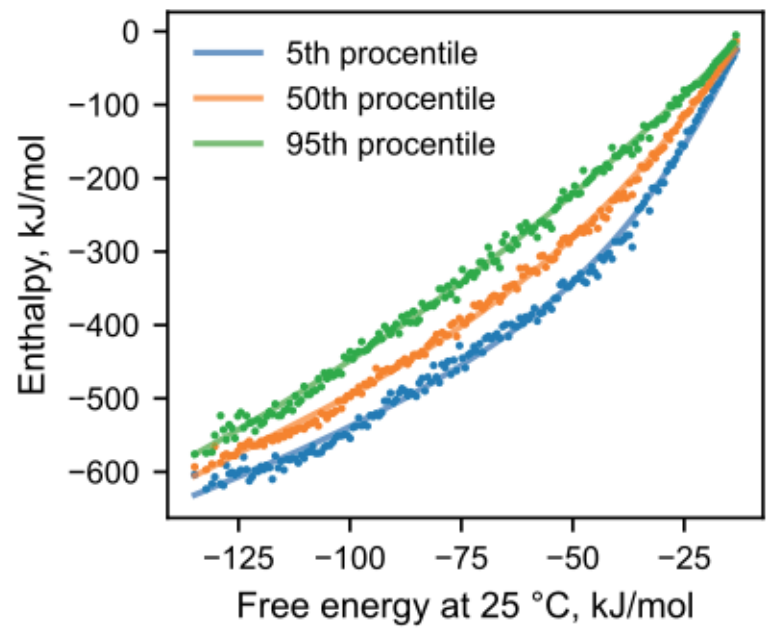

Supplementary Fig. 16. Scatter plot of enthalpy on Gibbs free energy at 25 °C and approximation with B-splines (solid lines) for 3 selected percentiles.

Supplementary Table 1. Coefficients of cubic B-splines used to approximate dependence of enthalpy on Gibbs free energy at 25 °C. t refers to root vectors, and c are coefficients.

p5			p10	p20	p30	p40	p50	p60	p70	p80	p90	p95
**t**	**c**	**t**	**c**	**c**	**c**	**c**	**c**	**c**	**c**	**c**	**c**	**c**
-134.84	-631.47	-134.84	-632.92	-622.6	-614.94	-609.81	-605.39	-600.88	-595.3	-590.12	-581.86	-576.43
-134.84	-579.53	-134.84	-493.06	-489.78	-488.64	-483.55	-478.72	-475.48	-471.23	-461.27	-449.22	-440.22
-134.84	-473.94	-134.84	-466.94	-436.4	-411.12	-390.78	-372.37	-352.05	-329.64	-306.62	-282.17	-262.73
-134.84	-268.59	-134.84	-32.2	-28.6	-26.41	-25.36	-24.06	-23.45	-22.94	-22.43	-19.61	-17.79
-72.17	-27.99	-13.38	0	0	0	0	0	0	0	0	0	0
-13.38	0	-13.38	0	0	0	0	0	0	0	0	0	0
-13.38	0	-13.38	0	0	0	0	0	0	0	0	0	0
-13.38	0	-13.38	0	0	0	0	0	0	0	0	0	0
-13.38	0											

Supplementary Note 1. Analysis of the feasibility of temperature compensation in the context of protein–protein interactions.

As previously noted, the existence of temperature compensation in networks of interacting oligonucleotides is enabled by the existence of “hard-to-melt” and “easy-to-melt” pairs. Importantly, in this context, nucleic acids serve merely as a convenient model system that allows straightforward evaluation of their thermodynamic properties. More generally, any biomolecules that exhibit distinct temperature-dependent behaviors are likewise capable of demonstrating analogous compensatory effects.

To demonstrate this, we consider the case of protein–protein interactions. For the analysis, the Stites (1997) dataset[1] was selected. Because, for such interactions, the enthalpy typically exhibits a strong temperature dependence[1] (i.e., the change in heat capacity ΔC_p is significantly different from zero), only data for which ΔC_p was directly measured were included in the analysis. Otherwise, the use of enthalpy and entropy values obtained at a single temperature (e.g., from isothermal titration calorimetry performed at a single temperature point as is the case in most other datasets) may be misleading when analyzing the temperature dependence of the interaction.

The distribution of dissociation constants at 25 and 50 °C for protein–protein interactions is shown in **Supplementary Fig. 1A**. Notably, the same characteristic feature observed for the analogous distribution of DNA–DNA interactions (**Fig. 1A**) is evident here: for a given K_d at 25 °C, there exist protein–protein pairs with substantially different extrapolated K_d values at 50 °C.

Moreover, a similar pattern can be observed even in the case where one of the proteins remains unchanged while single-point mutations are introduced into the other. For instance, an analogous distribution is shown in **Supplementary Fig. 1B** for a mutational scan[2] of an anti-HER2 antibody reported in Kelley (1993). Notably, the VH-F100A and VL-Y92A antibody variants exhibit nearly identical Kd values at 25 °C (1.7 and 2.4 nM, respectively), yet display markedly different extrapolated K_d change at 50 °C (9.7- and 4.3-fold change, respectively), corresponding to a 2.3-fold difference. Besides that, certain antibodies are known to exhibit substantially greater temperature sensitivity. For example, increasing the temperature by only 2 °C (from 40 to 42 °C) may change the affinities of some malaria-associated antibodies[3] by 11.5-fold.

Supplementary Note 2. Construction of temperature-compensated systems without parameter optimization across the entire temperature range

In this section, we demonstrate that the topology alone of signal-processing systems with compensatory species is sufficient to achieve partial temperature compensation.

Let us consider the topology of a YES gate in its two variants: the first one without compensation (three oligos I, Q, S), and the second with compensation (four oligos I, Q, S, C). For the second one to be conceptually different from the first one, the concentration of C and the affinity of Q–C should not be zero or negligibly small, so let's fix these values to some reasonable values, for example, concentration of the compensator C to 5 μM and the dissociation constant of Q–C at 25°C to 1.7 nM (−50 kJ/mol). Now, consider random gates of these two topologies that have the same level of free S at 25 °C, for example, 100 and 200 nM in the absence and in the presence of input, respectively. Gate performance is typically defined as the difference between outputs in the True and False states (i.e., with and without input), which in this case is 100 nM (**Supplementary Fig. 3A, D**). How would the True-False difference behave at 50 °C for non-compensated and compensated groups of gates?

We have generated 100 distinct parameter sets for both groups of gates. **Supplementary Fig. 3B,C** presents the distributions of the True-False differences for these groups of gates at 25 and 50 °C. It is evident that the True-False difference at 50 °C is substantially higher in the compensated system, whereas for the majority of control systems it is close to zero; that is, the states with and without input at 50 °C are nearly indistinguishable. Similarly, we performed the same analysis for the NOT gate. The results are presented in **Supplementary Fig. 3E,F**, where the same pattern is observed. Thus, an appropriate system topology can ensure temperature stabilization even if its parameters are not optimized for operation at temperatures other than the reference one.

Supplementary Note 3. Calculation of the hybridization temperature dependence using NUPACK.

The principles underlying NUPACK are described in detail in the relevant publications[4]. Briefly, NUPACK considers the Boltzmann ensemble of all possible states of participating oligonucleotides. For each possible state, NUPACK calculates the Gibbs free energy, using the nearest-neighbor model with various corrections, such as loops, stacking interactions, dangling ends, and others. In this model, enthalpy and entropy of each individual state are assumed to be temperature-independent. The resulting information is then aggregated over all states by computing the partition function for each complex.

Let us consider a simple system consisting of two strands, s_1 and s_2. We restrict the analysis to the complexes (s_1), (s_2), and ($s_1 + s_2$), as the concentrations of all other complexes are negligible by design.

If:

1. Single-stranded species exist mostly as free strands (i.e., structures with non-zero free energy have negligible low concentrations);
2. The most favorable duplex state is significantly lower in free energy than all alternative states;

then the hybridization reaction can be described by only two parameters, (for example, enthalpy and entropy) and dependence of reaction Gibbs free energy on temperature will be linear.

To test this assumption for the oligonucleotides used in this study, we analyzed the same dataset as in Fig. 1A. To exclude extreme cases, we considered oligonucleotide pairs with Gibbs free energy at 25 °C ranging from −30 to −80 kJ/mol. For these 4,684 oligonucleotides, we computed the dependence of ΔG(T) over the temperature range from 25 to 50 °C.

We then estimated apparent enthalpy and entropy from 25 and 50 °C temperature endpoints using simple linear approximation and evaluated the maximum error across all intermediate temperatures arising from predicting ΔG in this manner. The median error was 0.31 kJ/mol, while the maximum error reached 2.6 kJ/mol (0.7% and 10% for the relative error). This supports the validity of the approximate description of the hybridization reaction using only two parameters. In addition, it should be noted that this approximation serves only as an intermediate step to simplify the identification of nucleic acid sequences and, therefore, does not require particularly high accuracy.

Supplementary Note 4. Temperature compensation in receptor-activator networks with monomolecular temperature-dependent processes

Consider a receptor–activator system analogous to that shown in Fig. 6D. In addition, let us introduce into this system a process of reversible misfolding.

$$A \leftrightarrows A_m$$

with equilibrium constant

$$q_i = \frac{m_i^{eq}}{c_i^{eq}}$$

Here c_i^{eq} and m_i^{eq} denote the equilibrium concentrations of the native and misfolded forms of the i-th molecule, respectively. Let K_{ij} denote the affinity constants for dimerization, c_i^{total} the total concentrations, and c_{ij}^{eq} the equilibrium concentrations of the dimers. The i-th mass-balance equation can then be written as:

$$c_i^{total} = c_i^{eq} + m_i^{eq} + \sum_{i \neq j} c_{ij}^{eq}$$

Substituting the definitions of the equilibrium constants:

$$c_i^{total} = c_i^{eq} + q_i c_i^{eq} + \sum_{i \neq j} K_{ij} c_i^{eq} c_j^{eq}$$

Solving for c_i^{eq}:

$$c_i^{eq} = \frac{c_i^{total}}{1 + q_i + \sum_{i \neq j} K_{ij} c_j^{eq}}$$

Thus, we obtain a fixed-point map that differs from that in the work of van Dorp[5] by the presence of q_i in the denominator and can be reduced to a canonical form by rescaling the concentrations and the dimerization constants by a factor of $1 + q_i$. Accordingly, the equilibrium concentrations are determined in the same manner as for all other systems considered in the paper, except for this modification.

To specify particular misfolding constants, we proceed as follows. For each molecule in the system, we define the fraction f_i of the misfolded form at 25 and 50°C, which is straightforwardly related to q_i:

$$q_i = \frac{f_i}{1 - f_i}$$

If needed, the values of the constants at intermediate temperatures are calculated using van 't Hoff interpolation. For definiteness, we assume $f_i^{25°C} = 0.001$, i.e., almost all molecules are in the native state at 25 °C, and vary only $f_i^{50°C}$. The network parameters are specified in the same way as for the network in Fig. 6D, except that each activator is assigned a random value of $f_i^{50°C}$ in the range from 0.2 to 0.9. These values remain fixed during the optimization process. **Supplementary Fig. 15** compares the performance of the optimized and control systems: the optimized system shows nearly identical activation at both temperatures, whereas the control system exhibits significant discrepancies.

Supplementary Methods

Parameters in .json files

Please note that the affinities reported here are rounded to two significant digits due to space limitations. For programmatic access and exact reproducibility, we recommend using the .json files in Source Data.

We use a .json format that can be directly loaded with the NetworkX Python library. Each system is specified as a list of nodes and edges.

Node parameters:

- "Seq" – sequence from 5′ to 3′. If no sequence is provided, the system was simulated only at the level of affinity matrices and concentrations.
- "Conc" – species concentration in nM.
- "lb" and "ub" – lower and upper bounds of concentration (nM), used during PyBADS optimization.
- "id" – species name.
- "input" and "output" – indicate whether a node is treated as an input or output, respectively.

Edge parameters:

- "source" and "target" specify the "id" if the species that the edge describes.
- "G25" – Gibbs free energy (kJ/mol) at 25 °C, as optimized by PyBADS. Note that this value does not correspond to the energy predicted by NUPACK.
- "lb" and "ub" – lower and upper bounds of "G25" (kJ/mol), used during PyBADS optimization.
- "spl" – "easy-to-melt" level.

Other parameters are derived from these.

For receptor–activator networks, node parameters "activator" and "receptor" are used instead of "input" and "output". For networks with misfolding, "f25" and "f50" denote the fraction of misfolded species at 25 °C and 50 °C, respectively.

YES-gate

Optimization parameters for determining concentrations and binding affinities.

		Concentration lower bound, nM	Concentration upper bound, nM	Concentration start value, nM	
**Nodes**	**I**	100	5000	1000	
	**Q**	100	5000	1000	
	**S**	200	300	220	
	**M**	100	5000	1000	
		**Gibbs energy at 25 °C lower bound, kJ/mol**	**Gibbs energy at 25 °C upper bound, kJ/mol**	**Gibbs energy at 25 °C start value, kJ/mol**	**Easy-to-melt level**
**Edges**	**I-Q**	-60	-30	-50	p90
	**Q-S**	-60	-30	-50	p90
	**Q-M**	-60	-30	-50	p5

The target values for the free concentration of the output oligonucleotide were 100 nM and 200 nM in the absence and presence of the input, respectively.

Optimization parameters for sequence finding: *hightemp_ka_atten* = 10^3, *hairpin_atten = 2*, *early stop condition = 0.3 kJ/mol.*

Optimized sequences and concentrations

ID	Sequence, 5'-3'	Concentration, µM	Hairpin formation energy at 25 °C, kcal/mol
I	TATTTTATGTTTTACCTGAA	4.99	0.00
Q	GTGAGGGAAAACATAAACGA	0.70	0.00
S	TCGTTTATGTTCTTATTATC	0.22	0.00
C	CCGTTTTAGATTTTCCTCAC	1.50	0.00

Mutual affinity of the oligos. Approximate dissociation constant K_d at 25 °C in M, as predicted by NUPACK:

	I	Q	S	M
**I**	3.43E-03	7.35E-11	2.02E-03	2.87E-03
**Q**	7.35E-11	2.16E-04	9.68E-11	1.88E-10
**S**	2.02E-03	9.68E-11	1.31E-03	1.35E-03
**M**	2.87E-03	1.88E-10	1.35E-03	1.53E-03

Mutual affinity of the oligos. Approximate dissociation constant K_d at 50 °C in M, as predicted by NUPACK:

	I	Q	S	C
**I**	6.15E-03	3.52E-07	5.82E-03	5.99E-03
**Q**	3.52E-07	2.86E-03	4.46E-07	2.07E-05
**S**	5.82E-03	4.46E-07	5.96E-03	5.99E-03
**C**	5.99E-03	2.07E-05	5.99E-03	7.43E-03

YES-gate (compl_size = 3 optimization)

Optimized sequences and concentrations

ID	Sequence, 5'-3'	Concentration, µM	Hairpin formation energy at 25 °C, kcal/mol
I	ACTTTTATGTTTTACCTGAA	4,65	0,00
Q	GTGAGGGAAAACATAAACGA	0,78	0,00
S	TCGTTTATGTTCTTATTATC	0,22	0,00
M	TCGTTTTAGATTTTCCCAAC	1,71	0,00

Mutual affinity of the oligos. Approximate dissociation constant K_d at 25 °C in M, as predicted by NUPACK:

	I	Q	S	M
**I**	3,8E-03	2,1E-11	2,6E-03	3,1E-03
**Q**	2,1E-11	2,2E-04	9,7E-11	8,5E-11
**S**	2,6E-03	9,7E-11	1,3E-03	1,3E-03
**M**	3,1E-03	8,5E-11	1,3E-03	2,1E-03

Mutual affinity of the oligos. Approximate dissociation constant K_d at 50 °C in M, as predicted by NUPACK:

	I	Q	S	M
**I**	6,6E-03	2,2E-07	6,3E-03	5,3E-03
**Q**	2,2E-07	2,9E-03	4,5E-07	6,1E-06
**S**	6,3E-03	4,5E-07	6,0E-03	4,7E-03
**M**	5,3E-03	6,1E-06	4,7E-03	5,9E-03

NOT-gate

Optimization parameters for determining concentrations and binding affinities.

		Concentration lower bound, nM	Concentration upper bound, nM	Concentration start value, nM	
**Nodes**	**I**	100	7000	1000	
	**M**	100	7000	1000	
	**Q**	100	7000	1000	
	**S**	100	300	300	
	**Q1**	100	7000	1000	
	**I1**	100	7000	1000	
		**Gibbs energy at 25 °C lower bound, kJ/mol**	**Gibbs energy at 25 °C upper bound, kJ/mol**	**Gibbs energy at 25 °C start value, kJ/mol**	**Easy-to-melt level**
**Edges**	**I-M**	-60	-30	-60	p90
	**M-Q**	-60	-30	-60	p90
	**Q-S**	-60	-30	-60	p90
	**Q1-Q**	-50	-30	-60	p20
	**I1-I**	-50	-30	-60	p20

The target values for the free concentration of the output oligonucleotide were 200 nM and 100 nM in the absence and presence of the input, respectively.

Optimization parameters for sequence finding: *hightemp_ka_atten* = 10^3, *lowtemp_ka_atten* = 10^4, *hairpin_atten = 5, early stop condition = 1 kJ/mol.*

Optimized sequences and concentrations:

ID	Sequence, 5'-3'	Concentration, µM	Hairpin formation energy at 25 °C, kcal/mol
I	UCCAUACUUAUUUAAAGUAC	5.55	-3.24
M	CUUCUUUAAUUUAGUAUGGA	5.48	-0.61
Q	UCUAAACUAAAUUAGAAAAG	4.05	-3.64
S	GUUUUCUCAUUUAGUUUACA	0.96	0.00
Q1	CUUUUCUUAUUCUGUUUAGU	4.17	0.00
I1	GUACUUUCAAUAAAUAUCGG	5.88	0.00

Mutual affinity of the oligos. Approximate dissociation constant K_d at 25 °C in M, as predicted by NUPACK:

	I	M	Q	S	Q1	I1
I	2.5E-02	2.5E-11	3.8E+00	3.3E-01	5.8E-01	7.1E-09
M	2.5E-11	6.4E-03	4.4E-10	2.6E-04	5.8E-04	5.6E-04
Q	3.8E+00	4.4E-10	2.4E-02	3.1E-11	1.5E-10	8.9E-02
S	3.3E-01	2.6E-04	3.1E-11	1.6E-03	1.7E-02	1.7E-03
Q1	5.8E-01	5.8E-04	1.5E-10	1.7E-02	1.3E-03	7.0E-04
I1	7.1E-09	5.6E-04	8.9E-02	1.7E-03	7.0E-04	1.2E-04

Mutual affinity of the oligos. Approximate dissociation constant K_d at 50 °C in M, as predicted by NUPACK:

	I	M	Q	S	Q1	I1
I	1.1E-02	1.7E-07	2.5E-01	9.2E-02	1.3E-01	1.1E-04
M	1.7E-07	1.4E-02	1.1E-06	7.5E-03	1.2E-02	4.7E-03
Q	2.5E-01	1.1E-06	8.2E-03	1.6E-07	1.1E-05	5.5E-02
S	9.2E-02	7.5E-03	1.6E-07	4.8E-02	4.3E-02	1.7E-02
Q1	1.3E-01	1.2E-02	1.1E-05	4.3E-02	5.1E-02	1.0E-02
I1	1.1E-04	4.7E-03	5.5E-02	1.7E-02	1.0E-02	8.9E-03

NOT-gate (compl_size = 3 optimization)

Optimized sequences and concentrations:

ID	Sequence, 5'-3'	Concentration, µM	Hairpin formation energy at 25 °C, kcal/mol
I	UCCAUACUUAUUUAAAGUAC	6.72	-3.24
M	CUUCUUUAAUUUAGUAUGGA	6.98	-0.61
Q	UCUAAGCUAAAUUAGAAAAG	3.57	-4.70
S	GUUUUCUCAUUUAGUUUACU	0.72	0.00
Q1	CUUUUCUUAUUCUGUUUAGU	4.36	0.00
I1	GAACUUUAAAUAACUAUCGG	6.90	0.00

The target values for the free concentration of the output oligonucleotide were 200 nM and 100 nM in the absence and presence of the input, respectively.

Mutual affinity of the oligos. Approximate dissociation constant K_d at 25 °C in M, as predicted by NUPACK:

	I	M	Q	S	Q1	I1
I	2.5E-02	2.5E-11	5.4E+00	2.9E-01	5.8E-01	7.4E-10
M	2.5E-11	6.4E-03	1.4E-09	2.4E-04	5.8E-04	1.7E-03
Q	5.4E+00	1.4E-09	8.6E-03	1.8E-10	9.7E-10	7.9E-02
S	2.9E-01	2.4E-04	1.8E-10	7.7E-04	1.9E-02	8.2E-05
Q 1	5.8E-01	5.8E-04	9.7E-10	1.9E-02	1.3E-03	1.2E-04
I1	7.4E-10	1.7E-03	7.9E-02	8.2E-05	1.2E-04	1.7E-03

Mutual affinity of the oligos. Approximate dissociation constant K_d at 50 °C in M, as predicted by NUPACK:

	I	M	Q	S	Q1	I1
I	1.1E-02	1.7E-07	3.6E-01	7.8E-02	1.3E-01	8.4E-06
M	1.7E-07	1.4E-02	1.6E-06	6.9E-03	1.2E-02	6.0E-03
Q	3.6E-01	1.6E-06	8.6E-03	2.8E-07	1.9E-05	1.2E-01
S	7.8E-02	6.9E-03	2.8E-07	3.5E-02	5.1E-02	2.8E-03
Q1	1.3E-01	1.2E-02	1.9E-05	5.1E-02	5.1E-02	3.6E-03
I1	8.4E-06	6.0E-03	1.2E-01	2.8E-03	3.6E-03	1.1E-02

AND-gate

Optimization parameters for determining concentrations and binding affinities.

		Concentration lower bound, nM	Concentration upper bound, nM	Concentration start value, nM	
**Nodes**	**I1**	100	5000	1000	
	**I2**	100	5000	1000	
	**Q1**	100	5000	1000	
	**Q2**	100	5000	1000	
	**S**	100	400	400	
	**C1**	100	5000	1000	
	**C2**	100	5000	1000	
	**C3**	100	5000	1000	
		**Gibbs energy at 25 °C lower bound, kJ/mol**	**Gibbs energy at 25 °C upper bound, kJ/mol**	**Gibbs energy at 25 °C start value, kJ/mol**	**Easy-to-melt level**
**Edges**	**I1-Q1**	-60	-30	-60	p95
	**I2-Q2**	-60	-30	-60	p95
	**Q1-S**	-60	-30	-60	p95
	**Q2-S**	-60	-30	-60	p95
	**C1-Q1**	-60	-30	-60	p40
	**C2-Q2**	-60	-30	-60	p40
	**C3-S**	-60	-30	-60	p40

The target values for the free concentration of the output oligonucleotide were 50 nM (when both inputs were absent), 100 nM (when only one input was present), and 250 nM (when both inputs were present).

Optimization parameters for sequence finding: *hightemp_ka_atten = 10^3, lowtemp_ka_atten = 10^4, hairpin_atten = 3, early stop condition = 1 kJ/mol.*

Optimized sequences and concentrations

ID	Sequence, 5'-3'	Concentration, µM	Hairpin formation energy at 25 °C, kcal/mol
I1	GGGTGTGGTAACGAAGACCG	5.00	-1.40
I2	CTGTGAGGATACGTACAATG	5.00	-1.68
Q1	CGGTCTTCGTTTTTTCACTC	0.74	-0.95
Q2	CATTGTACGTATCTTTACTG	0.74	-1.22
S	GAGTAAAGATACGAAGACCG	0.31	-1.40
C1	GGGTGACTAATCGAAGACCG	0.74	-1.58
C2	TAGTAATGATGCGTACAATA	0.73	-0.96
C3	GGGGTTTTGTTTCTTTGCTC	0.31	0.00

Mutual affinity of the oligos. Approximate dissociation constant K_d at 25 °C in M, as predicted by NUPACK:

	I1	I2	Q1	Q2	S	C1	C2	C3
I1	1.79E-03	6.37E-03	3.59E-11	3.32E-03	8.97E-04	1.81E-03	5.28E-03	1.69E-03
I2	6.37E-03	3.32E-04	3.15E-04	3.11E-11	2.87E-03	9.63E-03	1.31E-03	1.01E-03
Q1	3.59E-11	3.15E-04	9.73E-03	2.83E-03	4.86E-11	3.21E-11	1.36E-03	4.32E-03
Q2	3.32E-03	3.11E-11	2.83E-03	1.44E-04	4.49E-11	2.04E-03	3.50E-11	8.10E-03
S	8.97E-04	2.87E-03	4.86E-11	4.49E-11	5.06E-03	1.39E-03	1.79E-03	5.64E-06
C1	1.81E-03	9.63E-03	3.21E-11	2.04E-03	1.39E-03	1.63E-03	3.82E-03	1.09E-03
C2	5.28E-03	1.31E-03	1.36E-03	3.50E-11	1.79E-03	3.82E-03	1.08E-03	8.29E-04
C3	1.69E-03	1.01E-03	4.32E-03	8.10E-03	5.64E-06	1.09E-03	8.29E-04	1.19E-03

Mutual affinity of the oligos. Approximate dissociation constant K_d at 50 °C in M, as predicted by NUPACK:

	I1	I2	Q1	Q2	S	C1	C2	C3
I1	3.93E-03	6.60E-03	1.67E-07	3.91E-03	4.49E-03	4.67E-03	7.45E-03	3.54E-03
I2	6.60E-03	8.92E-04	2.60E-03	1.58E-07	3.70E-03	9.60E-03	3.48E-03	3.10E-03
Q1	1.67E-07	2.60E-03	1.02E-02	4.82E-03	2.22E-07	1.18E-06	4.16E-03	9.33E-03
Q2	3.91E-03	1.58E-07	4.82E-03	6.18E-04	1.76E-07	5.34E-03	1.66E-06	9.09E-03
S	4.49E-03	3.70E-03	2.22E-07	1.76E-07	8.08E-03	3.60E-03	3.62E-03	6.59E-04
C1	4.67E-03	9.60E-03	1.18E-06	5.34E-03	3.60E-03	4.72E-03	7.04E-03	3.49E-03
C2	7.45E-03	3.48E-03	4.16E-03	1.66E-06	3.62E-03	7.04E-03	5.00E-03	3.32E-03
C3	3.54E-03	3.10E-03	9.33E-03	9.09E-03	6.59E-04	3.49E-03	3.32E-03	1.06E-02

AND-gate (compl_size = 3 optimization)

Optimized sequences and concentrations

ID	Sequence, 5'-3'	Concentration, µM	Hairpin formation energy at 25 °C, kcal/mol
I1	GGGTGTAGTAACGAAGACCG	5,00	-1,40
I2	CTGTGAGGATACGTACAATG	5,00	-1,68
Q1	CGGTCTTCGTTTTTTCACTC	1,05	-0,95
Q2	CATTGTACGTATCTTTACTG	0,58	-1,22
S	GAGTAAAGATACGAAGACCG	0,32	-1,40
C1	GGGTGACTAATCGAAGACCG	1,21	-1,58
C2	TAGTAATGATGCGTACAATA	0,51	-0,96
C3	CGGGGTTAATTTCTTAGCCC	1,76	-2,67

Mutual affinity of the oligos. Approximate dissociation constant K_d at 25 °C in M, as predicted by NUPACK:

	I1	I2	Q1	Q2	S	C1	C2	C3
**I1**	1,7E-03	3,1E-03	1,5E-11	4,1E-04	1,1E-03	1,1E-03	2,9E-03	2,9E-02
**I2**	3,1E-03	3,3E-04	3,2E-04	3,1E-11	2,9E-03	9,6E-03	1,3E-03	1,5E-01
**Q1**	1,5E-11	3,2E-04	9,7E-03	2,8E-03	4,9E-11	3,2E-11	1,4E-03	6,2E-02
**Q2**	4,1E-04	3,1E-11	2,8E-03	1,4E-04	4,5E-11	2,0E-03	3,5E-11	7,4E-02
**S**	1,1E-03	2,9E-03	4,9E-11	4,5E-11	5,1E-03	1,4E-03	1,8E-03	1,4E-02
**C1**	1,1E-03	9,6E-03	3,2E-11	2,0E-03	1,4E-03	1,6E-03	3,8E-03	2,0E-02
**C2**	2,9E-03	1,3E-03	1,4E-03	3,5E-11	1,8E-03	3,8E-03	1,1E-03	5,3E-02
**C3**	2,9E-02	1,5E-01	6,2E-02	7,4E-02	1,4E-02	2,0E-02	5,3E-02	2,9E-02

Mutual affinity of the oligos. Approximate dissociation constant K_d at 50 °C in M, as predicted by NUPACK:

	I1	I2	Q1	Q2	S	C1	C2	C3
**I1**	4,2E-03	4,8E-03	9,9E-08	1,8E-03	4,7E-03	3,5E-03	5,3E-03	1,2E-02
**I2**	4,8E-03	8,9E-04	2,6E-03	1,6E-07	3,7E-03	9,6E-03	3,5E-03	2,9E-02
**Q1**	9,9E-08	2,6E-03	1,0E-02	4,8E-03	2,2E-07	1,2E-06	4,2E-03	2,6E-02
**Q2**	1,8E-03	1,6E-07	4,8E-03	6,2E-04	1,8E-07	5,3E-03	1,7E-06	3,1E-02
**S**	4,7E-03	3,7E-03	2,2E-07	1,8E-07	8,1E-03	3,6E-03	3,6E-03	8,1E-03
**C1**	3,5E-03	9,6E-03	1,2E-06	5,3E-03	3,6E-03	4,7E-03	7,0E-03	1,2E-02
**C2**	5,3E-03	3,5E-03	4,2E-03	1,7E-06	3,6E-03	7,0E-03	5,0E-03	2,9E-02
**C3**	1,2E-02	2,9E-02	2,6E-02	3,1E-02	8,1E-03	1,2E-02	2,9E-02	1,3E-02

0.1-50 °C control YES-gate (one compensatory oligo, Fig. 2D)

Optimization parameters for determining concentrations and binding affinities.

		**Concentration lower bound, nM**	**Concentration upper bound, nM**	**Concentration start value, nM**	
**Nodes**	**I**	100	5000	1000	
	**Q**	100	5000	1000	
	**S**	100	200	200	
	**M**	100	5000	1000	
		**Gibbs energy at 25 °C lower bound, kJ/mol**	**Gibbs energy at 25 °C upper bound, kJ/mol**	**Gibbs energy at 25 °C start value, kJ/mol**	**Easy-to-melt level**
**Edges**	**I-Q**	-60	-30	-60	p95
	**Q-S**	-60	-30	-60	p95
	**Q-M**	-60	-30	-60	p70

The target values for the free concentration of the output oligonucleotide were 50 nM and 100 nM in the absence and presence of the input, respectively.

Optimization parameters for sequence finding: *hightemp_ka_atten* = 10^3, *lowtemp_ka_atten* = 10^4, *hairpin_atten = 5, early stop condition = 0.5 kJ/mol.*

Optimized sequences and concentrations

**ID**	**Sequence, 5'-3'**	**Concentration, μM**	**Hairpin formation energy at 25 °C, kcal/mol**
I	TTCCACTCCTGACCCCCAAG	2.83	-0.48
Q	TGATGTAGATAGGAGTGGAA	0.50	0.00
S	TTCCACTCCTGCCAGCCGAC	0.12	0.00
C1	TTACACTCCTAATTTTATCA	1.50	0.00

Mutual affinity of the oligos. Approximate dissociation constant K_d at 25 °C in M, as predicted by NUPACK:

	**I**	**Q**	**S**	**C1**
**I**	8.13E-03	7.82E-11	3.40E-03	6.82E-03
**Q**	7.82E-11	3.70E-03	6.38E-11	6.64E-10
**S**	3.40E-03	6.38E-11	5.78E-04	5.46E-03
**C1**	6.82E-03	6.64E-10	5.46E-03	1.48E-03

Mutual affinity of the oligos. Approximate dissociation constant K_d at 50 °C in M, as predicted by NUPACK:

	**I**	**Q**	**S**	**C1**
**I**	1.00E-02	1.96E-07	7.26E-03	1.03E-02
**Q**	1.96E-07	7.64E-03	1.82E-07	1.89E-06
**S**	7.26E-03	1.82E-07	3.16E-03	1.10E-02
**C1**	1.03E-02	1.89E-06	1.10E-02	5.53E-03

0.1-50 °C YES-gate (with compensatory cascade, Fig. 2E)

Optimization parameters for determining concentrations and binding affinities.

		Concentration lower bound, nM	Concentration upper bound, nM	Concentration start value, nM	
**Nodes**	**I**	100	5000	1000	
	**Q**	100	2500	1000	
	**S**	100	200	120	
	**M1**	100	5000	1000	
	**M2**	100	5000	1000	
	**M3**	100	5000	1000	
		**Gibbs energy at 25 °C lower bound, kJ/mol**	**Gibbs energy at 25 °C upper bound, kJ/mol**	**Gibbs energy at 25 °C start value, kJ/mol**	**Easy-to-melt level**
**Edges**	**I-Q**	-60	-30	-60	p95
	**Q-S**	-60	-30	-60	p95
	**Q-M1**	-60	-30	-60	p80
	**M2-M1**	-60	-30	-60	p60
	**M3-M2**	-60	-30	-60	p40

The target values for the free concentration of the output oligonucleotide were 50 nM and 100 nM in the absence and presence of the input, respectively.

Optimization parameters for sequence finding: *hightemp_ka_atten = 10^3, lowtemp_ka_atten = 10^4, hairpin_atten = 3, early stop condition = 1 kJ/mol.*

Optimized sequences and concentrations

ID	Sequence, 5'-3'	Concentration, µM	Hairpin formation energy at 25 °C, kcal/mol
I	TAATAAGGAGGAAGCTAAAC	5.00	-0.72
Q	ACCTACCTTCCTCCTTCCTA	0.97	0.00
S	GATTAAGGAGGCAGGTGTCG	0.12	0.00
C1	GGCGAGGGAGGCAGGTAGAT	2.93	-1.34
C2	ATCTATCCCATTCCCTCGTC	2.80	0.00
C3	GACGGGGTCATGGGATTGGT	5.00	-1.03

Mutual affinity of the oligos. Approximate dissociation constant K_d at 25 °C in M, as predicted by NUPACK:

	I	Q	S	C1	C2	C3
I	6.53E-04	3.76E-11	1.13E-03	3.77E-03	2.95E-05	2.40E-03
Q	3.76E-11	3.00E-02	2.68E-11	9.10E-11	6.65E-03	7.67E-06
S	1.13E-03	2.68E-11	8.69E-04	1.92E-03	1.89E-05	3.11E-03
C1	3.77E-03	9.10E-11	1.92E-03	6.12E-03	1.37E-09	6.21E-03
C2	2.95E-05	6.65E-03	1.89E-05	1.37E-09	1.82E-03	2.82E-11
C3	2.40E-03	7.67E-06	3.11E-03	6.21E-03	2.82E-11	1.54E-03

Mutual affinity of the oligos. Approximate dissociation constant K_d at 50 °C in M, as predicted by NUPACK:

	I	Q	S	C1	C2	C3
I	1.48E-03	1.64E-07	3.80E-03	7.16E-03	4.79E-04	6.59E-03
Q	1.64E-07	2.80E-02	1.30E-07	3.82E-07	1.59E-02	5.79E-04
S	3.80E-03	1.30E-07	4.42E-03	6.02E-03	4.88E-04	6.85E-03
C1	7.16E-03	3.82E-07	6.02E-03	6.83E-03	3.23E-06	8.93E-03
C2	4.79E-04	1.59E-02	4.88E-04	3.23E-06	9.15E-03	1.01E-06
C3	6.59E-03	5.79E-04	6.85E-03	8.93E-03	1.01E-06	5.67E-03

0.1-50 °C YES-gate with compensatory cascade, Supplementary Fig. 7 (compl_size = 3 optimization)

Optimized sequences and concentrations

ID	Sequence, 5'-3'	Concentration, µM	Hairpin formation energy at 25 °C, kcal/mol
I	AAATAAGGAGGAAGCTAAAC	5,00	-0,72
Q	ACCTACCTTCCTCCTTCCTA	0,83	0,00
S	GATTAAGGAGGCAGGTGTCT	0,12	0,00
M1	TGCGACGGAGGCAGGTAGAT	2,80	-1,41
M2	ACATTTCCTCAACACTCGTC	0,93	0,00
M3	TACGGGGGCCAGGGATTGGT	3,20	-2,95

Mutual affinity of the oligos. Approximate dissociation constant K_d at 25 °C in M, as predicted by NUPACK:

	I	Q	S	M1	M2	M3
**I**	6,7E-04	3,8E-11	1,7E-03	4,3E-03	2,6E-06	1,0E-02
**Q**	3,8E-11	3,0E-02	4,2E-11	1,3E-10	1,1E-02	4,0E-05
**S**	1,7E-03	4,2E-11	1,0E-03	2,2E-03	2,4E-05	2,6E-03
**M1**	4,3E-03	1,3E-10	2,2E-03	1,9E-03	4,7E-05	1,8E-03
**M2**	2,6E-06	1,1E-02	2,4E-05	4,7E-05	3,9E-03	1,2E-04
**M3**	1,0E-02	4,0E-05	2,6E-03	1,8E-03	1,2E-04	4,2E-04

Mutual affinity of the oligos. Approximate dissociation constant K_d at 50 °C in M, as predicted by NUPACK:

	I	Q	S	M1	M2	M3
**I**	1,5E-03	1,6E-07	4,0E-03	8,5E-03	2,0E-04	1,2E-02
**Q**	1,6E-07	2,8E-02	1,6E-07	7,7E-07	1,6E-02	7,2E-04
**S**	4,0E-03	1,6E-07	4,7E-03	7,7E-03	5,4E-04	5,5E-03
**M1**	8,5E-03	7,7E-07	7,7E-03	7,4E-03	1,6E-03	7,7E-03
**M2**	2,0E-04	1,6E-02	5,4E-04	1,6E-03	9,6E-03	1,9E-03
**M3**	1,2E-02	7,2E-04	5,5E-03	7,7E-03	1,9E-03	1,6E-03

Kinetics study (Fig. 3A)

For this experiment oligonucleotides from manufacturer Lumiprobe (Moscow, Russia) were used.

Sequences and concentrations:

ID	Sequence, 5'-3'	Concentration, µM
S	[Cy3]GGCTTAACATATTTCAGGGG	0.10
Q1	TCCCTGGATTATGTTCTGCG[BHQ2]	0.10
I1	GGCTTAACATATTTCAGGGA	0.10
Q2	CCCCCGAAATATGTCATCAC[BHQ2]	0.10
I2	GGCATAACATATTTCAGGGG	0.10
Q3	CCCCTGAAATATATTATGTA[BHQ2]	0.10
I3	TGCTTAACATATTTCAGGGG	0.10
Q4	CCCGTGAAATATAGTAAGCC[BHQ2]	0.10
I4	GGCTTAATATATTTCAGGGG	0.10
Q5	CCCCTATAATATGTTAAGCC[BHQ2]	0.10
I5	GGCTTAACATATTACAGGGG	0.10

Mutual affinity of the oligos. Approximate dissociation constant K_d at 25 °C in M, as predicted by NUPACK:

	S	Q1	I1	Q2	I2	Q3	I3	Q4	I4	Q5	I5
S	1.9E-03	3.2E-09	1.8E-03	1.7E-10	1.3E-03	1.8E-11	2.0E-03	5.5E-13	8.6E-04	8.6E-15	1.9E-03
Q1	3.2E-09	1.0E-04	1.6E-09	6.5E-04	2.3E-10	6.7E-04	3.5E-09	3.5E-04	1.7E-06	5.5E-04	3.6E-10
I1	1.8E-03	1.6E-09	1.7E-03	7.6E-10	1.2E-03	4.0E-11	1.9E-03	1.1E-12	7.3E-04	1.9E-14	1.8E-03
Q2	1.7E-10	6.5E-04	7.6E-10	5.2E-04	1.5E-10	7.8E-04	1.9E-10	2.5E-04	1.4E-08	5.2E-03	6.2E-08
I2	1.3E-03	2.3E-10	1.2E-03	1.5E-10	8.3E-04	2.6E-13	8.1E-04	1.8E-10	7.7E-04	4.5E-12	1.3E-03
Q3	1.8E-11	6.7E-04	4.0E-11	7.8E-04	2.6E-13	5.9E-05	1.8E-11	2.8E-04	5.1E-14	7.8E-05	9.1E-09
I3	2.0E-03	3.5E-09	1.9E-03	1.9E-10	8.1E-04	1.8E-11	2.0E-03	2.1E-12	8.6E-04	3.3E-14	2.0E-03
Q4	5.5E-13	3.5E-04	1.1E-12	2.5E-04	1.8E-10	2.8E-04	2.1E-12	5.2E-04	8.6E-15	5.8E-04	3.2E-10
I4	8.6E-04	1.7E-06	7.3E-04	1.4E-08	7.7E-04	5.1E-14	8.6E-04	8.6E-15	9.1E-06	6.8E-11	5.4E-04
Q5	8.6E-15	5.5E-04	1.9E-14	5.2E-03	4.5E-12	7.8E-05	3.3E-14	5.8E-04	6.8E-11	2.4E-03	2.7E-15
I5	1.9E-03	3.6E-10	1.8E-03	6.2E-08	1.3E-03	9.1E-09	2.0E-03	3.2E-10	5.4E-04	2.7E-15	1.8E-03

Mutual affinity of the oligos. Approximate dissociation constant K_d at 50 °C in M, as predicted by NUPACK:

	S	Q1	I1	Q2	I2	Q3	I3	Q4	I4	Q5	I5
S	3.5E-03	1.6E-05	3.4E-03	5.1E-07	3.6E-03	5.5E-08	3.7E-03	1.7E-07	2.4E-03	2.4E-09	3.4E-03
Q1	1.6E-05	8.6E-04	6.8E-06	1.8E-03	6.1E-06	2.6E-03	1.7E-05	1.6E-03	6.3E-05	1.7E-03	1.5E-06
I1	3.4E-03	6.8E-06	3.3E-03	9.1E-07	3.5E-03	8.3E-08	3.5E-03	2.4E-07	2.3E-03	3.5E-09	3.3E-03
Q2	5.1E-07	1.8E-03	9.1E-07	2.1E-03	5.5E-07	2.6E-03	5.5E-07	1.6E-03	1.3E-05	4.9E-03	2.1E-05
I2	3.6E-03	6.1E-06	3.5E-03	5.5E-07	3.6E-03	1.5E-08	3.1E-03	3.2E-06	2.5E-03	2.6E-07	3.5E-03
Q3	5.5E-08	2.6E-03	8.3E-08	2.6E-03	1.5E-08	1.1E-03	5.7E-08	2.0E-03	3.5E-09	1.8E-03	2.8E-06
I3	3.7E-03	1.7E-05	3.5E-03	5.5E-07	3.1E-03	5.7E-08	3.8E-03	4.6E-07	2.5E-03	7.3E-09	3.5E-03
Q4	1.7E-07	1.6E-03	2.4E-07	1.6E-03	3.2E-06	2.0E-03	4.6E-07	2.3E-03	3.1E-09	2.3E-03	1.0E-05
I4	2.4E-03	6.3E-05	2.3E-03	1.3E-05	2.5E-03	3.5E-09	2.5E-03	3.1E-09	4.9E-04	2.8E-06	2.0E-03
Q5	2.4E-09	1.7E-03	3.5E-09	4.9E-03	2.6E-07	1.8E-03	7.3E-09	2.3E-03	2.8E-06	3.4E-03	3.2E-10
I5	3.4E-03	1.5E-06	3.3E-03	2.1E-05	3.5E-03	2.8E-06	3.5E-03	1.0E-05	2.0E-03	3.2E-10	3.3E-03

NUPACK prediction accuracy screening (Fig. 3B)

For this experiment oligonucleotides from manufacturer Lumiprobe (Moscow, Russia) were used.

ID	Sequence, 5'-3'	Concentration, nM
S	[Cy3]AAAGAGTAAAGTGACGGGC	50
Q	CACCTACGCTTTACTCTAT[BHQ2]	200
I	See table below	800

The following table lists affinity between I and Q as concentration of the duplex C_{AB} (μM) when oligos are mixed at 1 μM each. Affinity is either assessed from experiment or predicted by NUPACK. The maximum concentration of triple complexes, when NUPACK max_compl_size was set to 3, was 0.046 nM.

For the experiment in Fig. 4 oligonucleotide #69 was used as input oligonucleotide, #62 as compensatory. For the experiment in Supplementary Fig. 9, oligonucleotide #69 was used as input oligonucleotide, #59 as compensatory.

ID	I sequence, 5'-3'	Experimental affinity at 25 °C	NUPACK affinity at 25 °C	Experimental affinity at 50 °C	NUPACK affinity at 50 °C
#1	TCAGAGTAAAGCGTGTGTA	0.88	0.99975	0.62	0.93409
#2	ATAGAGTAAAGCGGAATAT	0.90	0.99974	0.66	0.91294
#3	ATAGAGTAAAGCGATAAAG	0.85	0.99974	0.62	0.90610
#4	TTAGAGTAAAGCGAGTGTT	0.86	0.99973	0.49	0.90153
#5	TGAGAGTAAAGCGTCGATT	0.79	0.99972	0.45	0.93687
#6	AGGGAGTAAAGCGTGGATA	0.89	0.99972	0.67	0.87406
#7	GCAGAGTAAAGCGTATTAG	0.87	0.99971	0.60	0.93357
#8	ATAGAGTAAAGCGACCATT	0.81	0.99969	0.39	0.91016
#9	ATAGAGTAAAAGCGAGGTA	0.93	0.99969	0.67	0.66933
#10	CTGGAGTAAAGCGTGGATT	0.89	0.99968	0.64	0.86682
#11	GTGGAGTAAAGCGAGGGTA	0.83	0.99967	0.60	0.85715
#12	TTAGAGTAAAGCGAAAATT	0.66	0.99966	0.37	0.90454
#13	AATGAGTAAAGCGAGGATG	0.79	0.99964	0.49	0.80298
#14	GAAGAGTAAAGCGTTCGTT	0.82	0.99963	0.59	0.92359
#15	GCAGAGTAAAGCGGTGGGG	0.89	0.99963	0.59	0.88837
#16	CTAGAGTAAAGCGGGATTG	0.81	0.99962	0.34	0.88221
#17	GAGGTGTAAAGGGTAGGTG	0.92	0.99959	0.66	0.81012
#18	TCAGTGTAAAGCGTTGGTA	0.90	0.99959	0.59	0.88148
#19	GTAGAGTAATGCGTTGGAA	0.89	0.99958	0.45	0.78540
#20	GGAGTGTAAAGGGTAGGTT	0.89	0.99957	0.47	0.83694
#21	AGAGAGTAAAGCGGGAATG	0.82	0.99957	0.42	0.88697
#22	TTTGAGTAAAGGGTAGGCA	0.91	0.99956	0.61	0.82399
#23	TTAGAGTAAAGCGACCATG	0.65	0.99955	0.16	0.89561
#24	GTAGAGTAAAGCGAATTGG	0.80	0.99954	0.27	0.88657
#25	AAAGAGTAAAGGGTAGAAC	0.87	0.99953	0.43	0.82496
#26	AAAGAGGAAAGCGTTGGGT	0.92	0.99953	0.65	0.90185
#27	GTGGAGTAAAGCGTAAATC	0.78	0.99953	0.54	0.88068
#28	TGAGAGTAAAGCGAGCGTG	0.64	0.99952	0.27	0.89676
#29	GTGGAGTAAAGCGTGTGTC	0.89	0.99952	0.58	0.86093
#30	TTGGAGTAAAGCGTATATT	0.85	0.99950	0.61	0.86693
#31	TTGGAGTAAAGCGGGGGTC	0.87	0.99948	0.47	0.82851
#32	CTAGTGTAAAGCGTTGGGA	0.89	0.99948	0.52	0.78819
#33	CTAGAGAAAAGCGTTGGAT	0.85	0.99948	0.32	0.84854
#34	AGAGAGTAAAGCAGGTGTG	0.90	0.99947	0.48	0.81452
#35	AAAGAGTTAAGCGTGGTGG	0.87	0.99947	0.47	0.76478
#36	ATTGAGTAAAGCGTAAAAC	0.85	0.99946	0.55	0.87333
#37	GGTGAGTAAAGGGTTGGTG	0.90	0.99945	0.42	0.74641
#38	CTAGAGTAAAGCGGACATC	0.82	0.99944	0.52	0.88174
#39	AGAGAGTAAAGCGAATGAC	0.57	0.99943	0.12	0.89242
#40	CAAGAGTAAAGCGAACGGG	0.72	0.99943	0.15	0.89098
#41	TAAGAGTAAAGCATAGGAC	0.85	0.99942	0.37	0.85810
#42	CTGGAGTAAAGAGTAGGAC	0.84	0.99941	0.41	0.78648
#43	ATAGAGTAAAGGGATAGGG	0.93	0.99941	0.51	0.55584
#44	CTAGAGGAAAGCGTGGATA	0.88	0.99940	0.50	0.86036
#45	ATGGAGTAAAGCGGGGGGA	0.80	0.99939	0.42	0.79678

#46	TAAGAGAAAAGGGTAGGTC	0.84	0.99938	0.22	0.85573
#47	CAAGAGGAAAGCGTGGGTA	0.91	0.99937	0.61	0.90223
#48	GAAGAGTAAAGCGGATGAG	0.80	0.99937	0.33	0.88140
#49	ATAGGGTAAAGCGAGGTGG	0.94	0.99934	0.67	0.65844
#50	TTGCGGTAAAGGGTAGGTG	0.89	0.99932	0.50	0.77118
#51	AAGATGTAAAGCGTCGGTG	0.88	0.99930	0.45	0.76674
#52	AGAGTATAAAGCGTTGGTA	0.81	0.99930	0.15	0.81596
#53	GGAGAGTAAAGGGTAGTTC	0.87	0.99930	0.30	0.77387
#54	AGAGAGAAAAGCGTTGGAA	0.88	0.99928	0.40	0.84555
#55	ATAGAGGAAAGCGATGGAT	0.88	0.99927	0.50	0.82222
#56	ATAGAGGAAAGCGTACAGA	0.91	0.99926	0.59	0.88364
#57	TGGGAGTAAAGCGTCGTTG	0.84	0.99925	0.50	0.85019
#58	TTAGAGTAAAGAGTAGAGA	0.84	0.99925	0.28	0.73390
#59	TGGGAGTAATGGGTAGGTG	0.87	0.99923	0.21	0.61236
#60	GATAAGTAAAGAGTAGGTG	0.90	0.99922	0.38	0.80474
#61	TTTGAGGAAAGCGTTGGTC	0.90	0.99920	0.51	0.87771
#62	TTAGAGTAAAGGGCAGGAA	0.80	0.99916	0.06	0.73106
#63	ATAGAGGAAAGCGGGGATA	0.89	0.99915	0.46	0.80554
#64	AGAGAGTAAAGCGGTCGTT	0.68	0.99914	0.18	0.85439
#65	AGGAAGTAAAGGGTAGGGG	0.88	0.99914	0.48	0.76946
#66	ATAGAGTAAAGCGGCAGGA	0.92	0.99996	0.57	0.95230
#67	TGTGAGTAAAGCGTTGGTC	0.92	0.99992	0.61	0.95843
#68	ACAGAGTAAAGCGAGGTAG	0.85	0.99990	0.56	0.93999
#69	GGAGAGTAAAGCGTTGTGG	0.93	0.99989	0.71	0.94370
#70	TTAGAGTAAAGCGTAAAAC	0.89	0.99988	0.59	0.95303
#71	GTAGAGTAAAGCGGGGGGA	0.84	0.99986	0.39	0.91965
#72	CTAGAGTAAAGCGTGATGG	0.91	0.99986	0.63	0.94302
#73	ATAGAGTAAAGCGTCCCCA	0.92	0.99985	0.67	0.94671
#74	ACTGAGTAAAGCGTTGGAT	0.88	0.99985	0.45	0.92442
#75	CTAGAGTAAAGCGTATCAA	0.89	0.99985	0.49	0.94701
#76	GACGAGTAAAGGGTAGGTT	0.90	0.99985	0.55	0.92162
#77	AGAGAGTAAAGCGTGTGAT	0.92	0.99985	0.70	0.94213
#78	TCAGAGTAAAGCGTAAATA	0.84	0.99983	0.49	0.94774
#79	GCAGAGTAAAGCGTGGATC	0.88	0.99982	0.53	0.93723
#80	AAAGAGTAAAGCGAGGGAC	0.86	0.99982	0.44	0.91807
#81	AGAGAGTAAAGCGTGATAG	0.87	0.99981	0.55	0.94196
#82	GCAGAGTAAAGAGTAGGAA	0.87	0.99981	0.29	0.90773
#83	GCTGAGTAAAGGGTAGGAG	0.90	0.99980	0.52	0.88529
#84	CCAGAGTAAAGCGTATGAC	0.86	0.99980	0.47	0.94112
#85	GAAGAGTAAAGCGTGAATG	0.84	0.99979	0.58	0.93776
#86	CTAGAGTAAAGCGTCGAGA	0.87	0.99979	0.53	0.94173
#87	AATGAGTAAAGCGTCGGAC	0.89	0.99979	0.52	0.90687
#88	GTAGAGTAAAGCGTCCATT	0.86	0.99978	0.54	0.93590
#89	CAAGAGTAAAGAGTAGGGT	0.90	0.99977	0.54	0.88199
#90	GGAGAGTAAAGCGTGCGAG	0.79	0.99977	0.52	0.93906
#91	GTAGAGTAAAGCGTTAAAG	0.90	0.99977	0.64	0.93254
#92	TGGGAGTAAAGGGTAGGCG	0.90	0.99976	0.48	0.85413
#93	CGAGAGTAAAGCGGGGTTG	0.87	0.99976	0.58	0.92020
#94	TTGGAGTAAAGCGTCGGGA	0.92	0.99975	0.65	0.89581
#95	TTAGAGTAAAGCGGTGAGG	0.91	0.99975	0.65	0.88884

Experimental temperature-independent YES-gate (Fig. 4).

Optimized sequences and concentrations

ID	Sequence, 5'-3'	Concentration, μM	Hairpin formation energy at 25 °C, kcal/mol
I	GGAGAGTAAAGCGTTGTGG	10,00	0,00
C1	TTAGAGTAAAGGGCAGGAA	7,00	0,00
Q	CACCTACGCTTTACTCTAT	1,90	0,00
S	AAAGAGTAAAGTGACGGGC	0,24	0,00

Mutual affinity of the oligos. Approximate dissociation constant K_d at 25 °C in M, as predicted by NUPACK:

	I	C1	Q	S
I	3,0E-04	1,8E-03	1,1E-14	7,9E-04
C1	1,8E-03	2,7E-04	7,0E-13	8,3E-04
Q	1,1E-14	7,0E-13	1,6E-03	9,9E-10
S	7,9E-04	8,3E-04	9,9E-10	8,1E-04

Mutual affinity of the oligos. Approximate dissociation constant K_d at 50 °C in M, as predicted by NUPACK:

	I	C1	Q	S
I	5,8E-03	8,0E-03	3,4E-09	3,7E-03
C1	8,0E-03	3,9E-03	9,9E-08	5,3E-03
Q	3,4E-09	9,9E-08	7,6E-03	5,5E-06
S	3,7E-03	5,3E-03	5,5E-06	4,6E-03

Below we give affinities for the same oligos, approximately calculated from experiment. Values in blank cells were not measured.

Mutual affinity of the oligos. Approximate dissociation constant K_d at 25 °C in M, as measured experimentally:

	I	C1	Q	S
I			5,7E-08	
C1			1,2E-07	
Q	5,7E-08	1,2E-07		2,9E-08
S			2,9E-08	

Mutual affinity of the oligos. Approximate dissociation constant K_d at 50 °C in M, as measured experimentally:

	I	C1	Q	S
I			5,6E-07	
C1			1,6E-05	
Q	5,6E-07	1,6E-05		1,0E-06
S			1,0E-06	

Experimental temperature-independent YES-gate (Supplementary Fig. 9, replicate of experiment in Fig. 4).

For this experiment oligonucleotides from manufacturer Lumiprobe (Moscow, Russia) were used.

Optimized sequences and concentrations

ID	Sequence, 5'-3'	Concentration, µM	Hairpin formation energy at 25 °C, kcal/mol
I	GGAGAGTAAAGCGTTGTGG	2.00	0.00
C1	TGGGAGTAATGGGTAGGTG	1.40	0.00
Q	CACCTACGCTTTACTCTAT[BHQ2]	0.70	0.00
S	[Cy3]AAAGAGTAAAGTGACGGGC	0.24	0.00

Mutual affinity of the oligos. Approximate dissociation constant K_d at 25 °C in M, as predicted by NUPACK:

	I	C1	Q	S
I	3.03E-04	1.53E-02	1.15E-14	7.94E-04
C1	1.53E-02	3.11E-02	5.96E-13	5.37E-03
Q	1.15E-14	5.96E-13	1.63E-03	9.86E-10
S	7.94E-04	5.37E-03	9.86E-10	8.13E-04

Mutual affinity of the oligos. Approximate dissociation constant K_d at 50 °C in M, as predicted by NUPACK:

	I	C1	Q	S
I	5.78E-03	2.23E-02	3.36E-09	3.66E-03
C1	2.23E-02	3.50E-02	2.45E-07	1.04E-02
Q	3.36E-09	2.45E-07	7.63E-03	5.47E-06
S	3.66E-03	1.04E-02	5.47E-06	4.57E-03

Below we give affinities for the same oligos, approximately calculated from experiment. Values in blank cells were not measured.

Mutual affinity of the oligos. Approximate dissociation constant K_d at 25 °C in M, as measured experimentally:

	I	C1	Q	S
I			5.27E-09	
C1			1.94E-08	
Q	5.27E-09	1.94E-08		8.90E-09
S			8.90E-09	

Mutual affinity of the oligos. Approximate dissociation constant K_d at 50 °C in M, as measured experimentally:

	I	C1	Q	S
I			1.18E-07	
C1			2.97E-06	
Q	1.18E-07	2.97E-06		2.85E-07
S			2.85E-07	

Salt-independent gates simulation: sodium

Optimized sequences and concentrations

ID	Sequence, 5'-3'	Concentration, µM	Hairpin formation energy at 25 °C and 1M Na^+, kcal/mol
I	TTAATTCACGGTTTCTAT	5.42	0.00
Q	ATTGTAAACGTGAAGTAA	0.99	-0.97
S	GTAATTCACGTCTACACT	0.38	0.00
C	TTAATTCACTTTTACCAT	2.48	0.00

The target values for the free concentration of the output oligonucleotide were 150 nM and 300 nM in the absence and presence of the input, respectively.

Mutual affinity of the oligos. Approximate dissociation constant K_d (at 25 °C, 1M Na^+) in M, as predicted by NUPACK:

	I	Q	S	C
I	4.62E-04	1.01E-08	5.49E-04	3.52E-04
Q	1.01E-08	5.86E-04	6.73E-09	2.27E-08
S	5.49E-04	6.73E-09	3.13E-04	1.39E-03
C	3.52E-04	2.27E-08	1.39E-03	3.00E-03

Mutual affinity of the oligos. Approximate dissociation constant K_d (at 25 °C, 0.1M Na^+) in M, as predicted by NUPACK:

	I	Q	S	C
I	1.03E-03	2.29E-07	9.97E-04	7.95E-04
Q	2.29E-07	8.75E-04	1.70E-07	9.87E-07
S	9.97E-04	1.70E-07	6.20E-04	3.52E-03
C	7.95E-04	9.87E-07	3.52E-03	5.46E-03

Salt-independent gates simulation: magnesium

Optimized sequences and concentrations

ID	Sequence, 5'-3'	Concentration, µM	Hairpin formation energy at 25 °C, kcal/mol
I	TTAATTCACGGTTTCTAT	9.29	0.00
Q	ATTGTAAACGTGAATTTA	0.55	-0.98
S	GTAATTCACGTCTACACT	0.34	0.00
M	TTAATTCACTTTTACCAT	2.35	0.00

The target values for the free concentration of the output oligonucleotide were 150 nM and 300 nM in the absence and presence of the input, respectively.

Mutual affinity of the oligos. Approximate dissociation constant K_d (at 25 °C, 10 mM Mg^{2+}, 0.05M Na^+) in M, as predicted by NUPACK:

	I	Q	S	M
I	6.52E-04	3.45E-09	7.06E-04	4.91E-04
Q	3.45E-09	6.36E-04	1.46E-09	9.98E-09
S	7.06E-04	1.46E-09	4.18E-04	2.07E-03
M	4.91E-04	9.98E-09	2.07E-03	3.78E-03

Mutual affinity of the oligos. Approximate dissociation constant K_d (at 25 °C, 0 mM Mg^{2+}, 0.05M Na^+) in M, as predicted by NUPACK:

	I	Q	S	M
I	1.30E-03	5.02E-08	1.20E-03	1.03E-03
Q	5.02E-08	7.16E-04	3.02E-08	2.72E-07
S	1.20E-03	3.02E-08	7.63E-04	4.60E-03
M	1.03E-03	2.72E-07	4.60E-03	6.71E-03

Screening for the construction of salt-independent YES-gate

ID	Sequence, 5'-3'	Concentration, nM
S	[Cy3]TTAGGAGGTGAG	100
Q	CTCACCTTAAAA[BHQ2]	100
I	See table below	1000

The following table lists affinity between I and Q as concentration of the duplex C_{AB} (μM) when oligos are mixed at 1 μM each. Affinity is either assessed from experiment or predicted by NUPACK. Based on the screening result, #1 was chosen as input oligonucleotide and #6 as compensatory oligonucleotide for the salt-independent gate.

#	I sequence, 5'-3'	Experimental affinity at 50 mM NaCl	NUPACK affinity at 50 mM NaCl	NUPACK affinity at 1000 mM NaCl	Experimental affinity at 1000 mM NaCl
1	GTTTAAGGGGAG	0,50	0,33	0,90	0,90
2	ATTGAAGGTGAG	0,40	0,58	0,94	0,89
3	TGTTAAGGTGAA	0,25	0,67	0,96	0,85
4	TTGTAAGGTGCG	0,10	0,27	0,79	0,76
5	TGTTGAGGTGAG	0,19	0,26	0,78	0,81
6	TTTTAAGGTCGG	0,19	0,31	0,87	0,83
7	GTTTAAGGTGGG	0,34	0,68	0,96	0,89
8	TTTTTAGGTGTG	0,14	0,14	0,74	0,92
9	CTTTAAGGTCGG	0,18	0,22	0,79	0,63
10	CTAGAAGGTGAG	0,16	0,51	0,90	0,70
11	TTCTAAGGTGTG	0,18	0,27	0,77	0,64
12	CATTTAGGTGAG	0,16	0,31	0,82	0,74
13	CTTTTAGGTGAA	0,13	0,25	0,81	0,67
14	GTTTAAGGTGCG	0,33	0,63	0,96	0,86
15	TTTTAAGGTCAT	0,14	0,29	0,86	0,69
16	GATTAAGGTGAG	0,40	0,80	0,98	0,87
17	TTTAAAGGTGGG	0,15	0,22	0,76	0,71
18	CTTTAAGGTGAT	0,19	0,82	0,98	0,81
19	TTTTAAGGTGTT	0,50	0,74	0,97	0,94
20	TATGAAGGTGAG	0,20	0,54	0,92	0,76
21	TTTTTAGGTGAA	0,26	0,31	0,86	0,85
22	TGGTAAGGTGAG	0,37	0,64	0,95	0,84
23	TGTTAAGGTGAT	0,20	0,66	0,95	0,76
24	TCTGAAGGTGAA	0,02	0,35	0,81	0,22

Salt-independent gates: sodium

Optimized sequences and concentrations

ID	Sequence, 5'-3'	Concentration, µM	Hairpin formation energy at 25 °C, kcal/mol
I	GTTTAAGGGGAG	7,00	0,00
C1	TTTTAAGGTCGG	1,00	0,00
Q	CTCACCTTAAAA	3,49	0,00
S	TTAGGAGGTGAG	0,23	0,00

Mutual affinity of the oligos. Approximate dissociation constant K_d (at 25 °C, 1M Na^+) in M, as predicted by NUPACK:

	I	C1	Q	S
I	3.1E-03	2.6E-03	1.1E-08	2.5E-02
C1	2.6E-03	9.3E-04	1.8E-08	9.2E-03
Q	1.1E-08	1.8E-08	3.1E-03	7.9E-08
S	2.5E-02	9.2E-03	7.9E-08	4.8E-02

Mutual affinity of the oligos. Approximate dissociation constant K_d (at 25 °C, 0.1M Na^+) in M, as predicted by NUPACK:

	I	C1	Q	S
I	1.1E-02	8.0E-03	4.8E-07	4.5E-02
C1	8.0E-03	2.4E-03	5.6E-07	1.6E-02
Q	4.8E-07	5.6E-07	1.0E-02	1.1E-06
S	4.5E-02	1.6E-02	1.1E-06	6.8E-02

Below we give affinities for the same oligos, approximately calculated from experiment. Values in blank cells were not measured.

Mutual affinity of the oligos. Approximate dissociation constant K_d (at 25 °C, 1M Na^+) in M, as measured experimentally:

	I	C1	Q	S
I			1,4E-07	
C1			4,1E-07	
Q	1,4E-07	4,1E-07		8,7E-08
S			8,7E-08	

Mutual affinity of the oligos. Approximate dissociation constant K_d (at 25 °C, 0.1M Na^+) in M, as measured experimentally:

	I	C1	Q	S
I			8,8E-07	
C1			3,9E-06	
Q	8,8E-07	3,9E-06		5,1E-07
S			5,1E-07	

Salt-independent gates: magnesium

Optimized sequences and concentrations

ID	Sequence, 5'-3'	Concentration, µM	Hairpin formation energy at 25 °C, kcal/mol
I	GTTTAAGGGGAG	3,00	0,00
C1	TTTTAAGGTCGG	0,70	0,00
Q	CTCACCTTAAAA	1,32	0,00
S	TTAGGAGGTGAG	0,23	0,00

Mutual affinity of the oligos. Approximate dissociation constant K_d (at 25 °C, 10 mM Mg^{2+}) in M, as predicted by NUPACK:

	I	C1	Q	S
I	5.3E-03	4.3E-03	5.5E-08	3.2E-02
C1	4.3E-03	1.4E-03	7.8E-08	1.2E-02
Q	5.5E-08	7.8E-08	5.2E-03	2.4E-07
S	3.2E-02	1.2E-02	2.4E-07	5.5E-02

Mutual affinity of the oligos. Approximate dissociation constant K_d (at 25 °C, 1 mM Mg^{2+}) in M, as predicted by NUPACK:

	I	C1	Q	S
I	8.6E-03	6.6E-03	2.4E-07	4.0E-02
C1	6.6E-03	2.0E-03	3.0E-07	1.5E-02
Q	2.4E-07	3.0E-07	8.1E-03	6.9E-07
S	4.0E-02	1.5E-02	6.9E-07	6.4E-02

Below we give affinities for the same oligos, approximately calculated from experiment. Values in blank cells were not measured.

Mutual affinity of the oligos. Approximate dissociation constant K_d (at 25 °C, 10 mM Mg^{2+}) in M, as measured experimentally:

	I	C1	Q	S
I			7,5E-08	
C1			2,2E-07	
Q	7,5E-08	2,2E-07		9,2E-08
S			9,2E-08	

Mutual affinity of the oligos. Approximate dissociation constant K_d (at 25 °C, 1 mM Mg^{2+}) in M, as measured experimentally:

	I	C1	Q	S
I			2,4E-07	
C1			1,3E-06	
Q	2,4E-07	1,3E-06		4,1E-07
S			4,1E-07	

Receptor-activator networks

Please note, that parameters for receptor-activator networks with 100 receptors (Fig. 6D, Supplementary Fig. 14, 15) are provided in the corresponding Source Data files.

For all networks the following optimization parameters were used:

	Receptors	Activators	Compensators Ci	Compensators Di
Concentration lower bound, nM	499	100	100	100
Concentration upper bound, nM	500	5000	5000	5000
Concentration start value, nM	500	500	500	500
	Receptor-Activator	Activator-Compensator Ci	Receptor-Compensator Di	
Gibbs energy at 25 °C lower bound, kJ/mol	-60	-60	-60	
Gibbs energy at 25 °C upper bound, kJ/mol	-20	-20	-20	
Gibbs energy at 25 °C start value, kJ/mol	-45	-45	-45	
Easy-to-melt level	p95	p5	p5	

N = 1 case (1 receptor, 1 activator)

Optimized concentrations of the network participants

ID	Concentration, µM
R0	0,50
A0	0,50
C0	0,50

Mutual affinity of the oligos. Approximate dissociation constant K_d (25 °C) in M.

	R0	A0	C0
R0	1,0E+00	6,7E-11	1,0E+00
A0	6,7E-11	1,0E+00	6,7E-11
C0	1,0E+00	6,7E-11	1,0E+00

Mutual affinity of the oligos. Approximate dissociation constant K_d (50 °C) in M.

	R0	A0	C0
R0	1,0E+00	2,4E-07	1,0E+00
A0	2,4E-07	1,0E+00	1,1E-05
C0	1,0E+00	1,1E-05	1,0E+00

N = 2 case (2 receptors, 2 activators)

Optimized concentrations of the network participants:

ID	Concentration, µM
R0	0,50
A0	1,02
R1	0,50
A1	0,35
C0	1,34
C1	2,22

Mutual affinity of the oligos. Approximate dissociation constant K_d (25 °C) in M:

	R0	A0	R1	A1	C0	C1
**R0**	1,0E+00	1,9E-08	1,0E+00	1,4E-10	1,0E+00	1,0E+00
**A0**	1,9E-08	1,0E+00	4,6E-10	1,0E+00	1,0E-10	1,0E+00
**R1**	1,0E+00	4,6E-10	1,0E+00	5,5E-11	1,0E+00	1,0E+00
**A1**	1,4E-10	1,0E+00	5,5E-11	1,0E+00	1,0E+00	2,7E-04
**C0**	1,0E+00	1,0E-10	1,0E+00	1,0E+00	1,0E+00	1,0E+00
**C1**	1,0E+00	1,0E+00	1,0E+00	2,7E-04	1,0E+00	1,0E+00

Mutual affinity of the oligos. Approximate dissociation constant K_d (50 °C) in M:

	R0	A0	R1	A1	C0	C1
**R0**	1,0E+00	7,3E-06	1,0E+00	3,7E-07	1,0E+00	1,0E+00
**A0**	7,3E-06	1,0E+00	7,9E-07	1,0E+00	1,4E-05	1,0E+00
**R1**	1,0E+00	7,9E-07	1,0E+00	2,1E-07	1,0E+00	1,0E+00
**A1**	3,7E-07	1,0E+00	2,1E-07	1,0E+00	1,0E+00	7,8E-03
**C0**	1,0E+00	1,4E-05	1,0E+00	1,0E+00	1,0E+00	1,0E+00
**C1**	1,0E+00	1,0E+00	1,0E+00	7,8E-03	1,0E+00	1,0E+00

N = 3 case (3 receptors, 3 activators)

Optimized concentrations of the network participants

ID	Concentration, µM
R0	0,50
A0	0,50
R1	0,50
A1	0,50
R2	0,50
A2	0,50
C0	0,50
D0	0,86
C1	0,50
D1	0,50
C2	0,10
D2	0,50

Mutual affinity of the oligos. Approximate dissociation constant K_d (25 °C) in M.

	R0	A0	R1	A1	R2	A2	C0	D0	C1	D1	C2	D2
R0	1,0E+00	4,5E-08	1,0E+00	9,0E-09	1,0E+00	1,0E-06	1,0E+00	1,4E-05	1,0E+00	1,0E+00	1,0E+00	1,0E+00
A0	4,5E-08	1,0E+00	2,8E-09	1,0E+00	2,4E-04	1,0E+00	1,6E-10	1,0E+00	1,0E+00	1,0E+00	1,0E+00	1,0E+00
R1	1,0E+00	2,8E-09	1,0E+00	4,2E-07	1,0E+00	1,4E-10	1,0E+00	1,0E+00	1,0E+00	9,1E-09	1,0E+00	1,0E+00
A1	9,0E-09	1,0E+00	4,2E-07	1,0E+00	6,7E-11	1,0E+00	1,0E+00	1,0E+00	9,7E-11	1,0E+00	1,0E+00	1,0E+00
R2	1,0E+00	2,4E-04	1,0E+00	6,7E-11	1,0E+00	3,4E-11	1,0E+00	1,0E+00	1,0E+00	1,0E+00	1,0E+00	1,2E-08
A2	1,0E-06	1,0E+00	1,4E-10	1,0E+00	3,4E-11	1,0E+00	1,0E+00	1,0E+00	1,0E+00	1,0E+00	2,0E-04	1,0E+00
C0	1,0E+00	1,6E-10	1,0E+00	1,0E+00	1,0E+00	1,0E+00	1,0E+00	1,0E+00	1,0E+00	1,0E+00	1,0E+00	1,0E+00
D0	1,4E-05	1,0E+00	1,0E+00	1,0E+00	1,0E+00	1,0E+00	1,0E+00	1,0E+00	1,0E+00	1,0E+00	1,0E+00	1,0E+00
C1	1,0E+00	1,0E+00	1,0E+00	9,7E-11	1,0E+00	1,0E+00	1,0E+00	1,0E+00	1,0E+00	1,0E+00	1,0E+00	1,0E+00
D1	1,0E+00	1,0E+00	9,1E-09	1,0E+00	1,0E+00	1,0E+00	1,0E+00	1,0E+00	1,0E+00	1,0E+00	1,0E+00	1,0E+00
C2	1,0E+00	1,0E+00	1,0E+00	1,0E+00	1,0E+00	2,0E-04	1,0E+00	1,0E+00	1,0E+00	1,0E+00	1,0E+00	1,0E+00
D2	1,0E+00	1,0E+00	1,0E+00	1,0E+00	1,2E-08	1,0E+00	1,0E+00	1,0E+00	1,0E+00	1,0E+00	1,0E+00	1,0E+00

Mutual affinity of the oligos. Approximate dissociation constant K_d (50 °C) in M.

	R0	A0	R1	A1	R2	A2	C0	D0	C1	D1	C2	D2
R0	1,0E+00	1,2E-05	1,0E+00	4,7E-06	1,0E+00	7,5E-05	1,0E+00	3,9E-03	1,0E+00	1,0E+00	1,0E+00	1,0E+00
A0	1,2E-05	1,0E+00	2,4E-06	1,0E+00	1,6E-03	1,0E+00	1,9E-05	1,0E+00	1,0E+00	1,0E+00	1,0E+00	1,0E+00
R1	1,0E+00	2,4E-06	1,0E+00	4,5E-05	1,0E+00	3,7E-07	1,0E+00	1,0E+00	1,0E+00	1,8E-04	1,0E+00	1,0E+00
A1	4,7E-06	1,0E+00	4,5E-05	1,0E+00	2,4E-07	1,0E+00	1,0E+00	1,0E+00	1,3E-05	1,0E+00	1,0E+00	1,0E+00
R2	1,0E+00	1,6E-03	1,0E+00	2,4E-07	1,0E+00	1,6E-07	1,0E+00	1,0E+00	1,0E+00	1,0E+00	1,0E+00	2,1E-04
A2	7,5E-05	1,0E+00	3,7E-07	1,0E+00	1,6E-07	1,0E+00	1,0E+00	1,0E+00	1,0E+00	1,0E+00	7,4E-03	1,0E+00
C0	1,0E+00	1,9E-05	1,0E+00	1,0E+00	1,0E+00	1,0E+00	1,0E+00	1,0E+00	1,0E+00	1,0E+00	1,0E+00	1,0E+00
D0	3,9E-03	1,0E+00	1,0E+00	1,0E+00	1,0E+00	1,0E+00	1,0E+00	1,0E+00	1,0E+00	1,0E+00	1,0E+00	1,0E+00
C1	1,0E+00	1,0E+00	1,0E+00	1,3E-05	1,0E+00	1,0E+00	1,0E+00	1,0E+00	1,0E+00	1,0E+00	1,0E+00	1,0E+00
D1	1,0E+00	1,0E+00	1,8E-04	1,0E+00	1,0E+00	1,0E+00	1,0E+00	1,0E+00	1,0E+00	1,0E+00	1,0E+00	1,0E+00
C2	1,0E+00	1,0E+00	1,0E+00	1,0E+00	1,0E+00	7,4E-03	1,0E+00	1,0E+00	1,0E+00	1,0E+00	1,0E+00	1,0E+00
D2	1,0E+00	1,0E+00	1,0E+00	1,0E+00	2,1E-04	1,0E+00	1,0E+00	1,0E+00	1,0E+00	1,0E+00	1,0E+00	1,0E+00

Algebraic systems

For all systems the following optimization parameters were used:

**Concentration lower bound, nM**	1
**Concentration upper bound, nM**	10000
**Concentration of output species, nM**	30
**Starting number of nodes**	9
**Gibbs energy at 25 °C lower bound, kJ/mol**	-60
**Gibbs energy at 25 °C upper bound, kJ/mol**	-20
**Easy-to-melt level of "main" species**	p95
**Easy-to-melt level of "compensatory" species**	p5
**Pruning concentration threshold, nM**	10
**Pruning gibbs energy threshold, kJ/mol**	-25

Line function – temperature independent system.

Concentrations of the participants.

ID	Concentration, µM
M1	6.09
M2	1.93
M3	7.52
M4	6.68
M5	3.17
M6	9.18
M7	8.60
S	0.03
C1	4.93
C2	8.96
C3	9.93
C4	8.64
C5	0.21
C6	1.09
C7	7.00
C8	9.53
C9	0.55

Mutual affinity of the participants. Approximate dissociation constant K_d (25 °C) in M.

	I	M1	M2	M3	M4	M5	M6	M7	S	C1	C2	C3	C4	C5	C6	C7	C8	C9
I	1.0E+00	1.8E-04	7.9E-09	2.1E-09	4.4E-09	1.5E-04	6.0E-07	2.0E-04	3.1E-04	1.3E-08	1.0E+00	1.0E+00	1.0E+00	1.0E+00	1.0E+00	1.0E+00	1.0E+00	1.0E+00
M1	1.8E-04	1.0E+00	3.8E-10	9.1E-08	4.1E-09	3.0E-11	9.0E-07	1.8E-07	3.0E-06	1.0E+00	8.3E-10	1.0E+00	1.0E+00	1.0E+00	1.0E+00	1.0E+00	1.0E+00	1.0E+00
M2	7.9E-09	3.8E-10	1.0E+00	3.2E-09	6.8E-08	5.3E-09	3.6E-09	6.4E-09	7.5E-07	1.0E+00	1.0E+00	1.7E-05	1.0E+00	1.0E+00	1.0E+00	1.0E+00	1.0E+00	1.0E+00
M3	2.1E-09	9.1E-08	3.2E-09	1.0E+00	5.7E-07	6.2E-06	3.1E-04	2.5E-10	3.9E-07	1.0E+00	1.0E+00	1.0E+00	8.5E-10	1.0E+00	1.0E+00	1.0E+00	1.0E+00	1.0E+00
M4	4.4E-09	4.1E-09	6.8E-08	5.7E-07	1.0E+00	6.5E-07	6.3E-07	8.6E-07	1.4E-04	1.0E+00	1.0E+00	1.0E+00	1.0E+00	1.5E-05	1.0E+00	1.0E+00	1.0E+00	1.0E+00
M5	1.5E-04	3.0E-11	5.3E-09	6.2E-06	6.5E-07	1.0E+00	2.0E-08	6.9E-06	5.7E-06	1.0E+00	1.0E+00	1.0E+00	1.0E+00	1.0E+00	6.3E-09	1.0E+00	1.0E+00	1.0E+00
M6	6.0E-07	9.0E-07	3.6E-09	3.1E-04	6.3E-07	2.0E-08	1.0E+00	1.1E-09	2.4E-08	1.0E+00	1.0E+00	1.0E+00	1.0E+00	1.0E+00	1.0E+00	2.3E-10	1.0E+00	1.0E+00
M7	2.0E-04	1.8E-07	6.4E-09	2.5E-10	8.6E-07	6.9E-06	1.1E-09	1.0E+00	4.9E-05	1.0E+00	1.0E+00	1.0E+00	1.0E+00	1.0E+00	1.0E+00	1.0E+00	8.1E-06	1.0E+00
S	3.1E-04	3.0E-06	7.5E-07	3.9E-07	1.4E-04	5.7E-06	2.4E-08	4.9E-05	1.0E+00	1.0E+00	1.0E+00	1.0E+00	1.0E+00	1.0E+00	1.0E+00	1.0E+00	1.0E+00	2.2E-06
C1	1.3E-08	1.0E+00	1.0E+00	1.0E+00	1.0E+00	1.0E+00	1.0E+00	1.0E+00	1.0E+00	1.0E+00	1.0E+00	1.0E+00	1.0E+00	1.0E+00	1.0E+00	1.0E+00	1.0E+00	1.0E+00
C2	1.0E+00	8.3E-10	1.0E+00	1.0E+00	1.0E+00	1.0E+00	1.0E+00	1.0E+00	1.0E+00	1.0E+00	1.0E+00	1.0E+00	1.0E+00	1.0E+00	1.0E+00	1.0E+00	1.0E+00	1.0E+00
C3	1.0E+00	1.0E+00	1.7E-05	1.0E+00	1.0E+00	1.0E+00	1.0E+00	1.0E+00	1.0E+00	1.0E+00	1.0E+00	1.0E+00	1.0E+00	1.0E+00	1.0E+00	1.0E+00	1.0E+00	1.0E+00
C4	1.0E+00	1.0E+00	1.0E+00	8.5E-10	1.0E+00	1.0E+00	1.0E+00	1.0E+00	1.0E+00	1.0E+00	1.0E+00	1.0E+00	1.0E+00	1.0E+00	1.0E+00	1.0E+00	1.0E+00	1.0E+00
C5	1.0E+00	1.0E+00	1.0E+00	1.0E+00	1.5E-05	1.0E+00	1.0E+00	1.0E+00	1.0E+00	1.0E+00	1.0E+00	1.0E+00	1.0E+00	1.0E+00	1.0E+00	1.0E+00	1.0E+00	1.0E+00
C6	1.0E+00	1.0E+00	1.0E+00	1.0E+00	1.0E+00	6.3E-09	1.0E+00	1.0E+00	1.0E+00	1.0E+00	1.0E+00	1.0E+00	1.0E+00	1.0E+00	1.0E+00	1.0E+00	1.0E+00	1.0E+00
C7	1.0E+00	1.0E+00	1.0E+00	1.0E+00	1.0E+00	1.0E+00	2.3E-10	1.0E+00	1.0E+00	1.0E+00	1.0E+00	1.0E+00	1.0E+00	1.0E+00	1.0E+00	1.0E+00	1.0E+00	1.0E+00
C8	1.0E+00	1.0E+00	1.0E+00	1.0E+00	1.0E+00	1.0E+00	1.0E+00	8.1E-06	1.0E+00	1.0E+00	1.0E+00	1.0E+00	1.0E+00	1.0E+00	1.0E+00	1.0E+00	1.0E+00	1.0E+00
C9	1.0E+00	1.0E+00	1.0E+00	1.0E+00	1.0E+00	1.0E+00	1.0E+00	1.0E+00	2.2E-06	1.0E+00	1.0E+00	1.0E+00	1.0E+00	1.0E+00	1.0E+00	1.0E+00	1.0E+00	1.0E+00

Mutual affinity of the participants. Approximate dissociation constant K_d (50 °C) in M.

	I	M1	M2	M3	M4	M5	M6	M7	S	C1	C2	C3	C4	C5	C6	C7	C8	C9
I	1,0E+00	1,4E-03	4,4E-06	1,9E-06	3,1E-06	1,3E-03	5,6E-05	1,4E-03	1,8E-03	2,2E-04	1,0E+00	1,0E+00	1,0E+00	1,0E+00	1,0E+00	1,0E+00	1,0E+00	1,0E+00
M1	1,4E-03	1,0E+00	7,0E-07	1,9E-05	2,9E-06	1,5E-07	7,0E-05	2,7E-05	1,4E-04	1,0E+00	4,9E-05	1,0E+00	1,0E+00	1,0E+00	1,0E+00	1,0E+00	1,0E+00	1,0E+00
M2	4,4E-06	7,0E-07	1,0E+00	2,5E-06	1,6E-05	3,4E-06	2,7E-06	3,9E-06	6,3E-05	1,0E+00	1,0E+00	4,1E-03	1,0E+00	1,0E+00	1,0E+00	1,0E+00	1,0E+00	1,0E+00
M3	1,9E-06	1,9E-05	2,5E-06	1,0E+00	5,4E-05	2,1E-04	1,9E-03	5,4E-07	4,3E-05	1,0E+00	1,0E+00	1,0E+00	5,0E-05	1,0E+00	1,0E+00	1,0E+00	1,0E+00	1,0E+00
M4	3,1E-06	2,9E-06	1,6E-05	5,4E-05	1,0E+00	5,8E-05	5,7E-05	6,8E-05	1,2E-03	1,0E+00	1,0E+00	1,0E+00	1,0E+00	3,9E-03	1,0E+00	1,0E+00	1,0E+00	1,0E+00
M5	1,3E-03	1,5E-07	3,4E-06	2,1E-04	5,8E-05	1,0E+00	7,6E-06	2,2E-04	2,0E-04	1,0E+00	1,0E+00	1,0E+00	1,0E+00	1,0E+00	1,5E-04	1,0E+00	1,0E+00	1,0E+00
M6	5,6E-05	7,0E-05	2,7E-06	1,9E-03	5,7E-05	7,6E-06	1,0E+00	1,3E-06	8,5E-06	1,0E+00	1,0E+00	1,0E+00	1,0E+00	1,0E+00	1,0E+00	2,3E-05	1,0E+00	1,0E+00
M7	1,4E-03	2,7E-05	3,9E-06	5,4E-07	6,8E-05	2,2E-04	1,3E-06	1,0E+00	6,7E-04	1,0E+00	1,0E+00	1,0E+00	1,0E+00	1,0E+00	1,0E+00	1,0E+00	3,3E-03	1,0E+00
S	1,8E-03	1,4E-04	6,3E-05	4,3E-05	1,2E-03	2,0E-04	8,5E-06	6,7E-04	1,0E+00	1,0E+00	1,0E+00	1,0E+00	1,0E+00	1,0E+00	1,0E+00	1,0E+00	1,0E+00	2,1E-03
C1	2,2E-04	1,0E+00	1,0E+00	1,0E+00	1,0E+00	1,0E+00	1,0E+00	1,0E+00	1,0E+00	1,0E+00	1,0E+00	1,0E+00	1,0E+00	1,0E+00	1,0E+00	1,0E+00	1,0E+00	1,0E+00
C2	1,0E+00	4,9E-05	1,0E+00	1,0E+00	1,0E+00	1,0E+00	1,0E+00	1,0E+00	1,0E+00	1,0E+00	1,0E+00	1,0E+00	1,0E+00	1,0E+00	1,0E+00	1,0E+00	1,0E+00	1,0E+00
C3	1,0E+00	1,0E+00	4,1E-03	1,0E+00	1,0E+00	1,0E+00	1,0E+00	1,0E+00	1,0E+00	1,0E+00	1,0E+00	1,0E+00	1,0E+00	1,0E+00	1,0E+00	1,0E+00	1,0E+00	1,0E+00
C4	1,0E+00	1,0E+00	1,0E+00	5,0E-05	1,0E+00	1,0E+00	1,0E+00	1,0E+00	1,0E+00	1,0E+00	1,0E+00	1,0E+00	1,0E+00	1,0E+00	1,0E+00	1,0E+00	1,0E+00	1,0E+00
C5	1,0E+00	1,0E+00	1,0E+00	1,0E+00	3,9E-03	1,0E+00	1,0E+00	1,0E+00	1,0E+00	1,0E+00	1,0E+00	1,0E+00	1,0E+00	1,0E+00	1,0E+00	1,0E+00	1,0E+00	1,0E+00
C6	1,0E+00	1,0E+00	1,0E+00	1,0E+00	1,0E+00	1,5E-04	1,0E+00	1,0E+00	1,0E+00	1,0E+00	1,0E+00	1,0E+00	1,0E+00	1,0E+00	1,0E+00	1,0E+00	1,0E+00	1,0E+00
C7	1,0E+00	1,0E+00	1,0E+00	1,0E+00	1,0E+00	1,0E+00	2,3E-05	1,0E+00	1,0E+00	1,0E+00	1,0E+00	1,0E+00	1,0E+00	1,0E+00	1,0E+00	1,0E+00	1,0E+00	1,0E+00
C8	1,0E+00	1,0E+00	1,0E+00	1,0E+00	1,0E+00	1,0E+00	1,0E+00	3,3E-03	1,0E+00	1,0E+00	1,0E+00	1,0E+00	1,0E+00	1,0E+00	1,0E+00	1,0E+00	1,0E+00	1,0E+00
C9	1,0E+00	1,0E+00	1,0E+00	1,0E+00	1,0E+00	1,0E+00	1,0E+00	1,0E+00	2,1E-03	1,0E+00	1,0E+00	1,0E+00	1,0E+00	1,0E+00	1,0E+00	1,0E+00	1,0E+00	1,0E+00

Line function – control system without compensatory participants.

Concentrations of the participants.

ID	Concentration, µM
M1	5.78
M2	1.91
M3	7.58
M4	7.01
M5	3.36
M6	9.23
M7	8.59
S	0.03

Mutual affinity of the participants. Approximate dissociation constant K_d (25 °C) in M.

	I	M1	M2	M3	M4	M5	M6	M7	S
I	1.0E+00	8.0E-08	5.2E-08	1.2E-08	2.5E-09	5.1E-05	6.7E-08	1.9E-07	3.1E-06
M1	8.0E-08	1.0E+00	4.8E-10	2.2E-07	1.1E-09	1.9E-10	1.2E-09	3.6E-07	1.3E-05
M2	5.2E-08	4.8E-10	1.0E+00	1.0E-10	5.5E-09	5.4E-10	7.4E-10	1.1E-09	1.7E-07
M3	1.2E-08	2.2E-07	1.0E-10	1.0E+00	1.3E-07	3.2E-06	6.8E-05	7.3E-11	3.8E-07
M4	2.5E-09	1.1E-09	5.5E-09	1.3E-07	1.0E+00	7.6E-07	5.2E-07	8.3E-06	1.1E-04
M5	5.1E-05	1.9E-10	5.4E-10	3.2E-06	7.6E-07	1.0E+00	1.1E-07	1.5E-05	1.6E-06
M6	6.7E-08	1.2E-09	7.4E-10	6.8E-05	5.2E-07	1.1E-07	1.0E+00	1.5E-10	1.6E-08
M7	1.9E-07	3.6E-07	1.1E-09	7.3E-11	8.3E-06	1.5E-05	1.5E-10	1.0E+00	1.7E-05
S	3.1E-06	1.3E-05	1.7E-07	3.8E-07	1.1E-04	1.6E-06	1.6E-08	1.7E-05	1.0E+00

Mutual affinity of the participants. Approximate dissociation constant K_d (50 °C) in M.

	I	M1	M2	M3	M4	M5	M6	M7	S
I	1,0E+00	1,7E-05	1,3E-05	5,6E-06	2,2E-06	6,9E-04	1,5E-05	2,8E-05	1,4E-04
M1	1,7E-05	1,0E+00	8,1E-07	3,1E-05	1,3E-06	4,6E-07	1,4E-06	4,1E-05	3,2E-04
M2	1,3E-05	8,1E-07	1,0E+00	3,1E-07	3,5E-06	8,7E-07	1,0E-06	1,3E-06	2,7E-05
M3	5,6E-06	3,1E-05	3,1E-07	1,0E+00	2,3E-05	1,4E-04	8,1E-04	2,5E-07	4,3E-05
M4	2,2E-06	1,3E-06	3,5E-06	2,3E-05	1,0E+00	6,4E-05	5,1E-05	2,5E-04	1,1E-03
M5	6,9E-04	4,6E-07	8,7E-07	1,4E-04	6,4E-05	1,0E+00	2,1E-05	3,5E-04	9,9E-05
M6	1,5E-05	1,4E-06	1,0E-06	8,1E-04	5,1E-05	2,1E-05	1,0E+00	3,9E-07	6,7E-06
M7	2,8E-05	4,1E-05	1,3E-06	2,5E-07	2,5E-04	3,5E-04	3,9E-07	1,0E+00	3,7E-04
S	1,4E-04	3,2E-04	2,7E-05	4,3E-05	1,1E-03	9,9E-05	6,7E-06	3,7E-04	1,0E+00

Quadratic function – temperature-independent system

Concentrations of the participants.

ID	Concentration, µM
M1	2.52
M2	2.75
M3	1.70
M4	6.92
M5	5.33
M6	7.11
S	0.03
C1	2.77
C2	0.14
C3	1.73
C4	1.75
C5	4.86
C6	0.00
C7	4.25
C8	6.80

Mutual affinity of the participants. Approximate dissociation constant K_d (25 °C) in M.

	I	M1	M2	M3	M4	M5	M6	S	C1	C2	C3	C4	C5	C6	C7	C8
**I**	1.0E+00	7.6E-10	6.6E-07	1.7E-04	3.0E-11	2.4E-04	3.0E-08	3.1E-04	9.6E-09	1.0E+00	1.0E+00	1.0E+00	1.0E+00	1.0E+00	1.0E+00	1.0E+00
**M1**	7.6E-10	1.0E+00	3.0E-05	3.0E-11	3.4E-11	3.1E-08	5.4E-09	1.3E-06	1.0E+00	1.4E-07	1.0E+00	1.0E+00	1.0E+00	1.0E+00	1.0E+00	1.0E+00
**M2**	6.6E-07	3.0E-05	1.0E+00	3.7E-08	1.3E-05	3.8E-08	1.1E-07	2.4E-04	1.0E+00	1.0E+00	4.2E-09	1.0E+00	1.0E+00	1.0E+00	1.0E+00	1.0E+00
**M3**	1.7E-04	3.0E-11	3.7E-08	1.0E+00	6.9E-05	6.2E-06	3.0E-04	2.2E-06	1.0E+00	1.0E+00	1.0E+00	3.6E-11	1.0E+00	1.0E+00	1.0E+00	1.0E+00
**M4**	3.0E-11	3.4E-11	1.3E-05	6.9E-05	1.0E+00	4.6E-07	2.8E-08	9.5E-05	1.0E+00	1.0E+00	1.0E+00	1.0E+00	1.1E-09	1.0E+00	1.0E+00	1.0E+00
**M5**	2.4E-04	3.1E-08	3.8E-08	6.2E-06	4.6E-07	1.0E+00	5.5E-10	8.5E-06	1.0E+00	1.0E+00	1.0E+00	1.0E+00	1.0E+00	2.4E-05	1.0E+00	1.0E+00
**M6**	3.0E-08	5.4E-09	1.1E-07	3.0E-04	2.8E-08	5.5E-10	1.0E+00	9.2E-09	1.0E+00	1.0E+00	1.0E+00	1.0E+00	1.0E+00	1.0E+00	2.3E-10	1.0E+00
**S**	3.1E-04	1.3E-06	2.4E-04	2.2E-06	9.5E-05	8.5E-06	9.2E-09	1.0E+00	1.0E+00	1.0E+00	1.0E+00	1.0E+00	1.0E+00	1.0E+00	1.0E+00	5.5E-05
**C1**	9.6E-09	1.0E+00	1.0E+00	1.0E+00	1.0E+00	1.0E+00	1.0E+00	1.0E+00	1.0E+00	1.0E+00	1.0E+00	1.0E+00	1.0E+00	1.0E+00	1.0E+00	1.0E+00
**C2**	1.0E+00	1.4E-07	1.0E+00	1.0E+00	1.0E+00	1.0E+00	1.0E+00	1.0E+00	1.0E+00	1.0E+00	1.0E+00	1.0E+00	1.0E+00	1.0E+00	1.0E+00	1.0E+00
**C3**	1.0E+00	1.0E+00	4.2E-09	1.0E+00	1.0E+00	1.0E+00	1.0E+00	1.0E+00	1.0E+00	1.0E+00	1.0E+00	1.0E+00	1.0E+00	1.0E+00	1.0E+00	1.0E+00
**C4**	1.0E+00	1.0E+00	1.0E+00	3.6E-11	1.0E+00	1.0E+00	1.0E+00	1.0E+00	1.0E+00	1.0E+00	1.0E+00	1.0E+00	1.0E+00	1.0E+00	1.0E+00	1.0E+00
**C5**	1.0E+00	1.0E+00	1.0E+00	1.0E+00	1.1E-09	1.0E+00	1.0E+00	1.0E+00	1.0E+00	1.0E+00	1.0E+00	1.0E+00	1.0E+00	1.0E+00	1.0E+00	1.0E+00
**C6**	1.0E+00	1.0E+00	1.0E+00	1.0E+00	1.0E+00	2.4E-05	1.0E+00	1.0E+00	1.0E+00	1.0E+00	1.0E+00	1.0E+00	1.0E+00	1.0E+00	1.0E+00	1.0E+00
**C7**	1.0E+00	1.0E+00	1.0E+00	1.0E+00	1.0E+00	1.0E+00	2.3E-10	1.0E+00	1.0E+00	1.0E+00	1.0E+00	1.0E+00	1.0E+00	1.0E+00	1.0E+00	1.0E+00
**C8**	1.0E+00	1.0E+00	1.0E+00	1.0E+00	1.0E+00	1.0E+00	1.0E+00	5.5E-05	1.0E+00	1.0E+00	1.0E+00	1.0E+00	1.0E+00	1.0E+00	1.0E+00	1.0E+00

Mutual affinity of the participants. Approximate dissociation constant K_d (50 °C) in M.

	I	M1	M2	M3	M4	M5	M6	S	C1	C2	C3	C4	C5	C6	C7	C8
**I**	1,0E+00	1,1E-06	5,9E-05	1,3E-03	1,5E-07	1,6E-03	9,7E-06	1,8E-03	1,9E-04	1,0E+00	1,0E+00	1,0E+00	1,0E+00	1,0E+00	1,0E+00	1,0E+00
**M1**	1,1E-06	1,0E+00	5,1E-04	1,5E-07	1,6E-07	9,8E-06	3,5E-06	8,6E-05	1,0E+00	7,0E-04	1,0E+00	1,0E+00	1,0E+00	1,0E+00	1,0E+00	1,0E+00
**M2**	5,9E-05	5,1E-04	1,0E+00	1,1E-05	3,1E-04	1,1E-05	2,1E-05	1,6E-03	1,0E+00	1,0E+00	1,2E-04	1,0E+00	1,0E+00	1,0E+00	1,0E+00	1,0E+00
**M3**	1,3E-03	1,5E-07	1,1E-05	1,0E+00	8,2E-04	2,1E-04	1,8E-03	1,2E-04	1,0E+00	1,0E+00	1,0E+00	7,3E-06	1,0E+00	1,0E+00	1,0E+00	1,0E+00
**M4**	1,5E-07	1,6E-07	3,1E-04	8,2E-04	1,0E+00	4,8E-05	9,2E-06	9,7E-04	1,0E+00	1,0E+00	1,0E+00	1,0E+00	5,9E-05	1,0E+00	1,0E+00	1,0E+00
**M5**	1,6E-03	9,8E-06	1,1E-05	2,1E-04	4,8E-05	1,0E+00	8,8E-07	2,5E-04	1,0E+00	1,0E+00	1,0E+00	1,0E+00	1,0E+00	4,6E-03	1,0E+00	1,0E+00
**M6**	9,7E-06	3,5E-06	2,1E-05	1,8E-03	9,2E-06	8,8E-07	1,0E+00	4,8E-06	1,0E+00	1,0E+00	1,0E+00	1,0E+00	1,0E+00	1,0E+00	2,3E-05	1,0E+00
**S**	1,8E-03	8,6E-05	1,6E-03	1,2E-04	9,7E-04	2,5E-04	4,8E-06	1,0E+00	1,0E+00	1,0E+00	1,0E+00	1,0E+00	1,0E+00	1,0E+00	1,0E+00	5,6E-03
**C1**	1,9E-04	1,0E+00	1,0E+00	1,0E+00	1,0E+00	1,0E+00	1,0E+00	1,0E+00	1,0E+00	1,0E+00	1,0E+00	1,0E+00	1,0E+00	1,0E+00	1,0E+00	1,0E+00
**C2**	1,0E+00	7,0E-04	1,0E+00	1,0E+00	1,0E+00	1,0E+00	1,0E+00	1,0E+00	1,0E+00	1,0E+00	1,0E+00	1,0E+00	1,0E+00	1,0E+00	1,0E+00	1,0E+00
**C3**	1,0E+00	1,0E+00	1,2E-04	1,0E+00	1,0E+00	1,0E+00	1,0E+00	1,0E+00	1,0E+00	1,0E+00	1,0E+00	1,0E+00	1,0E+00	1,0E+00	1,0E+00	1,0E+00
**C4**	1,0E+00	1,0E+00	1,0E+00	7,3E-06	1,0E+00	1,0E+00	1,0E+00	1,0E+00	1,0E+00	1,0E+00	1,0E+00	1,0E+00	1,0E+00	1,0E+00	1,0E+00	1,0E+00
**C5**	1,0E+00	1,0E+00	1,0E+00	1,0E+00	5,9E-05	1,0E+00	1,0E+00	1,0E+00	1,0E+00	1,0E+00	1,0E+00	1,0E+00	1,0E+00	1,0E+00	1,0E+00	1,0E+00
**C6**	1,0E+00	1,0E+00	1,0E+00	1,0E+00	1,0E+00	4,6E-03	1,0E+00	1,0E+00	1,0E+00	1,0E+00	1,0E+00	1,0E+00	1,0E+00	1,0E+00	1,0E+00	1,0E+00
**C7**	1,0E+00	1,0E+00	1,0E+00	1,0E+00	1,0E+00	1,0E+00	2,3E-05	1,0E+00	1,0E+00	1,0E+00	1,0E+00	1,0E+00	1,0E+00	1,0E+00	1,0E+00	1,0E+00
**C8**	1,0E+00	1,0E+00	1,0E+00	1,0E+00	1,0E+00	1,0E+00	1,0E+00	5,6E-03	1,0E+00	1,0E+00	1,0E+00	1,0E+00	1,0E+00	1,0E+00	1,0E+00	1,0E+00

Quadratic function – control system without compensatory participants

Concentrations of the participants.

ID	Concentration, µM
M1	5.44
M2	2.29
M3	1.50
M4	7.66
M5	5.69
M6	7.45
M7	0.75
S	0.03

Mutual affinity of the participants. Approximate dissociation constant K_d (25 °C) in M.

	I	M1	M2	M3	M4	M5	M6	M7	S
I	1.0E+00	3.0E-11	1.2E-05	2.8E-06	3.0E-11	6.0E-06	2.0E-05	9.8E-08	3.6E-06
M1	3.0E-11	1.0E+00	7.2E-08	1.1E-06	1.5E-05	1.4E-08	3.5E-11	1.1E-04	2.4E-07
M2	1.2E-05	7.2E-08	1.0E+00	8.4E-08	4.3E-06	1.4E-06	1.1E-04	1.9E-10	2.4E-04
M3	2.8E-06	1.1E-06	8.4E-08	1.0E+00	1.4E-06	1.0E-06	3.7E-05	8.1E-05	1.9E-04
M4	3.0E-11	1.5E-05	4.3E-06	1.4E-06	1.0E+00	1.1E-08	3.1E-11	2.2E-09	1.4E-06
M5	6.0E-06	1.4E-08	1.4E-06	1.0E-06	1.1E-08	1.0E+00	3.6E-09	1.0E-07	2.2E-08
M6	2.0E-05	3.5E-11	1.1E-04	3.7E-05	3.1E-11	3.6E-09	1.0E+00	2.9E-04	4.4E-05
M7	9.8E-08	1.1E-04	1.9E-10	8.1E-05	2.2E-09	1.0E-07	2.9E-04	1.0E+00	3.7E-05
S	3.6E-06	2.4E-07	2.4E-04	1.9E-04	1.4E-06	2.2E-08	4.4E-05	3.7E-05	1.0E+00

Mutual affinity of the participants. Approximate dissociation constant K_d (50 °C) in M.

	I	M1	M2	M3	M4	M5	M6	M7	S
I	1,0E+00	1,5E-07	3,1E-04	1,3E-04	1,5E-07	2,1E-04	4,1E-04	1,9E-05	1,6E-04
M1	1,5E-07	1,0E+00	1,6E-05	7,7E-05	3,5E-04	6,1E-06	1,6E-07	1,1E-03	3,3E-05
M2	3,1E-04	1,6E-05	1,0E+00	1,8E-05	1,7E-04	9,0E-05	1,0E-03	4,5E-07	1,6E-03
M3	1,3E-04	7,7E-05	1,8E-05	1,0E+00	9,0E-05	7,6E-05	5,7E-04	8,9E-04	1,4E-03
M4	1,5E-07	3,5E-04	1,7E-04	9,0E-05	1,0E+00	5,2E-06	1,5E-07	2,0E-06	9,0E-05
M5	2,1E-04	6,1E-06	9,0E-05	7,6E-05	5,2E-06	1,0E+00	2,7E-06	2,0E-05	8,0E-06
M6	4,1E-04	1,6E-07	1,0E-03	5,7E-04	1,5E-07	2,7E-06	1,0E+00	1,8E-03	6,3E-04
M7	1,9E-05	1,1E-03	4,5E-07	8,9E-04	2,0E-06	2,0E-05	1,8E-03	1,0E+00	5,7E-04
S	1,6E-04	3,3E-05	1,6E-03	1,4E-03	9,0E-05	8,0E-06	6,3E-04	5,7E-04	1,0E+00

Cubic function – temperature-independent system

Concentrations of the participants.

ID	Concentration, µM
M1	0.66
M2	10.00
M3	4.45
M4	3.53
M5	4.33
M6	7.02
M7	9.55
S	0.03
C1	1.42
C2	10.00
C3	9.21
C4	4.20
C5	4.91
C6	1.57
C7	3.06
C8	2.50

Mutual affinity of the participants. Approximate dissociation constant K_d (25 °C) in M.

	I	M1	M2	M3	M4	M5	M6	M7	S	C1	C2	C3	C4	C5	C6	C7	C8
I	1.0E+00	6.5E-10	9.5E-06	2.5E-04	9.7E-11	3.1E-11	2.9E-04	5.4E-07	3.0E-04	7.6E-10	1.0E+00	1.0E+00	1.0E+00	1.0E+00	1.0E+00	1.0E+00	1.0E+00
M1	6.5E-10	1.0E+00	1.5E-04	2.9E-10	7.2E-11	3.3E-07	7.1E-11	1.2E-06	3.1E-11	1.0E+00	1.1E-10	1.0E+00	1.0E+00	1.0E+00	1.0E+00	1.0E+00	1.0E+00
M2	9.5E-06	1.5E-04	1.0E+00	3.0E-07	8.3E-08	2.1E-04	1.1E-09	7.5E-05	1.2E-05	1.0E+00	1.0E+00	2.6E-10	1.0E+00	1.0E+00	1.0E+00	1.0E+00	1.0E+00
M3	2.5E-04	2.9E-10	3.0E-07	1.0E+00	1.6E-06	1.3E-06	2.4E-06	4.1E-11	8.3E-07	1.0E+00	1.0E+00	1.0E+00	1.0E+00	1.0E+00	1.0E+00	1.0E+00	1.0E+00
M4	9.7E-11	7.2E-11	8.3E-08	1.6E-06	1.0E+00	2.0E-06	2.5E-04	1.8E-09	2.2E-06	1.0E+00	1.0E+00	1.0E+00	6.5E-10	1.0E+00	1.0E+00	1.0E+00	1.0E+00
M5	3.1E-11	3.3E-07	2.1E-04	1.3E-06	2.0E-06	1.0E+00	5.5E-05	1.2E-05	3.2E-07	1.0E+00	1.0E+00	1.0E+00	1.0E+00	2.3E-09	1.0E+00	1.0E+00	1.0E+00
M6	2.9E-04	7.1E-11	1.1E-09	2.4E-06	2.5E-04	5.5E-05	1.0E+00	8.6E-08	2.2E-05	1.0E+00	1.0E+00	1.0E+00	1.0E+00	1.0E+00	3.7E-11	1.0E+00	1.0E+00
M7	5.4E-07	1.2E-06	7.5E-05	4.1E-11	1.8E-09	1.2E-05	8.6E-08	1.0E+00	5.8E-07	1.0E+00	1.0E+00	1.0E+00	1.0E+00	1.0E+00	1.0E+00	5.4E-09	1.0E+00
S	3.0E-04	3.1E-11	1.2E-05	8.3E-07	2.2E-06	3.2E-07	2.2E-05	5.8E-07	1.0E+00	1.0E+00	1.0E+00	1.0E+00	1.0E+00	1.0E+00	1.0E+00	1.0E+00	1.5E-04
C1	7.6E-10	1.0E+00	1.0E+00	1.0E+00	1.0E+00	1.0E+00	1.0E+00	1.0E+00	1.0E+00	1.0E+00	1.0E+00	1.0E+00	1.0E+00	1.0E+00	1.0E+00	1.0E+00	1.0E+00
C2	1.0E+00	1.1E-10	1.0E+00	1.0E+00	1.0E+00	1.0E+00	1.0E+00	1.0E+00	1.0E+00	1.0E+00	1.0E+00	1.0E+00	1.0E+00	1.0E+00	1.0E+00	1.0E+00	1.0E+00
C3	1.0E+00	1.0E+00	2.6E-10	1.0E+00	1.0E+00	1.0E+00	1.0E+00	1.0E+00	1.0E+00	1.0E+00	1.0E+00	1.0E+00	1.0E+00	1.0E+00	1.0E+00	1.0E+00	1.0E+00
C4	1.0E+00	1.0E+00	1.0E+00	1.0E+00	6.5E-10	1.0E+00	1.0E+00	1.0E+00	1.0E+00	1.0E+00	1.0E+00	1.0E+00	1.0E+00	1.0E+00	1.0E+00	1.0E+00	1.0E+00
C5	1.0E+00	1.0E+00	1.0E+00	1.0E+00	1.0E+00	2.3E-09	1.0E+00	1.0E+00	1.0E+00	1.0E+00	1.0E+00	1.0E+00	1.0E+00	1.0E+00	1.0E+00	1.0E+00	1.0E+00
C6	1.0E+00	1.0E+00	1.0E+00	1.0E+00	1.0E+00	1.0E+00	3.7E-11	1.0E+00	1.0E+00	1.0E+00	1.0E+00	1.0E+00	1.0E+00	1.0E+00	1.0E+00	1.0E+00	1.0E+00
C7	1.0E+00	1.0E+00	1.0E+00	1.0E+00	1.0E+00	1.0E+00	1.0E+00	5.4E-09	1.0E+00	1.0E+00	1.0E+00	1.0E+00	1.0E+00	1.0E+00	1.0E+00	1.0E+00	1.0E+00
C8	1.0E+00	1.0E+00	1.0E+00	1.0E+00	1.0E+00	1.0E+00	1.0E+00	1.0E+00	1.5E-04	1.0E+00	1.0E+00	1.0E+00	1.0E+00	1.0E+00	1.0E+00	1.0E+00	1.0E+00

Mutual affinity of the participants. Approximate dissociation constant K_d (50 °C) in M.

	I	M1	M2	M3	M4	M5	M6	M7	S	C1	C2	C3	C4	C5	C6	C7	C8
I	1,0E+00	9,7E-07	2,7E-04	1,7E-03	3,0E-07	1,5E-07	1,8E-03	5,2E-05	1,8E-03	4,7E-05	1,0E+00	1,0E+00	1,0E+00	1,0E+00	1,0E+00	1,0E+00	1,0E+00
M1	9,7E-07	1,0E+00	1,3E-03	5,9E-07	2,5E-07	4,0E-05	2,5E-07	8,2E-05	1,5E-07	1,0E+00	1,5E-05	1,0E+00	1,0E+00	1,0E+00	1,0E+00	1,0E+00	1,0E+00
M2	2,7E-04	1,3E-03	1,0E+00	3,7E-05	1,8E-05	1,5E-03	1,4E-06	8,5E-04	3,1E-04	1,0E+00	1,0E+00	2,5E-05	1,0E+00	1,0E+00	1,0E+00	1,0E+00	1,0E+00
M3	1,7E-03	5,9E-07	3,7E-05	1,0E+00	9,7E-05	8,8E-05	1,2E-04	1,8E-07	6,7E-05	1,0E+00	1,0E+00	1,0E+00	1,0E+00	1,0E+00	1,0E+00	1,0E+00	1,0E+00
M4	3,0E-07	2,5E-07	1,8E-05	9,7E-05	1,0E+00	1,1E-04	1,6E-03	1,8E-06	1,2E-04	1,0E+00	1,0E+00	1,0E+00	4,3E-05	1,0E+00	1,0E+00	1,0E+00	1,0E+00
M5	1,5E-07	4,0E-05	1,5E-03	8,8E-05	1,1E-04	1,0E+00	7,2E-04	3,1E-04	3,9E-05	1,0E+00	1,0E+00	1,0E+00	1,0E+00	8,7E-05	1,0E+00	1,0E+00	1,0E+00
M6	1,8E-03	2,5E-07	1,4E-06	1,2E-04	1,6E-03	7,2E-04	1,0E+00	1,8E-05	4,3E-04	1,0E+00	1,0E+00	1,0E+00	1,0E+00	1,0E+00	7,4E-06	1,0E+00	1,0E+00
M7	5,2E-05	8,2E-05	8,5E-04	1,8E-07	1,8E-06	3,1E-04	1,8E-05	1,0E+00	5,5E-05	1,0E+00	1,0E+00	1,0E+00	1,0E+00	1,0E+00	1,0E+00	1,4E-04	1,0E+00
S	1,8E-03	1,5E-07	3,1E-04	6,7E-05	1,2E-04	3,9E-05	4,3E-04	5,5E-05	1,0E+00	1,0E+00	1,0E+00	1,0E+00	1,0E+00	1,0E+00	1,0E+00	1,0E+00	7,0E-03
C1	4,7E-05	1,0E+00	1,0E+00	1,0E+00	1,0E+00	1,0E+00	1,0E+00	1,0E+00	1,0E+00	1,0E+00	1,0E+00	1,0E+00	1,0E+00	1,0E+00	1,0E+00	1,0E+00	1,0E+00
C2	1,0E+00	1,5E-05	1,0E+00	1,0E+00	1,0E+00	1,0E+00	1,0E+00	1,0E+00	1,0E+00	1,0E+00	1,0E+00	1,0E+00	1,0E+00	1,0E+00	1,0E+00	1,0E+00	1,0E+00
C3	1,0E+00	1,0E+00	2,5E-05	1,0E+00	1,0E+00	1,0E+00	1,0E+00	1,0E+00	1,0E+00	1,0E+00	1,0E+00	1,0E+00	1,0E+00	1,0E+00	1,0E+00	1,0E+00	1,0E+00
C4	1,0E+00	1,0E+00	1,0E+00	1,0E+00	4,3E-05	1,0E+00	1,0E+00	1,0E+00	1,0E+00	1,0E+00	1,0E+00	1,0E+00	1,0E+00	1,0E+00	1,0E+00	1,0E+00	1,0E+00
C5	1,0E+00	1,0E+00	1,0E+00	1,0E+00	1,0E+00	8,7E-05	1,0E+00	1,0E+00	1,0E+00	1,0E+00	1,0E+00	1,0E+00	1,0E+00	1,0E+00	1,0E+00	1,0E+00	1,0E+00
C6	1,0E+00	1,0E+00	1,0E+00	1,0E+00	1,0E+00	1,0E+00	7,4E-06	1,0E+00	1,0E+00	1,0E+00	1,0E+00	1,0E+00	1,0E+00	1,0E+00	1,0E+00	1,0E+00	1,0E+00
C7	1,0E+00	1,0E+00	1,0E+00	1,0E+00	1,0E+00	1,0E+00	1,0E+00	1,4E-04	1,0E+00	1,0E+00	1,0E+00	1,0E+00	1,0E+00	1,0E+00	1,0E+00	1,0E+00	1,0E+00
C8	1,0E+00	1,0E+00	1,0E+00	1,0E+00	1,0E+00	1,0E+00	1,0E+00	1,0E+00	7,0E-03	1,0E+00	1,0E+00	1,0E+00	1,0E+00	1,0E+00	1,0E+00	1,0E+00	1,0E+00

Cubic function – control system without compensatory participants

Concentrations of the participants.

ID	Concentration, µM
M1	0.73
M2	3.75
M3	4.66
M4	2.71
M5	2.58
M6	5.15
M7	9.79
S	0.03

Mutual affinity of the participants. Approximate dissociation constant K_d (25 °C) in M.

	I	M1	M2	M3	M4	M5	M6	M7	S
**I**	1.0E+00	2.9E-10	2.9E-04	1.3E-06	3.0E-11	8.2E-11	4.2E-10	2.0E-07	8.1E-06
**M1**	2.9E-10	1.0E+00	2.4E-04	1.7E-10	8.9E-11	3.3E-05	7.6E-11	1.3E-07	1.5E-10
**M2**	2.9E-04	2.4E-04	1.0E+00	5.7E-06	1.8E-10	1.6E-09	1.5E-09	3.2E-07	3.5E-06
**M3**	1.3E-06	1.7E-10	5.7E-06	1.0E+00	7.5E-06	2.0E-06	2.8E-04	3.0E-11	1.1E-08
**M4**	3.0E-11	8.9E-11	1.8E-10	7.5E-06	1.0E+00	6.4E-06	2.9E-04	4.8E-09	2.2E-08
**M5**	8.2E-11	3.3E-05	1.6E-09	2.0E-06	6.4E-06	1.0E+00	4.2E-06	5.7E-09	5.0E-08
**M6**	4.2E-10	7.6E-11	1.5E-09	2.8E-04	2.9E-04	4.2E-06	1.0E+00	5.4E-08	4.1E-07
**M7**	2.0E-07	1.3E-07	3.2E-07	3.0E-11	4.8E-09	5.7E-09	5.4E-08	1.0E+00	1.4E-07
**S**	8.1E-06	1.5E-10	3.5E-06	1.1E-08	2.2E-08	5.0E-08	4.1E-07	1.4E-07	1.0E+00

Mutual affinity of the participants. Approximate dissociation constant K_d (50 °C) in M.

	I	M1	M2	M3	M4	M5	M6	M7	S
**I**	1,0E+00	5,9E-07	1,8E-03	8,8E-05	1,5E-07	2,7E-07	7,4E-07	3,0E-05	2,5E-04
**M1**	5,9E-07	1,0E+00	1,6E-03	4,2E-07	2,9E-07	5,4E-04	2,6E-07	2,3E-05	3,9E-07
**M2**	1,8E-03	1,6E-03	1,0E+00	2,0E-04	4,3E-07	1,6E-06	1,6E-06	3,8E-05	1,5E-04
**M3**	8,8E-05	4,2E-07	2,0E-04	1,0E+00	2,3E-04	1,1E-04	1,8E-03	1,5E-07	5,2E-06
**M4**	1,5E-07	2,9E-07	4,3E-07	2,3E-04	1,0E+00	2,2E-04	1,8E-03	3,2E-06	8,0E-06
**M5**	2,7E-07	5,4E-04	1,6E-06	1,1E-04	2,2E-04	1,0E+00	1,7E-04	3,6E-06	1,3E-05
**M6**	7,4E-07	2,6E-07	1,6E-06	1,8E-03	1,8E-03	1,7E-04	1,0E+00	1,4E-05	4,5E-05
**M7**	3,0E-05	2,3E-05	3,8E-05	1,5E-07	3,2E-06	3,6E-06	1,4E-05	1,0E+00	2,4E-05
**S**	2,5E-04	3,9E-07	1,5E-04	5,2E-06	8,0E-06	1,3E-05	4,5E-05	2,4E-05	1,0E+00

Algebraic systems in tandem with external systems

For all systems the following optimization parameters were used:

**Concentration lower bound, nM**	1
**Concentration upper bound, nM**	1000
**Concentration of output species, nM**	30
**Starting number of nodes**	6
**Gibbs energy at 25 °C lower bound, kJ/mol**	-60
**Gibbs energy at 25 °C upper bound, kJ/mol**	-20
**Pruning concentration threshold, nM**	5
**Pruning gibbs energy threshold, kJ/mol**	-25

Line function

Sequences and concentrations of the participants.

ID	Sequence, 5'-3'	Concentration, μM
M1	AAACTAATGGATGATGACTA	0.91
M2	TGGGCATCTTCTATTAGGTT	1.00
M3	ATTAAAAGGTCGAACGGCAC	0.99
M4	GTGGCGTTGGACATTTGGAT	0.36
Out	ATCCAAATGGAAGACGCCAA	0.03

Mutual affinity of the participants. Approximate dissociation constant K_d (25 °C) in M.

	M1	M2	M3	M4	Out
**M1**	4.9E-03	1.0E-07	1.2E-03	8.0E-04	2.2E-04
**M2**	1.0E-07	4.7E-04	4.5E-04	1.4E-03	1.1E-06
**M3**	1.2E-03	4.5E-04	5.3E-05	3.4E-11	8.0E-05
**M4**	8.0E-04	1.4E-03	3.4E-11	4.7E-03	2.8E-11
**Out**	2.2E-04	1.1E-06	8.0E-05	2.8E-11	5.5E-04

Mutual affinity of the participants. Approximate dissociation constant K_d (50 °C) in M.

	M1	M2	M3	M4	Out
**M1**	6.7E-03	2.2E-04	4.0E-03	3.6E-03	1.9E-03
**M2**	2.2E-04	4.9E-03	3.0E-03	6.3E-03	1.5E-04
**M3**	4.0E-03	3.0E-03	6.7E-04	3.4E-07	1.6E-03
**M4**	3.6E-03	6.3E-03	3.4E-07	9.9E-03	6.3E-07
**Out**	1.9E-03	1.5E-04	1.6E-03	6.3E-07	2.4E-03

Quadratic function

Sequences and concentrations of the participants.

ID	Sequence, 5'-3'	Concentration, µM
M1	TTGAGTGCCGACTTTTGAAA	1.00
M2	ATGCAAAAGTCTCACCACAA	0.45
M3	TTGTTGTGATCCTTTTGCCG	0.18
M4	ATGCAACACTAACACCACCA	0.89
M5	TTGGGGTGTTACATTTGCAT	0.75
Out	ATCCAAATGGAAGACGCCAA	0.03

Mutual affinity of the participants. Approximate dissociation constant K_d (25 °C) in M.

	M1	M2	M3	M4	M5	Out
**M1**	5.3E-04	2.9E-09	4.1E-03	2.7E-04	1.8E-03	4.2E-04
**M2**	2.9E-09	7.6E-05	3.7E-11	1.1E-04	4.0E-11	3.9E-04
**M3**	4.1E-03	3.7E-11	2.3E-04	2.0E-06	3.2E-04	5.6E-05
**M4**	2.7E-04	1.1E-04	2.0E-06	1.7E-04	6.3E-11	2.1E-03
**M5**	1.8E-03	4.0E-11	3.2E-04	6.3E-11	8.1E-05	1.4E-09
**Out**	4.2E-04	3.9E-04	5.6E-05	2.1E-03	1.4E-09	5.5E-04

Mutual affinity of the participants. Approximate dissociation constant K_d (50 °C) in M.

	M1	M2	M3	M4	M5	Out
**M1**	5.8E-03	2.6E-05	6.5E-03	1.5E-03	5.0E-03	4.1E-03
**M2**	2.6E-05	1.6E-03	4.4E-06	1.4E-03	9.9E-07	4.2E-03
**M3**	6.5E-03	4.4E-06	5.1E-03	3.6E-04	2.6E-03	2.0E-03
**M4**	1.5E-03	1.4E-03	3.6E-04	1.5E-03	3.0E-06	5.9E-03
**M5**	5.0E-03	9.9E-07	2.6E-03	3.0E-06	1.3E-03	2.4E-06
**Out**	4.1E-03	4.2E-03	2.0E-03	5.9E-03	2.4E-06	2.4E-03

Cubic function

Sequences and concentrations of the participants.

ID	Sequence, 5'-3'	Concentration, µM
M1	TAAGAAAACGAATACAGCAA	0.32
M2	TATGCGTCTTGAATATCTGG	0.03
M3	TTCCAACTCGAAATCTGCAA	0.61
M4	TTGCTGTCTTCGATTTGGAA	0.29
Out	ATCCAAATGGAAGACGCCAA	0.03

Mutual affinity of the participants. Approximate dissociation constant K_d (25 °C) in M.

	M1	M2	M3	M4	Out
**M1**	4.1E-04	4.1E-05	1.3E-04	3.9E-11	8.9E-04
**M2**	4.1E-05	1.1E-03	2.2E-04	1.5E-03	3.8E-08
**M3**	1.3E-04	2.2E-04	5.0E-05	1.8E-11	9.0E-04
**M4**	3.9E-11	1.5E-03	1.8E-11	4.2E-04	2.5E-11
**Out**	8.9E-04	3.8E-08	9.0E-04	2.5E-11	5.5E-04

Mutual affinity of the participants. Approximate dissociation constant K_d (50 °C) in M.

	M1	M2	M3	M4	Out
**M1**	2.9E-03	6.3E-04	1.3E-03	3.7E-07	5.9E-03
**M2**	6.3E-04	6.2E-03	2.3E-03	5.8E-03	1.6E-05
**M3**	1.3E-03	2.3E-03	9.5E-04	1.5E-06	3.5E-03
**M4**	3.7E-07	5.8E-03	1.5E-06	2.7E-03	6.5E-07
**Out**	5.9E-03	1.6E-05	3.5E-03	6.5E-07	2.4E-03

Sine function

Sequences and concentrations of the participants.

ID	Sequence, 5'-3'	Concentration, µM
M1	TACCACATCAATCACGTCCC	1.00
M2	TGGGGTTCTTTCATTAGGAT	0.15
M3	ACCCTCATGCAACACGAATC	0.35
M4	TGGGCGTGTTCAATGTGGGT	0.75
Out	ATCCAAATGGAAGACGCCAA	0.03

Mutual affinity of the participants. Approximate dissociation constant K_d (25 °C) in M.

	M1	M2	M3	M4	Out
M1	1.1E-04	3.6E-05	3.3E-04	1.5E-11	2.5E-05
M2	3.6E-05	7.1E-03	1.4E-05	1.7E-03	2.8E-07
M3	3.3E-04	1.4E-05	1.6E-04	2.2E-11	1.1E-03
M4	1.5E-11	1.7E-03	2.2E-11	9.6E-04	6.9E-10
Out	2.5E-05	2.8E-07	1.1E-03	6.9E-10	5.5E-04

Mutual affinity of the participants. Approximate dissociation constant K_d (50 °C) in M.

	M1	M2	M3	M4	Out
M1	7.5E-04	6.0E-04	2.2E-03	6.8E-06	1.1E-03
M2	6.0E-04	1.1E-02	5.0E-04	6.6E-03	2.6E-04
M3	2.2E-03	5.0E-04	1.7E-03	1.9E-06	6.2E-03
M4	6.8E-06	6.6E-03	1.9E-06	6.0E-03	1.2E-06
Out	1.1E-03	2.6E-04	6.2E-03	1.2E-06	2.4E-03

Cubic function (ionic strength)

Sequences and concentrations of the participants.

ID	Sequence, 5'-3'	Concentration, µM
M1	CTTGAGTCCTCCATTGGGTT	0.91
M2	ATCCAGAAGTAAGATGCTAA	0.57
M3	TTAGGGTCTTACTTTTGGAG	0.23
Out	ATCCAAATGGAAGACGCCAA	0.04

Mutual affinity of the participants. Approximate dissociation constant K_d (25 °C) in M.

	M1	M2	M3	Out
**M1**	4.6E-04	1.3E-03	3.5E-04	1.1E-06
**M2**	1.3E-03	2.0E-03	1.8E-09	2.5E-03
**M3**	3.5E-04	1.8E-09	6.5E-03	2.5E-09
**Out**	1.1E-06	2.5E-03	2.5E-09	5.5E-04

Mutual affinity of the participants. Approximate dissociation constant K_d (50 °C) in M.

	M1	M2	M3	Out
**M1**	1.6E-03	2.8E-03	1.1E-03	5.3E-05
**M2**	2.8E-03	3.0E-03	5.8E-07	4.0E-03
**M3**	1.1E-03	5.8E-07	8.4E-03	2.6E-07
**Out**	5.3E-05	4.0E-03	2.6E-07	1.4E-03

Supplementary references

1. Stites, W. E. Protein−Protein Interactions: Interface Structure, Binding Thermodynamics, and Mutational Analysis. *Chem. Rev.* **97**, 1233–1250 (1997).

2. Kelley, R. F. & O'Connell, M. P. Thermodynamic analysis of an antibody functional epitope. *Biochemistry* **32**, 6828–6835 (1993).

3. Stan, R. C. *et al.* Febrile temperatures increase in vitro antibody affinity for malarial and dengue antigens. *PLoS Negl. Trop. Dis.* **13**, e0007239 (2019).

4. Dirks, R. M., Bois, J. S., Schaeffer, J. M., Winfree, E. & Pierce, N. A. Thermodynamic Analysis of Interacting Nucleic Acid Strands. *SIAM Rev.* **49**, 65–88 (2007).

5. van Dorp, M. G. A., Berger, F. & Carlon, E. Computing equilibrium concentrations for large heterodimerization networks. *Phys. Rev. E* **84**, 036114 (2011).
