# Supplementary material for: Enabling molecular signaling with temperature and ionic-strength independence or programmable dependence
Source: Nat Commun. 2026 Aug 7;17:8895. doi: 10.1038/s41467-026-76207-x (PMC13503822; doi:10.1038/s41467-026-76207-x)
Supplement: Supplementary file 3 [file 41467_2026_76207_MOESM3_ESM.pdf]

nature portfolio

Peer Review File

Enabling molecular signaling with temperature and ionic-strength independence or programmable dependence

Corresponding Author: Dr Maxim Nikitin

This file contains all reviewer reports in order by version, followed by all author rebuttals in order by version.

Version 0:

Reviewer comments:

Reviewer #1

(Remarks to the Author)
The manuscript "Topology of molecular networks offers signaling insusceptibility to temperature and ionic strength changes" explores a concept of temperature stabilisation of signal processing biochemical modules using buffering substances with temperature-calibrated affinities. It describes a diverse set of in-silico examples and an experimental verification of the simplest module, the repeater, or "yes" gate. The modules consist of several substances (oligonucleotides) that reversibly dimerise according to the law of mass action.

The conceptual design is similar to commonly used chemically-buffered modules, where a weakly bound substance is released or sequestered when the external conditions change, making the module itself less susceptible to those changes. Here the external conditions are defined as the temperature and ion concentrations, so the binding affinities of corresponding compensating substances have to be more sensitive to those changes than those of the principal components of the modules. To the best of my knowledge, this is new. The manuscript clearly and convincingly demonstrates that such a principle works and it is indeed possible to design modules that maintain their performance in the range of temperatures where the dissociation constants change by orders of magnitude. And the experimental verification complements the modeling and confirms the main conclusions.

However, the text begs a question of whether anything like this happens in any naturally-occurring biochemical system. Several examples of organisms (and organs) that function in a range of temperatures are referred to in the Introduction. Yet at the same time there are lots of examples when a species functions only in a narrow temperature range or maintains its body temperature in an even more precise manner. Do those species lack the apparently numerous compensatory proteins or other molecules that the thermally adaptable species employ?
And how does this thermostabilisation mechanism save against monomolecular transformations, such as temperature-sensitive folding and misfolding of proteins? Nevertheless, even without definite answers to those questions, the manuscript presents an interesting proof of principle study and could be an important contribution to the progressively more diverse topic of protein networks. So I would like to support publishing it in Nature Communications if the Authors address the following issues.

1. What I suggested above should in some way be reflected in the text as some kind of an honest disclaimer. Ideally, several examples of temperature-stabilized protein-protein or RNA-RNA interactions could be given. The specificity and limitations of the oligonucleotides dimerisation network should be discussed and compared to the protein-protein LMA networks. For example, similarly to the protein binding networks, the possibility of formation of complexes consisting of more than 2 strands need to be addressed. I guess this could be a real issue in the models presented in Figs 5 and 6.

2. The software that predicts dissociation constants, NUPACK, is used merely as a black box. It would be helpful to comment on how it works and why it is necessary to deconstruct the temperature-dependent free energy into the enthalpic and entropic parts while these parts are apparently computed separately inside NUPACK. Why the enthalpy and entropy for binding between entire strands can be considered temperature-independent, or is the separation into H and S just a linear regression of the temperature dependence of the free energy. Given the pure statistical nature of several models analyzed, would a random distribution of dissociation constants (or free energies) of a particular type and temperature correlation (such as shown in Fig 1 A and B) serve as an equally convincing example?

3. The term "computation" has become way too overloaded and is often applied to any system that has identifiable input and output and exhibits some kind of non-linear response between them. In my opinion, in the text, the term "computation" is misleading and even sounds like a false promise. While various non-linear and combinatorial functions can be implemented using a mass-action networks, those networks are intrinsically limited by the spatial decay of the signal, generally exponential, and the associated with it reduction in the range of binary or continuous signals.
So unless the Authors prove the opposite, I cannot see how a computer can be built using such a network.
I strongly suggest to tone it down and replace "computation" and adjectives that are derived from it by a less flashy but more realistic terms like "signal transformation" or "signal processing".

4. And several minor issues

The phrase in lines 71-74 is hard to parse. It's also not clear what the authors mean under the term "integrator".

Line 102, should it be an em (emphasis) dash instead of a hyphen?

Line 141 "that" -> "this"?

Fig. 1E, are homodimeric dissociation constants chosen randomly to be large and thus irrelevant?

Line 253, 100 nM vs. line 267, 1 μM.

"Output concentration" in the vertical axis of Fig. 2 vs. "Free S concentration" in Fig. 3

Line 572 "describing the fundamental algorithms of the service"?

Fig. 3 and the related discussion: One can argue that the non-compensated YES gate (as well as modules considered in subsequent examples) performs even better than the "temperature-independent" gate: While the difference between the signal and no-signal levels of S is identical at 50 degrees, this difference is more pronounced at 25 degrees for the gate with no C.

(Remarks on code availability)
Since the modeling involves several pre-packaged codes, it's hard to assess how the results are affected by the programmes written by the authors.

Reviewer #2

(Remarks to the Author)
I greatly appreciate the overarching goal of this work. The manuscript introduces a compelling and broadly useful design principle (which the authors call topological temperature/ionic-strength compensation) that could expand the toolkit for robust molecular circuit design and, more speculatively, suggest organizing principles for natural regulatory networks. While it is well known that nucleic-acid hybridization (eg the nearest-neighbor model) involves substantial enthalpic and entropic contributions, I am not aware of prior work using the discrepancy between these contributions as a design lever. The central idea, that of achieving temperature- and ionic-strength–insensitive behavior, is highly impactful both for molecular engineering and for thinking about principles that could operate in biological regulatory networks. If generalizable beyond DNA, this approach could enable more reliable molecular computation across fluctuating environmental conditions and provide a concrete framework for reasoning about compensation mechanisms in biochemical networks.

That said, the presentation would benefit substantially from making the thermodynamic intuition explicit much earlier. The manuscript relies on the different temperature dependence of entropic versus enthalpic contributions (i.e., that the same ΔG at one temperature can arise from different $\Delta H/\Delta S$ combinations, leading to different behavior across temperatures), but this framing is not clearly emphasized until late in the Discussion. I recommend stating this up front in the Introduction and including the basic relationship $\Delta G(T) = \Delta H - T\Delta S$ early on. I would also encourage the authors to cite prior DNA molecular programming work that leverages entropy/enthalpy tradeoffs, even if those examples involve different notions of entropy (e.g., numbers of complexes) than the nearest-neighbor hybridization contributions emphasized here (eg, refs. [a,b] below).

A related point concerns the claimed generality of the results. The authors argue (reasonably, in my view) that DNA serves mainly as a convenient test case for a broader compensation principle. However, the manuscript would be strengthened by citing representative literature directly showing that protein–protein interactions can exhibit similarly variable enthalpic and entropic terms, making analogous compensation plausible in protein-based networks.

One presentation issue may greatly confuse readers: the manuscript frames the initial demonstrations as building a “YES,” “NOT,” and “AND” gate, which suggests that the compensated designs should perform as good logic gates across temperature. In the current presentation, the uncompensated gates tend to separate logic states better at 25 °C than at 50 °C, whereas compensation largely makes the 25 °C behavior resemble the poorer separation seen at 50 °C (i.e., the low-temperature performance is “degraded” to match the high-temperature behavior). I understand that the primary goal is to illustrate temperature dependence and compensation rather than to optimize digital logic robustness as such, but as written, the fact that “temperature compensation” can make the system behave less like a crisp logic gate at low temperature may put off some readers. At minimum, it would help to acknowledge this tradeoff explicitly and clarify that invariance is achieved relative to a chosen operating point, not necessarily maximal gate fidelity across conditions.

The authors might also note that an alternative route to temperature robustness in these specific gate motifs would be simply to strengthen binding so the relevant complexes are nearly fully formed at both 25 °C and 50 °C. Of course, this would sacrifice the benefits of weakly complementary designs (and likely would not extend cleanly to the richer "algebraic" systems), but stating this explicitly would help readers understand what is special about the presented approach.

An important issue is about the use of NUPACK. Throughout the paper NUPACK was used with maximum complex size parameter set to 2. However, in typical systems especially at lower temperatures there can be substantial trimolecular and higher-order binding. I tried simulating the system in Figure 4B with max_complex_size = 3 and obtained substantially different results showing much less compensation at lower temperatures (I am attaching the comparison plots to this review). I think this might also be a reason that NUPACK predictions were not matching experimental data. Although the expected binding is dimerization, the authors need to be explicitly aware and acknowledge unexpected/undesired higher order binding. Ideally, the systems are reworked to make them robust to a higher max_complex_size parameter.

I appreciated that the full source code is provided (including a general README), which was very helpful. However, for the optimization problem (Bayesian adaptive search), the problem setup should be stated more clearly in the manuscript without requiring a code dive. In particular, it was not fully clear what constraints and criteria (e.g., maximum/minimum allowed concentrations, excluded interaction thresholds, etc.) are enforced. It would help to explicitly state the loss function and summarize the key constraints in the Methods or Supplementary Information.

Other more minor issues:

- Line 25: Typo: "up to" => "from" or some other wording
- Figure 1: The count of S's should be the same in both "no input" and "with input" cases
- Line 113: Incomplete sentence or awkward grammar of sentence starting with "If we consider duplexes that exhibit..."
- Line 158: Clarify which four dissociation constants. What about off-target/undesired dissociation constants?
- Line 192: it should probably be "M binds to I, reducing M's availability to bind Q."
- Figure 2 and other figures: The figures and/or figure captions should mention concentrations of inputs (what does 1 input mean), as well as concentrations of other components.
- Line 221: Typo: "Importantly, if instead of DNA they networks..."
- Figure 3A: In all the other cases, Q:I is stronger than Q:S, but in set 3, the Kd's are the same. Is this a typo?
- Figure 3C: More details of experimental conditions are needed, specifically how long was the incubation time. Otherwise, it's not clear what the connection is to Figure 3A.
- Figure 4: Please double-check all the sequences shown in the figures. The supplementary information has different sequences than shown in 4B, for example (SI, page 11, top). Also in 4B, the "M" strands should be "C". I believe the sequences in the SI are the correct ones. (I didn't check the consistency of other sequences.)
- Figure 5A: Because the top line has three things in order (A, R, Activated receptor), it is easy to visually misinterpret the second line (C1---A1---R), which also has three things, to refer to the molecules shown in the first line in order.
- Line 434: Since the equation on the previous line ends with a comma, this line should not begin with a capital "W".
- Figure 6: While the text is clear that X and Y are on vastly different scales (5 uM vs 20 nM), I think the figure should make this more clear in the figure itself or in the caption at least. Otherwise, the units are too abstract.
- Line 479: Unclear what "contemporary regulatory systems" means.
- Line 480: It would help to tighten this paragraph to a few sentences.
- Line 549: Should this refer to Figure S1 rather than S3?
- Line 652: Missing year in ref 18

References:

[a] Jaeseung Hahn, William M Shih, Thermal cycling of DNA devices via associative strand displacement, Nucleic Acids Research, Volume 47, Issue 20, 18 November 2019, Pages 10968–10975

[b] Song, T., Qian, L. Heat-rechargeable computation in DNA logic circuits and neural networks. Nature 646, 315–322 (2025)

(Remarks on code availability)

Reviewer #3

(Remarks to the Author)
The work by Korenkov and Nikitin investigates how equilibrium responses of molecular networks can be made insensitive to temperature and salt concentration by adding additional interactions whose interactions contribute at low temperature but not at high temperature, so that changes in binding strengths cancel out at the network level. This compensation mechanism is first discussed theoretically for DNA-oligos and then illustrated experimentally by implementing a temperature compensated DNA-based YES-gate. The proposed salt-compensation is supported by numerical modeling but is not experimentally validated. The remainder of the manuscript is also theoretical, addressing large networks with ligand-receptor binding as well as networks that can perform algebraic calculations. In all cases, Bayesian optimization methods are used to design the networks.

Major comments

This work builds on an earlier publication by Nikitin (Nature Chemistry 2022, introducing molecular commutation) and extends it into the temperature domain. The phenomenon of temperature compensation has been studied quite extensively in the context of circadian clocks, albeit for oscillating behaviors instead of equilibrium responses. Such responses have been approached in enzymatic networks (e.g., Ruoff, Zakhartsev and Westerhoff, 2006) or more specifically in the chemotactic reaction network of E. coli (Oleksiuk, 2011). While the framework of molecular commutation adds a different angle to it, it does therefore not constitute a major novelty by itself. Compensation with regard to ionic strength seems less systematically explored and is indeed another interesting aspect to robustness of molecular computation. But as the authors write themselves, its effect is similar to temperature, because both weakens interactions.

Another aspect of this work is that the construction principle described by the authors is an example of "fine-tuning", while it is generally accepted that biological systems evolve robust mechanism that would also work under perturbations. The fact that dissociation constants can be found that compensate for temperature effects is interesting, but not particularly surprising. The authors' approach to fine-tune the system is reminiscent of early circadian clock models going back to Leloup & Goldbeter, 1997 and Hong & Tyson, 1997. However, the field has moved to more robust temperature compensatory designs deeming a fine-tuned temperature compensation as too unrealistic for biological systems (compare, e.g., Kidd, Young & Siggia, 2015 among others). The temperature compensation mechanism proposed by the authors has the same explanatory difficulties – if not worse, as it would require precise compensatory reactions across a multifunctional network as opposed to a time-keeping motif. Therefore, it seems questionable if the proposed mechanism is plausible in vivo. Many of the explanations in this regard ("The phenomenon of topological compensation described above allows a reconsideration of both key evolutionary mechanisms and contemporary regulatory systems") are exaggerations and there is no proof that the described mechanisms have been evolved by cells.

Title and abstract do not convey that this work is mainly theoretical in nature and focused mainly on DNA-oligos. Interestingly, the experiments show that the NUPACK-predictions are not very accurate, while the main part of this work is based on exactly this software, but this contradiction is not discussed. Further it is unclear if optimization at two temperatures (25 and 50 degrees Celsius) is sufficient to get a flat response in all cases; it could well be that in some cases, the optimization procedure, because it is fine-tuned, would miss a complete compensation. For the large systems, the result looks over-optimized, because the response is unrealistically flat. Biological systems have to be robust in many ways and are never optimized so strongly only in one regard as done here.

In summary, the theoretical treatment on its own is neither methodologically nor conceptually a major advance and their framing as 'fundamentally novel property' (line 81) is not fully justified in light of, for example, the significant body of literature on temperature compensation in circadian clocks. The experimental implementation of a single temperature compensated YES-gate with statistics based on n=2 replicates and no experimental verification of the salt-compensation does not constitute a thorough experimental study of the many theory claims made in this work. In this regard, their "arbitrarily high accuracy" (line 84) to topological compensation claim feels stronger than what is shown – especially, when keeping in mind that this is much more a result of topology and careful parameter tuning than of topology alone. In addition to that, placing this in a biological setting as done by the authors needs more care, not just because of the fine-tuning aspect, but also given that their treatment is restricted to equilibrium systems, while biological signaling usually works out-of-equilibrium.

Minor comments

Figure 1: "olgios" is a typo

Figure 3: "bioimaging" is not appropriate

Line 144: Missing the: "...the system behaves like a Boolean function YES: the logic output of this system is always the same as the input"

It is great that the code is made available; a code availability statement is however missing.

(Remarks on code availability)
The code comes in the form of Jupyter notebooks and seems reasonable. It is great that the authors also supply the data and figures with it.

Reviewer #4

(Remarks to the Author)
I co-reviewed this manuscript with one of the reviewers who provided the listed reports. This is part of the Nature Communications initiative to facilitate training in peer review and to provide appropriate recognition for Early Career Researchers who co-review manuscripts.

(Remarks on code availability)

Version 1:

Reviewer comments:

Reviewer #1

(Remarks to the Author)
The Authors have addressed most of my and other reviewers' comments and, based on the content of the manuscsipt, I would recommend publishing it in Nature Communications.

However, a major remaining issue is the style of presentation.
I guess I would overstep my authority as a reviewer if start listing what I consider flaws in grammar and punctuation. I do see many of those and would strongly recommend an extensive technical editing before publishing this piece. Perhaps, it is a result of using an AI assistant acknowledged by the Authors. Even the new edition of the title is hard to parse.

"In the extreme case of long, fully complementary strands, the
interaction becomes effectively irreversible, and complexes remain stable almost indefinitely at room temperature conditions37, which in practice corresponds to an effectively infinite operating time for the system." Operating time is a wrong term in this context: it usually means how long smth works before a failure.

Another suggestion is to tone down (or even better, remove) the discussion about the possibility to use elevated body temperature as signalling. Physically, that would be very inefficient due to the high heat capacity of body fluids (essentially water) and slow heat conduction. As far as I know, the mechanism of resetting the body thermostat is fairly well understood and is attributed to chemical signalling by a fever-inducing substance (pyrogen).

Same suggestion applies to discussing the "evolutionary context". A necessary prerequisite for such a discussion is a well-established presense of those thermostabilizing mechanisms in the present life forms.

Perhaps a minor point that I missed preparing my previous review: can the authors comment on the actual rates of equilibration in their chemically-implemented "YES" gate? Such a long process seems virtually useless to buffer any important signalling pathway from, say, daily temperature variations.

(Remarks on code availability)

Reviewer #2

(Remarks to the Author)
The authors have addressed all the issues I raised in my original review. I believe the paper is ready for publication.

(Remarks on code availability)

Reviewer #3

(Remarks to the Author)
The authors have performed a very impressive revision, including many more theoretical and experimental results and responding in much detail to all comments by the three reviewers. We think that the question of novelty is very important, but now settled, because from the many results now contained in the revised manuscripts, some are certainly novel and it is a matter of context and community which exactly one wants to name here. It would not be entirely correct to say that earlier work on temperature compensation only considered enzymatic networks, or that the authors here consider affinity networks for the first time, or that biology has evolved exactly the same mechanisms as described here, but the new text is sufficiently balanced now that the readers see the complexity of and earlier answer to these questions. In general, the authors have done a very good job in explaining better what exactly has been achieved, including the rewritten title and abstract. This work will certainly appeal to many different communities and thus will find a broad readership, making it very appropriate for Nat Comms.

The authors have also done a good job in integrating new references. We in particular like the comments on temperature as a universal signal during fever. As a last comment, we note that the manuscript is relatively short on reviews. If more changes should be made (for example, the use of Kd/K_d could be homogenized in text and figures, and there are a few missing periods), the following reviews on temperature and fever could be included, it the authors find this appropriate:

Knapp, Benjamin D., and Kerwyn Casey Huang. "The effects of temperature on cellular physiology." Annual Review of Biophysics 51.1 (2022): 499-526.

Jacobs, Simen, et al. "Understanding the temperature response of biological systems: Part I-Phenomenological descriptions and microscopic models." Current Opinion in Systems Biology(2026): 100577.

Jacobs, Simen, et al. "Understanding the temperature response of biological systems: Part II-Network-level mechanisms and emergent dynamics." Current Opinion in Systems Biology(2026): 100578.

Bourn, Jess J., and Michael W. Dorrity. "Degrees of freedom: temperature's influence on developmental rate." Current Opinion in Genetics & Development 85 (2024): 102155.

Evans, Sharon S., Elizabeth A. Repasky, and Daniel T. Fisher. "Fever and the thermal regulation of immunity: the immune system feels the heat." Nature Reviews Immunology 15.6 (2015): 335-349.

(Remarks on code availability)
Code availability has now been clarified. Github would still be good, but supplemental material is also fine.

Reviewer #4

(Remarks to the Author)
I co-reviewed this manuscript with one of the reviewers who provided the listed reports. This is part of the Nature Communications initiative to facilitate training in peer review and to provide appropriate recognition for Early Career Researchers who co-review manuscripts.

(Remarks on code availability)

Version 2:

Reviewer comments:

Reviewer #1

(Remarks to the Author)
The Authors have addressed all my critique and suggestions and I think the manuscript can be accepted for publication in Nature Communications in its present form. As for the title, I personally prefer the new, shorter version, but it's naturally up to the Authors to decide which version better reflects the content.

(Remarks on code availability)

Reviewer #3

(Remarks to the Author)
The authors have responded very well to our comments and followed all our suggestions. In particular, they have improved clarity and language, included several relevant reviews, and will make their code publically available on GitHub. They also reacted well to the conflicting advise from another reviewer and ourselves on how much to discuss the issue of temperature as a universal signal, esp. in the context of fever. This subject and similar ones are now discussed in a balanced way in the text, so that the readers can form their own opinions and follow the references. Overall the text and the figures are easy to follow now. The title stays a bit bulky and hard to digest, because the authors want to include all aspects of their work, but I think that it is really up to them how to title their work and I do not have any objections here. In summary, this work is excellent and I recommend acceptance as is.

(Remarks on code availability)
I have looked at the code in a previous version and it looked good to me. It is great that it will be published on GitHub.

Reviewer #4

(Remarks to the Author)
I co-reviewed this manuscript with one of the reviewers who provided the listed reports. This is part of the Nature Communications initiative to facilitate training in peer review and to provide appropriate recognition for Early Career Researchers who co-review manuscripts.

(Remarks on code availability)

Open Access This Peer Review File is licensed under a Creative Commons Attribution 4.0 International License, which permits use, sharing, adaptation, distribution and reproduction in any medium or format, as long as you give appropriate credit to the original author(s) and the source, provide a link to the Creative Commons license, and indicate if changes were made.
In cases where reviewers are anonymous, credit should be given to 'Anonymous Referee' and the source.
The images or other third party material in this Peer Review File are included in the article's Creative Commons license, unless indicated otherwise in a credit line to the material. If material is not included in the article's Creative Commons license and your intended use is not permitted by statutory regulation or exceeds the permitted use, you will need to obtain permission directly from the copyright holder.
To view a copy of this license, visit https://creativecommons.org/licenses/by/4.0/

Dear Reviewers,

First of all, we would like to express our deep gratitude for the careful evaluation of our manuscript and insightful comments.

To thoroughly address all the Reviewers' remarks, we have performed additional experiments and simulations and have carefully revised the manuscript to improve its structure and language. Please find below the point-by-point description of the revisions according to your comments (Reviewer's questions given in **bold**, answers - in plain text, quotations from the manuscript – in *italic*).

To the Reviewer 1

The manuscript "Topology of molecular networks offers signaling insusceptibility to temperature and ionic strength changes" explores a concept of temperature stabilisation of signal processing biochemical modules using buffering substances with temperature-calibrated affinities. It describes a diverse set of in-silico examples and an experimental verification of the simplest module, the repeater, or "yes" gate. The modules consist of several substances (oligonucleotides) that reversibly dimerise according to the law of mass action.

The conceptual design is similar to commonly used chemically-buffered modules, where a weakly bound substance is released or sequestered when the external conditions change, making the module itself less susceptible to those changes. Here the external conditions are defined as the temperature and ion concentrations, so the binding affinities of corresponding compensating substances have to be more sensitive to those changes than those of the principal components of the modules. To the best of my knowledge, this is new. The manuscript clearly and convincingly demonstrates that such a principle works and it is indeed possible to design modules that maintain their performance in the range of temperatures where the dissociation constants change by orders of magnitude. And the experimental verification complements the modeling and confirms the main conclusions.

We sincerely appreciate the reviewer's positive and thoughtful evaluation of our work and are especially grateful for the constructive suggestions that helped improve the manuscript. Please find our responses below.

1.1 However, the text begs a question of whether anything like this happens in any naturally-occurring biochemical system

We have added to the Introduction a description of the biochemical systems, which were demonstrated to be temperature independent:

"Interestingly, enzymatic networks may be involved in temperature independence in naturally occurring systems under certain conditions[15]*, namely when the weighted sum of the activation energies of specific reactions in the network equals zero. This mechanism is considered to contribute to the thermal stability of particular biochemical cascades, such as circadian rhythms*[16–18] *or bacterial chemotaxis*[19]*. However, evolution-driven or artificial design of the enzymatic networks that adhere to such a narrow constraint seems to be highly complex as these systems must additionally develop and retain other critical parameters for each molecule of the network, including substrate affinity, substrate selectivity, catalytic function, reaction rates etc.*

…

15. Ruoff, P., Zakhartsev, M. & Westerhoff, H. V. Temperature compensation through systems biology. FEBS J. ***274****, 940–950 (2007).*

16. Leloup, J.-C. & Goldbeter, A. Temperature Compensation of Circadian Rhythms: Control of the Period in a Model for Circadian Oscillations of the Per Protein in Drosophila. Chronobiol. Int. ***14****, 511–520 (1997).*

17. Hong, C. I. & Tyson, J. J. A Proposal for Temperature Compensation of the Orcadian Rhythm in Drosophila Based on Dimerization of the Per Protein. Chronobiol. Int. ***14****, 521–529 (1997).*

18. Kidd, P. B., Young, M. W. & Siggia, E. D. Temperature compensation and temperature sensation in the circadian clock. Proc. Natl. Acad. Sci. ***112****, (2015).*

19. Oleksiuk, O. et al. Thermal Robustness of Signaling in Bacterial Chemotaxis. Cell ***145****, 312–321 (2011).”*

At the same time, the underlying mechanism responsible for such stabilization of higher-level systems remains to be elucidated. The compensation mechanism we propose for affinity-based networks substantially surpasses the demonstrated compensatory capabilities of enzymatic systems due to the much less stringent thermodynamic requirements imposed on the network components. In our case the molecules are required to optimize only a single property: their affinity toward interaction partners. In contrast, compensation in enzymatic networks requires precise matching of activation energies, while retaining other parameters, like substrate affinity, substrate selectivity, catalytic function, reaction rates, etc.

Given that temperature independence is indeed observed in nature, and that our proposed compensation mechanism is both readily realizable and exhibits superior compensatory capabilities, we believe that future studies may discover that it is precisely this mechanism that is responsible for temperature independence in real biological systems, for example, in enzyme-based networks through control of the temperature dependence of enzyme expression levels.

To demonstrate the latter statement and taking into account the comments of all Reviewers, we have performed an additional study that generated a very interesting result: affinity-based systems can be designed to exhibit virtually any predefined temperature or ionic strength dependence (not just be independent from them as we showed in the initial version of the manuscript):

*“**Programmable temperature/ionic strength dependence***

After thoroughly discussing temperature independence (i.e., a dependence reduced to a constant), we proceed to the more general task of designing systems with arbitrary dependence on temperature or ionic strength. Such behavior could be beneficial, for example, for inducing signals of arbitrary forms in different cell types as body temperature rises during disease.

Another illustrative example of where such systems may be important is in ensuring temperature independence of a higher-level molecular system with its own complex temperature dependence. For example, consider temperature-independent bacterial chemotaxis. In this well-studied system[19] the key determinants of this independence include (1) the enzymatic network and (2) the mRNAs encoding these enzymes together with the translation-related machinery. The latter ensures a selective and tightly controlled increase in the expression of specific components of the enzymatic network as temperature rises.

In turn, as demonstrated in our prior work[21], there exist mechanisms by which molecular commutation can significantly influence protein translation levels. Specifically, binding of DNA

*oligonucleotides to a specific site on an mRNA molecule may lead to its cleavage mediated by RNAse H, thereby reducing the expression of the corresponding protein (**Fig. 7A**). Consequently, free mRNA levels (i.e., mRNA not bound to oligonucleotides), representing the output of the nucleic acid affinity-based network, may directly determine protein concentrations.*

*Thus, suppose that a certain complex biochemical system requires, for its temperature independence or other higher-level signaling, a specific temperature-dependent profile of the level of free mRNA (**Fig. 7B**). In this section we show that it is possible to identify sets of DNA oligonucleotides that realize predefined complex, non-monotonic dependencies on temperature or ionic strength.*

The formal statement of the problem is analogous to that of the previous section: the variable Y (the level of free mRNA) is defined in exactly the same way, whereas the variable X is defined as the normalized and centered temperature:

$$X = \frac{T - T_{min}}{T_{max} - T_{min}}.$$

The identification of DNA sets having a predefined temperature dependence Y(X) was carried out in a manner similar to the design of logic gates. We first fixed the sequence of the "output oligonucleotide" as the AUG-containing region of the firefly luciferase mRNA that was used in our prior study[21] for the experimental demonstration molecular commutation effect on protein expression.

For each specified function Y(X), the required dissociation constants, "easy-to-melt" levels, and concentrations of the components were first determined using Bayesian optimization. After that, DNA sequences realizing these parameters were identified using an evolutionary algorithm. Finally, an evolutionary fine-tuning of both sequences and concentrations was performed to ensure that the system exhibited the desired behavior while accounting for nonzero concentrations of triple complexes (NUPACK max_compl_size = 3).

*This procedure was carried out for four sample dependencies Y(X): linear, quadratic, cubic, and sinusoidal. The resulting DNA sets, their interaction graphs, and their temperature dependencies are presented in **Fig. 7C–F**.*

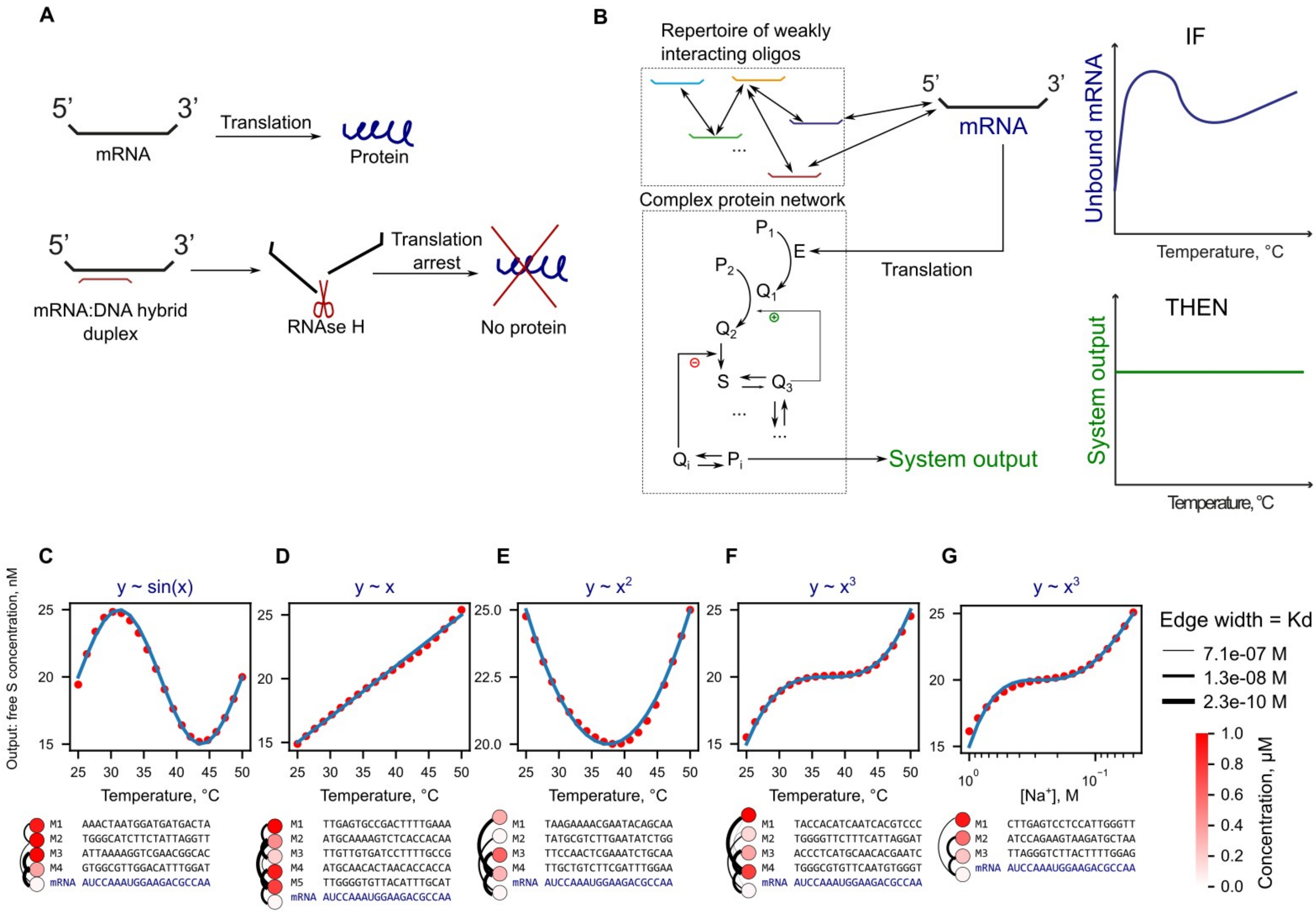

***Fig.** 7. Molecular commutation systems with programmable temperature/ionic strength dependence. (A) Schematic of translation control via an RNAse H-mediated mechanism. (B) Scheme of a nucleic acid molecular commutation system that compensates for the complex behavior of a protein network. Temperature compensation of the protein network requires a predefined level of protein expression, which is regulated by mRNA levels. (C–F) Performance and interaction graphs of molecular commutation systems exhibiting linear, quadratic, cubic, and sinusoidal temperature dependence. (G) Performance and interaction graph of molecular commutation system exhibiting cubic temperature dependence on ionic strength. X axis direction is reversed in (G). Solid lines represent the objective profile, and dots represent the simulated system performance. Node colors represent oligonucleotide concentrations, and edge widths represent the corresponding dissociation constants. DNA-RNA interaction was simulated according to DNA-DNA NUPACK model. The simulation was performed taking into account complexes consisting of up to three strands (NUPACK max_compl_size = 3).*

*In addition, **Fig. 7G** shows that nucleic acid networks can exhibit similarly complex, predefined dependencies on ionic strength. Since the free energy of hybridization depends logarithmically[40] on sodium concentration (in contrast to its linear dependence on temperature), for consistency, we chose the logarithm of the normalized sodium concentration as the independent variable (X):*

$$X_{ionic} = \log_{10}\left(a\left[Na^{+}\right]+b\right),$$

the constants a and b were chosen such that the sodium concentration range from 50 mM to 1 M is mapped onto the interval [0, 1] for X. Using a DNA set previously optimized to produce a

cubic dependence on temperature (see Materials and Methods for details), we applied an evolutionary algorithm to identify a new set that realizes an analogous dependence on ionic strength.

To summarize, this remarkable feature of the molecular commutation networks allows them to receive a single cue of the environment (e.g., temperature or ionic strength) and react in many different ways. This phenomenon seems to be a perfect match for a hypothesis that the elevated body temperature during disease can act as a universal information carrier to all cells of the organism, potentially capable of triggering, in each cell type, its own multitude of independent signaling profiles. Initiating these programmable responses via temperature may be highly valuable since it does not dependent on mass transport of molecular messengers throughout the body.»

In our view, such programmability of temperature dependence in molecular networks has potential relevance across an even broader range of systems, either as a compensatory mechanism or as a standalone signaling tool.

Accordingly, we have changed the title and the abstract to reflect these new results. The new title is "*Temperature- and ionic strength-independent or programmably dependent molecular signaling enabled by affinity-based molecular commutation networks*", and the new "Here we show" statement of the abstract is "*Here, we show that molecular commutation enables complex signaling systems that are independent of temperature and ionic strength and, even more generally, programmably dependent on these parameters.*"

1.2 Several examples of organisms (and organs) that function in a range of temperatures are referred to in the Introduction. Yet at the same time there are lots of examples when a species functions only in a narrow temperature range or maintains its body temperature in an even more precise manner. Do those species lack the apparently numerous compensatory proteins or other molecules that the thermally adaptable species employ?

This is indeed an interesting topic for discussion; therefore, we have added the following paragraph into the Discussion:

"An interesting question is that, if early living systems hypothetically achieved temperature independence via this mechanism, why do many existing organisms (perhaps their evolutionary descendants) nonetheless exhibit significant sensitivity to changing temperature? One possible explanation is the emergence of dedicated homeostatic mechanisms, which may have relieved molecular networks from the requirement for self-compensation leading to gradual repurposing of compensatory participants. As highlighted in Fig.7, more complex organisms may have adapted this temperature-response mechanism as a highly effective means of systemic signaling coupled to changes in body temperature (e.g., during disease). This may have enabled distinct cell types to independently evolve specialized responses (such as modulating metabolism or triggering particular signaling cascades) while contributing to a coordinated organism-level outcome. It is possible, that the universality and flexibility of such signaling could confer significant evolutionary advantages over mechanisms relying solely on chemical signaling via mass transport."

1.3 And how does this thermostabilisation mechanism save against monomolecular transformations, such as temperature-sensitive folding and misfolding of proteins? Nevertheless, even without definite answers to those questions, the manuscript presents an interesting proof of principle study and could be an important contribution to the progressively more diverse topic of protein networks. So I would like to support publishing it in Nature Communications if the Authors address the following issues.

Indeed, in the case of proteins, the aforementioned monomolecular reactions may contribute significantly to the overall temperature dependence. However, we hypothesize that as long as such changes remain within a reasonable range (i.e., for example, not all proteins in the system denature into an inactive state), they can be readily compensated by the same mechanism. To demonstrate this, we performed an additional simulation of large-scale receptor–activator networks in which the activators undergo reversible temperature-dependent misfolding (Supplementary Fig.15).

The following paragraph has been added to the Results section, "Receptor activation in complex networks":

"So far, we have shown compensation in systems in which only dimerization or higher-order processes were present. However, in real molecular systems, temperature dependence may also arise from monomolecular processes, such as protein misfolding. To demonstrate the performance of topological compensation in this case, we introduce a reversible temperature-dependent misfolding process (leading to transition of participants into fully inactive state) into similar large receptor-activator networks. Such reactions lead to, in addition to the previously considered dimerization temperature dependence, a temperature dependent reduction in the effective total concentrations. ***Supplementary Note 4*** *and* ***Supplementary Fig.15*** *show that such systems can also be efficiently stabilized against temperature changes."*

"Supplementary Note 4. Temperature compensation in receptor-activator networks with monomolecular temperature-dependent processes

Consider a receptor–activator system analogous to that shown in Fig.5D. In addition, let us introduce into this system a process of reversible misfolding.

$$A \leftrightarrows A_m$$

with equilibrium constant

$$q_i = \frac{m_i^{eq}}{c_i^{eq}}$$

Here c_i^{eq} *and* m_i^{eq} *denote the equilibrium concentrations of the native and misfolded forms of the i-th molecule, respectively. Let* K_{ij} *denote the affinity constants for dimerization,* c_i^{total} *the total concentrations, and* c_{ij}^{eq} *the equilibrium concentrations of the dimers. The i-th mass-balance equation can then be written as:*

$$c_i^{total} = c_i^{eq} + m_i^{eq} + \sum_{i \neq j} c_{ij}^{eq}$$

Substituting the definitions of the equilibrium constants:

$$c_i^{total} = c_i^{eq} + q_i c_i^{eq} + \sum_{i \neq j} K_{ij} c_i^{eq} c_j^{eq}$$

Solving for c_i^{eq}*:*

$$c_i^{eq} = \frac{c_i^{total}}{1 + q_i + \sum_{i \neq j} K_{ij} c_j^{eq}}$$

Thus, we obtain a fixed-point map that differs from that in the work of van Dorp[4] by the presence of q_i *in the denominator and can be reduced to a canonical form by rescaling the concentrations*

and the dimerization constants by a factor of $1+q_i$. *Accordingly, the equilibrium concentrations are determined in the same manner as for all other systems considered in the paper, except for this modification.*

To specify particular misfolding constants, we proceed as follows. For each molecule in the system, we define the fraction f_i *of the misfolded form at 25 and 50 °C, which is straightforwardly related to* q_i*:*

$$q_i = \frac{f_i}{1 - f_i}$$

If needed, the values of the constants at intermediate temperatures are calculated using van 't Hoff interpolation. For definiteness, we assume $f_i^{25^\circ C} = 0.001$, *i.e., almost all molecules are in the native state at 25 °C, and vary only* $f_i^{50^\circ C}$. *The network parameters are specified in the same way as for the network in Fig.5D, except that each activator is assigned a random value of* $f_i^{50^\circ C}$ *in the range from 0.2 to 0.9. These values remain fixed during the optimization process.*

Supplementary Fig.15 *compares the performance of the optimized and control systems: the optimized system shows nearly identical activation at both temperatures, whereas the control system exhibits significant discrepancies."*

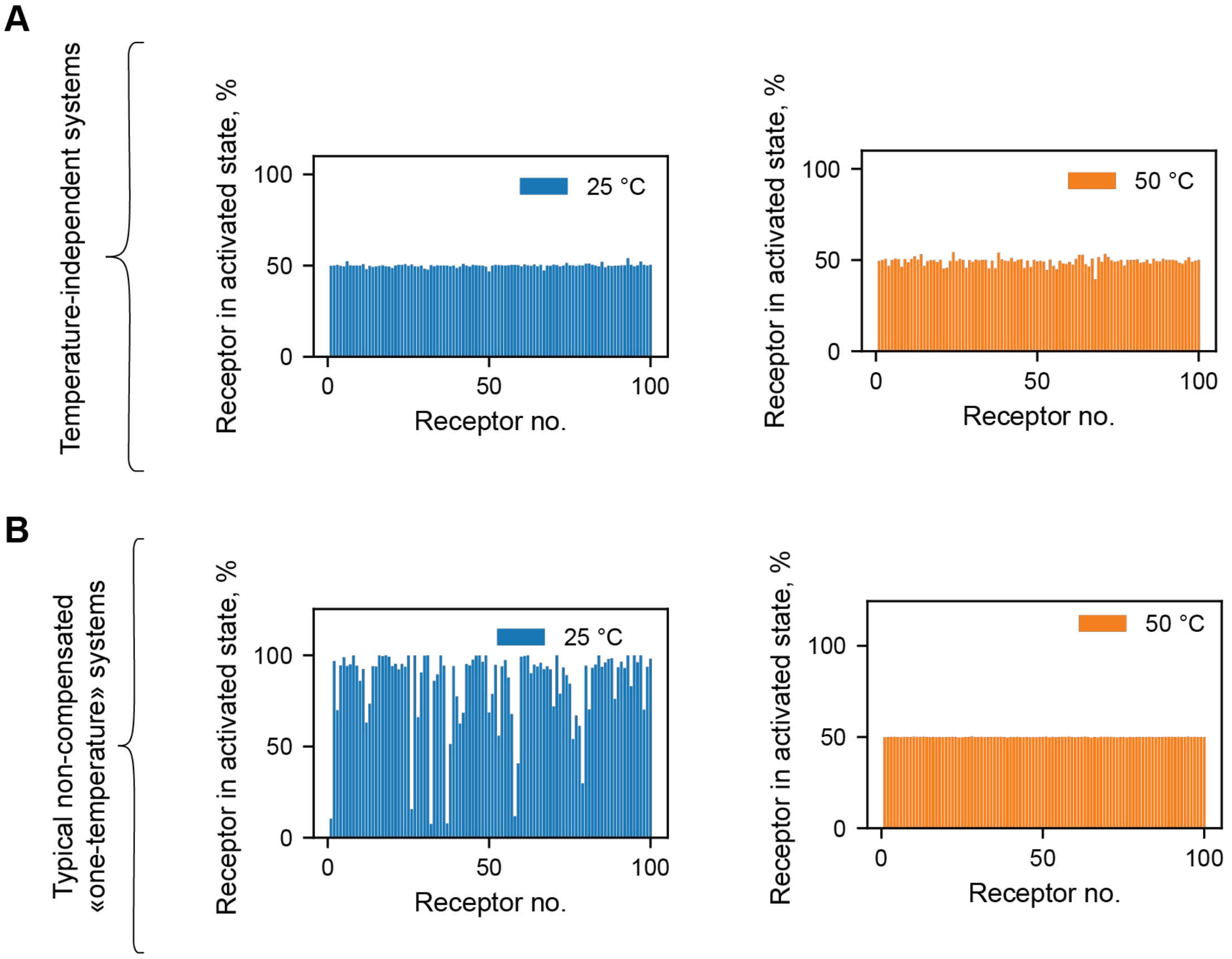

Supplementary Fig.15*. Performance of random receptor-activator network with activator misfolding (A) Temperature-independent system (B) Typical system without compensation, optimized separately to have predefined activation levels at 50 °C only. A detailed description of the system is provided in Supplementary Note 4."*

1.4 What I suggested above should in some way be reflected in the text as some kind of an honest disclaimer

We hope that we have discussed these truly interesting proposed topics in sufficient depth in the answers above, and we would be happy to address any follow-up comments.

1.5 Ideally, several examples of temperature-stabilized protein-protein or RNA-RNA interactions could be given.

Thank you very much for the question. Indeed, explicitly stating that the compensation principle may also apply to other types of molecules could strengthen the manuscript. To demonstrate that RNA–RNA interactions are also suitable for a temperature-compensated system, we redesigned the NOT gate from Fig.2B using RNA sequences.

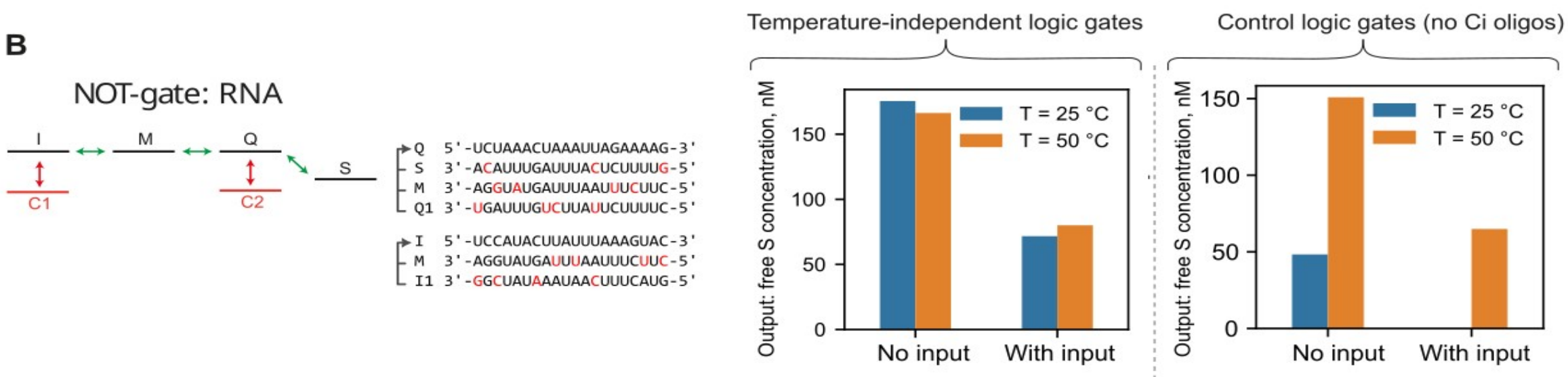

"Fig.2. Design and predicted performance of temperature-independent logic gates. (A) DNA-based YES gate. (B) RNA-based NOT gate..."

To demonstrate that topological compensation is feasible in the case of protein–protein interactions, we have added analysis of existing databases (Supplementary Fig.1 and Supplementary Note 1) and have shown that protein interactions likewise exhibit the characteristics required for such compensation.

The following excerpts have been added to Results sections:

"The different behavior of Kd at two temperatures arises from the relative independence of enthalpy and entropy …

*Importantly, this property is not unique to nucleic acids, and can also be observed, for example, in protein interactions (**Supplementary Note 1**)….*

Below, we show that this variety of "easy-to-melt" and "hard-to-melt" pairs can be exploited to render a wide range of affinity-based signaling systems temperature-independent."

"Supplementary Note 1. Analysis of the feasibility of temperature compensation in the context of protein–protein interactions.

As previously noted, the existence of temperature compensation in networks of interacting oligonucleotides is enabled by the existence of "hard-to-melt" and "easy-to-melt" pairs. Importantly, in this context, nucleic acids serve merely as a convenient model system that allows straightforward evaluation of their thermodynamic properties. More generally, any biomolecules that exhibit distinct temperature-dependent behaviors are likewise capable of demonstrating analogous compensatory effects.

To demonstrate this, we consider the case of protein–protein interactions. For the analysis, the Stites (1997) dataset[1] *was selected. Because, for such interactions, the enthalpy typically exhibits a strong temperature dependence*[1] *(i.e., the change in heat capacity ΔC_p is significantly different from zero), only data for which ΔC_p was directly measured were included in the analysis. Otherwise, the use of enthalpy and entropy values obtained at a single temperature (e.g., from isothermal titration calorimetry performed at a single temperature point as is the case in most other datasets) may be misleading when analyzing the temperature dependence of the interaction.*

*The distribution of dissociation constants at 25 and 37 °C for protein–protein interactions is shown in Supplementary Fig.1A. Notably, the same characteristic feature observed for the analogous distribution of DNA–DNA interactions (**Fig.1A**) is evident here: for a given K_d at 25 °C, there exist protein–protein pairs with substantially different K_d values at 37 °C.*

*Moreover, a similar pattern can be observed even in the case where one of the proteins remains unchanged while single-point mutations are introduced into the other. For instance, an analogous distribution is shown in **Supplementary Fig.1B** for a mutational scan*[2] *of an anti-HER2 antibody reported in Kelley (1993). Notably, the V_H-F100A and V_L-Y92A antibody variants exhibit nearly identical K_d values at 25 °C (1.7 and 2.4 nM, respectively), yet display markedly different K_d values at 37 °C (9769 and 452 nM, respectively), corresponding to a 21.7-fold difference.*

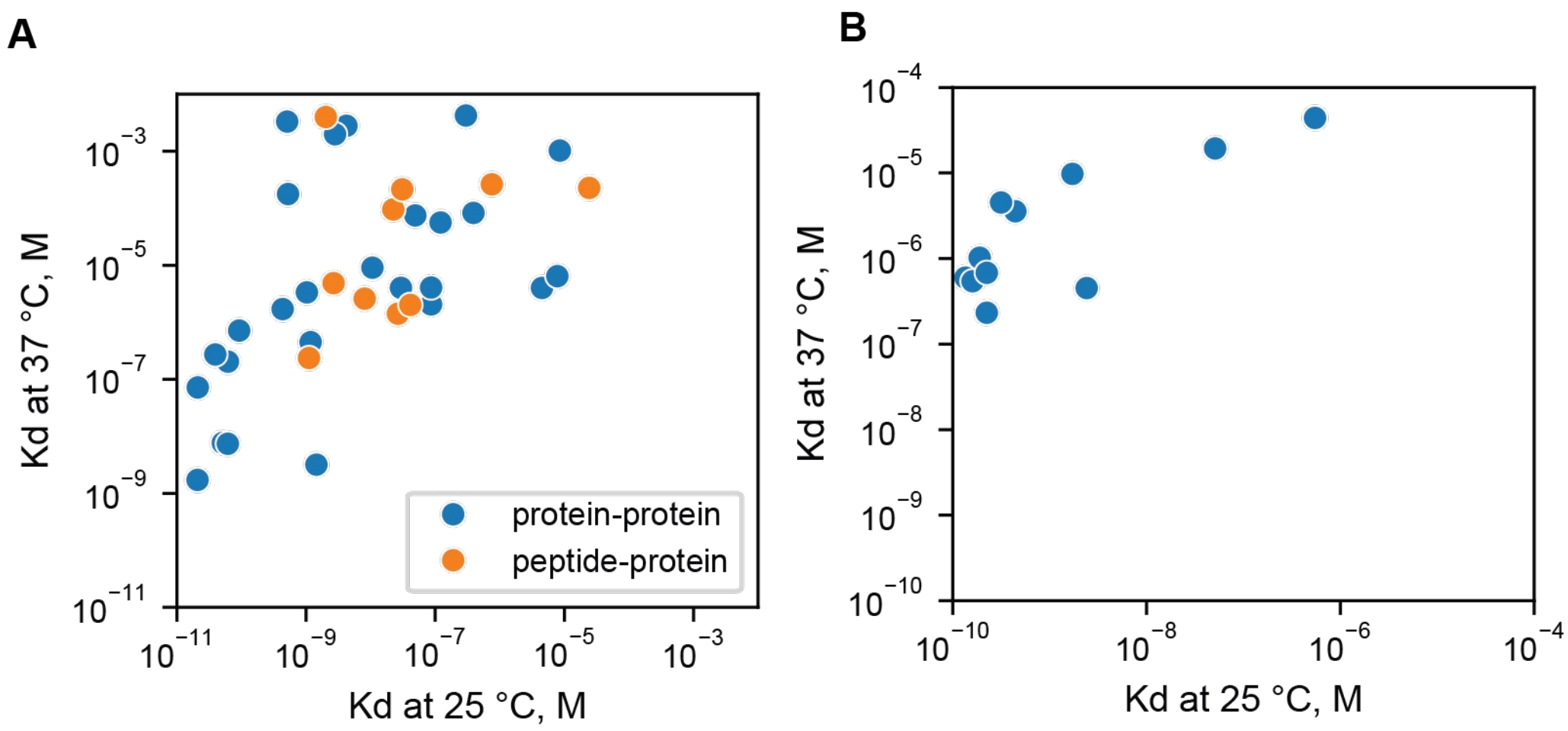

***Supplementary Fig.1**. Kd distributions at 25 and 37 °C for protein-protein interactions for (A) various protein-protein and protein-peptide pairs from Stites 1997*[1]*; for (B) single-point mutational scan of an anti-HER2 antibody from Kelley 1993*[2]*.*

…

1. Stites, W. E. Protein−Protein Interactions: Interface Structure, Binding Thermodynamics, and Mutational Analysis. Chem. Rev. 97, 1233–1250 (1997).

2. Kelley, R. F. & O'Connell, M. P. Thermodynamic analysis of an antibody functional epitope. Biochemistry 32, 6828–6835 (1993).”

Moreover, we cited several works in the Introduction on existing temperature-independent biological systems, including enzymatic networks (see our reply to the comment 1.1).

1.6 For example, similarly to the protein binding networks, the possibility of formation of complexes consisting of more than 2 strands need to be addressed. I guess this could be a real issue in the models presented in Figs 5 and 6.

Thank you for this important comment. Indeed, in many cases the simulation results differed when non-zero concentrations of multimeric complexes were taken into account. Therefore, for all systems relying on NUPACK computations, we performed an additional fine-tuning step of concentrations and sequences to achieve comparable performance when accounting for nonzero concentrations of such complexes. We have added the following explanation to the Concept chapter of the Results section and included new Supplementary Fig. 4–7, 10. Systems in the new Fig.7 were also designed to account for these higher-order complexes. Please note, that NUPACK simulations were not used in Figures 5 and 6 as they were only considered at the level of affinity matrices.

"Another simplification was the consideration of only complexes consisting of at most two strands. At the same time, real biomolecules (especially nucleic acids) are typically capable of forming higher-order complexes. However, accounting for such complexes does not introduce any fundamental changes and does not compromise the compensatory capabilities of the mechanism: Supplementary Figures 4–7 demonstrate equally robust performance of the YES, NOT, AND and 0.1-50 °C YES gates respectively when triple complexes are taken into account (NUPACK max_compl_size = 3), designed through simple fine-tuning of previously optimized sequences and concentrations."

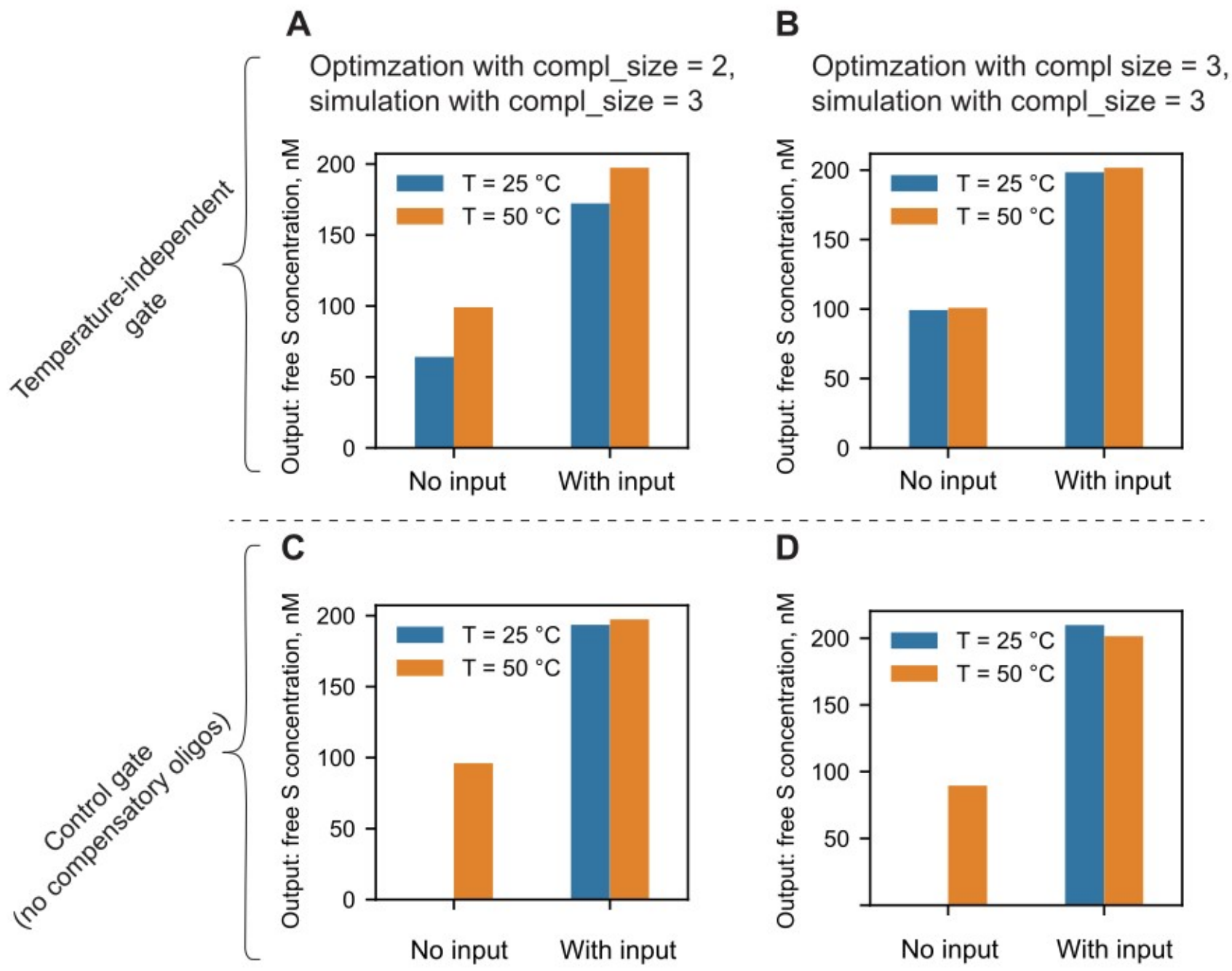

Supplementary Fig.4. *Performance of a temperature-independent and control YES logic gates, simulated in NUPACK while accounting for complexes with up to three strands (max_compl_size = 3). For (A), the exact gate parameters (sequences and concentrations) from Fig.2A were used, where only complexes with up to two strands were considered during optimization. Gate in (B) represents the same system from (A), fine-tuned to perform while accounting for complexes with up to three strands. (C) and (D) show simulations of control systems, identical to those in (A) and (B), respectively, but without compensatory species.*

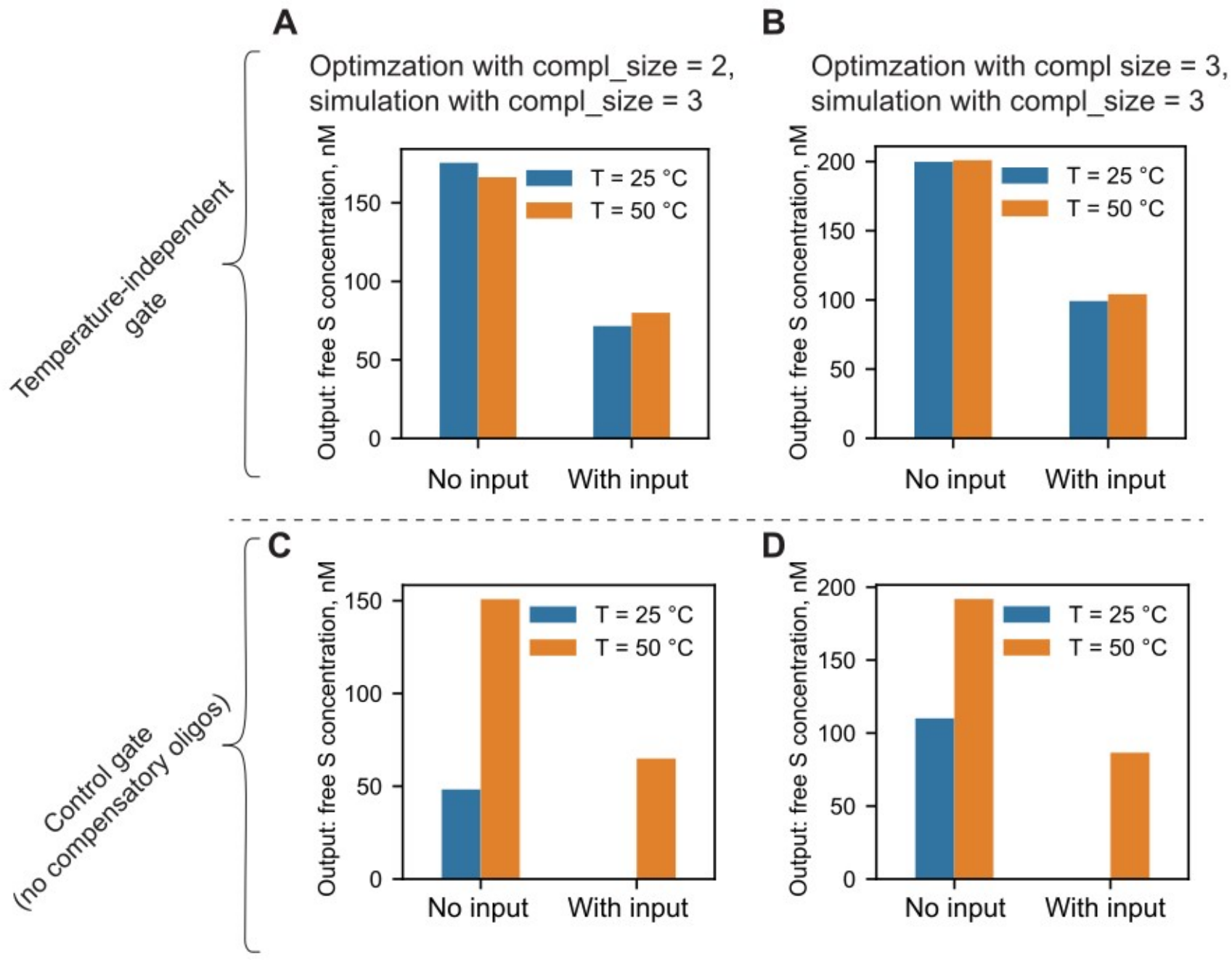

Supplementary Fig.5. *Performance of a temperature-independent and control NOT logic gates, simulated in NUPACK while accounting for complexes with up to three strands (max_compl_size = 3). For (A), the exact gate parameters (sequences and concentrations) from Fig.2B were used, where only complexes with up to two strands were considered during optimization. Gate in (B) represents the same system from (A), fine-tuned to perform while accounting for complexes with up to three strands. (C) and (D) show simulations of control systems, identical to those in (A) and (B), respectively, but without compensatory species.*

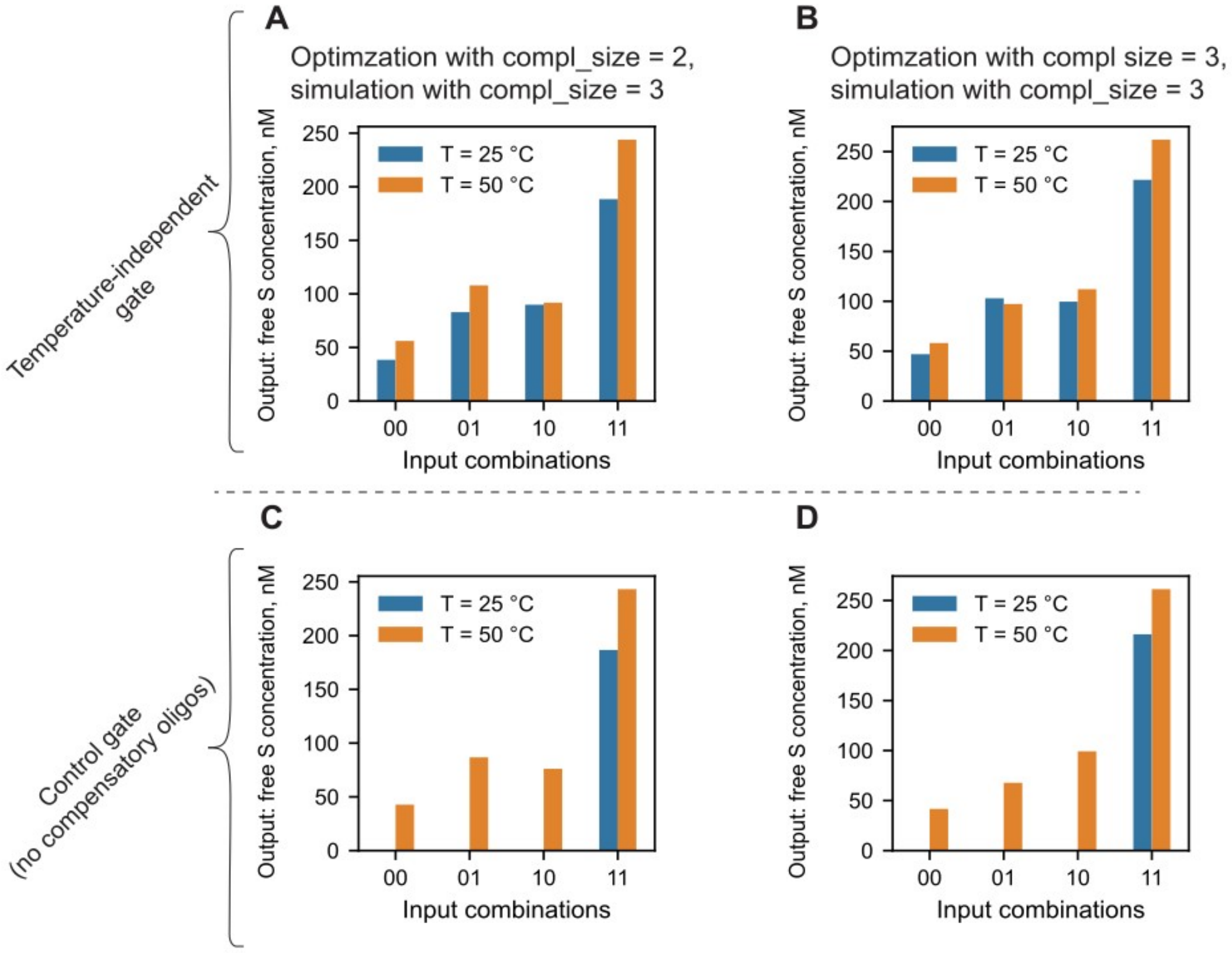

Supplementary Fig.6. *Performance of a temperature-independent and control AND logic gates, simulated in NUPACK while accounting for complexes with up to three strands (max_compl_size = 3). For (A), the exact gate parameters (sequences and concentrations) from Fig.2C were used, where only complexes with up to two strands were considered during optimization. Gate in (B) represents the same system from (A), fine-tuned to perform while accounting for complexes with up to three strands. (C) and (D) show simulations of control systems, identical to those in (A) and (B), respectively, but without compensatory species.*

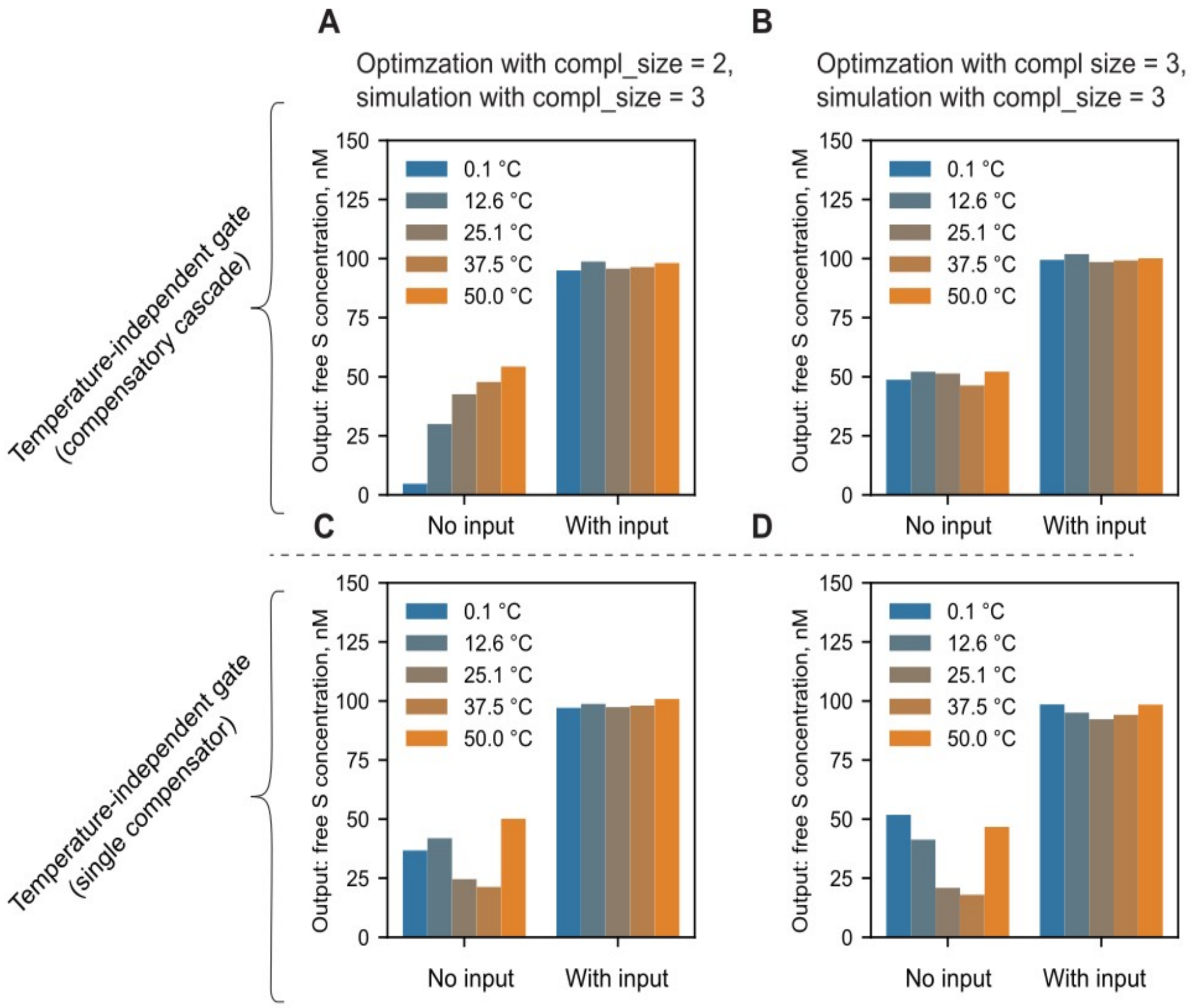

Supplementary Fig.7. *Performance comparison of a temeperature-independent YES logic gate with compensatory cascade and gate with a single compensator (0.1-50 °C range), simulated in NUPACK while accounting for complexes with up to three strands (max_compl_size = 3). For (A, C), the exact gate parameters (sequences and concentrations) from Fig.2E and D were used, where only complexes with up to two strands were considered during optimization. Gates in (B, D) represents the same systems from (A, C) respectively, fine-tuned to perform while accounting for complexes with up to three strands. (C) and (D) show simulations of control systems, identical to those in (A) and (B), respectively, but without compensatory species.*

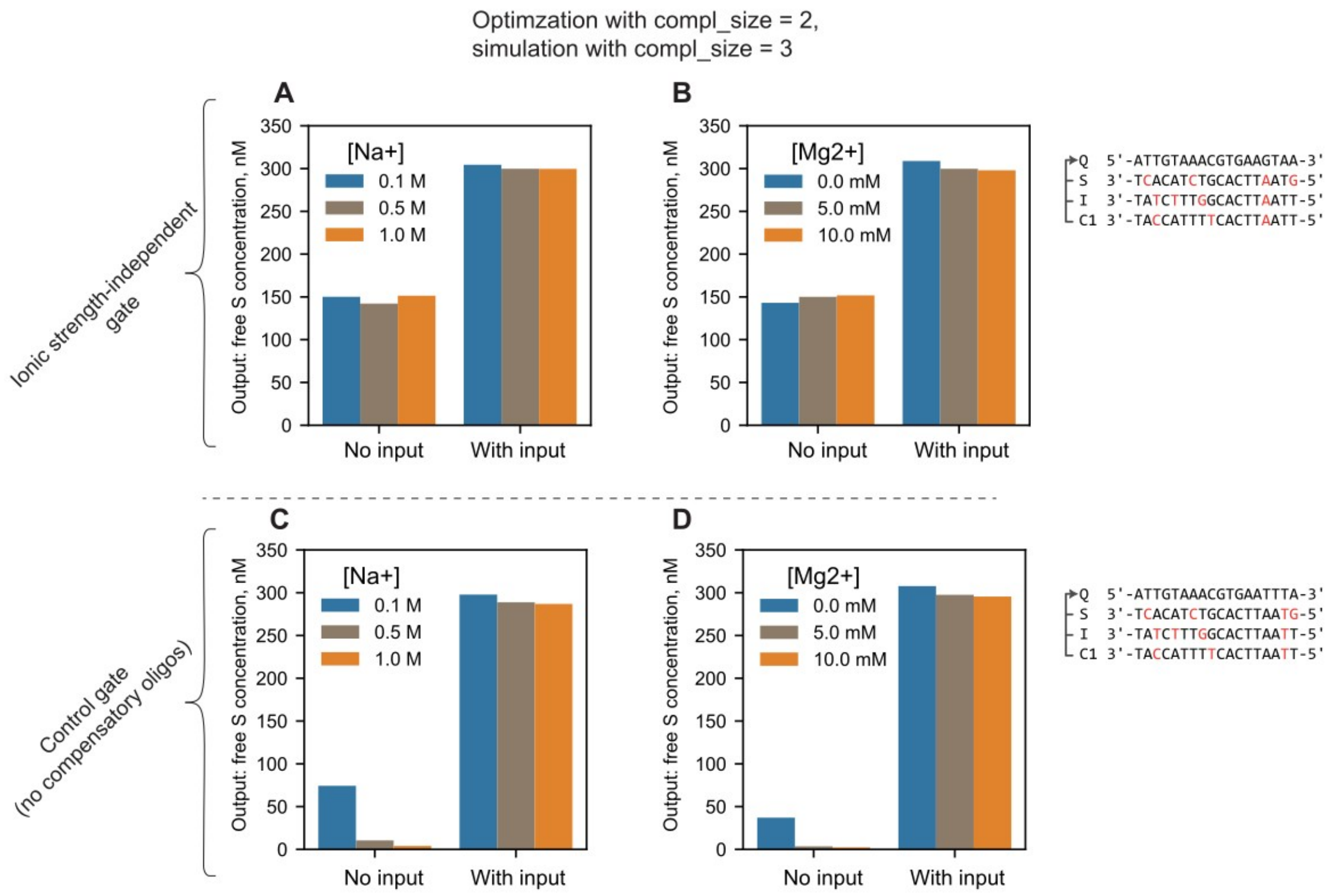

Supplementary Fig.10. *Performance of salt-independent and control YES logic gates, simulated in NUPACK while accounting for complexes with up to three strands (max_compl_size = 3). (A, C) show gate performance under Na^+ variation (Mg^{2+} concentration is zero), (B, D) show gate performance under Mg^{2+} variation (Na^+ concentration is 50 mM).* Please note that some sequences are written from 3′ to 5′ for demonstrating mismatches*"*

The description of the corresponding optimization procedure was added to the Methods section:

*"**System fine-tuning to account for non-zero concentrations of triple complexes.***

In all simulations involving NUPACK (Fig.2, Fig.7, Supplementary Figures 4-7, 10), an additional optimization step was performed to ensure that the equilibrium concentrations of the output oligonucleotide attained their target values for all possible input states when using max_compl_size = 3 and higher

The objective function at this stage was analogous to that used in the PyBADS optimization section. During this refinement procedure, evolutionary sequence optimization steps were alternated with Bayesian optimization of concentrations. The concentration-optimization parameters were identical to those used during the initial system design (listed in Supplementary Methods), with the exception of the maximum number of objective-function evaluations, which was limited to 100 (no such restriction was imposed at the initial stage).

For sequence fine-tuning, a population size of 192 was employed. At each evolutionary step, between one and three random mutations were introduced into each sequence with a probability of 50% per oligonucleotide."

1.7 The software that predicts dissociation constants, NUPACK, is used merely as a black box. It would be helpful to comment on how it works and why it is necessary to deconstruct the temperature-dependent free energy into the enthalpic and entropic parts while these parts are apparently computed separately inside NUPACK. Why the enthalpy and entropy

for binding between entire strands can be considered temperature-independent, or is the separation into H and S just a linear regression of the temperature dependence of the free energy.

We have added the following paragraph into the results section, and added Supplementary Note 3 (see below). Briefly, although NUPACK does not directly compute the enthalpy and entropy of the entire process, this representation is simple and sufficiently accurate for our purposes. For example, for the dataset used in Fig.1A, the approximation error in the worst case scenario (in terms of Gibbs energy) was less than 10%, and the median error was 0.7%.

"When not only endpoint temperatures are considered, accurate evaluation of binding parameters at intermediate temperatures becomes essential. During the intermediate stage of dissociation constants optimization, we assumed a linear dependence of the Gibbs free energy on temperature, despite the fact that NUPACK predicts deviations from this behavior in some cases (Supplementary Note 3). Nevertheless, at the final stage after the sequence design, simulations were performed in NUPACK (without such assumption)."

*"**Supplementary Note 3. Calculation of the hybridization temperature dependence using NUPACK.***

The principles underlying NUPACK are described in detail in the relevant publications[3]. Briefly, NUPACK considers the Boltzmann ensemble of all possible states of participating oligonucleotides. For each possible state, NUPACK calculates the Gibbs free energy, using the nearest-neighbor model with various corrections, such as loops, stacking interactions, dangling ends, and others. In this model, enthalpy and entropy of each individual state are assumed to be temperature-independent. The resulting information is then aggregated over all states by computing the partition function for each complex.

Let us consider a simple system consisting of two strands, s_1 and s_2. We restrict the analysis to the complexes (s_1), (s_2), and ($s_1 + s_2$), as the concentrations of all other complexes are negligible by design.

If:

1. *Single-stranded species exist mostly as free strands (i.e., structures with non-zero free energy have negligible low concentrations);*
2. *The most favorable duplex state is significantly lower in free energy than all alternative states;*

then the hybridization reaction can be described by only two parameters, (for example, enthalpy and entropy) and dependence of reaction Gibbs free energy on temperature will be linear.

To test this assumption for the oligonucleotides used in this study, we analyzed the same dataset as in Fig.1A. To exclude extreme cases, we considered oligonucleotide pairs with Gibbs free energy at 25 °C ranging from −30 to −80 kJ/mol. For these 4,684 oligonucleotides, we computed the dependence of $\Delta G(T)$ over the temperature range from 25 to 50 °C.

We then estimated apparent enthalpy and entropy from 25 and 50 °C temperature endpoints using simple linear approximation and evaluated the maximum error across all intermediate temperatures arising from predicting ΔG in this manner. The median error was 0.31 kJ/mol, while the maximum error reached 2.6 kJ/mol (0.7% and 10% for the relative error). This supports the validity of the approximate description of the hybridization reaction using only two parameters. In addition, it should be noted that this approximation serves only as an

intermediate step to simplify the identification of nucleic acid sequences and, therefore, does not require particularly high accuracy."

1.8 Given the pure statistical nature of several models analyzed, would a random distribution of dissociation constants (or free energies) of a particular type and temperature correlation (such as shown in Fig 1 A and B) serve as an equally convincing example?

Yes, exactly. The only requirement imposed on this distribution (K_d at 25 °C vs K_d at 50 °C) is that it has a nonzero width; that is, for similar K_d values at one temperature, there must exist a sufficiently diverse set of molecules exhibiting substantially different K_d values at another temperature. We have mentioned this in the Discussion:

"With regard to the fundamental feasibility of compensation in such networks, the only property of oligonucleotide networks that we explicitly relied upon is that the behavior of the complex formation reaction at low temperatures does not fully determine its behavior at higher temperatures."

Moreover, for nucleic acids, this assumption is true according to experimental data, for example, see the screening experiment in Fig.3B, through which we identified pairs of oligonucleotides exhibiting precisely this property (and which were subsequently used to implement the experimental YES gate). The same holds for protein–protein interactions (see the response to Comment 1.3).

1.9 The term "computation" has become way too overloaded and is often applied to any system that has identifiable input and output and exhibits some kind of non-linear response between them. In my opinion, in the text, the term "computation" is misleading and even sounds like a false promise. While various non-linear and combinatorial functions can be implemented using a mass-action networks, those networks are intrinsically limited by the spatial decay of the signal, generally exponential, and the associated with it reduction in the range of binary or continuous signals. So unless the Authors prove the opposite, I cannot see how a computer can be built using such a network. I strongly suggest to tone it down and replace "computation" and adjectives that are derived from it by a less flashy but more realistic terms like "signal transformation" or "signal processing".

We agree that in many studies the term *biocomputing* is used in the sense you described. However, from our perspective, a more appropriate use of this term refers to systems capable of performing a broad range of signal-processing operations within a single framework, for example, implementing a complete set of Boolean logic functions. For instance, the detection of a heavy metal ion via fluorescence quenching of quantum dots should not be described as implementing a NOT gate, as it is not clear how such a system could be extended to realize all other logical operations.

In contrast, in our case, remaining entirely within the paradigm of affinity-based interactions (molecular commutation principle), an extremely wide spectrum of computations is possible. In addition to being one of the very few paradigms that enable the implementation of a complete set of Boolean functions, molecular commutation remains the only approach that allows the solution of elementary algebraic problems and the construction of ultra–high-input gates (as demonstrated in our 2023 Nature Chemistry paper). Furthermore, this term is essential in the context of the new results (Fig.7) on programmable temperature dependence, as it highlights the capacity of molecular commutation systems to implement *predefined* responses such as those *computed* as elementary algebraic functions.

For these reasons, and given that *biocomputing* is the established term for the field, we prefer to retain it in this narrower and more rigorous sense, to ensure that other researchers can readily identify this work and evaluate its contributions relative to the current state of the art.

At the same time, we acknowledge that the term should not be used unnecessarily, and have therefore replaced it with alternative terms in the majority of instances throughout the manuscript. However, we have retained the original terminology in key contexts, such as in the introduction of the molecular commutation phenomenon, as well as when referring to prior works in which this term has been explicitly used.

1.10 The phrase in lines 71-74 is hard to parse. It's also not clear what the authors mean under the term "integrator".

We have expanded the description of what we refer to as a signal integrator, provided the corresponding examples, and simplified the sentence in question:

"In such systems, one of the key factors is the presence of "signal integrators": elements that combine multiple inputs into a single output. For example, in the olfactory receptor system[13] the integrator is central nervous system that analyzes signals from many receptors (inputs) to give the sense of a certain smell (a single perceptual output). Similarly, dimerizing transcription factors system[12] require presence of a specific "integrator" enzyme, which can be phosphorylated or dephosphorylated by multiple network components (inputs) with a very particular single output: the concomitant change in catalytic activity.

... in other words, in this computation paradigm any molecule can act as a signal integrator, and no higher-level signal integrator systems (such as the abovementioned enzymes or brain cell networks) are required."

1.11 The specificity and limitations of the oligonucleotides dimerisation network should be discussed and compared to the protein-protein LMA networks.

The following section was added to the Discussion:

*"**Topological compensation in protein-protein networks***

In our study, we demonstrate the realization of a compensatory mechanism using nucleic acids as an example. We believe that the demonstrated here results may be extrapolated to other affinity-based molecular networks, including, of course, small molecule and protein networks[41,42].

With regard to the fundamental feasibility of compensation in such networks, the only property of oligonucleotide networks that we explicitly relied upon is that the behavior of the dimerization reaction at low temperatures does not fully determine its behavior at higher temperatures. Evidently, even with the increased complexity of processes encountered in protein systems, this key assumption remains valid (see Supplementary Note 1). Furthermore, we do not see any conceptual limitations for this idea to be implemented within the mixed networks of nucleic acids, proteins, small molecules, etc.

Moreover, protein networks possess the potential for even more complex behavior. In contrast to oligonucleotides, proteins undergo temperature-dependent folding and denaturation processes, exhibit intricate cooperative effects, and their free energy typically demonstrates a strongly nonlinear dependence on temperature. We believe, that the presence of more complex processes does not complicate the problem of compensation; rather, it provides nature with a broader repertoire of mechanisms for its implementation.

It is also interesting to consider a special case of protein interactions, namely, enzymatic networks, which are regarded as the primary source of temperature-independence in some naturally occurring systems (such as circadian rhythms[18]). We, however, believe that affinity-

based networks have many important advantages over enzymatic networks in terms of their compensatory capabilities. For an enzymatic network to be temperature-independent, not only must activation energies be tuned in a precise manner, but also other more critical parameters, such as substrate selectivity and overall catalytic efficiency, so that each enzyme remains functional. All of these parameters are governed by substrate–enzyme interactions and are typically controlled by only a few amino acid residues. Therefore, fulfilling the compensation requirement (i.e., matching activation energies) is inherently difficult. In contrast, in a general case of an affinity-based network, where binding thermodynamics is largely decoupled from such complex functional constraints, the compensatory behavior is considerably easier to achieve (whether through natural evolution or artificial engineering), as much fewer parameters must be optimized simultaneously. i.e., only the binding affinities themselves and the concentrations."

1.12 Line 102, should it be an em (emphasis) dash instead of a hyphen?

Yes, this is a typo. Corrected.

1.13 Line 141 "that" -> "this"?

Corrected.

1.14 Fig. 1E, are homodimeric dissociation constants chosen randomly to be large and thus irrelevant?

Yes. At the stage of numerical parameter optimization, certain interactions, namely all those not represented in the logical gate diagram (specifically, homodimerization and the I–C, S–C, and I–S interactions in the case of the YES gate), are preferred to be as weak as possible (corresponding Kd = inf). At the sequence design stage, sequences are selected so that the energies of these "forbidden" interactions do not exceed a specified threshold. We have added further details on sequence optimization to the Methods section.

"Sequence finding optimization

Sequence selection was performed using an evolutionary algorithm using DEAP python library[53]. At this stage, the target Gibbs free energies $\Delta G_{ij}^{expected}$ of hybridization at the specified temperatures (typically 25 and 50 °C) are assumed to be known for all pairs of oligonucleotides in the system.

The algorithm proceeds iteratively, constructing one oligonucleotide at a time. The first oligonucleotide is initialized randomly.

We now describe the procedure for designing the N-th oligonucleotide, assuming that the preceding N−1 oligonucleotides have already been determined. The initial guess for this oligonucleotide was defined as the reverse complement of the oligonucleotide, that must exhibit the strongest binding affinity to the sequence being designed:

$$seq_N^{start} = rev.compl\left(seq_m\right), \; m = \underset{j=1\ldots N-1}{argmin}\left(\Delta G_{jN}\right).$$

The sequences in the initial population were generated from this starting sequence by introducing between one and four random mutations. Subsequently, during the evolutionary process, each oligonucleotide was either modified by introducing between one and six random mutations or reinitialized by applying one to two random mutations to the starting sequence. After evaluating the objective function for all members of the population, the next generation was constructed using random tournament selection with a tournament size of three.

The objective function was defined as the sum of absolute deviations between the Gibbs free energies of hybridization (at all target temperatures) with previously N-1 found oligonucleotides evaluated by NUPACK and the corresponding target Gibbs energy values:

$$L = \sum_{t \in \{25°C, 50°C\}} \sum_{i=1...N-1} \left| \Delta G_{iN}^{expected}(t) - \Delta G_{iN}^{NUPACK}(t) \right|.$$

In cases where no interaction between oligonucleotides i and j was desired (i.e., off-target interaction), the Gibbs free energy of hybridization of the selected sequences was required to remain below a threshold defined as follows. The dissociation constants of off-target interactions had to be at least k_atten-fold weaker than the weakest dissociation constant among all target interactions (as determined in the previous parameter-optimization step). If an off-target interaction was weaker than this threshold, it contributed zero to the loss function; otherwise, a large penalty was imposed that was guaranteed to exceed all other terms in the loss function.

The parameter k_atten was specified independently for low and high temperatures and denoted as lowtemp_ka_atten and hightemp_ka_atten, respectively. Additionally, if the self-dissociation constant of an oligonucleotide was stronger than 5×10^{-5} M, a penalty term exceeding all other components of the loss function was added. Finally, the loss function included the absolute value of the Gibbs free energy of secondary structure formation for the given oligonucleotide, divided by the parameter hairpin_atten.

Unless otherwise specified, the following optimization parameters were used: sequence length = 20, population size = 280, number of generations = 100, lowtemp_ka_atten = 10^5, hightemp_ka_atten = 10^4, hairpin_atten = 5, early stop condition = 1 kJ/mol.

If, upon reaching the maximum number of generations, the error still exceeded the threshold, all N−1 sequences identified up to that point were discarded, and the optimization was restarted from scratch."

1.15 Line 253, 100 nM vs. line 267, 1 µM.

Value of "100 nM" refers to experiment in Fig.3A, while "1 µM" refers to data in Fig.3B. On the other hand, the value of 100 nM is the actual concentration used in the experiment and the value of 1 µM in the sentence "we plotted the results as the equilibrium concentration of the IQ duplex, assuming both strands were initially present at 1 µM" is employed as a custom affinity metric that depends on the initial concentration. We have revised the latter sentence to eliminate the ambiguity:

"The affinity was plotted in custom units consistent with prior work[21], namely as the equilibrium concentration of the IQ duplex as if both strands are mixed at 1 µM concentration"

1.16 "Output concentration" in the vertical axis of Fig. 2 vs. "Free S concentration" in Fig. 3

We have revised all axis labels to read "Output: free S concentration, nM"

1.17 Line 572 "describing the fundamental algorithms of the service"?

The line has been change to *"The equilibrium constants were computed according to Dirks et al.[51]"*

1.18 Fig. 3 and the related discussion: One can argue that the non-compensated YES gate (as well as modules considered in subsequent examples) performs even better than the "temperature-independent" gate: While the difference between the signal and no-signal

levels of S is identical at 50 degrees, this difference is more pronounced at 25 degrees for the gate with no C.

To clarify this, we have added the following discussion to the Results section:

"We should also note that, for example, for the compensated YES gate (Fig.2A) the True-False signal difference is not greater than that in the non-stabilized case at their respective optimal temperatures. This reflects the classic "robustness over peak performance" trade-off, a well-known concept in biotechnology (e.g., enzyme engineering[35,36]) where expanding the operational range and ensuring stability is typically more advantageous than maximizing performance within a narrow parameter window. On the other hand, this effect arises from the gate construction strategy: the control and compensated gates were designed to operate identically at 50 °C (as compensators are "easy-to-melt"). However, if we consider the gates that have identical outputs at 25 °C, then the True-False difference at 50 °C becomes significantly greater for the compensated gate. Moreover, if the objective function is this True-False separation (instead of the preservation of absolute concentrations), the gate design problem becomes substantially simpler. In this case, virtually any YES gate with a topology that has a compensatory component binding Q (at a concentration comparable to that of the other components) will outperform a YES gate lacking such a compensator, provided that both exhibit identical performance at 25 °C (i.e., the same level of free S). This behavior is illustrated for both YES and NOT gates in Supplementary Note 2 and Supplementary Fig. 3

...

34. Nezhad, N. G. et al. Recent advances in simultaneous thermostability-activity improvement of industrial enzymes through structure modification. Int. J. Biol. Macromol. ***232****, 123440 (2023).*

35. Vanella, R. et al. Understanding activity-stability tradeoffs in biocatalysts by enzyme proximity sequencing. Nat. Commun. ***15****, 1807 (2024)."*

To substantiate this claim, we have performed additional simulations, as described in Supplementary Note 2 and Supplementary Fig. 3.

"***Supplementary Note 2. Construction of temperature-compensated systems without parameter optimization across the entire temperature range***

In this section, we demonstrate that the topology alone of signal-processing systems with compensatory species is sufficient to achieve partial temperature compensation.

*Let us consider the topology of a YES gate in its two variants: the first one without compensation (three oligos I, Q, S), and the second with compensation (four oligos I, Q, S, C). For the second one to be conceptually different from the first one, the concentration of C and the affinity of Q–C should not be zero or negligibly small, so let's fix these values to some reasonable values, for example, concentration of the compensator C to 5 µM and the dissociation constant of Q–C at 25°C to 1.7 nM (−50 kJ/mol). Now, consider random gates of these two topologies that have the same level of free S at 25 °C, for example, 100 and 200 nM in the absence and in the presence of input, respectively. Gate performance is typically defined as the difference between outputs in the True and False states (i.e., with and without input), which in this case is 100 nM (****Supplementary Fig. 3A, D****). How would the True-False difference behave at 50 °C for non-compensated and compensated groups of gates?*

We have generated 100 distinct parameter sets for both groups of gates. ***Supplementary Fig. 3B,C*** *presents the distributions of the True-False differences for these groups of gates at 25 and 50 °C. It is evident that the True-False difference at 50 °C is substantially higher in the compensated system, whereas for the majority of control systems it is close to zero; that is, the states with and without input at 50 °C are nearly indistinguishable. Similarly, we performed the same analysis for the NOT gate. The results are presented in* ***Supplementary Fig. 3E,F****, where the same pattern is observed. Thus, an appropriate system topology can ensure temperature stabilization even if its parameters are not optimized for operation at temperatures other than the reference one.*

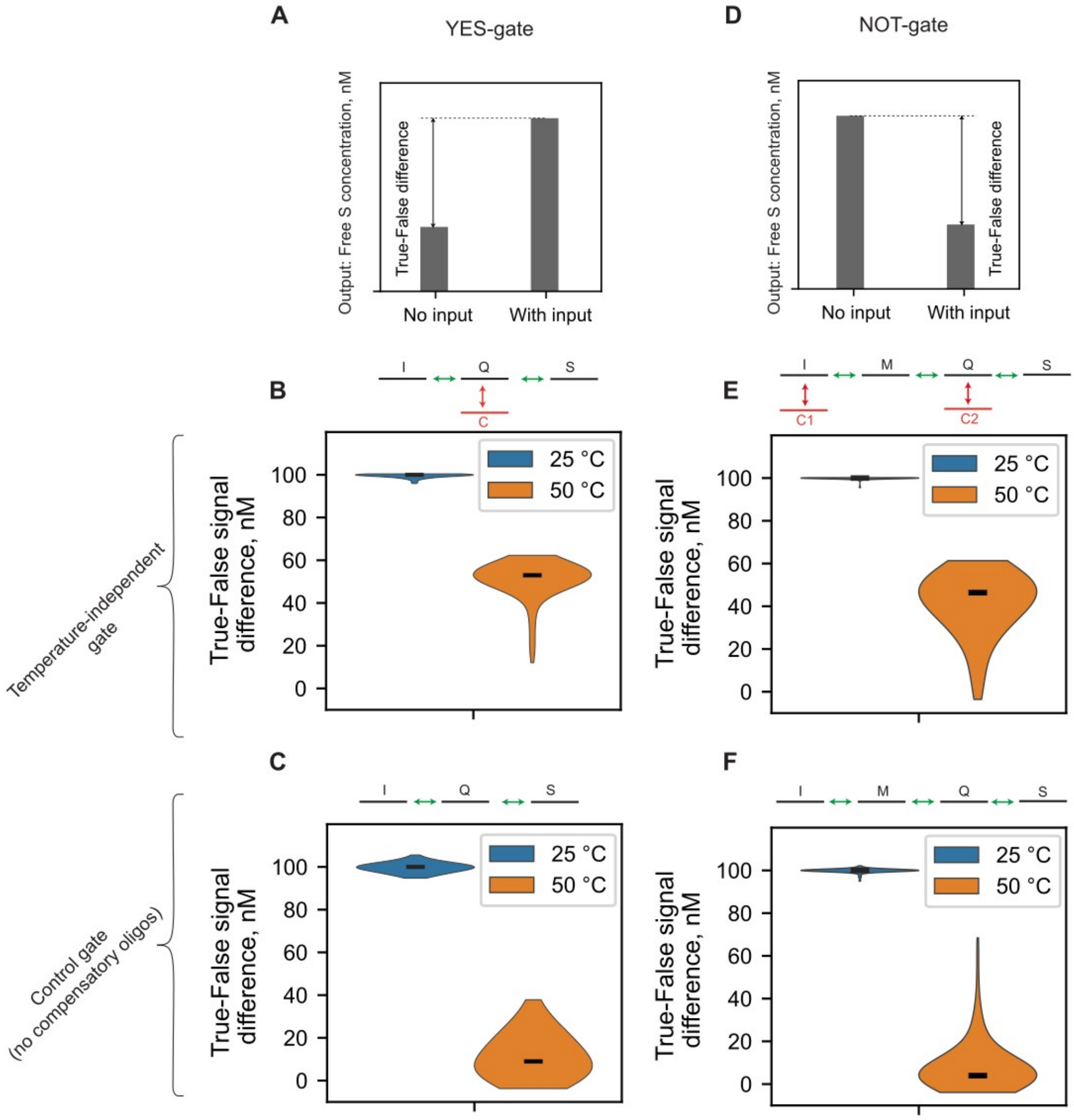

Supplementary Fig. 3*. Sufficiency of compensatory topology alone for stabilizing logic gates under temperature variation. (A, D) Visualization of True-False signal difference for YES and NOT gates. Distribution of this True-False signal difference (i.e., with inputs vs. without inputs) at 25 and 50 °C is shown for 100 (B) YES gates and (E) NOT gates. Panels (C, F) show the same distribution for the control YES and NOT gates (i.e., without compensatory oligos). Black lines show the median values. See Supplementary Note 2 for a detailed description."*

1.19 (Remarks on code availability) Since the modeling involves several pre-packaged codes, it's hard to assess how the results are affected by the programmes written by the authors.

Regarding the programs developed in this work, the full source code is available as a Supplementary File.

As concerns pre-packaged codes, indeed, one of the key dependencies of our work is NUPACK, and we rely on its calculations. We note that NUPACK is a widely accepted tool in the field, its source code is available, and its algorithms are described in detail in numerous papers by the developers of the service, for example:

- Dirks, R. M.; Pierce, N. A. A partition function algorithm for nucleic acid secondary structure including pseudoknots. J. Comput. Chem. 2003, 24, 1664– 1677, DOI: 10.1002/jcc.10296
- Dirks, R. M.; Lin, M.; Winfree, E.; Pierce, N. A. Paradigms for computational nucleic acid design. Nucleic Acids Res. 2004, 32, 1392– 1403, DOI: 10.1093/nar/gkh291
- Dirks, R. M.; Pierce, N. A. An algorithm for computing nucleic acid base-pairing probabilities including pseudoknots. J. Comput. Chem. 2004, 25, 1295– 1304, DOI: 10.1002/jcc.20057
- Dirks, R. M.; Bois, J. S.; Schaeffer, J. M.; Winfree, E.; Pierce, N. A. Thermodynamic analysis of interacting nucleic acid strands. SIAM Rev. 2007, 49, 65– 88, DOI: 10.1137/060651100
- Zadeh, J. N.; Wolfe, B. R.; Pierce, N. A. Nucleic acid sequence design via efficient ensemble defect optimization. J. Comput. Chem. 2011, 32, 439– 452, DOI: 10.1002/jcc.21633
- Wolfe, B. R.; Pierce, N. A. Sequence design for a test tube of interacting nucleic acid strands. ACS Synth. Biol. 2015, 4, 1086– 1100, DOI: 10.1021/sb5002196
- Wolfe, B. R.; Porubsky, N. J.; Zadeh, J. N.; Dirks, R. M.; Pierce, N. A. Constrained multistate sequence design for nucleic acid reaction pathway engineering. J. Am. Chem. Soc. 2017, 139, 3134– 3144, DOI: 10.1021/jacs.6b12693
- Fornace, M. E.; Porubsky, N. J.; Pierce, N. A. A unified dynamic programming framework for the analysis of interacting nucleic acid strands: Enhanced models, scalability, and speed. ACS Synth. Biol. 2020, 9, 2665– 2678, DOI: 10.1021/acssynbio.9b00523
- Nanjundiah, A.; Fornace, M. E.; Schulte, S. J.; Pierce, N. A. Models and algorithms for equilibrium analysis of mixed-material nucleic acid systems. ACS Synth. Biol. 2026, 15, 74– 87, DOI: 10.1021/acssynbio.5c00470

Regarding the correctness of our use of NUPACK, we would like to note that Reviewer #2, based on the sequences we reported, conducted an independent simulation for Fig. 4B (in the revised manuscript this is Fig. 2E), and the results fully matched ours (when they used the same value of max_compl_size = 2 as we did originally) – see Reviewer's #2 comment 2.5:

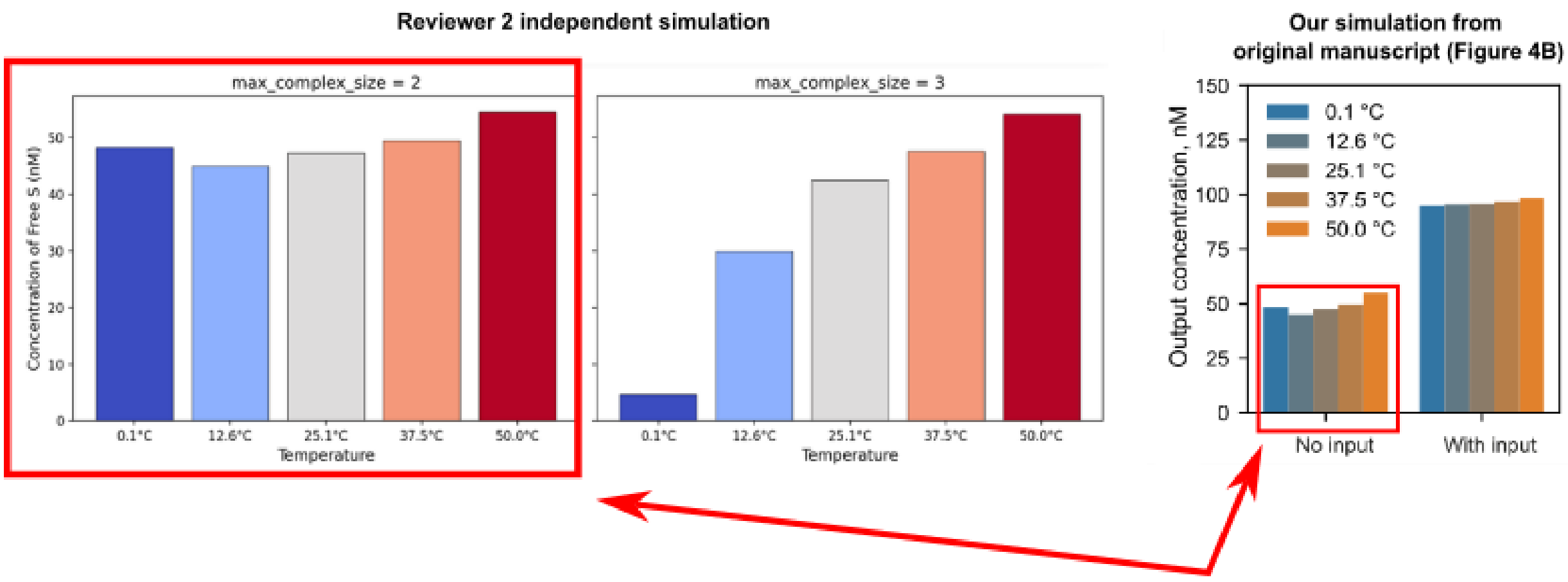

Moreover, precisely because NUPACK predictions are not perfect, we performed experimental validation of some of the obtained results (Fig. 3C, new Fig. 4C, D) and showed that, despite the observed discrepancies between predictions and experimental data, the compensation phenomenon is still clearly observed.

Other major dependencies include optimization packages, namely PyBADS (Bayesian optimization) and DEAP (evolutionary optimization). However, the correctness of these packages affects only the speed of convergence to the final result, while this final result is then tested and verified within our code without using non-standard black-box prepackaged libraries.

To the Reviewer 2

I greatly appreciate the overarching goal of this work. The manuscript introduces a compelling and broadly useful design principle (which the authors call topological temperature/ionic-strength compensation) that could expand the toolkit for robust molecular circuit design and, more speculatively, suggest organizing principles for natural regulatory networks. While it is well known that nucleic-acid hybridization (eg the nearest-neighbor model) involves substantial enthalpic and entropic contributions, I am not aware of prior work using the discrepancy between these contributions as a design lever. The central idea, that of achieving temperature- and ionic-strength–insensitive behavior, is highly impactful both for molecular engineering and for thinking about principles that could operate in biological regulatory networks. If generalizable beyond DNA, this approach could enable more reliable molecular computation across fluctuating environmental conditions and provide a concrete framework for reasoning about compensation mechanisms in biochemical networks.

Thank you very much for your positive feedback on our work and for such a thorough evaluation of the manuscript. We are grateful for the constructive suggestions that helped improve the manuscript, and especially for your careful efforts in independently validating our code.

Below, we provide a point-by-point response. However, following your comments and being inspired by all the Reviewers, we have performed an additional study that generated a very interesting result: affinity-based systems can be designed to exhibit virtually any predefined temperature or ionic strength dependence (not just be independent from them as we showed in the initial version of the manuscript):

*“**Programmable temperature/ionic strength dependence***

After thoroughly discussing temperature independence (i.e., a dependence reduced to a constant), we proceed to the more general task of designing systems with arbitrary dependence on temperature or ionic strength. Such behavior could be beneficial, for example, for inducing signals of arbitrary forms in different cell types as body temperature rises during disease.

Another illustrative example of where such systems may be important is in ensuring temperature independence of a higher-level molecular system with its own complex temperature dependence. For example, consider temperature-independent bacterial chemotaxis. In this well-studied system[19] the key determinants of this independence include (1) the enzymatic network and (2) the mRNAs encoding these enzymes together with the translation-related machinery. The latter ensures a selective and tightly controlled increase in the expression of specific components of the enzymatic network as temperature rises.

*In turn, as demonstrated in our prior work[21], there exist mechanisms by which molecular commutation can significantly influence protein translation levels. Specifically, binding of DNA oligonucleotides to a specific site on an mRNA molecule may lead to its cleavage mediated by RNAse H, thereby reducing the expression of the corresponding protein (**Fig. 7A**). Consequently, free mRNA levels (i.e., mRNA not bound to oligonucleotides), representing the output of the nucleic acid affinity-based network, may directly determine protein concentrations.*

*Thus, suppose that a certain complex biochemical system requires, for its temperature independence or other higher-level signaling, a specific temperature-dependent profile of the level of free mRNA (**Fig. 7B**). In this section we show that it is possible to identify sets of DNA*

oligonucleotides that realize predefined complex, non-monotonic dependencies on temperature or ionic strength.

The formal statement of the problem is analogous to that of the previous section: the variable Y (the level of free mRNA) is defined in exactly the same way, whereas the variable X is defined as the normalized and centered temperature:

$$X = \frac{T - T_{min}}{T_{max} - T_{min}}.$$

The identification of DNA sets having a predefined temperature dependence Y(X) was carried out in a manner similar to the design of logic gates. We first fixed the sequence of the "output oligonucleotide" as the AUG-containing region of the firefly luciferase mRNA that was used in our prior study[21] for the experimental demonstration molecular commutation effect on protein expression.

For each specified function Y(X), the required dissociation constants, "easy-to-melt" levels, and concentrations of the components were first determined using Bayesian optimization. After that, DNA sequences realizing these parameters were identified using an evolutionary algorithm. Finally, an evolutionary fine-tuning of both sequences and concentrations was performed to ensure that the system exhibited the desired behavior while accounting for nonzero concentrations of triple complexes (NUPACK max_compl_size = 3).

This procedure was carried out for four sample dependencies Y(X): linear, quadratic, cubic, and sinusoidal. The resulting DNA sets, their interaction graphs, and their temperature dependencies are presented in ***Fig. 7C–F****.*

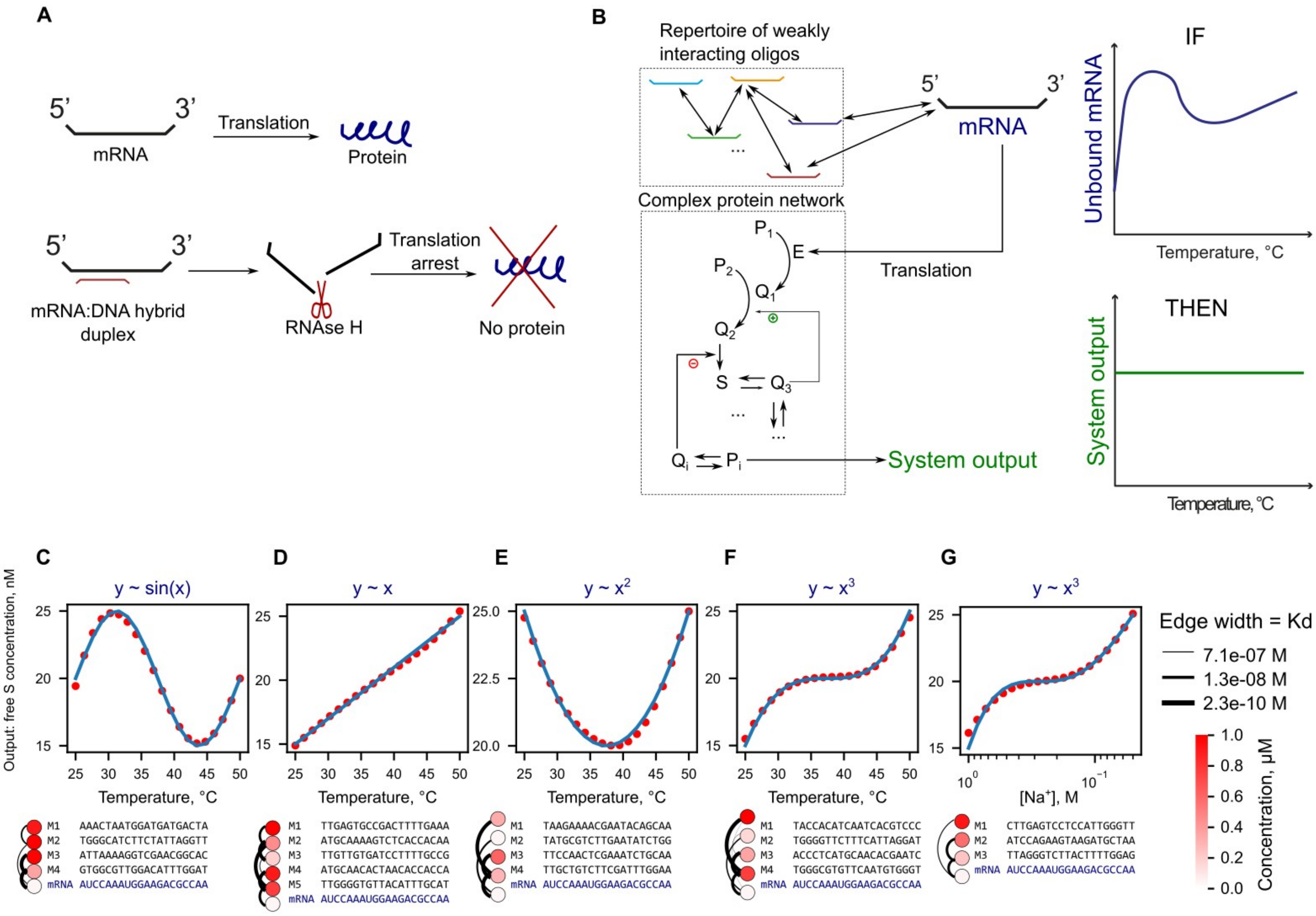

***Fig.** 7. Molecular commutation systems with programmable temperature/ionic strength dependence. (A) Schematic of translation control via an RNAse H-mediated mechanism. (B) Scheme of a nucleic acid molecular commutation system that compensates for the complex behavior of a protein network. Temperature compensation of the protein network requires a predefined level of protein expression, which is regulated by mRNA levels. (C–F) Performance and interaction graphs of molecular commutation systems exhibiting linear, quadratic, cubic, and sinusoidal temperature dependence. (G) Performance and interaction graph of molecular commutation system exhibiting cubic temperature dependence on ionic strength. X axis direction is reversed in (G). Solid lines represent the objective profile, and dots represent the simulated system performance. Node colors represent oligonucleotide concentrations, and edge widths represent the corresponding dissociation constants. DNA-RNA interaction was simulated according to DNA-DNA NUPACK model. The simulation was performed taking into account complexes consisting of up to three strands (NUPACK max_compl_size = 3).*

*In addition, **Fig. 7G** shows that nucleic acid networks can exhibit similarly complex, predefined dependencies on ionic strength. Since the free energy of hybridization depends logarithmically[40] on sodium concentration (in contrast to its linear dependence on temperature), for consistency, we chose the logarithm of the normalized sodium concentration as the independent variable (X):*

$$X_{ionic} = \log_{10}\left(a\left[Na^{+}\right]+b\right),$$

the constants a and b were chosen such that the sodium concentration range from 50 mM to 1 M is mapped onto the interval [0, 1] for X. Using a DNA set previously optimized to produce a

cubic dependence on temperature (see Materials and Methods for details), we applied an evolutionary algorithm to identify a new set that realizes an analogous dependence on ionic strength.

To summarize, this remarkable feature of the molecular commutation networks allows them to receive a single cue of the environment (e.g., temperature or ionic strength) and react in many different ways. This phenomenon seems to be a perfect match for a hypothesis that the elevated body temperature during disease can act as a universal information carrier to all cells of the organism, potentially capable of triggering, in each cell type, its own multitude of independent signaling profiles. Initiating these programmable responses via temperature may be highly valuable since it does not dependent on mass transport of molecular messengers throughout the body.»

In our view, such programmability of temperature dependence in molecular networks has potential relevance across an even broader range of systems, either as a compensatory mechanism or as a standalone signaling tool.

Accordingly, we have changed the title and the abstract to reflect these new results. The new title is "*Temperature- and ionic strength-independent or programmably dependent molecular signaling enabled by affinity-based molecular commutation networks*", and the new "Here we show" statement of the abstract is "*Here, we show that molecular commutation enables complex signaling systems that are independent of temperature and ionic strength and, even more generally, programmably dependent on these parameters.*"

2.1 That said, the presentation would benefit substantially from making the thermodynamic intuition explicit much earlier. The manuscript relies on the different temperature dependence of entropic versus enthalpic contributions (i.e., that the same ΔG at one temperature can arise from different ΔH/ΔS combinations, leading to different behavior across temperatures), but this framing is not clearly emphasized until late in the Discussion. I recommend stating this up front in the Introduction and including the basic relationship ΔG(T) = ΔH − TΔS early on. I would also encourage the authors to cite prior DNA molecular programming work that leverages entropy/enthalpy tradeoffs, even if those examples involve different notions of entropy (e.g., numbers of complexes) than the nearest-neighbor hybridization contributions emphasized here (eg, refs. [a,b] below).

We have inserted the proposed excerpt at the very beginning of the concept description.

"A well-known fact is that two DNA duplexes may have the same affinity at a room temperature, but substantially different melting temperature (in other words, different affinity at another temperature). More formally, affinity (e.g., dissociation constant, K_d) is determined by the Gibbs free energy ($K_d = e^{\frac{\Delta G}{RT}}$), which in turn is related to the enthalpy and entropy of binding $\Delta G = \Delta H - T \Delta S$. The different behavior of Kd at two temperatures arises from the relative independence of enthalpy and entropy. For example, this feature has been exploited to implement heat-induced recycling in DNA biocomputing systems[29,30]"

We have also added a more detailed discussion of the underlying thermodynamics to the Supplementary Materials:

*"**Supplementary Note 3. Calculation of the hybridization temperature dependence using NUPACK**.*

The principles underlying NUPACK are described in detail in the relevant publications[3]. Briefly, NUPACK considers the Boltzmann ensemble of all possible states of participating

oligonucleotides. For each possible state, NUPACK calculates the Gibbs free energy, using the nearest-neighbor model with various corrections, such as loops, stacking interactions, dangling ends, and others. In this model, enthalpy and entropy of each individual state are assumed to be temperature-independent. The resulting information is then aggregated over all states by computing the partition function for each complex.

Let us consider a simple system consisting of two strands, s_1 and s_2. We restrict the analysis to the complexes (s_1), (s_2), and ($s_1 + s_2$), as the concentrations of all other complexes are negligible by design.

If:

3. *Single-stranded species exist mostly as free strands (i.e., structures with non-zero free energy have negligible low concentrations);*

4. *The most favorable duplex state is significantly lower in free energy than all alternative states;*

then the hybridization reaction can be described by only two parameters, (for example, enthalpy and entropy) and dependence of reaction Gibbs free energy on temperature will be linear.

To test this assumption for the oligonucleotides used in this study, we analyzed the same dataset as in Fig. 1A. To exclude extreme cases, we considered oligonucleotide pairs with Gibbs free energy at 25 °C ranging from −30 to −80 kJ/mol. For these 4,684 oligonucleotides, we computed the dependence of ΔG(T) over the temperature range from 25 to 50 °C.

We then estimated apparent enthalpy and entropy from 25 and 50 °C temperature endpoints using simple linear approximation and evaluated the maximum error across all intermediate temperatures arising from predicting ΔG in this manner. The median error was 0.31 kJ/mol, while the maximum error reached 2.6 kJ/mol (0.7% and 10% for the relative error). This supports the validity of the approximate description of the hybridization reaction using only two parameters. In addition, it should be noted that this approximation serves only as an intermediate step to simplify the identification of nucleic acid sequences and, therefore, does not require particularly high accuracy."

2.2 A related point concerns the claimed generality of the results. The authors argue (reasonably, in my view) that DNA serves mainly as a convenient test case for a broader compensation principle. However, the manuscript would be strengthened by citing representative literature directly showing that protein–protein interactions can exhibit similarly variable enthalpic and entropic terms, making analogous compensation plausible in protein-based networks.

Thank you for this important comment. We have analyzed available protein–protein interaction datasets and demonstrate that these interactions exhibit the same properties: the dissociation constant (K_d) at one temperature does not fully determine K_d at another temperature, reflecting an incomplete correlation between enthalpic and entropic contributions. This analysis is presented in Supplementary Note 1 and is referenced in the Concept section of the Results."

"The different behavior of K_d at two temperatures arises from the relative independence of enthalpy and entropy …

Importantly, this property is not unique to nucleic acids, and can also be observed, for example, in protein interactions (Supplementary Note 1)"

"Supplementary Note 1. Analysis of the feasibility of temperature compensation in the context of protein–protein interactions.

As previously noted, the existence of temperature compensation in networks of interacting oligonucleotides is enabled by the existence of "hard-to-melt" and "easy-to-melt" pairs. Importantly, in this context, nucleic acids serve merely as a convenient model system that allows straightforward evaluation of their thermodynamic properties. More generally, any biomolecules that exhibit distinct temperature-dependent behaviors are likewise capable of demonstrating analogous compensatory effects.

To demonstrate this, we consider the case of protein–protein interactions. For the analysis, the Stites (1997) dataset[1] *was selected. Because, for such interactions, the enthalpy typically exhibits a strong temperature dependence*[1] *(i.e., the change in heat capacity ΔC_p is significantly different from zero), only data for which ΔC_p was directly measured were included in the analysis. Otherwise, the use of enthalpy and entropy values obtained at a single temperature (e.g., from isothermal titration calorimetry performed at a single temperature point as is the case in most other datasets) may be misleading when analyzing the temperature dependence of the interaction.*

The distribution of dissociation constants at 25 and 37 °C for protein–protein interactions is shown in Supplementary Fig. 1A. Notably, the same characteristic feature observed for the analogous distribution of DNA–DNA interactions (Fig. 1A) is evident here: for a given K_d at 25 °C, there exist protein–protein pairs with substantially different K_d values at 37 °C.

Moreover, a similar pattern can be observed even in the case where one of the proteins remains unchanged while single-point mutations are introduced into the other. For instance, an analogous distribution is shown in Supplementary Fig. 1B for a mutational scan[2] *of an anti-HER2 antibody reported in Kelley (1993). Notably, the V_H-F100A and V_L-Y92A antibody variants exhibit nearly identical K_d values at 25 °C (1.7 and 2.4 nM, respectively), yet display markedly different K_d values at 37 °C (9769 and 452 nM, respectively), corresponding to a 21.7-fold difference.*

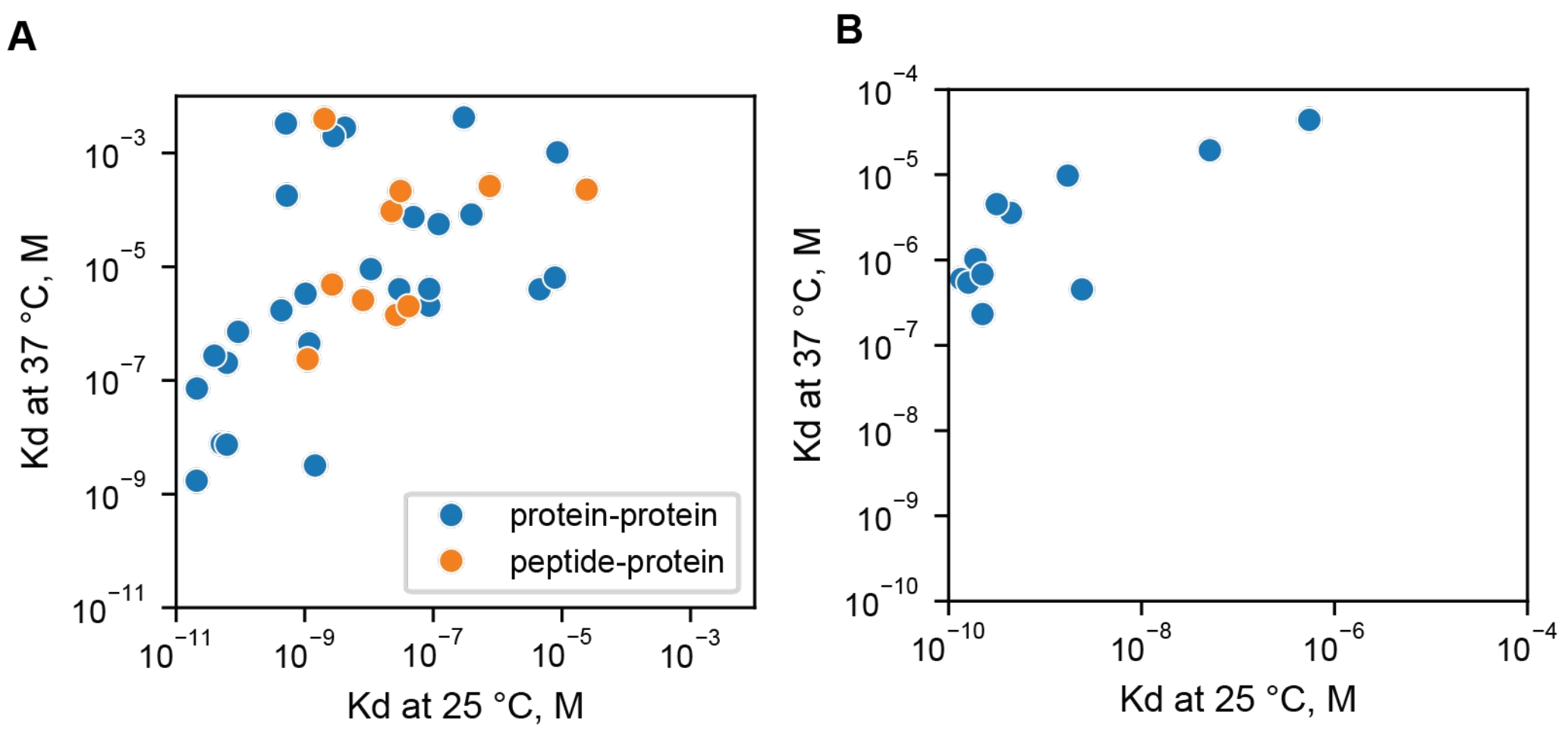

Supplementary Fig. 1. Kd distributions at 25 and 37 °C for protein-protein interactions for (A) various protein-protein and protein-peptide pairs from Stites 1997[1]*; for (B) single-point mutational scan of an anti-HER2 antibody from Kelley 1993*[2]*.*

…

1. Stites, W. E. Protein−Protein Interactions: Interface Structure, Binding Thermodynamics, and Mutational Analysis. Chem. Rev. 97, 1233–1250 (1997).

2. Kelley, R. F. & O'Connell, M. P. Thermodynamic analysis of an antibody functional epitope. Biochemistry 32, 6828–6835 (1993)."

A broader comparison of oligonucleotide and protein law-of-mass-action networks has been included in the Discussion:

*"**Topological compensation in protein-protein networks***

In our study, we demonstrate the realization of a compensatory mechanism using nucleic acids as an example. We believe that the demonstrated here results may be extrapolated to other affinity-based molecular networks, including, of course, small molecule and protein networks[41,42].

With regard to the fundamental feasibility of compensation in such networks, the only property of oligonucleotide networks that we explicitly relied upon is that the behavior of the dimerization reaction at low temperatures does not fully determine its behavior at higher temperatures. Evidently, even with the increased complexity of processes encountered in protein systems, this key assumption remains valid (see Supplementary Note 1). Furthermore, we do not see any conceptual limitations for this idea to be implemented within the mixed networks of nucleic acids, proteins, small molecules, etc.

Moreover, protein networks possess the potential for even more complex behavior. In contrast to oligonucleotides, proteins undergo temperature-dependent folding and denaturation processes, exhibit intricate cooperative effects, and their free energy typically demonstrates a strongly nonlinear dependence on temperature. We believe, that the presence of more complex processes does not complicate the problem of compensation; rather, it provides nature with a broader repertoire of mechanisms for its implementation.

It is also interesting to consider a special case of protein interactions, namely, enzymatic networks, which are regarded as the primary source of temperature-independence in some naturally occurring systems (such as circadian rhythms[18]). We, however, believe that affinity-based networks have many important advantages over enzymatic networks in terms of their compensatory capabilities. For an enzymatic network to be temperature-independent, not only must activation energies be tuned in a precise manner, but also other more critical parameters, such as substrate selectivity and overall catalytic efficiency, so that each enzyme remains functional. All of these parameters are governed by substrate–enzyme interactions and are typically controlled by only a few amino acid residues. Therefore, fulfilling the compensation requirement (i.e., matching activation energies) is inherently difficult. In contrast, in a general case of an affinity-based network, where binding thermodynamics is largely decoupled from such complex functional constraints, the compensatory behavior is considerably easier to achieve (whether through natural evolution or artificial engineering), as much fewer parameters must be optimized simultaneously. i.e., only the binding affinities themselves and the concentrations."

2.3 The authors might also note that an alternative route to temperature robustness in these specific gate motifs would be simply to strengthen binding so the relevant complexes are nearly fully formed at both 25 °C and 50 °C. Of course, this would sacrifice the benefits of weakly complementary designs (and likely would not extend cleanly to the richer "algebraic"

systems), but stating this explicitly would help readers understand what is special about the presented approach.

The discussion of this alternative approach to achieving temperature stability has been inserted into the chapter «Experimental realization of temperature-independent gates»:

"An alternative to the compensation mechanism described above is simply to increase the affinities of all interactions so that on/off behavior is preserved even at high temperatures. However, this approach has two major drawbacks. First, it generalizes poorly to behaviors beyond simple on/off responses, making it difficult to achieve intermediate states. This thereby eliminates the advantages of the molecular commutation regime over strand displacement technique (for example, elementary algebra or ultrahigh input gates has been shown to-date only with molecular commutation, and it is unclear whether a reasonable strand displacement solution can be engineered for such tasks).

Second, it introduces substantial kinetic limitations. In general, higher affinities lead to a significant decrease in the dissociation rates of complexes, which slows down the overall performance of the system. In the extreme case of long, fully complementary strands, the interaction becomes effectively irreversible, and complexes remain stable almost indefinitely at room temperature conditions[37], which in practice corresponds to an effectively infinite operating time for the system."

2.4 One presentation issue may greatly confuse readers: the manuscript frames the initial demonstrations as building a "YES," "NOT," and "AND" gate, which suggests that the compensated designs should perform as good logic gates across temperature. In the current presentation, the uncompensated gates tend to separate logic states better at 25 °C than at 50 °C, whereas compensation largely makes the 25 °C behavior resemble the poorer separation seen at 50 °C (i.e., the low-temperature performance is "degraded" to match the high-temperature behavior). I understand that the primary goal is to illustrate temperature dependence and compensation rather than to optimize digital logic robustness as such, but as written, the fact that "temperature compensation" can make the system behave less like a crisp logic gate at low temperature may put off some readers. At minimum, it would help to acknowledge this tradeoff explicitly and clarify that invariance is achieved relative to a chosen operating point, not necessarily maximal gate fidelity across conditions.

We fully agree with this comment and have added the following paragraphs to the Results:

"

The performance of a logic gate is typically perceived as separation between the output signals in the presence and absence of inputs; the larger this difference at both temperatures, the better the gate operates. However, logic gates are not our ultimate objective but rather serve as an illustrative example of a signal-processing system, we formulate a more general goal, namely, to achieve preservation of absolute concentration levels at all possible input states. Achieving this, however, requires careful optimization

...

We should also note that, for example, for the compensated YES gate (Fig. 2A) the True-False signal difference is not greater than that in the non-stabilized case at their respective optimal temperatures. This reflects the classic "robustness over peak performance" trade-off, a well-known concept in biotechnology (e.g., enzyme engineering[35,36]) where expanding the operational range and ensuring stability is typically more advantageous than maximizing performance within a narrow parameter window. On the other hand, this effect arises from the gate

construction strategy: the control and compensated gates were designed to operate identically at 50 °C (as compensators are "easy-to-melt"). However, if we consider the gates that have identical outputs at 25 °C, then the True-False difference at 50 °C becomes significantly greater for the compensated gate. Moreover, if the objective function is this True-False separation (instead of the preservation of absolute concentrations), the gate design problem becomes substantially simpler. In this case, virtually any YES gate with a topology that has a compensatory component binding Q (at a concentration comparable to that of the other components) will outperform a YES gate lacking such a compensator, provided that both exhibit identical performance at 25 °C (i.e., the same level of free S). This behavior is illustrated for both YES and NOT gates in Supplementary Note 2 and Supplementary Fig. 3

...

34. Nezhad, N. G. et al. Recent advances in simultaneous thermostability-activity improvement of industrial enzymes through structure modification. Int. J. Biol. Macromol. ***232****, 123440 (2023).*

35.Vanella, R. et al. Understanding activity-stability tradeoffs in biocatalysts by enzyme proximity sequencing. Nat. Commun. ***15****, 1807 (2024)."*

To substantiate this claim, we have performed additional simulations, as described in Supplementary Note 2 and Supplementary Fig. 3.

"***Supplementary Note 2. Construction of temperature-compensated systems without parameter optimization across the entire temperature range***

In this section, we demonstrate that the topology alone of signal-processing systems with compensatory species is sufficient to achieve partial temperature compensation.

*Let us consider the topology of a YES gate in its two variants: the first one without compensation (three oligos I, Q, S), and the second with compensation (four oligos I, Q, S, C). For the second one to be conceptually different from the first one, the concentration of C and the affinity of Q–C should not be zero or negligibly small, so let's fix these values to some reasonable values, for example, concentration of the compensator C to 5 µM and the dissociation constant of Q–C at 25°C to 1.7 nM (−50 kJ/mol). Now, consider random gates of these two topologies that have the same level of free S at 25 °C, for example, 100 and 200 nM in the absence and in the presence of input, respectively. Gate performance is typically defined as the difference between outputs in the True and False states (i.e., with and without input), which in this case is 100 nM (****Supplementary Fig. 3A, D****). How would the True-False difference behave at 50 °C for non-compensated and compensated groups of gates?*

We have generated 100 distinct parameter sets for both groups of gates. ***Supplementary Fig. 3B,C*** *presents the distributions of the True-False differences for these groups of gates at 25 and 50 °C. It is evident that the True-False difference at 50 °C is substantially higher in the compensated system, whereas for the majority of control systems it is close to zero; that is, the states with and without input at 50 °C are nearly indistinguishable. Similarly, we performed the same analysis for the NOT gate. The results are presented in* ***Supplementary Fig. 3E,F****, where the same pattern is observed. Thus, an appropriate system topology can ensure temperature stabilization even if its parameters are not optimized for operation at temperatures other than the reference one.*

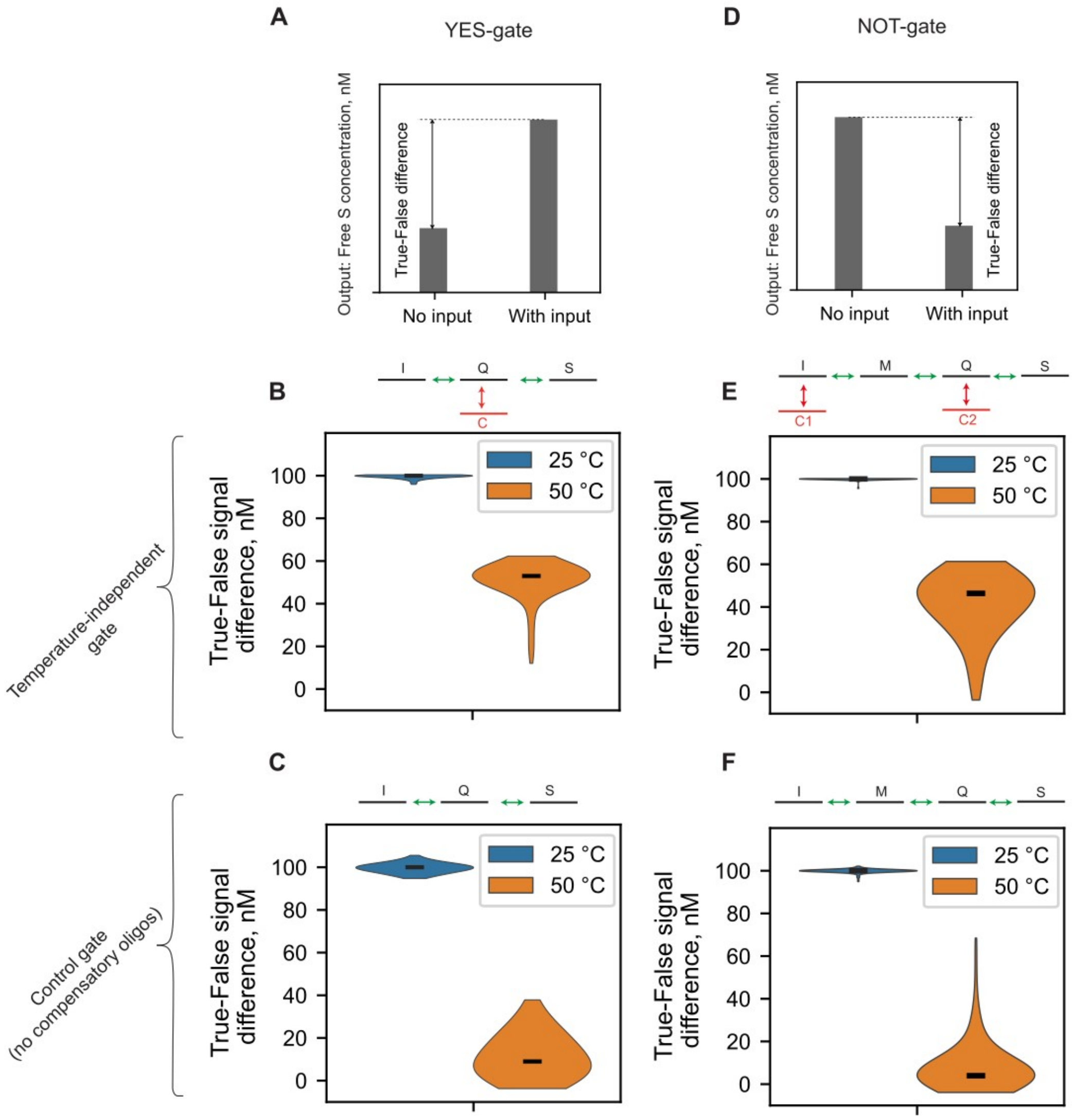

Supplementary Fig. 3. *Sufficiency of compensatory topology alone for stabilizing logic gates under temperature variation. (A, D) Visualization of True-False signal difference for YES and NOT gates. Distribution of this True-False signal difference (i.e., with inputs vs. without inputs) at 25 and 50 °C is shown for 100 (B) YES gates and (E) NOT gates. Panels (C, F) show the same distribution for the control YES and NOT gates (i.e., without compensatory oligos). Black lines show the median values. See Supplementary Note 2 for a detailed description."*

2.5.An important issue is about the use of NUPACK. Throughout the paper NUPACK was used with maximum complex size parameter set to 2. However, in typical systems especially at lower temperatures there can be substantial trimolecular and higher-order binding. I tried simulating the system in Fig. 4B with max_complex_size = 3 and obtained substantially different results showing much less compensation at lower temperatures (I am attaching the comparison plots to this review). I think this might also be a reason that NUPACK predictions were not matching experimental data. Although the expected binding is dimerization, the authors need to be explicitly aware and acknowledge unexpected/undesired higher order binding. Ideally, the systems are reworked to make them robust to a higher max_complex_size parameter.

Thank you very much for noticing this important detail and for your time and effort in performing the simulation. Indeed, predicted performance of many systems changed when the formation of triple complexes was taken into account.

However, all presented systems can be further optimized to account for multimeric complexes, as demonstrated by simulations in Supplementary Figures 4-7, 10. New Fig. 7 was also simulated with max_complex_size = 3. The following paragraph has been inserted into Results section:

"Another simplification was the consideration of only complexes consisting of at most two strands. At the same time, real biomolecules (especially nucleic acids) are typically capable of forming higher-order complexes. However, accounting for such complexes does not introduce any fundamental changes and does not compromise the compensatory capabilities of the mechanism: Supplementary Figures 4–7 demonstrate equally robust performance of the YES, NOT, AND and 0.1-50 °C YES gates respectively when triple complexes are taken into account (NUPACK max_compl_size = 3), designed through simple fine-tuning of previously optimized sequences and concentrations."

However, with regard to the experimental data, at the actual concentrations (50 nM, 200 nM, and 800 nM for the S, Q, and I oligonucleotides, respectively), the maximum concentration of triple complexes predicted by NUPACK was only 0.046 nM. Thus, nearly the same discrepancy with the experimental data is observed even when the max_compl_size parameter is set to 3 (instead of 2).

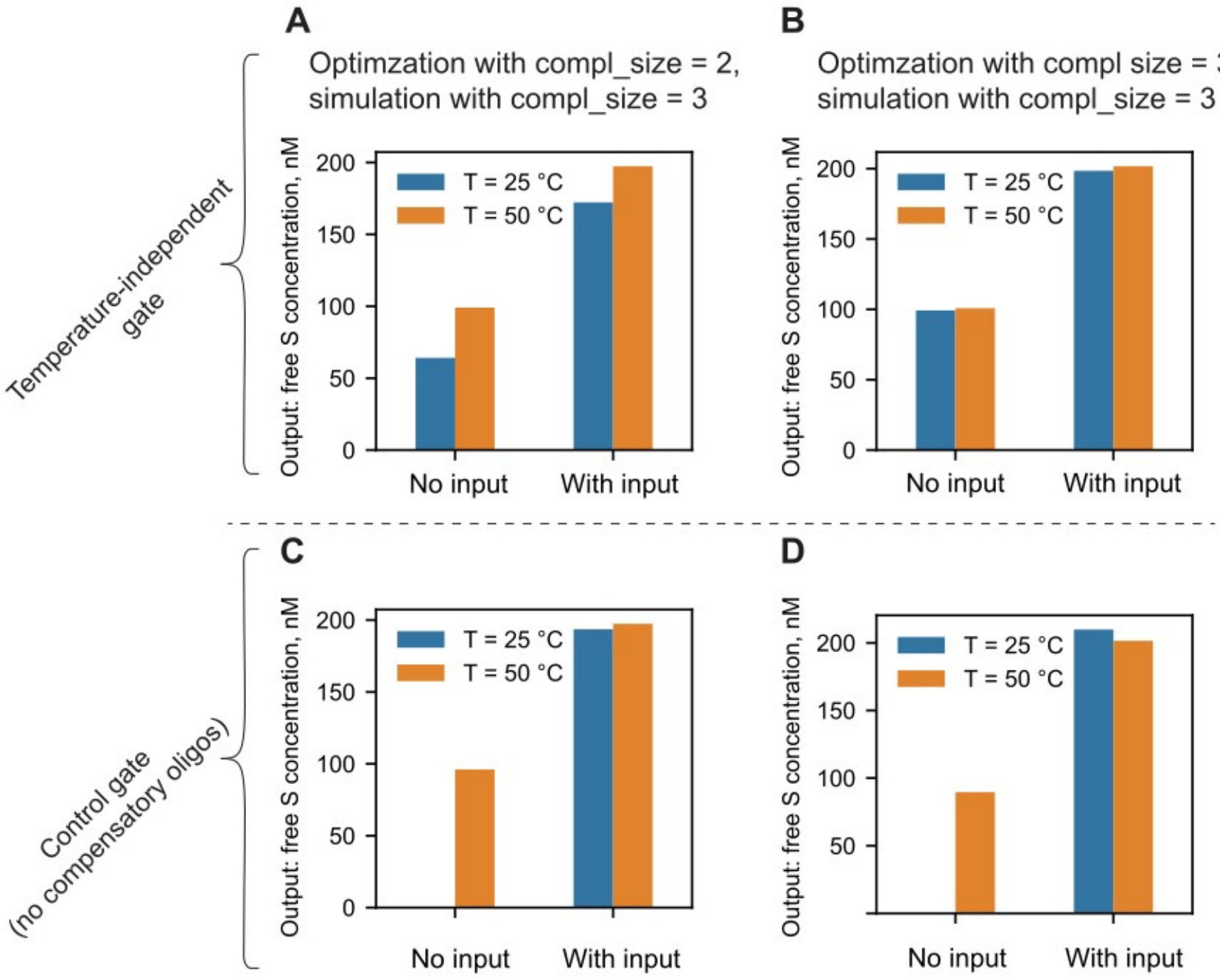

***Supplementary Fig. 4**. Performance of a temperature-independent and control YES logic gates, simulated in NUPACK while accounting for complexes with up to three strands (max_compl_size = 3). For (A), the exact gate parameters (sequences and concentrations) from Fig. 2A were used, where only complexes with up to two strands were considered during optimization. Gate in (B) represents the same system from (A), fine-tuned to perform while accounting for complexes with up to three strands. (C) and (D) show simulations of control systems, identical to those in (A) and (B), respectively, but without compensatory species.*

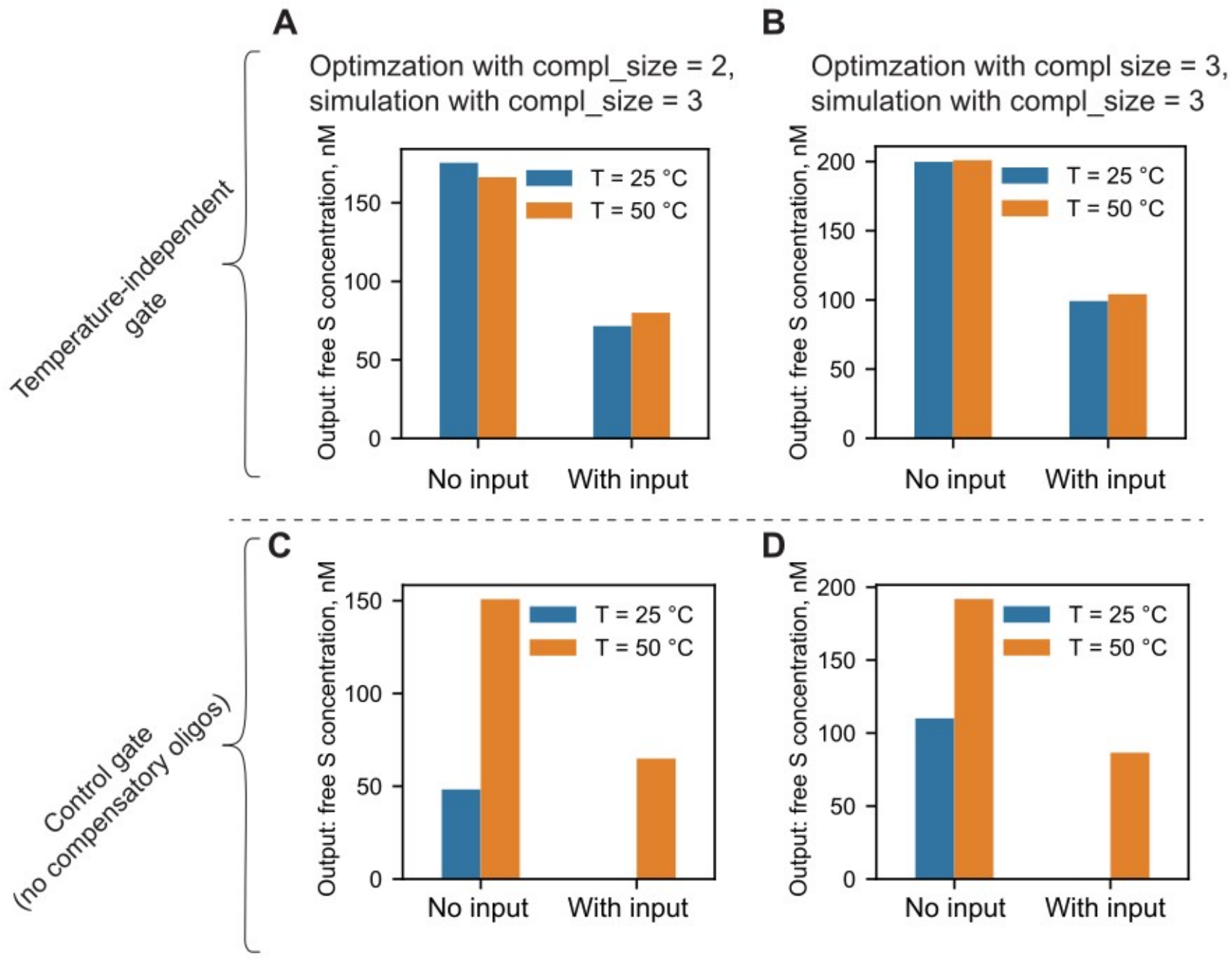

Supplementary Fig. 5*. Performance of a temperature-independent and control NOT logic gates, simulated in NUPACK while accounting for complexes with up to three strands (max_compl_size = 3). For (A), the exact gate parameters (sequences and concentrations) from Fig. 2B were used, where only complexes with up to two strands were considered during optimization. Gate in (B) represents the same system from (A), fine-tuned to perform while accounting for complexes with up to three strands. (C) and (D) show simulations of control systems, identical to those in (A) and (B), respectively, but without compensatory species.*

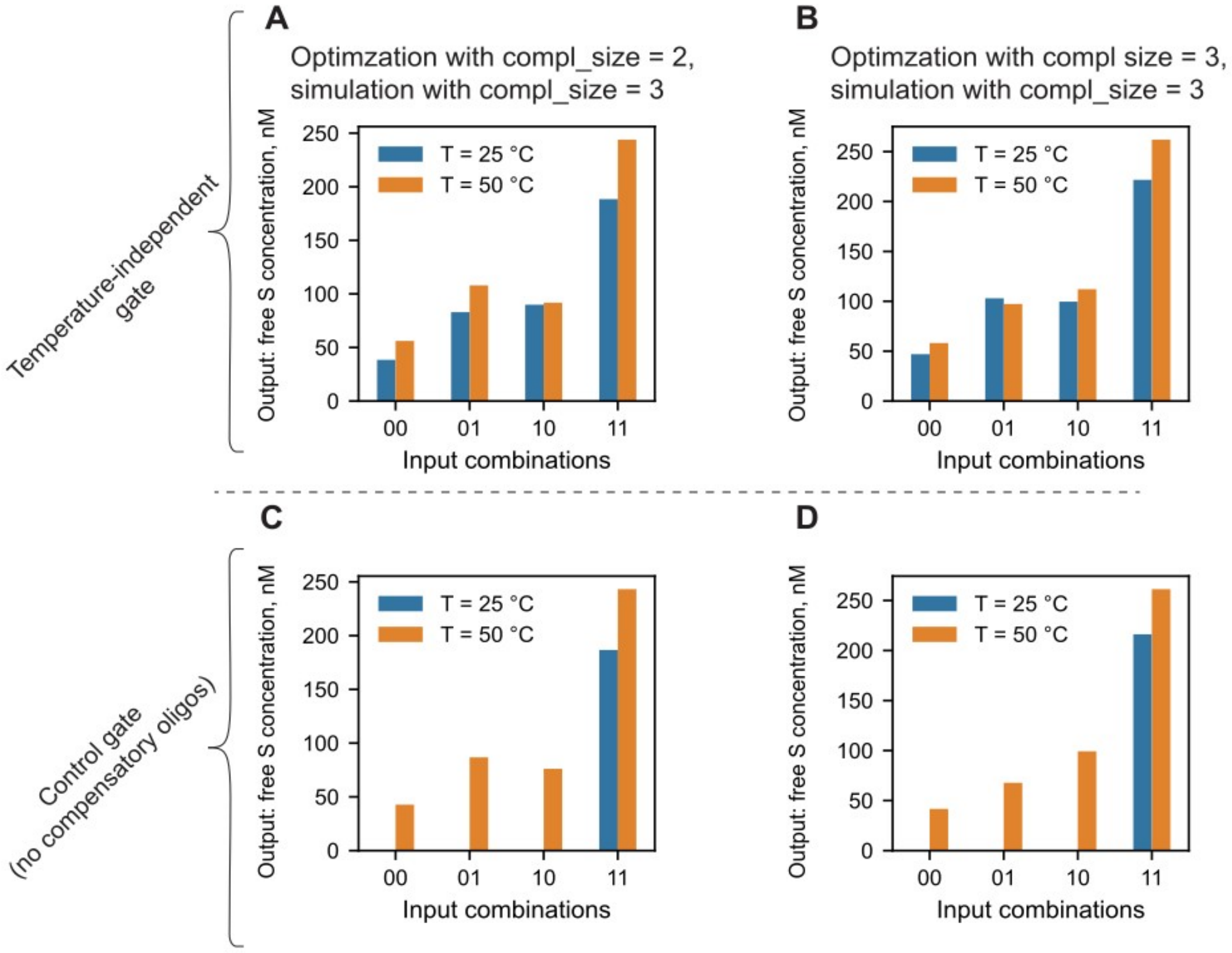

Supplementary Fig. 6. *Performance of a temperature-independent and control AND logic gates, simulated in NUPACK while accounting for complexes with up to three strands (max_compl_size = 3). For (A), the exact gate parameters (sequences and concentrations) from Fig. 2C were used, where only complexes with up to two strands were considered during optimization. Gate in (B) represents the same system from (A), fine-tuned to perform while accounting for complexes with up to three strands. (C) and (D) show simulations of control systems, identical to those in (A) and (B), respectively, but without compensatory species.*

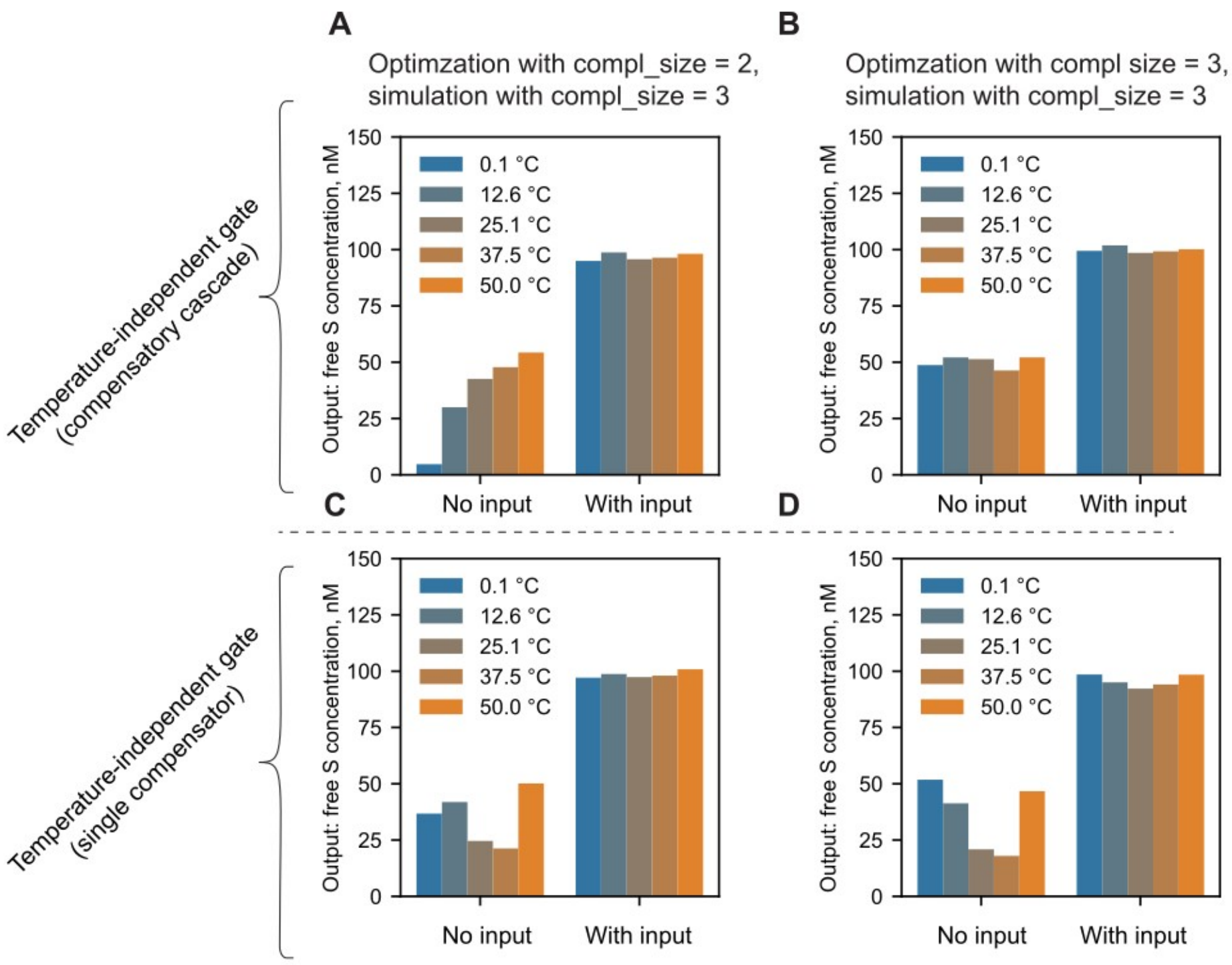

Supplementary Fig. *7. Performance comparison of a temeperature-independent YES logic gate with compensatory cascade and gate with a single compensator (0.1-50 °C diapason), simulated in NUPACK while accounting for complexes with up to three strands (max_compl_size = 3). For (A, C), the exact gate parameters (sequences and concentrations) from Fig. 2E and D were used, where only complexes with up to two strands were considered during optimization. Gates in (B, D) represents the same systems from (A, C) respectively, fine-tuned to perform while accounting for complexes with up to three strands. (C) and (D) show simulations of control systems, identical to those in (A) and (B), respectively, but without compensatory species.*

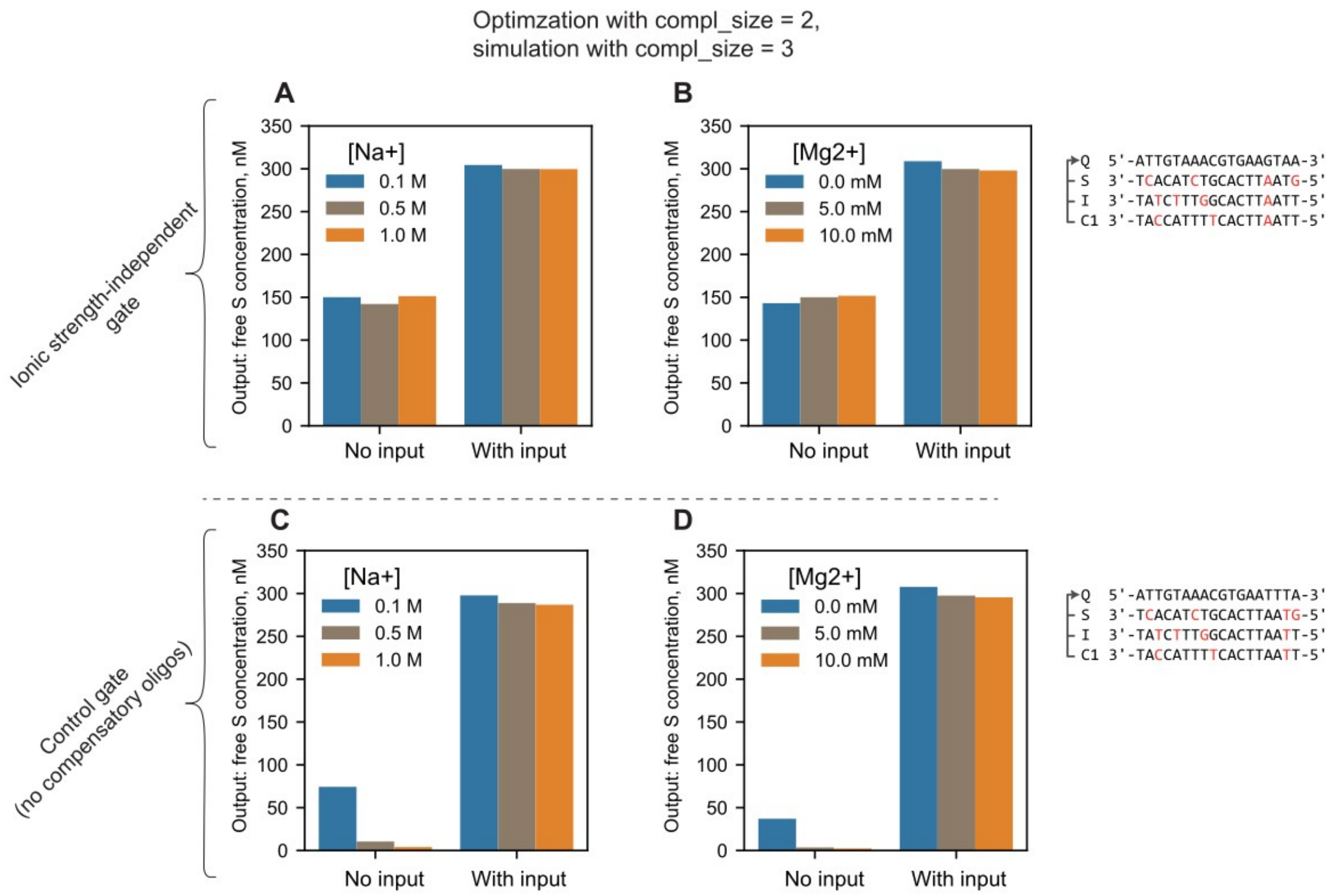

Supplementary Fig. 10. *Performance of salt-independent and control YES logic gates, simulated in NUPACK while accounting for complexes with up to three strands (max_compl_size = 3). (A, C) show gate performance under Na^+ variation (Mg^{2+} concentration is zero), (B, D) show gate performance under Mg^{2+} variation (Na^+ concentration is 50 mM).* Please note that some sequences are written from 3′ to 5′ for demonstrating mismatches*"*

The description of the corresponding optimization procedure was added to the Methods section:

"System fine-tuning to account for non-zero concentrations of triple complexes.

In all simulations involving NUPACK (Fig. 2, Fig. 7, Supplementary Figures 4-7, 10), an additional optimization step was performed to ensure that the equilibrium concentrations of the output oligonucleotide attained their target values for all possible input states when using max_compl_size = 3 and higher

The objective function at this stage was analogous to that used in the PyBADS optimization section. During this refinement procedure, evolutionary sequence optimization steps were alternated with Bayesian optimization of concentrations. The concentration-optimization parameters were identical to those used during the initial system design (listed in Supplementary Methods), with the exception of the maximum number of objective-function evaluations, which was limited to 100 (no such restriction was imposed at the initial stage).

For sequence fine-tuning, a population size of 192 was employed. At each evolutionary step, between one and three random mutations were introduced into each sequence with a probability of 50% per oligonucleotide."

2.6 I appreciated that the full source code is provided (including a general README), which was very helpful. However, for the optimization problem (Bayesian adaptive search), the

problem setup should be stated more clearly in the manuscript without requiring a code dive. In particular, it was not fully clear what constraints and criteria (e.g., maximum/minimum allowed concentrations, excluded interaction thresholds, etc.) are enforced. It would help to explicitly state the loss function and summarize the key constraints in the Methods or Supplementary Information.

The main optimization logic was described in the Methods section, the system-specific constraints were added to the Supplementary Information:

Main text (Materials and Methods):

"PyBADS optimization

During PyBADS optimization, only numerical parameters were optimized, namely concentrations, affinities (all figures) and "easy-to-melt" levels (only Fig. 7). PyBADS optimization uses hybrid approach, combining poll and search stage. Briefly, in the poll stage, points are evaluated on a mesh by taking steps in one direction at a time, until an improvement is found or all directions have been tried. In the search stage, a Gaussian process surrogate model of the target function is fit to a local subset of the points evaluated so far. All internal optimization parameters, including the definitions of priors in the second step, were used as described and implemented in the original papers[33,34].

Please note that, in some cases, a slight improvement in the objective can be achieved by rerunning the optimization one or two times, using the optimized values from the previous step as initial points. It is also advantageous to run multiple optimizations in parallel and select the run that yields the best result. Typically, for simpler systems (such as a YES gate), a single run is sufficient, whereas more complex systems (such as algebraic systems) may require 24–48 parallel runs.

In Fig. 7, where "easy-to-melt" levels were also tuned (in addition to dissociation constants and concentrations), they were optimized as a continuous parameter in the range [0, 10] and then converted to the nearest integer to yield the "easy-to-melt" level.

Below, we provide formal definitions of the objective functions optimized in this step. We use the following notation:

5. *T is the set of temperatures of interest, for most cases, $T = \{25, 50\}$°C.*
6. *Λ is the set of all possible logic input states. For one-input gates $\Lambda = \{0, 1\}$, for AND-gate $\Lambda = \{00, 01, 10, 11\}$.*
7. *p is the set of all system parameters, such as concentrations, dissociation constants, sequences etc.*
8. *$S(t, p, i)$ is the concentration of output oligonucleotide in free form at temperature t at input state i.*
9. *S_i^{target} is the desired concentration of the output oligonucleotide in free form at input state i.*

Then, for logic gates, objective function is defined as:

$$L(p) = \sum_{t \in T} \sum_{i \in \Lambda} \left| S(t, p, i) - S_i^{target} \right|.$$

For receptor activator system j is the receptor index, N is the total number of receptors, R_j^{target} *is the desired level of activation of the j-th receptor,* $R_j^{act}(t)$ *is the activation level of the j-th receptor at temperature t. The objective functions defined as:*

$$L(p)=\sum_{t\in T}\sum_{j=1}^{N}\left|R_j^{act}(t,p)-R_j^{target}\right|.$$

For algebraic systems (Fig. 6) x is the normalized and centered input concentration (see main text), X is the set of all x of interest, f(x) is a user defined function. The objective function is defined as:

$$L(p)=\sqrt{\sum_{t\in T}\sum_{x\in X}\left(S(t,p,x)-f(x)\right)^2}.$$

For molecular commutation systems with programmable temperature/ionic-strength dependence (Fig. 7) f(t) is a user defined function. The objective function is defined as:

$$L(p)=\sum_{t\in T}\left|S(t,p)-f(t)\right|.$$

Sequence finding optimization

Sequence selection was performed using an evolutionary algorithm using DEAP python library53. At this stage, the target Gibbs free energies $\Delta G_{ij}^{expected}$ *of hybridization at the specified temperatures (typically 25 and 50 °C) are assumed to be known for all pairs of oligonucleotides in the system.*

The algorithm proceeds iteratively, constructing one oligonucleotide at a time. The first oligonucleotide is initialized randomly.

We now describe the procedure for designing the N-th oligonucleotide, assuming that the preceding N−1 oligonucleotides have already been determined. The initial guess for this oligonucleotide was defined as the reverse complement of the oligonucleotide, that must exhibit the strongest binding affinity to the sequence being designed:

$$seq_N^{start}=rev.compl(seq_m),\ \ m=\underset{j=1\dots N-1}{argmin}(\Delta G_{jN}).$$

The sequences in the initial population were generated from this starting sequence by introducing between one and four random mutations. Subsequently, during the evolutionary process, each oligonucleotide was either modified by introducing between one and six random mutations or reinitialized by applying one to two random mutations to the starting sequence. After evaluating the objective function for all members of the population, the next generation was constructed using random tournament selection with a tournament size of three.

The objective function was defined as the sum of absolute deviations between the Gibbs free energies of hybridization (at all target temperatures) with previously N-1 found oligonucleotides evaluated by NUPACK and the corresponding target Gibbs energy values:

$$L=\sum_{t\in\{25°C,50°C\}}\sum_{i=1\dots N-1}\left|\Delta G_{i\mathrm{N}}^{expected}(t)-\Delta G_{i\mathrm{N}}^{NUPACK}(t)\right|.$$

In cases where no interaction between oligonucleotides i and j was desired (i.e., off-target interaction), the Gibbs free energy of hybridization of the selected sequences was required to remain below a threshold defined as follows. The dissociation constants of off-target interactions had to be at least k_atten-fold weaker than the weakest dissociation constant among all target

interactions (as determined in the previous parameter-optimization step). If an off-target interaction was weaker than this threshold, it contributed zero to the loss function; otherwise, a large penalty was imposed that was guaranteed to exceed all other terms in the loss function.

The parameter k_atten was specified independently for low and high temperatures and denoted as lowtemp_ka_atten and hightemp_ka_atten, respectively. Additionally, if the self-dissociation constant of an oligonucleotide was stronger than 5×10^{-5} M, a penalty term exceeding all other components of the loss function was added. Finally, the loss function included the absolute value of the Gibbs free energy of secondary structure formation for the given oligonucleotide, divided by the parameter hairpin_atten.

Unless otherwise specified, the following optimization parameters were used: sequence length = 20, population size = 280, number of generations = 100, lowtemp_ka_atten = 105, hightemp_ka_atten = 104, hairpin_atten = 5, early stop condition = 1 kJ/mol.

If, upon reaching the maximum number of generations, the error still exceeded the threshold, all N−1 sequences identified up to that point were discarded, and the optimization was restarted from scratch."

Example of table of parameters (for AND gate) in the Supplementary Methods:

"Optimization parameters for determining concentrations and binding affinities.

		Concentration lower bound, nM	Concentration upper bound, nM	Concentration start value, nM	
Nodes	I1	100	5000	1000	
	I2	100	5000	1000	
	Q1	100	5000	1000	
	Q2	100	5000	1000	
	S	100	400	400	
	C1	100	5000	1000	
	C2	100	5000	1000	
	C3	100	5000	1000	
		Gibbs energy at 25 °C lower bound, kJ/mol	Gibbs energy at 25 °C upper bound, kJ/mol	Gibbs energy at 25 °C start value, kJ/mol	Easy-to-melt level
Edges	I1-Q1	-60	-30	-60	p95
	I2-Q2	-60	-30	-60	p95
	Q1-S	-60	-30	-60	p95
	Q2-S	-60	-30	-60	p95
	C1-Q1	-60	-30	-60	p40
	C2-Q2	-60	-30	-60	p40
	C3-S	-60	-30	-60	p40

The target values for the free concentration of the output oligonucleotide were 50 nM (when both inputs were absent), 100 nM (when only one input was present), and 250 nM (when both inputs were present).

Optimization parameters for sequence finding: hightemp_ka_atten = 10^3, lowtemp_ka_atten = 10^4, hairpin_atten = 3, early stop condition = 1 kJ/mol"

2.7 Line 25: Typo: "up to" => "from" or some other wording

Corrected to:

"... we computationally show that "topological compensation" phenomenon can render their signaling function completely independent from temperature and ionic strength (e.g., in [0.1; 50] °C range)"

2.8 Fig. 1: The count of S's should be the same in both "no input" and "with input" cases

Corrected

2.8 Line 113: Incomplete sentence or awkward grammar of sentence starting with "If we consider duplexes that exhibit..."

Changed to:

"On average, this temperature increase leads to a rise in K_d by five orders of magnitude, with a variation of approximately two orders of magnitude. Among duplexes with similar stability at 25 °C, we refer to those with stronger binding at 50 °C as "hard-to-melt," while those with weaker binding at 50 °C are termed "easy-to-melt" (Fig. 1B)."

2.9 Line 158: Clarify which four dissociation constants. What about off-target/undesired dissociation constants?

Indeed, it is typo as there are only three dissociation constants. It has been corrected as follows:

"This leads to a purely numerical continuous optimization problem — in this case, involving the optimization of four initial concentrations (S, Q, C, and I) and three dissociation constants (for Q–S, Q–I, and Q–C interactions) to match the target free S levels at both temperatures. Other interactions are assumed to be absent (corresponding Kd = inf) at this stage and are therefore not optimized."

2.10 Line 192: it should probably be "M binds to I, reducing M's availability to bind Q."

Corrected

2.10 Figure 2 and other figures: The figures and/or figure captions should mention concentrations of inputs (what does 1 input mean), as well as concentrations of other components.

We understand the Reviewer's rationale and we agree with the importance of listing concentration (even better with the affinities) in the figures and/or captions. However, when we attempted to include all of this information, the figures became heavily overloaded and visually difficult to interpret. Figure captions also seemed very bulky. Therefore, we kindly ask the Reviewer not to insist on including these details in the figures/captions. Instead, we included reference to Supplementary Methods before Fig. 2, which lists all the concentrations and other relevant parameters for all systems in the manuscript, after the description of the first setup:

"Here and below, the concentrations, sequences, affinities, and other parameters of the systems presented in all figures are listed in Supplementary Methods. For all systems, each species has a concentration within the range of 1 nM to 10 µM."

2.11 Line 221: Typo: "Importantly, if instead of DNA they networks..."

Corrected. The line has been removed, and the analysis of the applicability of the compensation principle has been expanded and moved to the Discussion section (see reply to comment 2.2).

2.12 Figure 3A: In all the other cases, Q:I is stronger than Q:S, but in set 3, the Kd's are the same. Is this a typo?

Thank you for this observation. We aimed to achieve this ratio, mistakenly assuming that replacing a mismatch with a canonical Watson–Crick pair would always increase affinity. However, this is not always the case, as illustrated by this example. Specifically, we replaced a terminal mismatch with a terminal A–T pair, which, according to the NUPACK model, is destabilizing. Accordingly, we have retained the design description (I-Q has one mismatch fewer than Q-S) but removed the incorrect statement "*As a result, the affinity of I for Q was expected to be stronger than that of S for Q by design*".

2.13 Figure 3C: More details of experimental conditions are needed, specifically how long was the incubation time. Otherwise, it's not clear what the connection is to Figure 3A.

This is indeed an important point. However, because a pre-heating-cooling cycle was used in the experiment to ensure that equilibrium was reached at the starting point of the melting curve, the reported in the Methods section incubation times largely reflect the duration of the heating and cooling steps rather than the timescales of the reactions. To demonstrate the connection with Fig. 3A, we experimentally measured the kinetics of this process and added the actual equilibration times of the system. The legend of Fig. 3C has been revised as follows

"Fig. 3. ... Pre-heating-cooling cycle (25-50-25 °C) was used in the experiment to ensure equilibrium at the starting point of the melting curve. Without the cycle, the gate reaches equilibrium within 30 min at 25 °C, kinetics is shown in Supplementary Fig. 8.

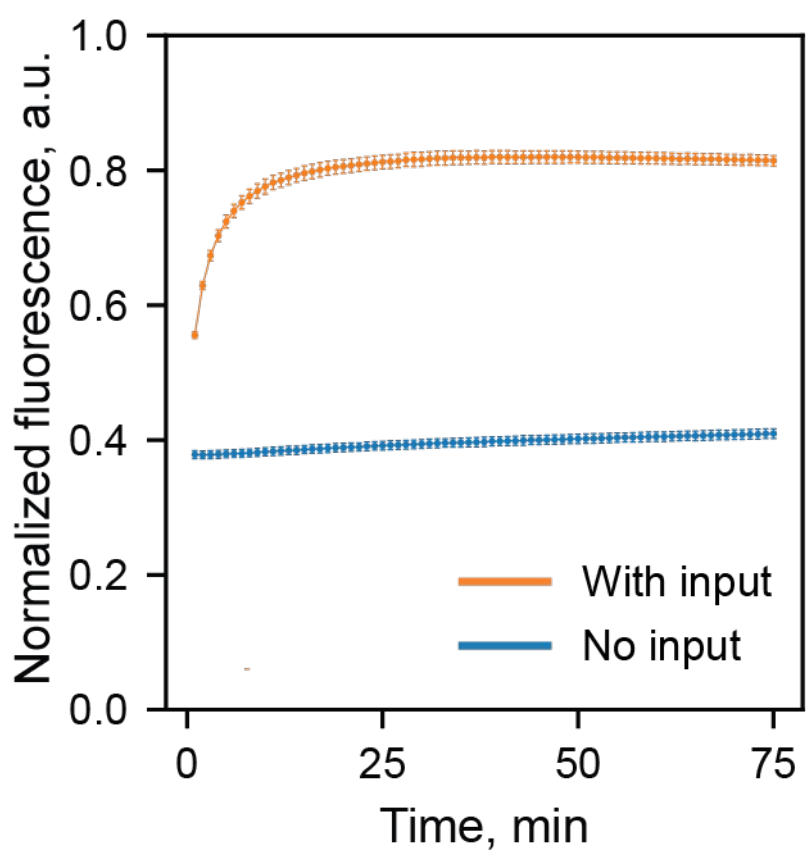

Supplementary Fig. 8. *Kinetics of the temperature-independent YES gate from Fig. 3C following the addition of the input oligonucleotide. Other components (S, Q, and C) were pre-equilibrated for 1 hour. Data are presented in relative fluorescence units as mean ± s.d., n = 3 independent samples. Fluorescence is normalized to the signal of a sample containing only S at the same concentration."*

According to the request by Reviewer #3, we have also added experimental implementation of salt-independent gates, and replaced gates simulation in Fig. 4 with wet lab experiment. Similar kinetic study has also been carried out for consistency with temperature-independent systems.

"Fig. 4. ... In both cases, the system reaches equilibrium in less than 5 min; the kinetics are shown in Supplementary Fig. 12"

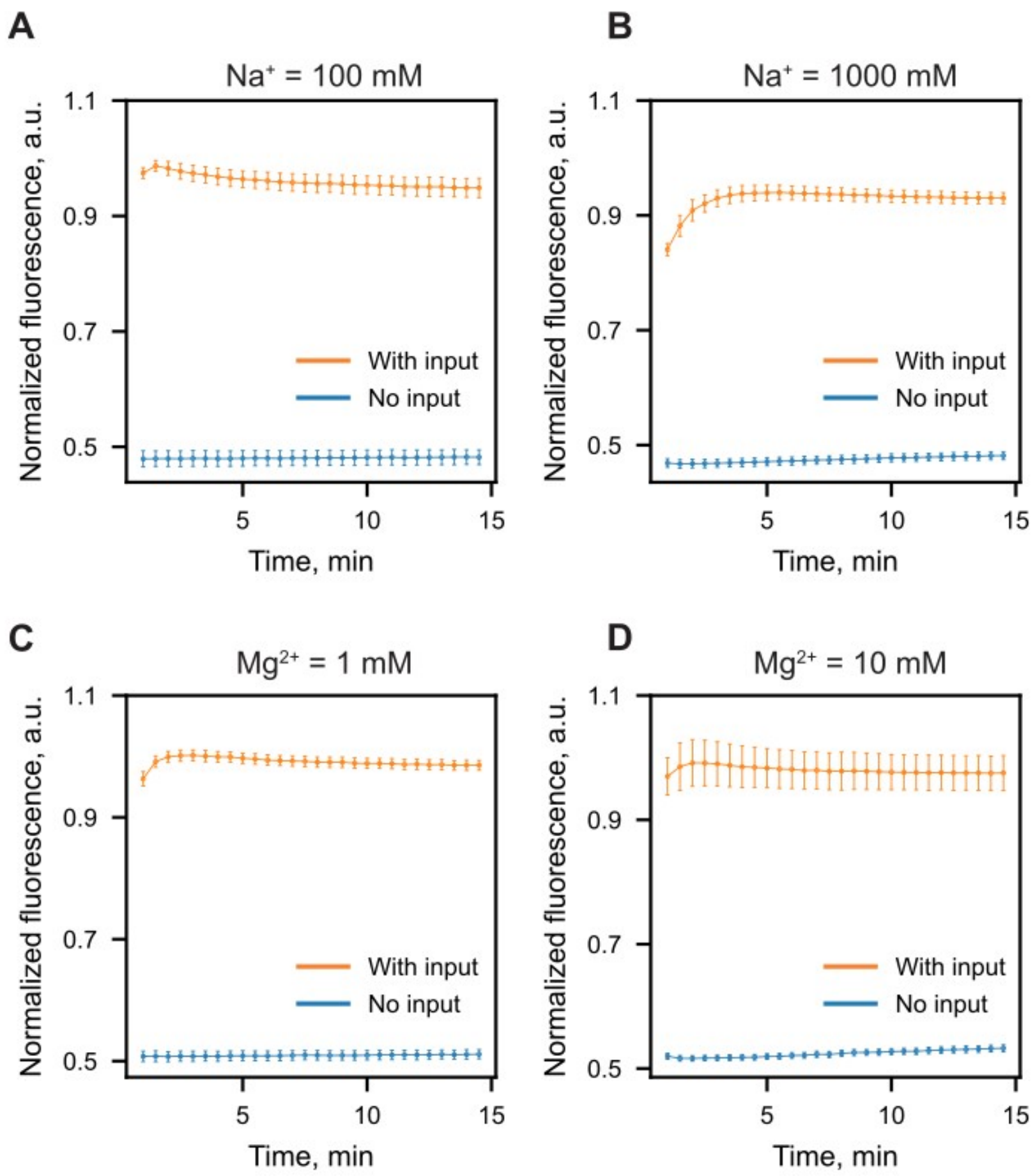

*"**Supplementary Fig. 12**. Kinetics of salt independent YES-gates from Fig. 4 following the addition of the input oligonucleotide. Other components (S, Q, and C) were pre-equilibrated for 1 hour. Data are presented in relative fluorescence units as mean ± s.d., n = 3 independent samples. Fluorescence is normalized to the signal of a sample containing only S at the same concentration. (A, B) show the gate performance optimized for robust operation under sodium variation, while (C, D) show the corresponding results for magnesium variation"*

2.14 Figure 4: Please double-check all the sequences shown in the figures. The supplementary information has different sequences than shown in 4B, for example (SI, page 11, top). Also in 4B, the "M" strands should be "C". I believe the sequences in the SI are the correct ones. (I didn't check the consistency of other sequences.)

Indeed, in Fig. 4B "M" oligonucleotides should be named as "C" – we corrected this typo.

Regarding the difference in the sequences, we confirm the sequences shown in original Fig. 4B correspond to those provided in the original Supplementary Information, but some of them were shown in different orientations (3' –> 5' vs. 5' –> 3'). For example,

in the original Fig. 4B M1 (should be C1) is **3'-TAG**ATGGACGGAGGGAGCGG-5',

in the Supplementary Information C1 is 5'-GGCGAGGGAGGCAGGTA**GAT-3'.**

In the figures, mismatches with other sequences are highlighted to present the distribution of the mismatches over each sequence. Accordingly, that requires presenting some oligonucleotides in reverse orientation (3′ - 5′), which may cause confusion. To clarify this, we have added the following note to all figure captions: *"Please note that some sequences are written from 3′ to 5′ for demonstrating mismatches."*

Furthermore, we double-checked all sequences and haven't found any other issues.

2.15 Figure 5A: Because the top line has three things in order (A, R, Activated receptor), it is easy to visually misinterpret the second line (C1---A1---R), which also has three things, to refer to the molecules shown in the first line in order.

We have reworked Fig. 5A to reduce this ambiguity:

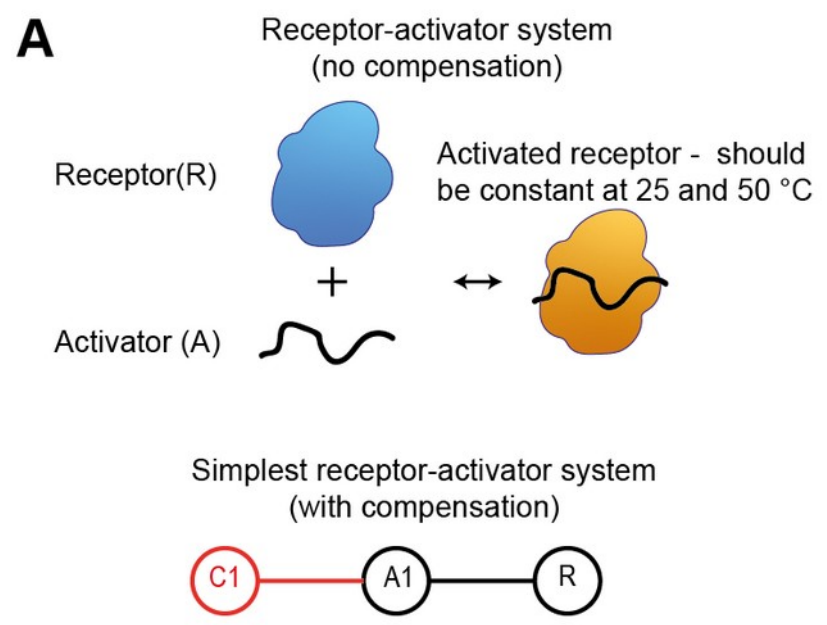

2.16 Line 434: Since the equation on the previous line ends with a comma, this line should not begin with a capital "W".

Corrected

2.17 Figure 6: While the text is clear that X and Y are on vastly different scales (5 uM vs 20 nM), I think the figure should make this more clear in the figure itself or in the caption at least. Otherwise, the units are too abstract.

We have revised the axis units to represent input and output concentrations (relative to its baseline value), replacing the previous notation of X and Y (example shown for cubic function):

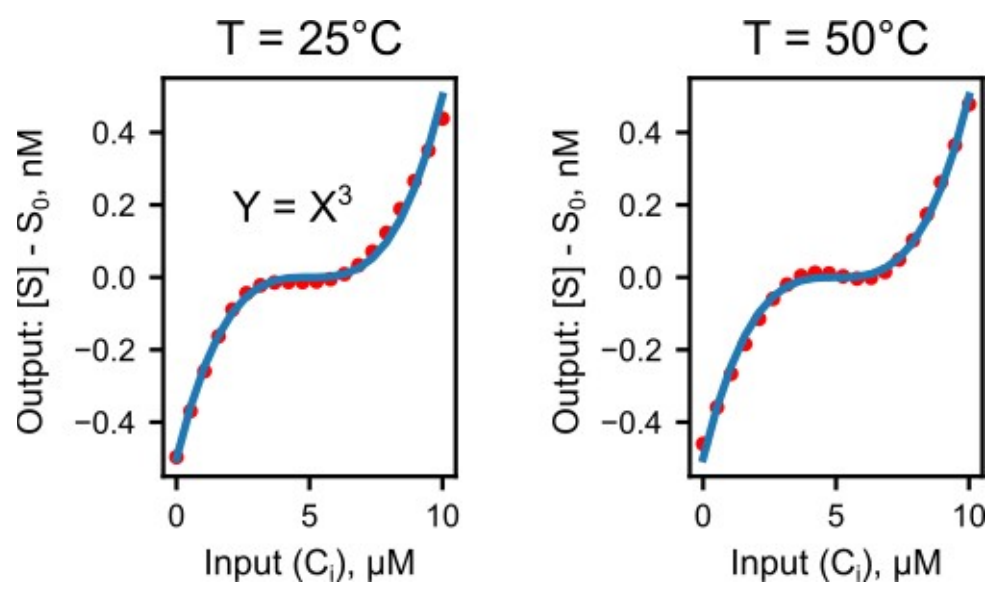

We have also added the following paragraph, discussing the different scales of X and Y:

"It is important to note that, in this section, our objective did not include maximization of the amplitude of the output concentration (Y) changes. Our goal was to demonstrate the general principle of compensation in complex networks. The chosen amplitude values (for X and Y) were therefore selected primarily to simplify the optimization problem. At the same time, achieving larger changes in Y in response to smaller changes in X is certainly possible, but will require longer optimization or use of more advanced optimization approaches[27]. Nevertheless, even relatively small variations in Y can be biologically significant, as downstream systems may strongly amplify such signals — for instance, when the output corresponds to mRNA concentration, from which many copies of a target protein can be synthesized."

2.18 Line 479: Unclear what "contemporary regulatory systems" means.

The phrase has been changed to:

"The described phenomenon of topological compensation in affinity-based molecular networks provides a number of interesting ideas and possibilities for both evolutionary theory and molecular regulatory systems in general."

2.19 Line 480: It would help to tighten this paragraph to a few sentences.

Corrected, revised version:

"Currently, the RNA world hypothesis is recognized as one of the fundamental stages in the emergence of life[42]*. Evidently, life in such a form was insufficiently developed to actively sustain constant physicochemical conditions. In this context, the demonstrated compensation mechanism may have provided an efficient and evolutionary simple tool enabling a broader range of permissible environmental conditions. Consequently, this mechanism may have played a crucial role in the development of all subsequent forms of life. "*

2.20 Line 549: Should this refer to Figure S1 rather than S3?

Corrected

2.21 Line 652: Missing year in ref 18

Corrected

To the Reviewer 3

The work by Korenkov and Nikitin investigates how equilibrium responses of molecular networks can be made insensitive to temperature and salt concentration by adding additional interactions whose interactions contribute at low temperature but not at high temperature, so that changes in binding strengths cancel out at the network level. This compensation mechanism is first discussed theoretically for DNA-oligos and then illustrated experimentally by implementing a temperature compensated DNA-based YES-gate. The proposed salt-compensation is supported by numerical modeling but is not experimentally validated. The remainder of the manuscript is also theoretical, addressing large networks with ligand-receptor binding as well as networks that can perform algebraic calculations. In all cases, Bayesian optimization methods are used to design the networks.

We thank the Reviewer for the careful analysis of our manuscript, for the valuable comments, and the references provided. We are grateful for the constructive suggestions that helped improve the manuscript. We hope that the responses presented below together with the newly added results address all of the Reviewer's questions, including concerns regarding the novelty of our ideas over the prior studies.

3.1. This work builds on an earlier publication by Nikitin (Nature Chemistry 2022, introducing molecular commutation) and extends it into the temperature domain. The phenomenon of temperature compensation has been studied quite extensively in the context of circadian clocks, albeit for oscillating behaviors instead of equilibrium responses. Such responses have been approached in enzymatic networks (e.g., Ruoff, Zakhartsev and Westerhoff, 2006) or more specifically in the chemotactic reaction network of E. coli (Oleksiuk, 2011). While the framework of molecular commutation adds a different angle to it, it does therefore not constitute a major novelty by itself. Compensation with regard to ionic strength seems less systematically explored and is indeed another interesting aspect to robustness of molecular computation. But as the authors write themselves, its effect is similar to temperature, because both weakens interactions.
Another aspect of this work is that the construction principle described by the authors is an example of "fine-tuning", while it is generally accepted that biological systems evolve robust mechanism that would also work under perturbations. The fact that dissociation constants can be found that compensate for temperature effects is interesting, but not particularly surprising. The authors' approach to fine-tune the system is reminiscent of early circadian clock models going back to Leloup & Goldbeter, 1997 and Hong & Tyson, 1997. However, the field has moved to more robust temperature compensatory designs deeming a fine-tuned temperature compensation as too unrealistic for biological systems (compare, e.g., Kidd, Young & Siggia, 2015 among others).

We thank you for pointing out these important works, we have cited them in the Introduction:

"Interestingly, enzymatic networks may be involved in temperature independence in naturally occurring systems under certain conditions[15], namely when the weighted sum of the activation energies of specific reactions in the network equals zero. This mechanism is considered to contribute to the thermal stability of particular biochemical cascades, such as circadian rhythms[16–18] or bacterial chemotaxis[19]. However, evolution-driven or artificial design of the enzymatic networks that adhere to such a narrow constraint seems to be highly complex as these systems must additionally develop and retain other critical parameters for each molecule of the network, including substrate affinity, substrate selectivity, catalytic function, reaction rates etc."

Below, we provide a point-by-point response regarding why we believe that even our original manuscript (before this revision) had significant novelty relative to the cited studies. However, following your comment and being inspired by all the Reviewers, we performed an additional study with a major new result (Fig. 7), which significantly broadens the capabilities of our concept.

In particular, we demonstrate that affinity-based networks can be engineered to exhibit virtually any desired temperature/ionic-strength dependence, not just provide temperature independence. To the best of our knowledge, this finding is entirely novel and has not been reported in the cited works or elsewhere. We kindly ask that you first consider these new results before proceeding to the point-by-point response.

*"**Programmable temperature/ionic strength dependence***

After thoroughly discussing temperature independence (i.e., a dependence reduced to a constant), we proceed to the more general task of designing systems with arbitrary dependence on temperature or ionic strength. Such behavior could be beneficial, for example, for inducing signals of arbitrary forms in different cell types as body temperature rises during disease.

Another illustrative example of where such systems may be important is in ensuring temperature independence of a higher-level molecular system with its own complex temperature dependence. For example, consider temperature-independent bacterial chemotaxis. In this well-studied system[19] the key determinants of this independence include (1) the enzymatic network and (2) the mRNAs encoding these enzymes together with the translation-related machinery. The latter ensures a selective and tightly controlled increase in the expression of specific components of the enzymatic network as temperature rises.

*In turn, as demonstrated in our prior work[21], there exist mechanisms by which molecular commutation can significantly influence protein translation levels. Specifically, binding of DNA oligonucleotides to a specific site on an mRNA molecule may lead to its cleavage mediated by RNAse H, thereby reducing the expression of the corresponding protein (**Fig. 7A**). Consequently, free mRNA levels (i.e., mRNA not bound to oligonucleotides), representing the output of the nucleic acid affinity-based network, may directly determine protein concentrations.*

*Thus, suppose that a certain complex biochemical system requires, for its temperature independence or other higher-level signaling, a specific temperature-dependent profile of the level of free mRNA (**Fig. 7B**). In this section we show that it is possible to identify sets of DNA oligonucleotides that realize predefined complex, non-monotonic dependencies on temperature or ionic strength.*

The formal statement of the problem is analogous to that of the previous section: the variable Y (the level of free mRNA) is defined in exactly the same way, whereas the variable X is defined as the normalized and centered temperature:

$$X = \frac{T - T_{min}}{T_{max} - T_{min}}.$$

The identification of DNA sets having a predefined temperature dependence Y(X) was carried out in a manner similar to the design of logic gates. We first fixed the sequence of the "output oligonucleotide" as the AUG-containing region of the firefly luciferase mRNA that was used in our prior study[21] for the experimental demonstration molecular commutation effect on protein expression.

For each specified function Y(X), the required dissociation constants, "easy-to-melt" levels, and concentrations of the components were first determined using Bayesian optimization. After that, DNA sequences realizing these parameters were identified using an evolutionary algorithm. Finally, an evolutionary fine-tuning of both sequences and concentrations was performed to ensure that the system exhibited the desired behavior while accounting for nonzero concentrations of triple complexes (NUPACK max_compl_size = 3).

This procedure was carried out for four sample dependencies Y(X): linear, quadratic, cubic, and sinusoidal. The resulting DNA sets, their interaction graphs, and their temperature dependencies are presented in ***Fig. 7C–F****.*

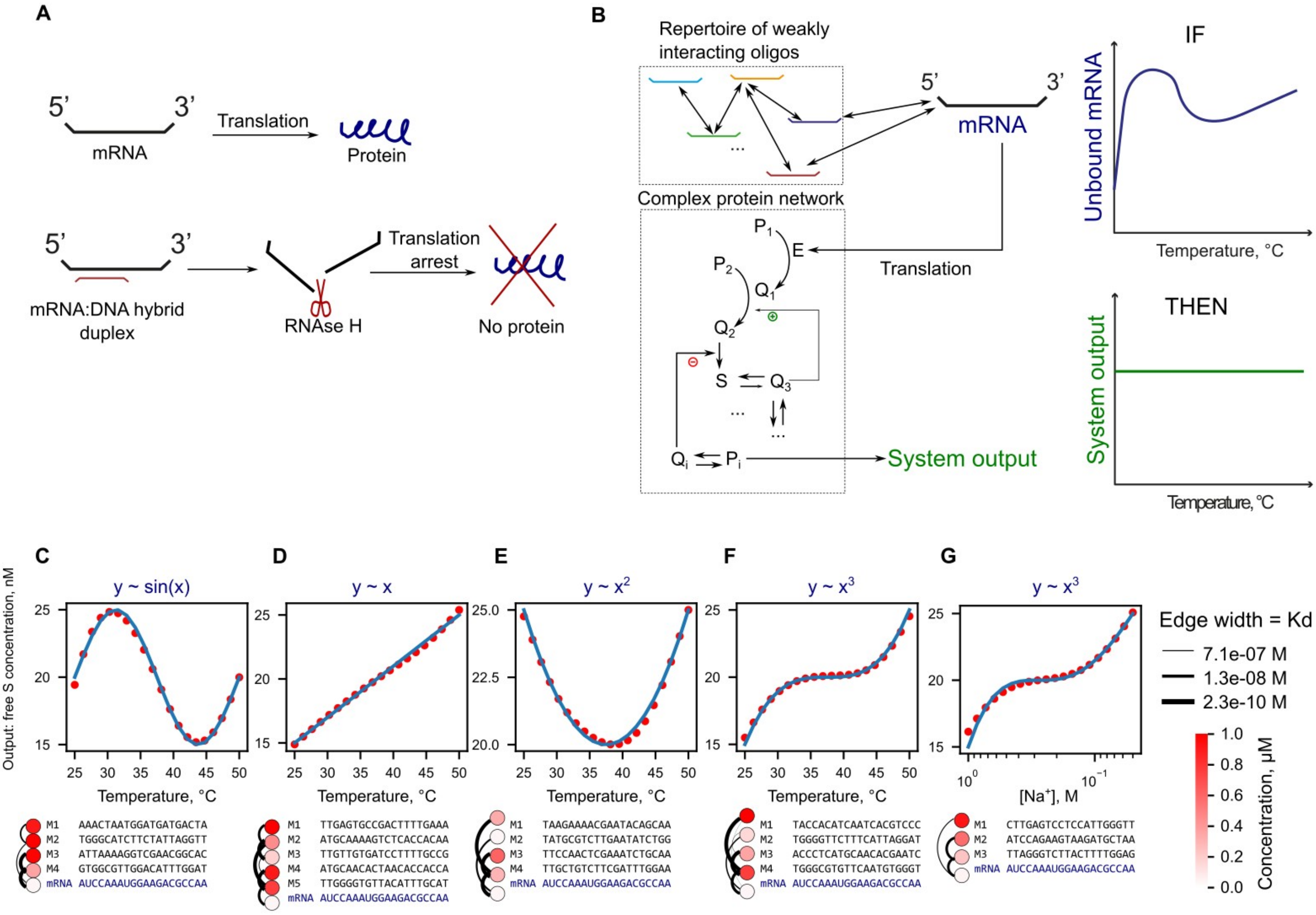

Fig. *7. Molecular commutation systems with programmable temperature/ionic strength dependence. (A) Schematic of translation control via an RNAse H-mediated mechanism. (B) Scheme of a nucleic acid molecular commutation system that compensates for the complex behavior of a protein network. Temperature compensation of the protein network requires a predefined level of protein expression, which is regulated by mRNA levels. (C–F) Performance and interaction graphs of molecular commutation systems exhibiting linear, quadratic, cubic, and sinusoidal temperature dependence. (G) Performance and interaction graph of molecular commutation system exhibiting cubic temperature dependence on ionic strength. X axis direction is reversed in (G). Solid lines represent the objective profile, and dots represent the simulated system performance. Node colors represent oligonucleotide concentrations, and edge widths represent the corresponding dissociation constants. DNA-RNA interaction was simulated*

according to DNA-DNA NUPACK model. The simulation was performed taking into account complexes consisting of up to three strands (NUPACK max_compl_size = 3).

In addition, ***Fig. 7G*** *shows that nucleic acid networks can exhibit similarly complex, predefined dependencies on ionic strength. Since the free energy of hybridization depends logarithmically*[40] *on sodium concentration (in contrast to its linear dependence on temperature), for consistency, we chose the logarithm of the normalized sodium concentration as the independent variable (X):*

$$X_{ionic} = \log_{10}\left(a\left[Na^{+}\right]+b\right),$$

the constants a and b were chosen such that the sodium concentration range from 50 mM to 1 M is mapped onto the interval [0, 1] for X. Using a DNA set previously optimized to produce a cubic dependence on temperature (see Materials and Methods for details), we applied an evolutionary algorithm to identify a new set that realizes an analogous dependence on ionic strength.

To summarize, this remarkable feature of the molecular commutation networks allows them to receive a single cue of the environment (e.g., temperature or ionic strength) and react in many different ways. This phenomenon seems to be a perfect match for a hypothesis that the elevated body temperature during disease can act as a universal information carrier to all cells of the organism, potentially capable of triggering, in each cell type, its own multitude of independent signaling profiles. Initiating these programmable responses via temperature may be highly valuable since it does not dependent on mass transport of molecular messengers throughout the body.»

Accordingly, we have changed the title and the abstract to reflect these new results. The new title is "*Temperature- and ionic strength-independent or programmably dependent molecular signaling enabled by affinity-based molecular commutation networks*", and the new "Here we show" statement of the abstract is "*Here, we show that molecular commutation enables complex signaling systems that are independent of temperature and ionic strength and, even more generally, programmably dependent on these parameters.*"

This study introduces two completely novel ideas, that in no way have been mentioned in the cited works:

1. Nucleic acid–based molecular networks can stabilize other complex, higher-order systems that may lack any intrinsic compensation mechanisms.
2. Affinity-based systems provide a flexible and powerful means to implement signaling of predefined shape in response to variations in internal temperature.

We emphasize, that despite more than 30 years of active development in biocomputing, our Nature Chemistry paper remains, to our knowledge, the only work to have demonstrated the ability to generate signals of predefined shape (e.g., sin(x)). Similarly, our present manuscript is the first to show that molecular systems can generate responses of programmable form in response to temperature changes. Therefore, previously reported observations of temperature independence in specific enzyme sets (i.e., circadian rhythm systems) do not anticipate these results and do not compromise the novelty of our study.

Point-by-point response regarding novelty of the Results of the initially submitted manuscript (before revision).

However, we also cannot agree with the Reviewer's conclusion regarding the lack of novelty in the original manuscript compared to the cited studies on enzymatic networks, for the following reasons:

1. These prior works describe only enzymatic networks. None of them even mention affinity-based networks and their compensation possibilities;
2. The general theory and written claims of these prior works straightaway concludes that the results of our study are impossible, highlighting the limited scope of their theory in the context of our systems;
3. We show that affinity-based systems can provide compensation with arbitrarily high accuracy, which is almost impossible for enzymatic networks from prior works;
4. The temperature compensation in these prior works might not be a consequence of enzymatic compensation, but a consequence of a different mechanism, potentially, ours.

We will now provide more detailed explanation of these statements.

Prior works envisioned compensation only via enzymes

In all of the cited works the attention is given only to enzymatic systems. At no point in any of these articles — neither in the Introduction, nor in the Results, nor in the Discussion — is it suggested that such compensation could be achieved using affinity-based systems. However, modifying the sequence of an enzyme in order to precisely tune the activation energy while preserving its catalytic function and substrate specificity is an incomparably more difficult (if at all possible) task than tuning mutual affinities of several macromolecules. Therefore, our novelty is that we are the first to recognize that compensation of temperature and ionic strength can be implemented in such a simple and efficient manner.

Published theory for enzymatic networks leads to erroneous conclusions about affinity-based systems

The study by Ruoff, Zakhartsev and Westerhoff, (2006), describing the general theory of compensation in enzymatic networks claims the following: «*Temperature compensation of an output flux is not possible for: (i) any (reversible or irreversible) chain or loop of (first-order) reactions or a branched network that has only one output flux and a product insensitive first step*». This statement straightforwardly suggests that according to their theory, our study and our results should not be possible: all temperature-independent gate realized in our work fall within the definition of "systems impossible to compensate." This contradiction points to significant mismatch between their overall theoretical setup (including definitions of what is compensation, what is output, etc.) and the systems we study.

Compensation with enzymes is incomparably harder to achieve than using our mechanism

According to the same study, a necessary condition for observing compensation in such networks is that the activation energies of the participating reactions coincide in such a way that their weighted sum equals zero (weights can be either +1 or -1 depending on network topology). Such a condition is unlikely to be realized in a considerable number of real enzymatic networks, given that enzyme–substrate interactions are far more likely to be shaped by evolutionary pressure toward catalytic optimization than by adherence to such a strict constraint.

In our view, this is precisely why the possibilities of such compensation remain very limited in the cited works. For example, in the study by Kidd, Young, and Siggia (2015), temperature independence of circadian rhythms was demonstrated using the following figure:

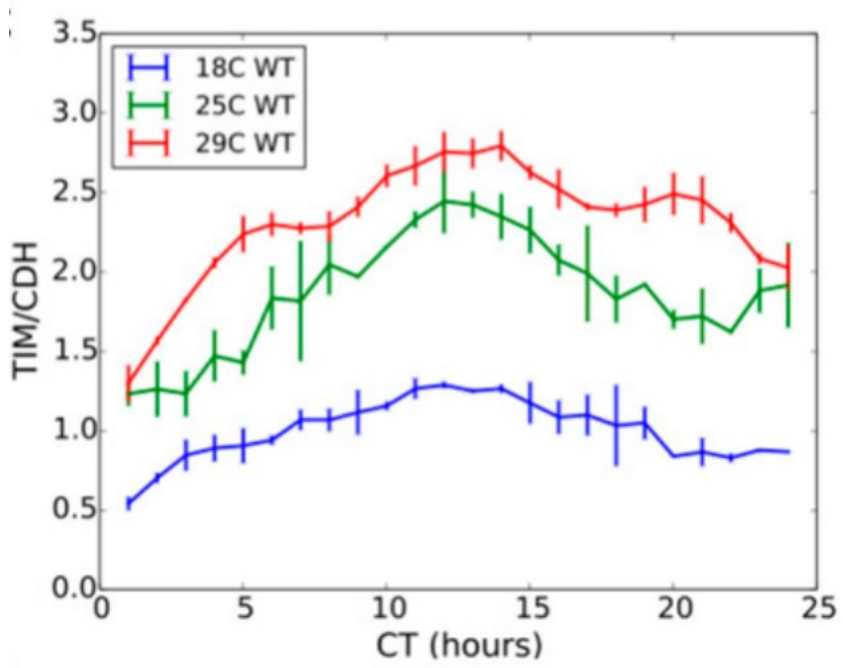

Although this figure demonstrates that the shape of the oscillations is preserved up to scaling, but the absolute signal amplitudes change significantly with increasing temperature.

Conversely, we emphasize that our low-affinity systems can make dramatically better compensation. In fact, it would be **particularly surprising** if one were able to identify or artificially construct even a comparable number of distinct signaling systems to those presented in our work using enzymatic networks alone. In summary, we do not believe that the existence of prior concepts based on enzymatic networks should preclude the development of new, more effective, and simpler theoretical frameworks.

Has the enzymatic compensation been really proven and validated as the source of circadian clocks temperature compensation?

The work of Kidd, Young, and Siggia (2015) provides one of the most detailed analyses of this temperature compensation in circadian clock system. The authors present an abstract mathematical model that predicts oscillatory behavior and is capable of compensating for temperature-induced variations in the oscillation period (but not amplitude) under condition of matching of three activation energies (in the original notation $E_K = E_r – E_f$):

"The effective rate d_X will be temperature-independent as long as $K >> 1$ at room temperature and $E_K = E_{d^*}$. In this case, the model will behave exactly as the temperature-compensated Goodwin oscillator discussed above and will show a simple scaling of oscillation shape with temperature. Additionally, although the model is abstract, it shares a number of features with the real circadian clock"

The authors then demonstrate, in a series of *in vivo* experiments, that the oscillation period is independent of temperature. However, they do not test whether the conditions required for temperature independence according to their theoretical framework are satisfied (namely, the exact matching of activation energies). Thus, while the empirical observation of temperature independence itself is not in question, the fundamental mechanism underlying this behavior remains uncertain. As in all *in vivo* experiments, it is difficult to isolate the contribution of individual mechanisms. Specifically, the authors write:

«New data from the laboratory of Patrick Emery (39) suggest that calcium signaling is essential for circadian temperature responses and that the above-mentioned neural pathways converge by calcium-dependent degradation of TIM. Given that there are clearly multiple pathways present for communicating temperature to the clock, it may be difficult to eliminate circadian temperature entrainment in an intact mutant animal…

…Elucidating the biochemical mechanisms of temperature compensation in the circadian clock remains an open problem»

Therefore, although we do not exclude the possibility that in this particular case the required matching of the thermodynamic characteristics of the participating molecules may indeed occur and thereby explain the observed behavior, we believe that this should currently be regarded only as a hypothesis. Interestingly, more recent analyses (e.g., doi.org/10.1038/s41540-023-00268-7, Fig. 3) indicate that for several models describing circadian oscillations, even when the parameters of the corresponding enzymatic networks are artificially optimized *in silico*, the resulting

temperature independence remains far from ideal (especially when compared with experimental *in vivo* observations). This suggests that additional compensatory mechanisms may be involved.

Temperature independence of chemotaxis does not stem from enzymatic compensation alone

An even more interesting situation arises in the case of bacterial chemotaxis (Oleksiuk, 2011). In this study, the authors explicitly tested whether the observed temperature compensation is consistent with the predictions of the theoretical model. They concluded that the enzymatic network alone cannot account for the experimentally observed temperature compensation. Instead, the compensation was partially attributed to an increase in the translation rates of certain participating proteins, through a mechanism that remains not fully understood.

«The observed mechanisms of temperature compensation include (1) opposing temperature effects on the activity of individual receptors in mixed complexes and on sequential steps in signal transduction, (2) similar temperature dependences of opposing enzymatic reactions, and (3) counterbalancing of the effect of temperature on reaction rates via preprogrammed temperature dependence of enzyme levels to achieve nearly optimal performance at the growth temperature.

…Due to the differences in their activation energies, the increases in the rates of phosphorylation and dephosphorylation of CheY with temperature are only partly compensatory; the compensation of temperature effects by mixed receptor complexes is apparently also imperfect. As a result, wild-type cells still show a significant increase in the steady-state level of FRET in the temperature range between 10C and 30C (Figure 3B). Such imprecise temperature compensation may be required for effective thermotaxis toward a preferred temperature.

…As the levels of mRNA for these constructs were similar at 27C and 37C (measured using real-time PCR, data not shown), this differential regulation of protein levels with temperature must arise from translational control of cheR, whereby the upstream fragment preferentially upregulates the level of CheR translation at 27 C.»

This straightforwardly suggests existence of other compensation mechanisms.

Molecular commutation networks can be the reason for compensation of higher-level systems (including mentioned enzymatic networks)

In this context, it is important to highlight a key point from our prior work describing molecular commutation networks (Nikitin (2023)). There, it was experimentally demonstrated that molecular commutation can remarkably affect protein translation: for example, how an oligo of maximum non-complementarity to the gene's sequence can still affect its expression via an affinity-based nucleic acid network.

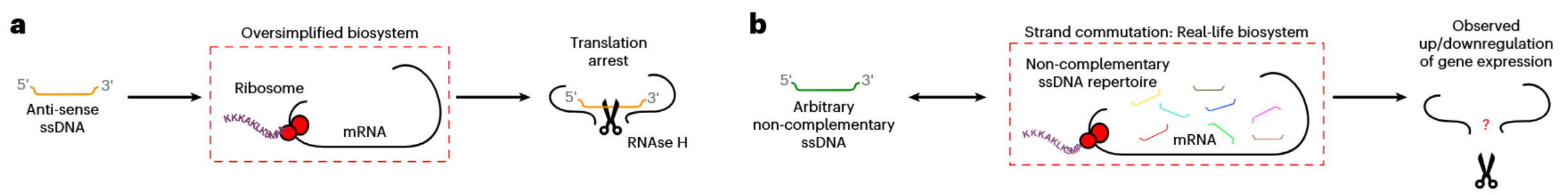

Accordingly, molecular networks that involve protein translation may inevitably, at some stage, become subject to effects of molecular commutation (for example, such effects may have been the not fully understood source of the selective changes in expression levels reported in Oleksiuk (2011)).

Our new results on (in Fig. 7), we have further demonstrated that molecular commutation effects can compensate for virtually any temperature or ionic-strength dependence of an existing system, including complex and non-monotonic behaviors. Thus, their observed results, namely, temperature independence due to preprogrammed increase of translation level of certain genes, may have potentially been consequence of our mechanism.

Novelty with respect to ionic strength compensation

As the reviewer noted, compensation for changes associated with fluctuations in ionic strength represents a distinct and interesting aspect of our study. At the same time, it may appear as an expected consequence, since this effect is "similar to temperature, because both weakens interactions". However, it is important to emphasize that this "consequence" is neither obvious, nor true in general case, particularly for enzymatic or other protein networks. At the same time,

as shown in Fig. 7G, our affinity-based systems can potentially stabilize other systems, including enzymatic ones.

Thus, prior to our work (including the studies cited by the reviewer), it was far from obvious how compensation of arbitrary perturbations in protein or other biochemical networks arising from changes in ionic strength could be achieved. Our results provide a simple and convincing mechanism for such compensation, thereby establishing a clear conceptual advance.

In conclusion, although the hypothesis of enzymatic compensation as a mechanism explaining compensation in two natural systems (chemotaxis and circadian rhythms) was proposed prior to our work, it does not preclude the existence of other, simpler and more effective underlying mechanisms, such as the phenomenon described in our work. The novelty of a potential reason should hardly be compromised by its known consequence. We hope that the given arguments are sufficient to demonstrate that in our work, we have advanced significantly and in a completely unexpected direction compared to the works cited by the Reviewer.

3.2.The temperature compensation mechanism proposed by the authors has the same explanatory difficulties – if not worse, as it would require precise compensatory reactions across a multifunctional network as opposed to a time-keeping motif. Therefore, it seems questionable if the proposed mechanism is plausible in vivo.

There are well-known natural systems that "require precise [compensatory] reactions across a multifunctional network" and function remarkably well *in vivo*, such as blood coagulation cascade. Even minor perturbations or mutations (i.e., slight deviations from these precise reactions) typically leads to severe and often fatal diseases.

On the contrary, in the present work we demonstrate, for the first time, a compensation mechanism within a single-functional network in a sense that molecules are required to optimize only a single property: their affinity toward interaction partners. By comparison, enzymatic systems, whether in the coagulation cascade or circadian rhythms, must be tuned to simultaneously balance multiple parameters for each molecule, including catalytic function, reaction rates, substrate selectivity etc.

This point, namely the necessity of tuning in enzymatic networks, is explicitly acknowledged by Kidd, Young, and Siggia (2015): "*There is certainly parameter tuning in this model as there is in the network model, which is perfectly reasonable because natural evolution has plausibly shaped the phenotype of the circadian oscillator.*" In the context raised by the Reviewer, our systems are, therefore, dramatically simpler to tune and, consequently, are no less plausible *in vivo* than the highly-tuned enzymatic cascades.

Considering time-keeping motif vs absolute concentration preservation comment, we note that the objective function we selected (preservation of absolute concentrations) constitutes a substantially more demanding requirement than the preservation of a given motif (which is a function of the concentrations). If the concentrations themselves are maintained, then any derived functions of those concentrations are also preserved; the converse, however, is not true. We deliberately chose absolute concentrations as the target parameter because they represent the most difficult objective to achieve. Clearly, affinity-based systems that only need to maintain the stability of relative parameters (e.g. True-False difference) are even easier to set up (see also our reply to comment 3.9).

3.3.Many of the explanations in this regard ("The phenomenon of topological compensation described above allows a reconsideration of both key evolutionary mechanisms and contemporary regulatory systems") are exaggerations and there is no proof that the described mechanisms have been evolved by cells.

We certainly did not intend to claim that these statements have been proven, as that would indeed be misleading: we only aimed to provide an outlook on the possible broader implications of our

work. To be more precise, we have softened the wording of this statement:

"The described phenomenon of topological compensation in affinity-based molecular networks provides a number of interesting ideas and possibilities for both evolutionary theory and molecular regulatory systems in general"

3.4.Title and abstract do not convey that this work is mainly theoretical in nature and focused mainly on DNA-oligos.

We agree with your comment regarding the abstract and have made the corresponding revisions. The key changes are underlined:

"The equilibrium constants of chemical reactions fundamentally depend on temperature, posing challenges for living systems. However, many conformer organisms do not maintain stable internal temperature. This raises the question: can molecular signaling pathways inherently resist temperature susceptibility? Molecular commutation is a recently discovered, fundamentally distinct mechanism of biological information processing and storage within molecular association/dissociation reactions. Here, we show that molecular commutation enables complex signaling systems that are independent of temperature and ionic strength and, even more generally, programmably dependent on these parameters. Using examples of various DNA logic gates, receptor-activator networks, and systems with complex input–output relationships (e.g., computed as algebraic functions), we demonstrate computationally that introducing compensatory reactions in these networks can render their signaling independent of temperature and ionic strength. We experimentally validate such independence for a case of a YES-logic gate. Finally, we computationally demonstrate networks with outputs that follow predefined functional forms of temperature and ionic strength (e.g., sin(T), where T is temperature). The presented intrinsic capabilities of affinity-based networks provide a remarkable homeostasis and signaling control mechanism that may be used by biological systems of arbitrarily high complexity."

As concerning DNA in the title, our main result is that affinity-based networks can implement signaling that is temperature independent or programmably dependent, while DNA merely serves as a convenient test case exclusively due to existence of convenient prediction tools. Clearly, any sets of molecules possessing similar thermodynamic characteristics (an imperfect correlation between binding enthalpy and entropy, which gives rise to "easy-to-melt" and "hard-to-melt" pairs) would be expected to exhibit the same effects. Once predictive tools are developed for other classes of molecules, this principle can be straightforwardly extended to them. Therefore, we would prefer to retain the current title and avoid explicitly mentioning DNA, as we believe that such a title would be misleading.

We would also like to provide a concrete example illustrating why we prefer not to narrow the title. The title of the original experimental work on molecular commutation referred specifically to DNA, although the text of the paper explicitly stated that the underlying principles are applicable to other classes of molecules including proteins, as also noted by the Reviewer in *News and Views (10.1038/s41557-022-01115-8)*.

In 2025, a paper (Parres-Gold et al., *Contextual computation by competitive protein dimerization networks*, 2025, 10.1016/j.cell.2025.01.036) published in *Cell* largely reproduced the core ideas of that original study without properly citing our work. The main claim of their paper was that proteins can also interact in a many-to-many manner and that such interactions could enable complex computations. Contrary to our experimental study, their study was 100% theoretical and no specific proteins were identified; instead, the discussion relied on 'abstract' proteins with assumed binding parameters. The "novelty" of their work arises solely from the fact that our title refers to DNA, whereas their study theoretically treats abstract protein systems (which in fact are just affinity matrices, not the real molecules); this difference is in fact superficial.

Even more remarkably, in this article, the authors specified in the author contribution statement that they were sole discoverers of this information processing mechanism. Within the ongoing discussion, *Cell* has already published an Erratum [Cell *188*, 1984–2002.e1–e6], conceptually rewriting the article's highlights, editing author contribution statement, etc. To avoid similar situations, we would prefer to explicitly include these straightforward implications within our manuscript, in particular by not limiting the title to DNA this time.

Moreover, according to the suggestions of Reviewers 1 and 2, we amended the Discussion noting that affinity-based protein networks could, in principle, also exhibit temperature compensation (see also the end of Section 3.1):

*"**Supplementary Note 1**. Analysis of the feasibility of temperature compensation in the context of protein–protein interactions.*

As previously noted, the existence of temperature compensation in networks of interacting oligonucleotides is enabled by the existence of "hard-to-melt" and "easy-to-melt" pairs. Importantly, in this context, nucleic acids serve merely as a convenient model system that allows straightforward evaluation of their thermodynamic properties. More generally, any biomolecules that exhibit distinct temperature-dependent behaviors are likewise capable of demonstrating analogous compensatory effects.

To demonstrate this, we consider the case of protein–protein interactions. For the analysis, the Stites (1997) dataset[1] was selected. Because, for such interactions, the enthalpy typically exhibits a strong temperature dependence[1] (i.e., the change in heat capacity ΔC_p is significantly different from zero), only data for which ΔC_p was directly measured were included in the analysis. Otherwise, the use of enthalpy and entropy values obtained at a single temperature (e.g., from isothermal titration calorimetry performed at a single temperature point as is the case in most other datasets) may be misleading when analyzing the temperature dependence of the interaction.

The distribution of dissociation constants at 25 and 37 °C for protein–protein interactions is shown in Supplementary Fig. 1A. Notably, the same characteristic feature observed for the analogous distribution of DNA–DNA interactions (Fig. 1A) is evident here: for a given K_d at 25 °C, there exist protein–protein pairs with substantially different K_d values at 37 °C.

Moreover, a similar pattern can be observed even in the case where one of the proteins remains unchanged while single-point mutations are introduced into the other. For instance, an analogous distribution is shown in Supplementary Fig. 1B for a mutational scan[2] of an anti-HER2 antibody reported in Kelley (1993). Notably, the V_H-F100A and V_L-Y92A antibody variants exhibit nearly identical K_d values at 25 °C (1.7 and 2.4 nM, respectively), yet display markedly different K_d values at 37 °C (9769 and 452 nM, respectively), corresponding to a 21.7-fold difference.

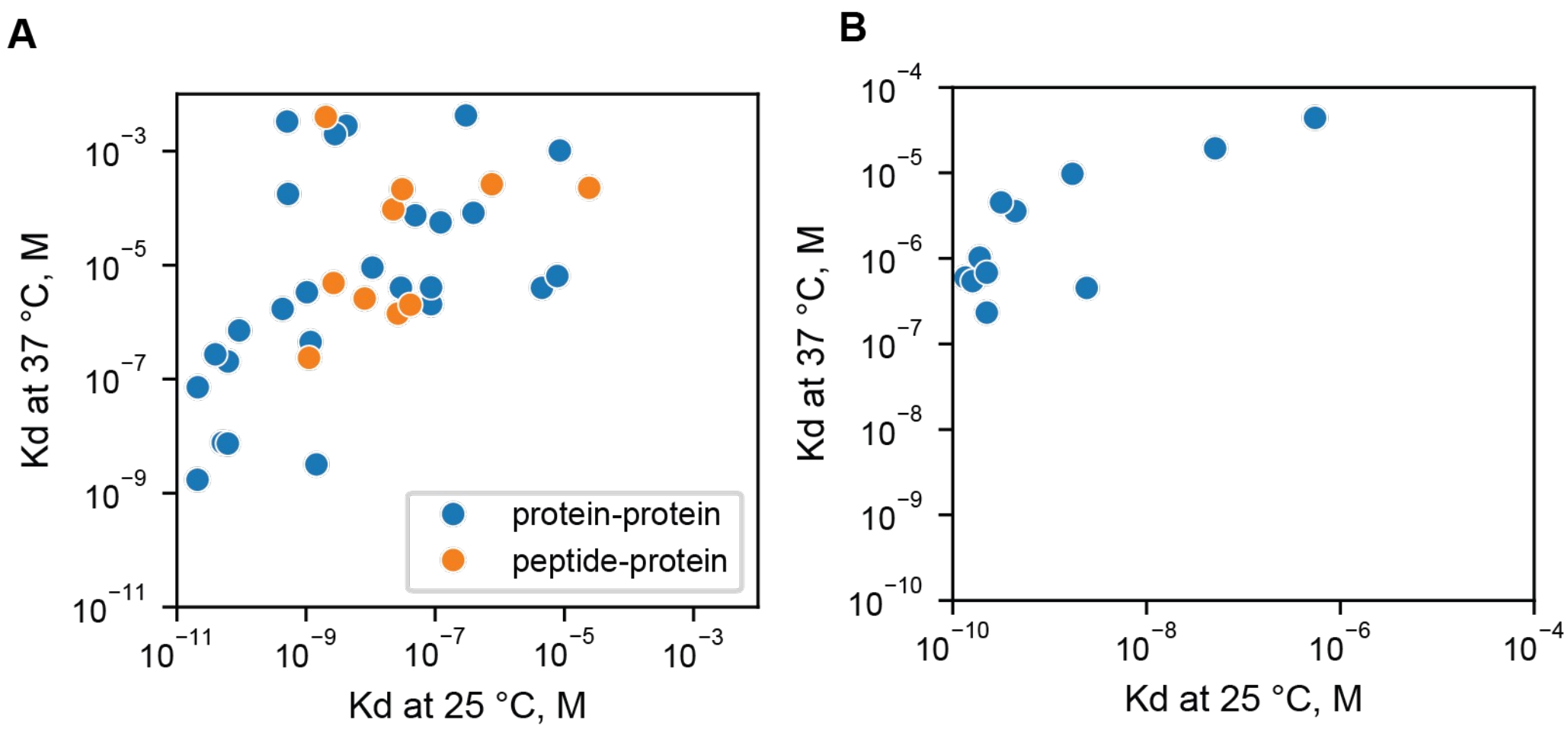

Supplementary Fig. 1. *Kd distributions at 25 and 37 °C for protein-protein interactions for (A) various protein-protein and protein-peptide pairs from Stites 1997[1]; for (B) single-point mutational scan of an anti-HER2 antibody from Kelley 1993[2].*

…

1. Stites, W. E. Protein−Protein Interactions: Interface Structure, Binding Thermodynamics, and Mutational Analysis. Chem. Rev. 97, 1233–1250 (1997).

2. Kelley, R. F. & O'Connell, M. P. Thermodynamic analysis of an antibody functional epitope. Biochemistry 32, 6828–6835 (1993).”

3.5 Interestingly, the experiments show that the NUPACK-predictions are not very accurate, while the main part of this work is based on exactly this software, but this contradiction is not discussed.

Thank you for this important comment. We have inserted a dedicated section in the Discussion to specifically address this issue.

*“**Limitations***

We should note, that our work relies on NUPACK calculations, which in some cases might be far from precise (for example, see screening in Fig. 3B). Does this imply that our conclusions drawn from its predictions are incorrect? From the fundamental point of view of the compensation feasibility the only condition required is the existence of biomolecules exhibiting sufficiently distinct temperature-dependent behaviors, which is definitely true both for nucleic acids and proteins, and most likely, for any other biomolecules.

On the other hand, the phenomenon of compensation (like molecular commutation signal processing more broadly) can be considered both at the level of affinity matrices and at the level of the real molecules that realize these matrices. If we consider only the first level (affinity matrices and law-of-mass-action equations), the compensation is exact, and no additional assumptions are required. In this context, NUPACK and similar prediction tools limit us only in this second aspect of correct identifying which specific molecules should be chosen to achieve a desired matrix.

This limitation can be addressed in several ways. For example, one may restrict attention to specific subsets of oligonucleotides for which NUPACK performs reliably: for example, as observed in the screening Supplementary Fig. 11, prediction accuracy was noticeably higher than in Fig. 3B, possibly due to shorter sequence lengths. Alternatively, specialized design strategies can be employed, such as the use of domain-based architectures for oligonucleotides based tasks.

A related question is whether certain target matrices might be inherently unrealizable with chosen type of molecules. In our view, the diversity of biomolecules (oligonucleotides or proteins) and their affinity continuum[21] is so vast that this should not pose any limitation. Moreover, this continuum can be expanded in almost unlimited manner — for example, through the use of non-canonical or chemically modified bases as monomers, or by employing molecules with nontrivial secondary or tertiary structures. The latter, for example, is intrinsic for proteins; and can also be achieved with DNA (for example, see metaDNA[48]).

Finally, our work highlights the importance of further developing predictive tools, particularly with an emphasis not only on high-affinity systems and structure prediction, but also on the quantitative characterization of weak interactions: as demonstrated in this and our previous studies[21], such interactions carry substantial and still unexplored biological significance."

3.6.Further it is unclear if optimization at two temperatures (25 and 50 degrees Celsius) is sufficient to get a flat response in all cases; it could well be that in some cases, the optimization procedure, because it is fine-tuned, would miss a complete compensation. For the large systems, the result looks over-optimized, because the response is unrealistically flat. Biological systems have to be robust in many ways and are never optimized so strongly only in one regard as done here.

Thank you for pointing this out. We agree that the manuscript did not present this point with sufficient clarity. To address this, we have moved the relevant results from "*Exploring gates at various conditions*" to a dedicated subsection, "*0.1–50 °C temperature range and stability at intermediate conditions*", which specifically focuses on compensation across intermediate temperatures.

In particular, we show that instead of a single compensatory oligonucleotide, a compensatory cascade can be used, in which individual components gradually "switch off" as temperature increases. However, it should be noted that this strategy is only necessary for very broad ranges such as 0.1–50 °C. For narrower ranges, simple optimization at the boundaries is sufficient. For example, the experimental YES gate was optimized only at the boundaries of the range, yet it still demonstrates good performance across the entire interval.

To further illustrate this point in more complex systems, we optimized a random receptor–activator network (N = 100, with the same topology as in the main text) to exhibit a flat response across intermediate temperatures as well. The corresponding results have been included in the Supplementary Materials.

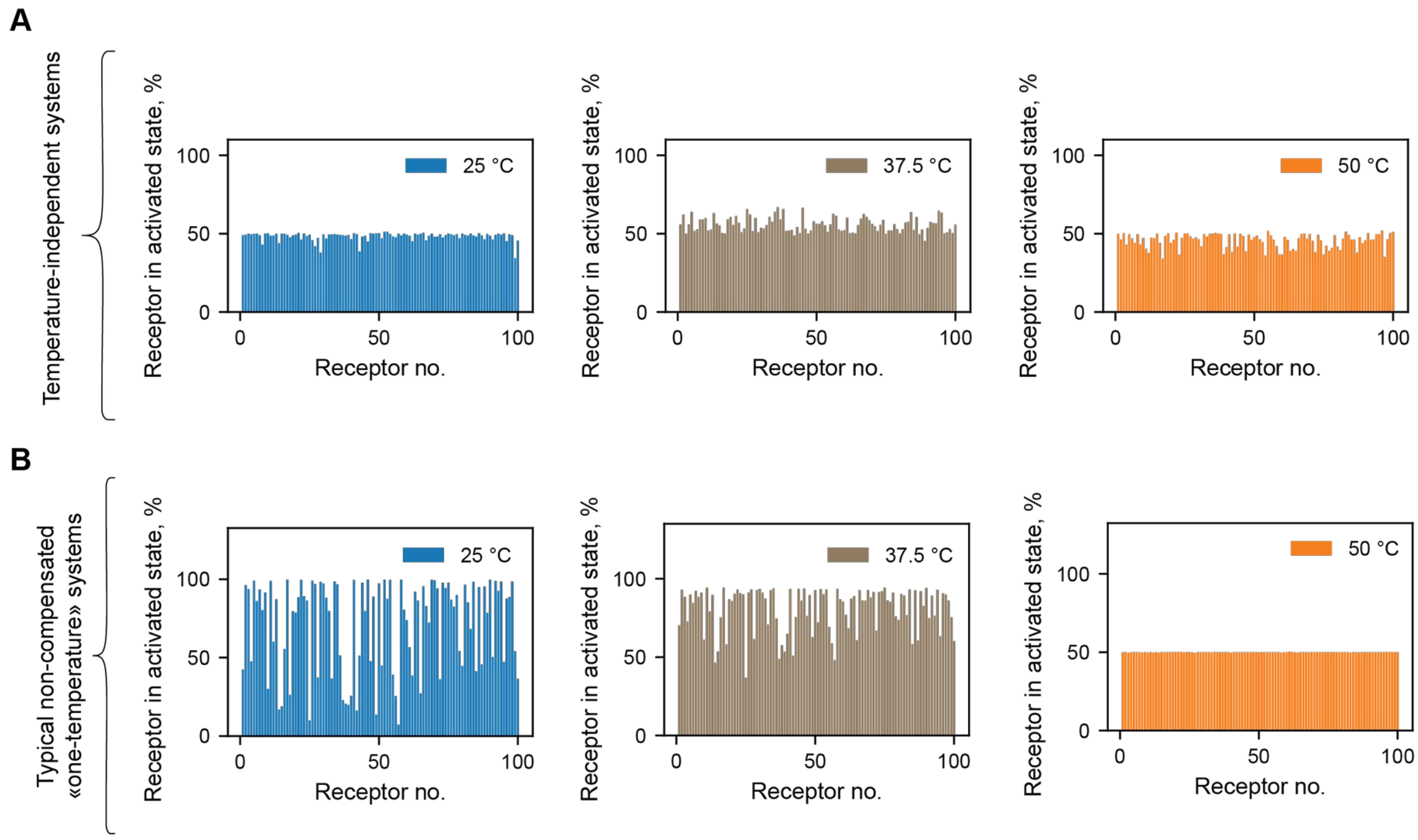

Supplementary Fig. 14. *Performance of random receptor-activator network at intermediate temperatures. (A) Temperature-independent system (B) Typical system without compensation, optimized separately to have predefined activation levels at 50 °C only.*

With regard to over-optimization, we agree that real biological systems must be robust in multiple respects, and it is unlikely that natural systems aim for absolute temperature stability at the expense of all other characteristics. We have added corresponding discussion in the Results section:

"The data in Fig. 5 show an ideal level of compensation that may even be perceived as unrealistic for biological systems. In practice, biological systems evolve to optimize multiple characteristics simultaneously, often trading off some properties against others. At the same time, we consider it important that, during certain evolutionary stages, the availability of such a tool of ideal compensation may have played a significant role in adaptation to specific environmental conditions and in enabling the emergence of other functional traits."

However, this ideal level of compensation should not be interpreted as a disadvantage. An ideally functioning system can always be deliberately adjusted to reduce performance, whereas achieving ideal performance from a suboptimal system is not guaranteed.

This comment about over-optimization is also strongly connected with comment 3.9, please see the follow-up discussion there.

3.7.In summary, the theoretical treatment on its own is neither methodologically nor conceptually a major advance and their framing as 'fundamentally novel property' (line 81) is not fully justified in light of, for example, the significant body of literature on temperature compensation in circadian clocks.

We thank the reviewer for the opportunity to cite and address these prior studies on circadian rhythms in detail and to provide a better context for our work.

We hope that our detailed responses in Section 3.1 and the corresponding revisions to the manuscript clearly present the key differences between mechanisms presented in our work and the cited works. To reiterate, we see the novelty of our work in: (1) compensation by affinity-based systems; and (2) the demonstration that such systems can achieve levels of stabilization (both of

themselves and of external systems) that are essentially unattainable within other paradigms, and therefore constitute a "fundamentally novel property.".

Moreover, this original framing was only restricted to the molecular commutation networks, and thus, in our view, cannot be compromised by prior works on circadian clocks:

"*In this work, we report a fundamentally novel property <u>of molecular commutation networks for biological systems</u>: these networks can overcome the inherent Boltzmann temperature dependence, preserving their signal processing functionality across a wide temperature range with arbitrarily high accuracy*".

Another reason for using such description is that many studies on molecular commutation systems (or "dimerization networks") currently aim to expand the repertoire of achievable computations, while our work highlights a completely unexplored aspect of these systems.

If, however, the reviewer feels that the revisions and clarifications provided do not fully address the questions raised or adequately convey the novelty of our work, we would be happy to address any follow-up comments.

3.8 The experimental implementation of a single temperature compensated YES-gate with statistics based on n=2 replicates and no experimental verification of the salt-compensation does not constitute a thorough experimental study of the many theory claims made in this work.

To address the issue of insufficient replicates, we repeated the experiment with the temperature-independent YES gate at n=3 technical replicates, moreover, with another oligonucleotide set from other manufacturer to demonstrate that the gate functions stably as anticipated:

"

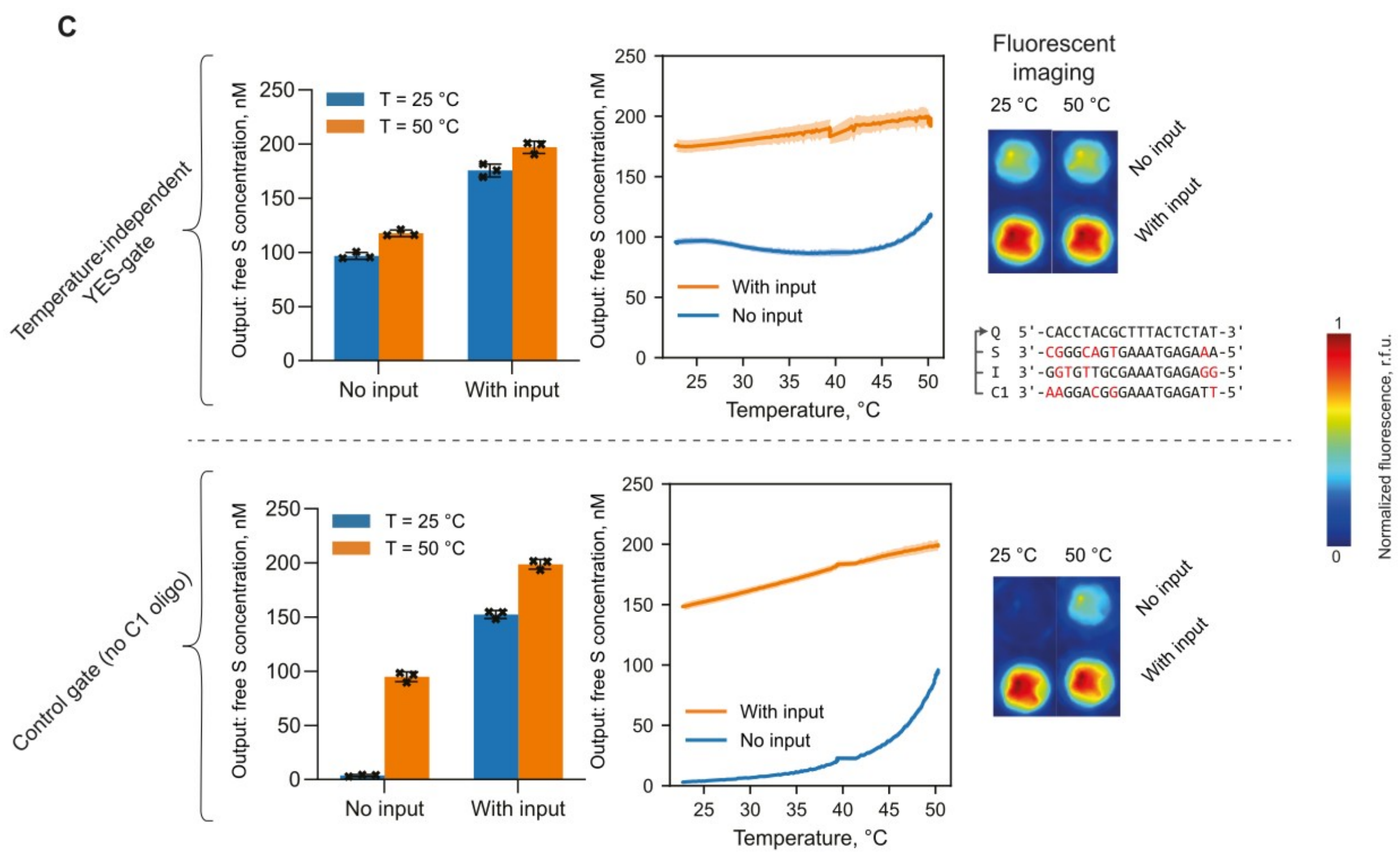

Fig. 3. Experimental realization of a temperature-independent YES gate. (C) Experimental performance of the temperature-independent and control YES gates. Left panel shows endpoint performance at 25 and 50 °C, right panel shows melting curves and fluorescent images of the

reaction wells. Bar graphs show mean ± s.d., melting curves show mean values (solid line) with 95% confidence intervals (shaded area), n = 3 independent samples for both cases. Due to the temperature dependence of Cyanine3 quantum yield, absolute fluorescence values differ between 25 °C and 50 °C. For clarity, images were linearly adjusted so that control wells containing Cyanine3 exhibit the same mean fluorescence. Pre-heating-cooling cycle (25-50-25 °C) was used in the experiment to ensure equilibrium at the starting point of the melting curve. Without the cycle, the gate reaches equilibrium within 30 min at 25 °C, kinetics is shown in ***Supplementary Fig. 8.*** *Please note that some sequences are written from 3' to 5' for demonstrating mismatches."*

Furthermore, we experimentally demonstrated independence from ionic strength in Fig. 4. The following paragraph has been inserted in the "Stability at intermediate conditions and ionic strength independence" section of the Results:

"To experimentally implement a salt-independent YES gate, we employed the same strategy as for the temperature-independent system, and the experimental performance of the salt-independent YES gate for both variations in Mg^{2+} and Na^{+} concentrations is shown in ***Fig. 4 C, D****. To implement these gates, we performed a screening procedure analogous to that shown in* ***Fig. 3B****; the corresponding results are presented in* ***Supplementary Fig. 11****. Interestingly, in this screening, a better correlation is observed between NUPACK predictions and experimentally measured affinities compared to that* ***Fig. 3B****. Based on this experimentally measured affinities, candidate input and compensatory oligonucleotides were selected, and the concentrations of all participants were optimized using Bayessian optimization. For variations in sodium and magnesium concentrations, identical sequences were used, with only the concentrations adjusted. "*

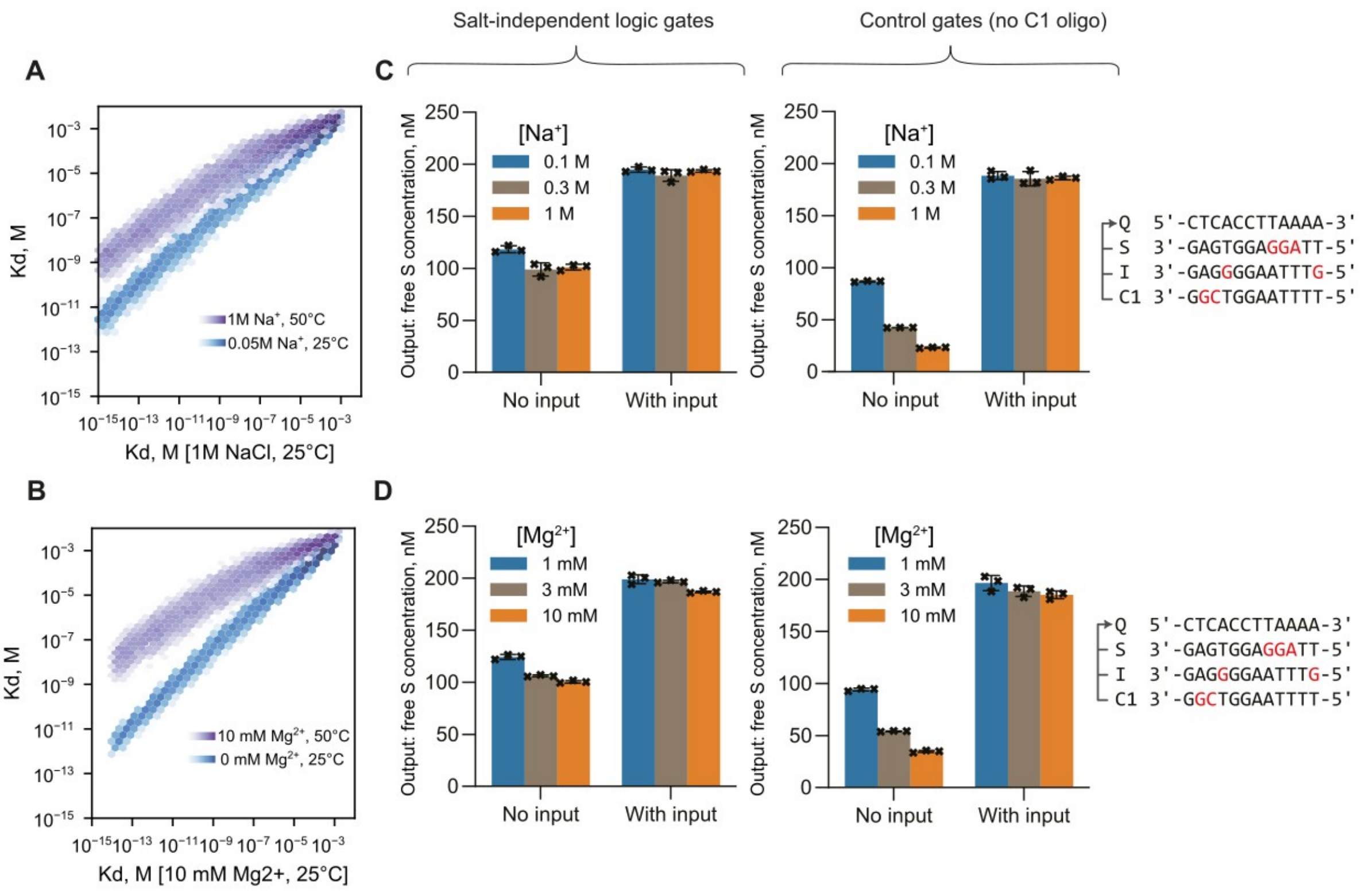

Fig.4. Experimental realization of salt-independent gates. ... (C) Experimental performance of salt-independent YES gates across Na^{+} concentrations ranging from 0.1 M to 1 M, magnesium

concentration is zero. The left panel shows the performance of the optimized gate with compensatory oligonucleotide, while the right panel shows the same gate without compensation. (D) Experimental performance of salt-independent YES gates across Mg^{2+} concentrations ranging from 1 mM to 10 mM, sodium concentration is zero. The left panel shows the performance of the optimized gate with compensatory oligonucleotide, while the right panel shows the same gate without compensation. For (C, D) data are shown as mean ± s.d. (n= 3 independent samples). In both cases, the system reaches equilibrium in less than 5 min; the kinetics are shown in Supplementary Fig.12. Please note that some sequences are written from 3′ to 5′ for mismatch highlighting."

Results of the screening to select appropriate oligonucleotides for the salt-independent gates has been inserted into the Supplementary Information: "

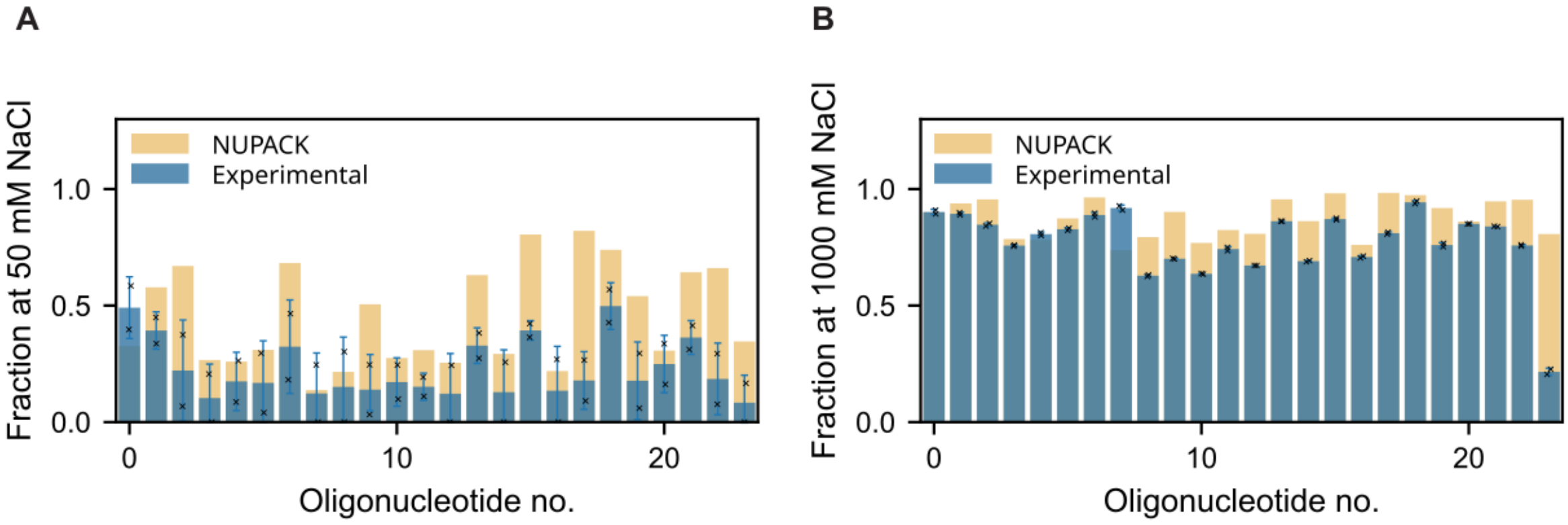

Supplementary Fig.11. *Accuracy of NUPACK affinity predictions at 50 and 1000 mM of Na^{+}. Data are presented as mean ± s.d., expressed as the concentration of the resulting duplex if both strands are mixed at 1 µM (n = 2 independent samples). Each bar represents a distinct oligonucleotide pair."*

Kinetics of the gates' equilibration has been inserted into the Supplementary Information:

"

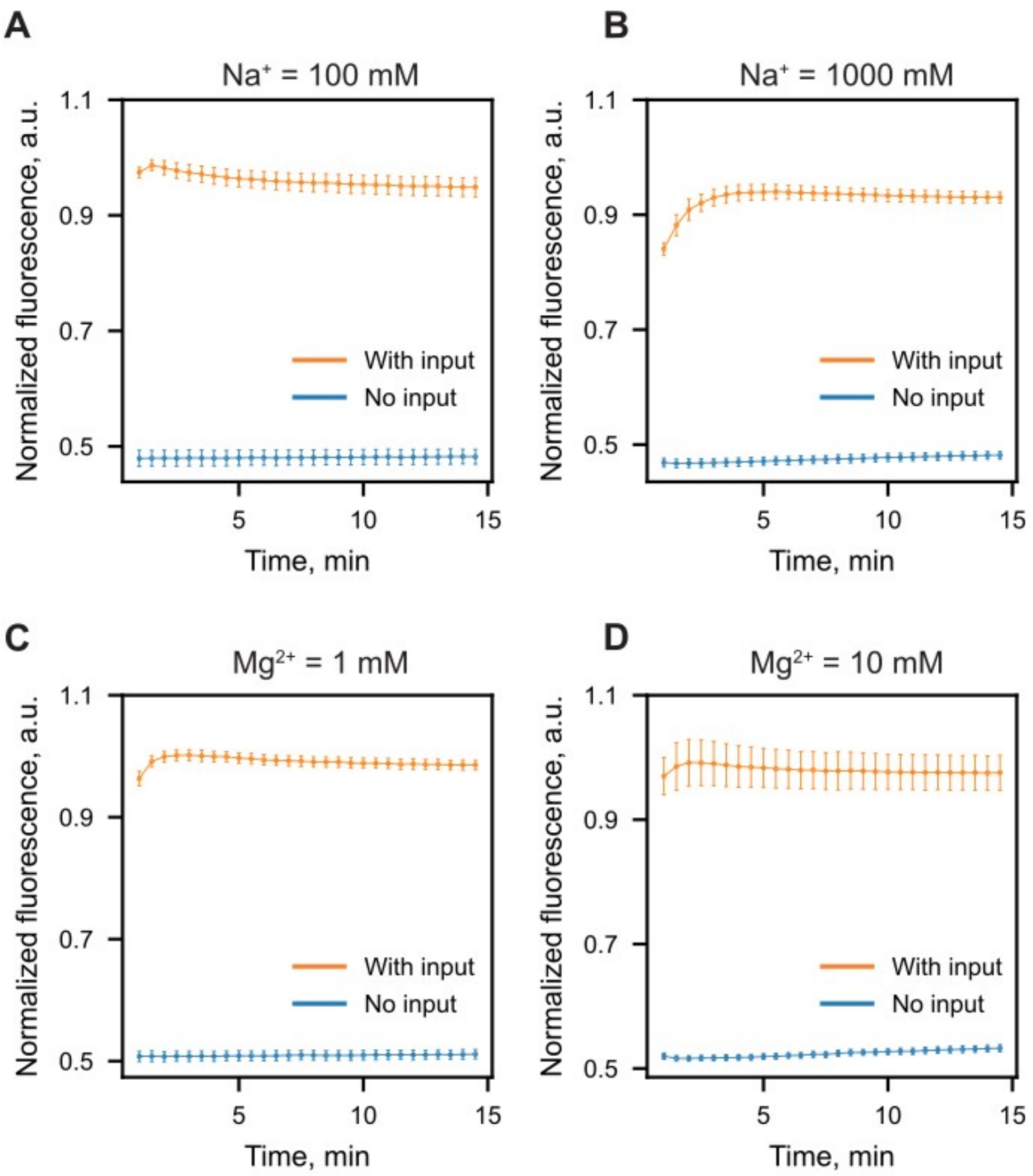

Supplementary Fig.12. *Kinetics of salt independent YES-gates from Fig.4 following the addition of the input oligonucleotide. Other components (S, Q, and C) were pre-equilibrated for 1 hour. Data are presented in relative fluorescence units as mean ± s.d., n = 3 independent samples. Fluorescence is normalized to the signal of a sample containing only S at the same concentration. (A, B) show the gate performance optimized for robust operation under sodium variation, while (C, D) show the corresponding results for magnesium variation."*

With regard to comprehensive wet-lab validation of all systems presented in the work, the primary limiting factor at this time is the inaccuracy of current prediction tools, such as NUPACK (which is unsurprising, as NUPACK was not originally developed with a focus on weakly complementary systems).

Such validation is, in principle, still feasible using the approach we describe (i.e., large-scale screening followed by selection of suitable candidates). However, the required scale of all these screenings together is so large that, in our view, a much more useful and systematic strategy would be to develop novel predictive tools using experimental data generated from such screens. This direction, however, lies far beyond the scope of the present work. Moreover, we believe that the already presented screening data are highly important (as are similar data reported in *Nature Chemistry*), as they clearly demonstrate to the scientific community the need for and value of developing such predictive tools.

Thus, once improved predictive frameworks become available, experimental validation of all large and complex systems presented in the work is straightforward.

3.9.In this regard, their "arbitrarily high accuracy" (line 84) to topological compensation claim feels stronger than what is shown – especially, when keeping in mind that this is much more a result of topology and careful parameter tuning than of topology alone.

To clarify what we mean by *arbitrarily high accuracy*, consider receptor systems as an example (the same reasoning applies to all other systems). To find such systems, we formulated an objective function defined as the sum of the absolute deviations between the target and actual receptor activation levels at a set of temperature points:

$$L = \sum_{T \in \{25\,°C, 50\,°C\}} \sum_{i=1}^{100} \left| R_i^{act}(T) - R_i^{target} \right|$$

Our claim is that we can identify system parameters (such as affinities, concentrations etc.) so that objective function becomes smaller than any (within reasonable limits) predefined threshold. In particular, for large systems, the parameters we obtained minimized this function so effectively that, as noted by Reviewer previously in the comment #3.6, "the result looks over-optimized, because the response is unrealistically flat" From our perspective, the ability to achieve such near-ideal performance clearly demonstrates the validity of our original *arbitrarily high accuracy* claim.

However, to soften the wording, we have changed "*arbitrarily high accuracy" to "nearly any desired level of accuracy".*

Considering parameter optimization, on the one hand, we agree that the compensation, like virtually any biological process, cannot operate if the system parameters are chosen entirely at random. On the other hand, since compensation can be viewed as stabilization of an already functional system (rather than the de novo construction of such a system), the question may be reformulated as follows: is the network topology alone sufficient to compensate (at least partially) for temperature variations, provided that the system operates as intended at a given reference temperature?

It turns out, that for this question the answer is positive. We demonstrated this using examples of YES and NOT logic gates, and have added a corresponding paragraph to the Results section, where the problem formulation is described, as well as Supplementary Note 2, which provides a detailed account of the results.

"Moreover, if the objective function is this True-False separation (instead of the preservation of absolute concentrations), the gate design problem becomes substantially simpler. In this case, virtually any YES gate with a topology that has a compensatory component (at a concentration comparable to that of the other components) will outperform a YES gate lacking such a compensator, provided that both exhibit identical performance at 25 °C (i.e., the same level of free S). This behavior is illustrated for both YES and NOT gates in Supplementary Note 2 and Supplementary Fig.3"

To substantiate this claim, we have performed additional simulations, as described in Supplementary Note 2 and Supplementary Fig.2.

"***Supplementary Note 2. Construction of temperature-compensated systems without parameter optimization across the entire temperature range***

In this section, we demonstrate that the topology alone of signal-processing systems with compensatory species is sufficient to achieve partial temperature compensation.

Let us consider the topology of a YES gate in its two variants: the first one without compensation (three oligos I, Q, S), and the second with compensation (four oligos I, Q, S, C). For the second one to be conceptually different from the first one, the concentration of C and the affinity of Q–C should not be zero or negligibly small, so let's fix these values to some reasonable values, for example, concentration of the compensator C to 5 µM and the dissociation constant of Q–C at

*25°C to 1.7 nM (−50 kJ/mol). Now, consider random gates of these two topologies that have the same level of free S at 25 °C, for example, 100 and 200 nM in the absence and in the presence of input, respectively. Gate performance is typically defined as the difference between outputs in the True and False states (i.e., with and without input), which in this case is 100 nM (**Supplementary Fig.3A, D**). How would the True-False difference behave at 50 °C for non-compensated and compensated groups of gates?*

*We have generated 100 distinct parameter sets for both groups of gates. **Supplementary Fig.3B,C** presents the distributions of the True-False differences for these groups of gates at 25 and 50 °C. It is evident that the True-False difference at 50 °C is substantially higher in the compensated system, whereas for the majority of control systems it is close to zero; that is, the states with and without input at 50 °C are nearly indistinguishable. Similarly, we performed the same analysis for the NOT gate. The results are presented in **Supplementary Fig.3E,F**, where the same pattern is observed. Thus, an appropriate system topology can ensure temperature stabilization even if its parameters are not optimized for operation at temperatures other than the reference one.*

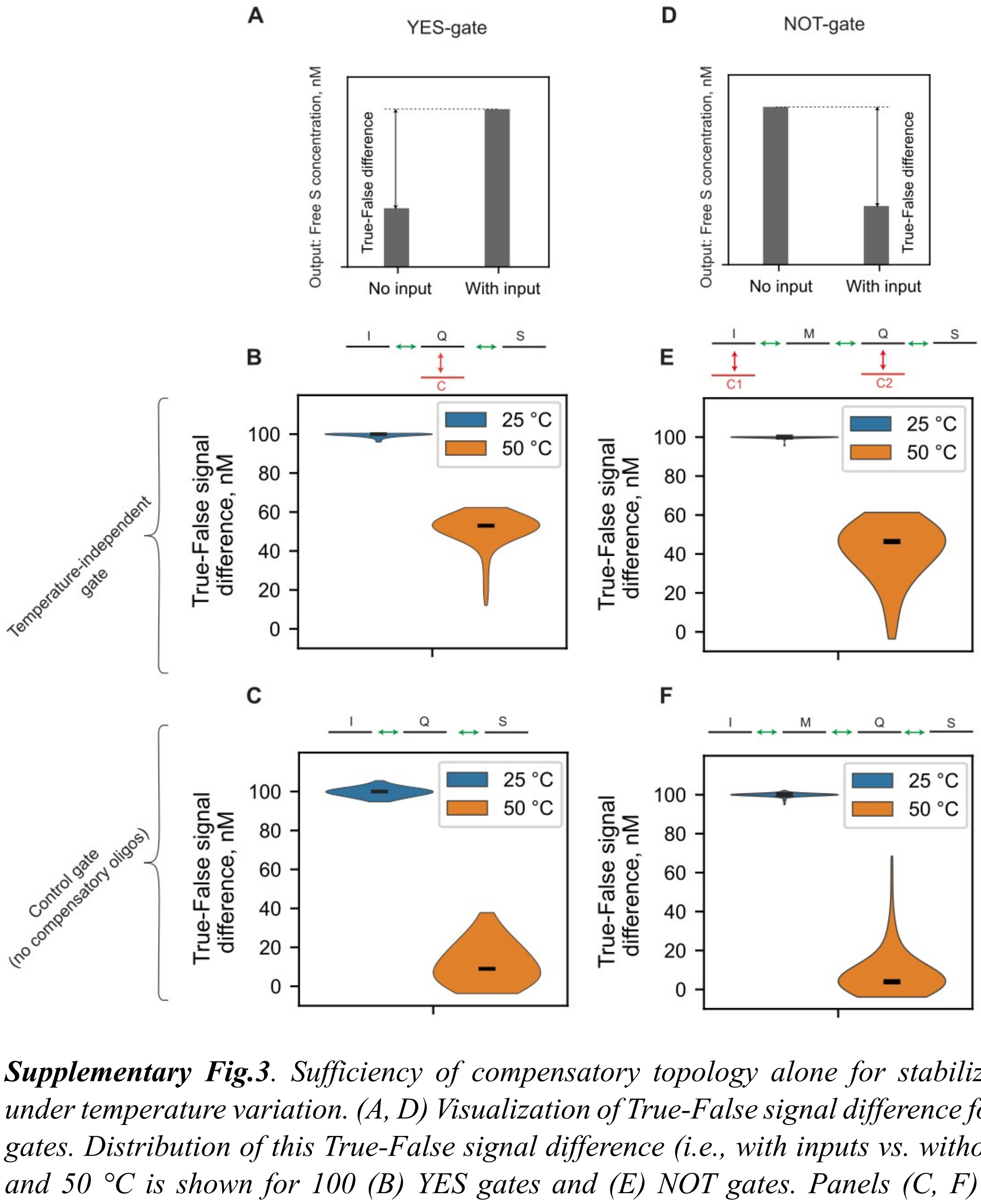

***Supplementary Fig.3**. Sufficiency of compensatory topology alone for stabilizing logic gates under temperature variation. (A, D) Visualization of True-False signal difference for YES and NOT gates. Distribution of this True-False signal difference (i.e., with inputs vs. without inputs) at 25 and 50 °C is shown for 100 (B) YES gates and (E) NOT gates. Panels (C, F) show the same*

distribution for the control YES and NOT gates (i.e., without compensatory oligos). Black lines show the median values. See Supplementary Note 2 for a detailed description."

3.10.In addition to that, placing this in a biological setting as done by the authors needs more care, not just because of the fine-tuning aspect, but also given that their treatment is restricted to equilibrium systems, while biological signaling usually works out-of-equilibrium.

The following section has been added to the Discussion to address this point:

"Applicability to non-equilibrium systems

An important aspect of the applicability of our mechanism to real biological systems is that many key biological processes operate under non-equilibrium conditions, whereas our work has focused exclusively on equilibrium systems. On the one hand, we do not see any fundamental limitations that would render such compensation infeasible in the kinetic regime, as one can expect the same or even greater diversity of temperature dependencies for kinetic rate constants. However, at present, there are no predictive tools for reaction rates comparable in maturity to NUPACK, which significantly complicates such investigations.

On the other hand, one of the key features of the low-affinity interaction regime is its intrinsic tendency toward fast kinetics[21]. Moreover, biological environments can further accelerate processes such as nucleic acid hybridization[40] through, for example, positively charged biopolymers, which reduce electrostatic repulsion between strands, or molecular crowding effects, which effectively increase local concentrations. Therefore, even if an entire complex system operates under non-equilibrium conditions, some of its subsystems may function on much faster timescales than the rest and can thus be treated as effectively at equilibrium with slowly varying external parameters."

3.11 Fig.1: "olgios" is a typo

Corrected.

3.12 Fig.3: "bioimaging" is not appropriate

Changed to "Fluorescent imaging".

3.13 Line 144: Missing the: "...the system behaves like a Boolean function YES: the logic output of this system is always the same as the input"

Corrected

3.14 It is great that the code is made available; a code availability statement is however missing. The code comes in the form of Jupyter notebooks and seems reasonable. It is great that the authors also supply the data and figures with it.

We thank the reviewer for the kind and encouraging comment. Code availability statement has been added:

"Code availability

The full source code is provided as a supplementary file ("source_code.zip")."

Reviewer #1 (Remarks to the Author):

The Authors have addressed most of my and other reviewers' comments and, based on the content of the manuscsipt, I would recommend publishing it in Nature Communications.

We thank the Reviewer for their positive assessment of our revised manuscript. We express our deepest appreciation to the Reviewer for the time and effort invested in the careful evaluation of our manuscript and for the Reviewer's valuable and constructive comments throughout the review process that helped us substantially improve the manuscript.

1.1 However, a major remaining issue is the style of presentation. I guess I would overstep my authority as a reviewer if start listing what I consider flaws in grammar and punctuation. I do see many of those and would strongly recommend an extensive technical editing before publishing this piece. Perhaps, it is a result of using an AI assistant acknowledged by the Authors. Even the new edition of the title is hard to parse.

We have carefully proofread the manuscript to identify and correct grammatical, punctuation, and stylistic issues, and have indeed found and addressed a substantial number of them (please, see the manuscript with tracked changes).

Regarding the title, we agree that it has become somewhat lengthy because it incorporates several key aspects of the study: temperature and ionic strength, independence and programmability, and the method by which these properties are achieved, namely molecular commutation networks.

We considered ways to shorten the title and concluded that the only element that can be removed is the "method" (i.e., molecular commutation networks). Removing any other elements could make the corresponding aspects of our work less discoverable in literature searches and could lead readers to incorrectly assume that they are not addressed within the study.

Therefore, we would suggest the following shortened version of the title "*Enabling temperature- and ionic strength-independent or programmably dependent molecular signaling*". However, if the Editorial team prefers the longer name, we would be happy to comply.

1.2"In the extreme case of long, fully complementary strands, the interaction becomes effectively irreversible, and complexes remain stable almost indefinitely at room temperature conditions37, which in practice corresponds to an effectively infinite operating time for the system." Operating time is a wrong term in this context: it usually means how long smth works before a failure.

Thank you for your comment, we have rewritten the sentence for clarity as follows:

"In the extreme case of long, fully complementary strands, the interaction becomes effectively irreversible[40], and complexes remain stable almost indefinitely under room-temperature conditions, making signal propagation mediated by such interactions prohibitively slow in practice."

1.3 Another suggestion is to tone down (or even better, remove) the discussion about the possibility to use elevated body temperature as signalling. Physically, that would be very inefficient due to the high heat capacity of body fluids (essentially water) and slow heat conduction. As far as I know, the mechanism of resetting the body thermostat is fairly well understood and is attributed to chemical signalling by a fever-inducing substance (pyrogen).

We are in a tricky position with this part of the discussion: while you (Reviewer #1) suggested to tone it down or remove it, Reviewer #3 stated that they especially liked it and even suggested to expand it by including some very interesting references not only related to fever but also to embryogenesis. After analysis, we understood that there was indeed certain ambiguity in our wording. Specifically, the statement that "*the elevated body temperature during disease can act as a universal information carrier to all cells of the organism*" could be interpreted as suggesting that temperature may serve not only as an input for our networks, as we intended, but also as an output, implying that our networks trigger changes in temperature. We definitely agree with the Reviewer that increase in temperature is well-studied and is generally attributed to pyrogens.

We greatly appreciate inputs of all Reviewers; therefore, we rephrased the piece to remove the ambiguity, and we ask to allow this excerpt as we believe it may be useful for the readership:

" To summarize, this remarkable feature of the molecular commutation networks allows them to receive a single cue of the environment (e.g., temperature or ionic strength) and react in many different ways. This phenomenon seems to be a perfect match for a hypothesis that the elevated body temperature during disease[44] can act as a universal signal to each cell of the organism to initiate its own unique response profile. Triggering a multitude of distinct programmable responses via a single physical input (temperature) may be highly valuable since it does not dependent on mass transport of molecular messengers throughout the body. Perhaps, such considerations may be also relevant for embryogenesis[45], in which changes in environmental temperature elicit differential responses across embryonic cells and give rise to temperature-induced phenotypes[46].

...

44. Evans, S. S., Repasky, E. A. & Fisher, D. T. Fever and the thermal regulation of immunity: the immune system feels the heat. Nat. Rev. Immunol. ***15****, 335–349 (2015).*

*45. Bourn, J. J. & Dorrity, M. W. Degrees of freedom: temperature's influence on developmental rate. Curr. Opin. Genet. Dev. **85**, 102155 (2024).*

*46. Dorrity, M. W. et al. Proteostasis governs differential temperature sensitivity across embryonic cell types. Cell **186**, 5015-5027.e12 (2023).*

"

1.4 Same suggestion applies to discussing the "evolutionary context". A necessary prerequisite for such a discussion is a well-established presense of those thermostabilizing mechanisms in the present life forms.

We agree with the reviewer that more caution is needed when discussing the evolution of a mechanism whose *in vivo* manifestations remain to be established.

At the same time, some evolutionary hypotheses, such as the RNA world hypothesis, have been proposed, discussed and considered to be valuable despite the fact that no RNA-only organisms are known to exist today. By analogy, we believe that some readers may find the discussion regarding the evolutionary potential of the proposed mechanism thought-provoking, even if such mechanism existed only in earlier life forms. We therefore believe that this idea is worth mentioning, at least in the Discussion section. However, to make it clear that the presence of this thermostabilizing mechanism in modern organisms has yet to be established, we have added the following sentence at the beginning of the section:

"*Although it is unclear whether this thermocompensation mechanism manifests in complex modern organisms, an intriguing possibility is that nature may have exploited this mechanism at much earlier stages of evolution."*

1.5 Perhaps a minor point that I missed preparing my previous review: can the authors comment on the actual rates of equilibration in their chemically-implemented "YES" gate? Such a long process seems virtually useless to buffer any important signalling pathway from, say, daily temperature variations.

This is indeed an important aspect of the study. During the first revision, we added kinetic measurements for both temperature- and salt-independent YES gates as Supplementary Figs. 8 and 12 (see below), which showed typical equilibration times of 1-30 min. However, in the present study we have not performed extensive studies of kinetics aspects, as it was already reported in the original Nature Chemistry paper (Nikitin, 2023) on molecular commutation. There it was shown, that logic-gates based on partially complementary oligonucleotides can exhibit highly diverse interaction kinetics, with timescales ranging from fraction of minutes to hours (see doi 10.1038/s41557-022-01111-y; Fig. 3d,f, Supplement Figs. 2-4, etc.)

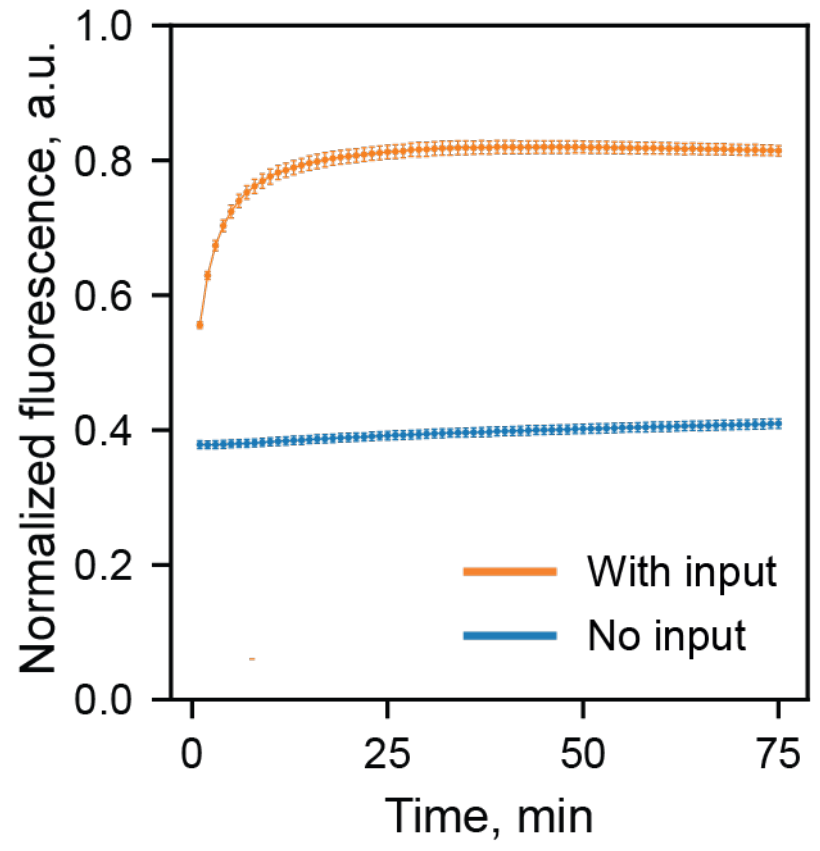

Supplementary Fig. 8. *Kinetics of the temperature-independent YES gate from Fig. 3C following the addition of the input oligonucleotide. Other components (S, Q, and C) were pre-equilibrated for 1 hour. Data are presented in relative fluorescence units as mean ± s.d., n = 3 independent samples. Fluorescence is normalized to the signal of a sample containing only S at the same concentration."*

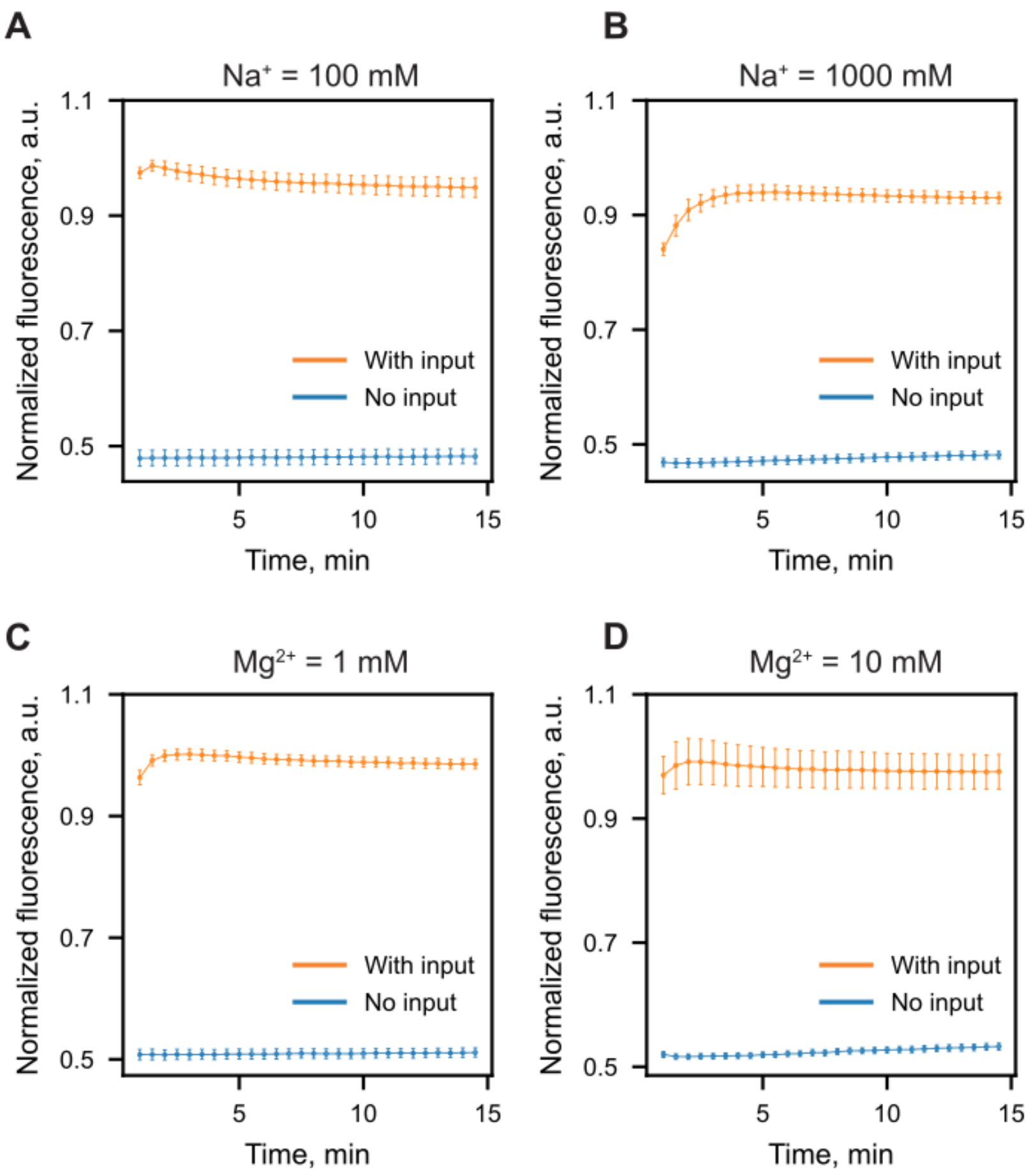

"***Supplementary Fig. 12***. *Kinetics of salt independent YES-gates from Fig. 4 following the addition of the input oligonucleotide. Other components (S, Q, and C) were pre-equilibrated for 1 hour. Data are presented in relative fluorescence units as mean ± s.d., n = 3 independent samples. Fluorescence is normalized to the signal of a sample containing only S at the same concentration. (A, B) show the gate performance optimized for robust operation under sodium variation, while (C, D) show the corresponding results for magnesium variation*"

Reviewer #2 (Remarks to the Author):

The authors have addressed all the issues I raised in my original review. I believe the paper is ready for publication.

We thank the Reviewer for their positive assessment of our revised manuscript. We are deeply grateful for the Reviewer's careful and meticulous assessment of the manuscript, for the time and expertise devoted to evaluating the code and data, and for the insightful and constructive suggestions that helped us substantially improve the manuscript.

Reviewer #3 (Remarks to the Author):

The authors have performed a very impressive revision, including many more theoretical and experimental results and responding in much detail to all comments by the three reviewers. We think that the question of novelty is very important, but now settled, because from the many results now contained in the revised manuscripts, some are certainly novel and it is a matter of context and community which exactly one wants to name here. It would not be entirely correct to say that earlier work on temperature compensation only considered enzymatic networks, or that the authors here consider affinity networks for the first time, or that biology has evolved exactly the same mechanisms as described here, but the new text is sufficiently balanced now that the readers see the complexity of and earlier answer to these questions. In general, the authors have done a very good job in explaining better what exactly has been achieved, including the rewritten title and abstract. This work will certainly appeal to many different communities and thus will find a broad readership, making it very appropriate for Nat Comms. The authors have also done a good job in integrating new references. We in particular like the comments on temperature as a universal signal during fever.

We thank the Reviewer for their positive assessment of our revised manuscript. We greatly appreciate the Reviewer's valuable and constructive comments throughout the review process, as well as their time and effort in conducting an in-depth analysis of the manuscript, particularly in the context of the related literature, which helped us substantially improve the manuscript and reframe its central ideas.

As a last comment, we note that the manuscript is relatively short on reviews. If more changes should be made (for example, the use of Kd/K_d could be homogenized in text and figures, and there are a few missing periods), the following reviews on temperature and fever could be included, it the authors find this appropriate:

Knapp, Benjamin D., and Kerwyn Casey Huang. "The effects of temperature on cellular physiology." Annual Review of Biophysics 51.1 (2022): 499-526.
Jacobs, Simen, et al. "Understanding the temperature response of biological systems: Part I-Phenomenological descriptions and microscopic models." Current Opinion in Systems Biology(2026): 100577.
Jacobs, Simen, et al. "Understanding the temperature response of biological systems: Part II-Network-level mechanisms and emergent dynamics." Current Opinion in Systems Biology(2026): 100578.

Bourn, Jess J., and Michael W. Dorrity. "Degrees of freedom: temperature's influence on developmental rate." Current Opinion in Genetics & Development 85 (2024): 102155.
Evans, Sharon S., Elizabeth A. Repasky, and Daniel T. Fisher. "Fever and the thermal regulation of immunity: the immune system feels the heat." Nature Reviews Immunology 15.6 (2015): 335-349.

We have homogenized the use of Kd/K_d and carefully identified and corrected grammatical, punctuation, and stylistic issues throughout the manuscript. Regarding the suggested references, we thank the reviewer for bringing them to our attention; they are indeed highly relevant to our manuscript and certainly worth citing. We especially appreciate the Reviewer's suggestion to include the review on temperature-dependent embryogenesis, which is a very interesting example of temperature-dependent signaling. We have incorporated all the suggested review articles as references 1, 6, 7, 44, and 45, as detailed below. In addition, the suggested preprints by Jacobs *et al.* have been replaced with the corresponding published journal articles:

Introduction:

"Almost all chemical reactions depend on temperature, typically following an exponential relationship of the form $e^{-\frac{E}{kT}}$*. This principle plays a critical role in biological systems, as even small variations in temperature can lead to significant changes in the parameters of biochemical reactions*[1]*, potentially resulting in severe physiological consequences at the organismal level.*

...

A question arises as to how, in all these cases, the complex regulatory and signaling networks of biochemical reactions, which form the basis of any living system, continue to properly function, given that the parameters of all the constituent reactions vary dramatically[6,7]*."*

"Programmable temperature/ionic strength dependence" section of Results:

"To summarize, this remarkable feature of the molecular commutation networks allows them to receive a single cue of the environment (e.g., temperature or ionic strength) and react in many different ways. This phenomenon seems to be a perfect match for a hypothesis that the elevated body temperature during disease[44] *can act as a universal signal to each cell of the organism to initiate its own unique response profile. Triggering a multitude of distinct programmable responses via a single physical input (temperature) may be highly valuable since it does not dependent on mass transport of molecular messengers throughout the body. Perhaps, such considerations may be also relevant for embryogenesis*[45]*, in which changes in environmental temperature elicit differential responses across embryonic cells and give rise to temperature-induced phenotypes*[46]*.*

References:

"1. Knapp, B. D. & Huang, K. C. The Effects of Temperature on Cellular Physiology. Annu. Rev. Biophys. 51, 499–526 (2022).

6. Jacobs, S., Voits, J. B., Frolov, N., Schwarz, U. S. & Gelens, L. Understanding the temperature response of biological systems: Part I–Phenomenological descriptions and microscopic models. Curr. Opin. Syst. Biol. 44, 100577 (2026).

7. Jacobs, S., Voits, J. B., Frolov, N., Schwarz, U. S. & Gelens, L. Understanding the temperature response of biological systems: Part II—network-level mechanisms and emergent dynamics. Curr. Opin. Syst. Biol. 44, 100578 (2026).

44. Evans, S. S., Repasky, E. A. & Fisher, D. T. Fever and the thermal regulation of immunity: the immune system feels the heat. Nat. Rev. Immunol. 15, 335–349 (2015).

45. Bourn, J. J. & Dorrity, M. W. Degrees of freedom: temperature's influence on developmental rate. Curr. Opin. Genet. Dev. 85, 102155 (2024)."

Reviewer #3 (Remarks on code availability):
Code availability has now been clarified. Github would still be good, but supplemental material is also fine.

We thank the reviewer for this comment. We agree that a GitHub repository would be useful for convenience.

However, the parts of the code were developed specifically in response to the peer-review process and directly reproduce results contained in the revised manuscript. We therefore prefer not to make the repository publicly accessible before the editorial decision, in order to avoid premature public disclosure of material generated during the confidential review process.

Thus, we have created a private GitHub repository for the code and included the repository link in the manuscript. The repository is currently private and will be made publicly accessible upon acceptance/publication, or earlier if suggested by the Editor. Nevertheless, the same code is still accessible as a supplementary material.

Corrected code availability statement:

*"**Code availability***

The full source code is provided as a Supplementary file ("source_code.zip") and is also available in a GitHub repository: github.com/korenkov-es/molec_comut_temp."
