# Supplementary material for: Enabling molecular signaling with temperature and ionic-strength independence or programmable dependence
Source: Nat Commun. 2026 Aug 7;17:8895. doi: 10.1038/s41467-026-76207-x (PMC13503822; doi:10.1038/s41467-026-76207-x)
Supplement: Supplementary file 4 [file 41467_2026_76207_MOESM4_ESM.zip › Source Data/readme.docx]

All source data are listed in corresponding .xlsx files. However, for programmatic access it is more convenient to use the .json files. We use a .json format that can be directly loaded with the NetworkX Python library. Each system is specified as a list of nodes and edges.

**Node parameters:**

- “Seq” – sequence from 5′ to 3′. If no sequence is provided, the system was simulated only at the level of affinity matrices and concentrations.
- “Conc” – species concentration in nM.
- “lb” and “ub” – lower and upper bounds of concentration (nM), used during PyBADS optimization.
- “id” – species name.
- “input” and “output” – indicate whether a node is treated as an input or output, respectively.

**Edge parameters:**

- “source” and “target” specify the “id” if the species that the edge describes.
- “G25” – Gibbs free energy (kJ/mol) at 25 °C, as optimized by PyBADS. **Note that this value does not correspond to the energy predicted by NUPACK.**
- “lb” and “ub” – lower and upper bounds of “G25” (kJ/mol), used during PyBADS optimization.
- “spl” – “easy-to-melt” level.

Other parameters are derived from these.

For receptor–activator networks, node parameters “activator” and “receptor” are used instead of “input” and “output”. For networks with misfolding, “f25” and “f50” denote the fraction of misfolded species at 25 °C and 50 °C, respectively.

**Histogram plots**

Please note that histogram data in all .xlsx files are provided as individual, unbinned observations rather than as precomputed bins with corresponding counts or frequencies.
